# A Unified Approach for the Synthesis of Conformationally Locked and sp^2^‐sp^3^ Fused Hybrids

**DOI:** 10.1002/chem.71359

**Published:** 2026-07-03

**Authors:** Ervis Saraci, Federica Plavi, Giovanni Viscardi, Chad N. Ungarean, Massimo Boiocchi, Christophe Salome, Quentin Lefebvre, Thomas C. Fessard, David Sarlah

**Affiliations:** ^1^ Department of Chemistry University of Pavia Pavia Italy; ^2^ Centro Grandi Strumenti University of Pavia Pavia Italy; ^3^ SpiroChem AG Basel Switzerland; ^4^ Department of Chemistry Rice University Houston Texas USA

**Keywords:** benzyne, bicycle, conformational restriction, diels‐alder, hybrids

## Abstract

Aromatics are indispensable structural motifs in pharmaceuticals due to conformational rigidity, unique noncovalent interactions, and stability, but they can also bear negative consequences related to poor solubility and metabolism. On the other hand, sp^3^‐hybridized structures address many of the deficiencies of aromatic counterparts. Based on this, we designed a systematic platform for the synthesis of hybrid sp^2^‐sp^3^ building blocks via benzyne cycloadditions, aiming to exploit a synergetic effect and explore new chemical space. The application of this strategy is demonstrated in the synthesis of Enalapril hybrid derivatives.

## Graphical abstract

This study presents a systematic platform to build rigid sp^2^–sp^3^ hybrids via benzyne cycloadditions. Arene‑fused bicyclic oxa‐, aza‐, and carbocycles are converted into medicinally relevant amino acids and applied to Enalapril analogues synthesis. The hybrids combine aromatic interactions with 3D rigidity, tuning lipophilicity, pKa, and solubility while expanding drug‑like chemical space.

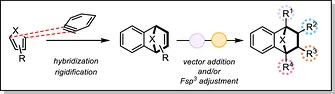

## 1 Introduction

The amalgamation of aryl and alicyclic components provides rigid, bicyclic structures, which are found in biologically active natural products, as seen in polyketide antibiotic sarubicin A (**1**), as well as isopavine alkaloid amurensinine (**2**, Figure 1a) [1, 2], and in other pharmaceuticals [3]. Taking inspiration from Nature, medicinal chemists have translated compounds containing this structural framework into pharmaceuticals, exemplified by the nicotine cessation treatment Chantix (**3,** Figure 1b). Bridged polycycles are privileged molecular skeletons that often lock molecules into specific conformations, enhancing selectivity and binding affinity when interacting with biological targets [4]. This makes them highly valuable in medicinal chemistry, where they are used to design drugs with improved metabolic stability and reduced off‐target effects [5]. Synthesizing bridged polycyclic structures is a captivating challenge in organic chemistry, given their rigid, 3D frameworks and biological relevance. The construction of these motifs has been a subject of interest in organic synthesis for decades, leading to the development of several strategies. Transition‐metal catalysis [6] and C‐H activation [7] are among the most widely used strategies in carbon‐carbon bond formation, but in recent years many elegant alternatives have emerged, including cycloadditions and rearrangements [8]. Dearomatization has emerged as a flourishing synthetic tool for the construction of complex scaffolds [9, 10, 11, 12, 13, 14, 15, 16, 17, 18, 19], and it is a valuable strategy that makes use of simple and available aromatics [20]^.^ Recently, metallaphotoredox C(sp^2^)–C(sp^3^) cross‐coupling has been used in combination with radical cyclization to assemble a variety of bridged ring systems that would be extremely difficult to make by conventional methods [21]. Despite these progresses, known methods present some limitations, including substrate‐specificity and long synthetic pathways, and an efficient and systematic approach to forge these hybrid systems remain highly desirable. With the underexplored yet seemingly promising prospective for this structural class, we imagined the identification and modification of known drug candidates (e.g. Enalapril **4**) that may benefit from arene incorporation and concomitant structural rigidification (Figure 1c). We considered designing a strategy to systematically generate rigid, bicyclic sp^2^‐sp^3^ hybrids for drug retrofitting. In our retrosynthetic evaluation, we deemed the cycloaddition of unsaturated 5‐membered (hetero)cycles (furans, pyrroles, and fulvenes) with benzyne as a rapid and convenient disconnection (Figure 1d).

**FIGURE 1:**
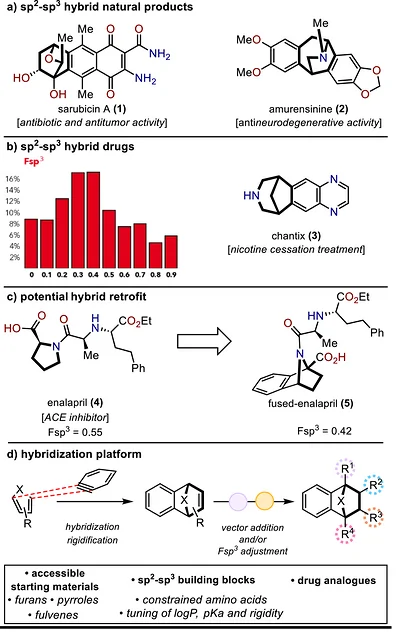
(a‐b) Examples of natural products and drugs containing sp^2^‐sp^3^ hybrid structures. (c) Selected candidate for the hybrid retrofit. (d) General platform to obtain hybrid building blocks.

Aryne‐based reactions have been employed in a variety of total synthesis and methodologies; [22, 23] among arynes, benzyne is prevalent due to its versatility and ease of synthesis. Various methods have been developed for its synthesis, and it exhibits different reactivities due to the unusually low‐lying LUMO [24]. The unique nature of the formal triple bond endows benzyne with the ability to undergo various pericyclic reactions. Concerning the [4+2] cycloaddition, dienes such as simple furans and pyrroles have been reported to react smoothly with benzyne, generating benzonorbornadienes structures [25]. These scaffolds are important intermediates in organic synthesis due to the strain of the bicyclic system and uniquely activated allylic/benzylic heteroatom functionality. For these reasons, most of their chemistry relies on the ring‐opening and/or olefin functionalization. Various nucleophiles have been used in transition metal‐catalyzed ring opening reactions: amine nucleophiles under Pd [26] and Rh [27] catalysis, as well as alkynes [28] and simple arenes [29]. On the other hand, bicyclic olefin can be engaged in cycloaddition reaction [30, 31] or simple addition of small groups such as amine [32], carbon [33], and silicon nucleophiles [34].

Our methodological approach could effectively generate differentially functionalized arene‐fused [2.2.1]bicyclic compounds, as well as several hybrid drug analogues, while retaining the benzonorbornadiene scaffold and introducing different exit vectors on the saturated portion. This key strategic design feature rests on the notion that a synergistic effect between the two fragments will prove beneficial [35]. Specifically, inclusion of aromatic cores may provide critical aromatic features (i.e. noncovalent interactions) while detrimental properties in drug design including poor metabolic stability and solubility can be ameliorated by the conjoined alicyclic fragment [36]. Furthermore, the consequential positive effects embedded in the synthesized compounds include structural rigidification [37] and Fsp^3^ carbon atoms [38], which have both been well documented to contribute to successful campaigns in medicinal chemistry.

## 2 Results and Discussion

We began our work by evaluating a suitable method for generating and trapping benzyne using ethyl 2‐furoate **6**. Fortuitously, the extensive chemical toolkit for benzyne generation provided ample options for identifying successful conditions that could be translated to our system [39, 40]. After initial screening (see Supporting Information), the oxidative decomposition of anthranilic acid in the presence of isoamylnitrite [41] was selected for our synthetic purposes based on several advantages. First, readily available anthranilic acid enabled a scalable and practical approach. Second, we were intrigued by the ability to generate benzyne at higher temperatures, as it is well known that benzyne cycloadditions with our substrates require additional thermal energy [42]. With these intermediates in hand, we geared our attention to the synthesis of oxabicyclo[2.2.1]heptane‐benzene hybrids. Subjection of either **6** or **7** to our standard conditions generated the corresponding bicycles **11** and **12**, respectively (Scheme 1). The separately formed constitutional isomers could both undergo hydrogenation to saturate the cyclic alkene, followed by hydrolysis to obtain the oxa‐bridged hybrid carboxylic acids **13** and **14**. Notably, the latter was obtained as a single diastereoisomer, which was confirmed using x‐ray analysis after derivatization of carboxylic acid (Compound **S4**, See Supporting Information).

**SCHEME 1:**
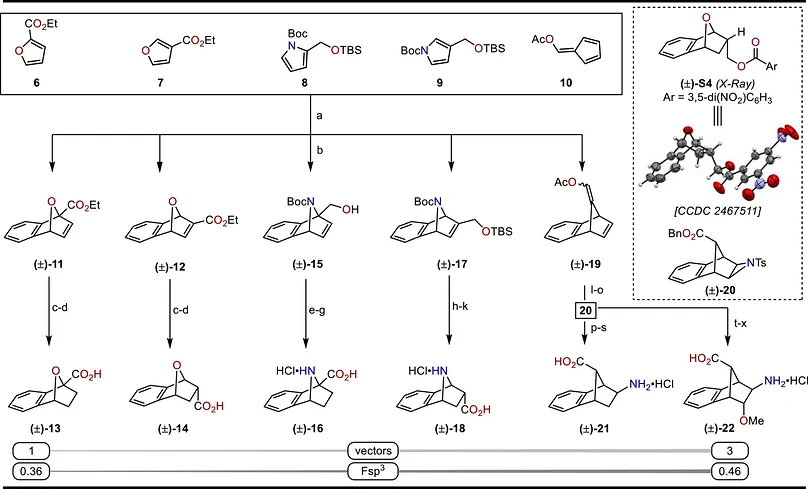
*Reactions and conditions*: (a) Anthranilic acid (2 equiv), *i*AmONO (2 equiv), THF, 3 h, reflux, 92% for **11**, 65% for **12**, 70% for 15, 63% for **17**, 65% for **19**; (b) TBAF (1.5 equiv), THF, 6 h, rt, 90%; (c) H_2_ (1 atm), Pd/C (0.1 equiv), EtOAc, 18 h, rt; (d) NaOH (2 equiv), EtOH, 18 h, rt, 71% for **13** (2 steps), 77% for **14** (2 steps). (e) H_2_ (1 atm), Pd/C (0.1 equiv), MeOH, 18 h, rt, 93%; (f) TPAP (0.1 equiv), NMO (5 equiv), MeCN, 12 h, rt, 76%; (g) HCl (4 M in Dioxane, 20 equiv), DCM, 18 h, rt, 76%; (h) H_2_ (1 atm), Pd/C (0.1 equiv), MeOH, 18 h, rt, 92%; (i) TBAF (1.5 equiv), THF, 6 h, rt, 94%; (j) TPAP (0.1 equiv), NMO (10 equiv), MeCN, 18 h, rt, 85%; (k) HCl (4 M in Dioxane, 20 equiv), DCM, 18 h, rt, 76%. (l) K_2_CO_3_ (2 equiv), MeOH, 12 h, rt, 98%; (m) NaClO_2_ (10 equiv), NaH_2_PO_4_ (15 equiv), 2‐methyl‐2‐butene (30 equiv), *t*BuOH:H_2_O, 12 h, rt, 91%; (n) BnBr (1.2 equiv), K_2_CO_3_ (2 equiv), DMF, 18 h, rt, 88%; (o) PhINTs (1.2 equiv), [Cu(MeCN)_4_]OTf (0.1 equiv), MeCN, 24 h, rt, 38%; (p) H_2_ (1 atm), Pd/C (0.1 equiv), EtOAc, 24 h, rt, 94%; (q) HClO_4_ (1.3 equiv), *t*BuOAc, 18 h, rt, 83%; (r) Boc_2_O (3.5 equiv), Et_3_N (3 equiv), DCM, 12 h, rt then Mg (20 equiv), MeOH, 24 h, rt, 72%; (s) HCl (4 M in Dioxane, 20 equiv), DCM, 24 h, rt, 99%; (t) Mg (20 equiv), MeOH, 24 h, rt, 74%; (u) Boc_2_O (1.5 equiv), Et_3_N (2 equiv), DCM, 12 h, rt, 92%; (v) H_2_ (1 atm), Pd/C (0.1 equiv), MeOH, 24 h, rt, 70%; (x) HCl (4 M in Dioxane, 20 equiv), DCM, 24 h, rt, 82%. DCM = dichloromethane; *i*AmONO = isoamyl nitrite; MeCN = Acetonitrile; NMO = *N*‐methylmorpholine *N*‐oxide; TPAP = tetrapropylammonium perruthenate; TBAF = Tetrabutylammonium fluoride; THF = tetrahydrofuran.

With a pair of oxabicyclo[2.2.1]heptanes in hand, we shifted our focus to synthesize a series of benzene‐fused azabicyclo[2.2.1]heptane structures through the employment of pyrroles for the benzyne cycloaddition. Starting from known bicycle **15** [43], reduction of the cyclic alkene through hydrogenation followed by Ley–Griffith oxidation furnished the corresponding saturated carboxylic acid. Finally, acid‐mediated *tert*‐butoxycarbonyl (Boc) removal yielded bicyclic α‐amino acid HCl salt **16**. A similar strategy was used to obtain the β‐amino acid derivative. Silyl ether **9** (see Supporting Information) was subjected to benzyne conditions to construct **17**. Hydrogenation of the bicyclic, trisubstituted alkene and silyl deprotection gave primary alcohol **S9**, which was oxidized to the corresponding acid and treated with HCl to provide derivative **18** as a single diastereoisomer.

Finally, we explored the feasibility of accessing carbon‐based bridged compounds by using fulvenes as partners for the cycloaddition. Acetoxy pentafulvene **10** [44] was selected, as we sought to use the acetoxy group as a handle for an additional vector. Cycloaddition proceeded, albeit in moderate yield, to afford the hybrid structure **19**. The enol ether underwent basic hydrolysis to the known aldehyde [45], which was thereafter subjected to Pinnick oxidation to provide an inseparable mixture of carboxylic acid diastereoisomers. This mixture was advanced and esterified to a benzyl ester, which was then subjected to copper‐catalyzed aziridination using the nitrene precursor PhINTs [46]. Fortuitously, only the minor isomer reacted, which allowed for the isolation of Ts‐aziridine **20** [47]. Opening of aziridine with hydrogen and Pd/C also deprotected the corresponding benzyl ester, which was reprotected as a *tert*‐butyl ester. Effective removal of the Ts group proved challenging and required a one‐pot Boc‐protection, followed by Mg‐based detosylation to yield protected amino acid. Finally, the desired γ‐amino acid **21** was obtained after Boc‐deprotection. Alternatively, intermediate **20** could be taken forward first performing a protecting group swap to Boc‐protected amine. Surprisingly, we found that hydrogenation in MeOH facilitated the nucleophilic addition of MeOH to the aziridine [48]. Following Boc‐deprotection, the functionalized γ‐amino acid **22** was obtained (Scheme 1).

Following the success in generating a small selection of bicyclic carboxylic and amino acids, we further applied these building blocks to the synthesis of hybrid analogues of pharmaceuticals. We envisioned an Enalapril analogue as a suitable candidate for study based on the proline‐derived core. Enalapril (**4**) serves as an important cardiovascular drug [49], as it blocks the conversion of angiotensin I to the potent vasoconstrictor angiotensin II and inactivates bradykinin [50]. Our synthesis required the amidation of amine **28** (ultimately derived from acid **S6**, see Supporting Information) with anhydride **27** (See Supporting Information for experimental details). These fragments were successfully coupled to yield a mixture of separable diastereomers. Each diastereoisomer was individually subjected to benzyl ester deprotection using hydrogenation conditions (Scheme 2b) to yield the final fused‐Enalapril analogues **5A** and **5B**, whose structures were confirmed by x‐Ray analysis after derivatization (See Supporting Information).

**SCHEME 2:**
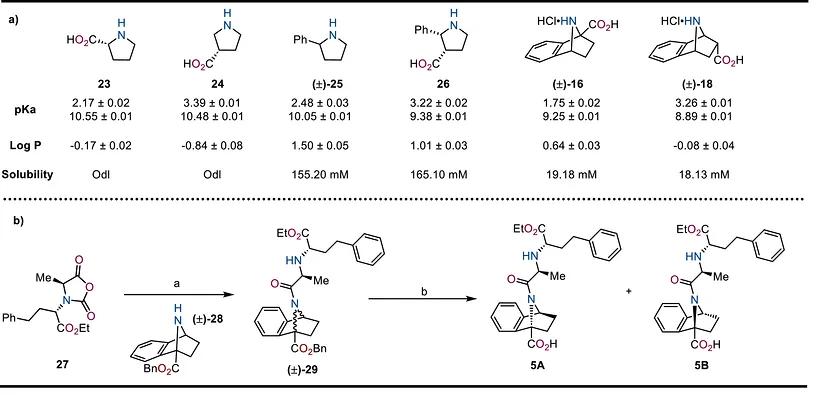
(a) Physicochemical measurements of proline‐based compounds and hybrid analogues. (b) Synthesis of Enalapril analogues. *Reactions and conditions*: (a) **27** (2 equiv), **28** (1 equiv), DCM, 48 h, rt, 92%, dr 1:1; (b) H_2_ (1 atm), Pd/C (0.1 equiv), MeOH, 18 h, rt, 93% for **5A**, 94% for **5B**. DCM = dichloromethane; Odl = Over detection limit.

Hybrid amino acids were analyzed, measuring different physicochemical properties; in particular, *p*K_a_, LogP, and solubility were determined, and benchmarked with those of commercial proline derivatives (Scheme 2a). Comparing compounds **16**, **23**, **25** on one side and **18**, **24**, **26** on the other side (hybrid, nonaromatic prolines and substituted prolines), the *p*K_a_’s are similar, with the second *p*K_a_ slightly higher for nonaromatic compounds. As expected, hybrid derivatives are less lipophilic than the substituted analogues. Finally, hybrids are less soluble than the substituted prolines by an order of magnitude, despite being less lipophilic, probably because they exert more ordered molecular packing.

## 3 Conclusion

In conclusion, a rapid and convenient method for the synthesis of sp^2^‐sp^3^‐fused building blocks has been developed, as well as the use of these derivatives in the synthesis of drug analogues. Specifically, we demonstrated how pyrrole‐ and furan‐derived benzyne cycloadducts can be systematically converted into more medicinally relevant building blocks. This study should further enable access and research to these highly valued building blocks and unique sp^2^‐sp^3^‐fused chemical space.

## Conflicts of Interest

The author declare no conflicts of interest

## Supporting information




**Supporting file**: The Supporting Information contains extensive further experimental and spectroscopic detail beyond the above example of a typical procedure. The authors have cited additional references within the Supporting Information [51, 52, 53, 54, 55, 56, 57, 58].

## Data Availability

The data that supports the findings of this study are available in the supplementary material of this article.
