# Supplementary material for: A Unified Approach for the Synthesis of Conformationally Locked and sp2‐sp3 Fused Hybrids
Source: Chemistry. 2026 Jul 3;32(38):e71359. doi: 10.1002/chem.71359 (PMC13660500; doi:10.1002/chem.71359)
Supplement: Supplementary file 1 [file CHEM-32-e71359-s001.pdf]

A Unified Approach for the Synthesis of Conformationally Locked and sp^2-sp^3 Fused Hybrids

Ervis Saraci, Federica Plavi, Giovanni Viscardi, Chad N. Ungarean, Massimo Boiocchi, Christophe Salome, Quentin Lefebvre, Thomas C. Fessard,* and David Sarlah*

Supporting Information

Table of Contents

General Experimental 5

Experimental Procedures 6

Optimization of benzyne Diels-Alder cycloaddition 6

General Procedure for Diels-Alder cycloaddition using anthranilic acid A: 7

General Procedure for Diels-Alder cycloaddition using benzyne precursor B: 7

Synthesis of oxabicyclo[2.2.1]heptane derivatives 8

Oxa-Bicyclic ester **S1** 8

Oxa-Bicyclic acid **13**. 8

Oxa-Bicyclic ester **S2**. 9

Oxa-Bicyclic acid **14**. 10

Oxa-Bicyclic alcohol **S3**. 10

Oxa-Bicyclic benzoate **S4**. 11

Synthesis of azabicyclo[2.2.1]heptane derivatives 12

Aza-Bicyclic alcohol **S5**. 12

Aza-Bicyclic acid **S6**. 12

Aza-Bicyclic hydrochloride salt **16**. 13

Pyrrole **9**. 14

Aza-Bicyclic silyl ether **17** 15

Aza-Bicyclic silyl ether **S8**. 15

Aza-Bicyclic alcohol **S9**. 16

Aza-Bicyclic acid **S10**. 16

Aza-Bicyclic hydrochloride salt **18**. 17

Synthesis of fulvene-based bicyclic derivatives 18

Bicyclic acid **S12**. 18

Bicyclic benzyl ester **S13**. 19

Bicyclic tosyl aziridine **20**. 20

Bicyclic acid **S14**. 21

Bicyclic tert-butyl ester **S15**. 21

Bicyclic Boc-amine **S16**. 22

Bicyclic hydrochloride salt **21**. 22

Bicyclic free aziridine **S17**. 23
Bicyclic Boc-aziridine **S18**. 24
Bicyclic acid **S19**. 24
Bicyclic hydrochloride salt **22**. 25
Synthesis of Enalapril bicyclic derivatives 26
Aza-bicyclic benzyl ester **S20**. 26
Aza-bicyclic benzyl ester **28**. 27
Enalapril Bicyclic Benzyl ester **29**. 27
Diastereomer **29A** 28
Diastereomer **29B** 28
Enalapril Bicyclic analogue **5A**. 29
Enalapril Bicyclic analogue **5B**. 29
Enalapril Bicyclic Analogue Derivatization **S22**. 30
PhysChem Analysis 31
pKa Measurement 31
Log P/Log D Measurement 31
Solubility Measurement 31
Results 32
Crystallographic Data 33
^{1}H and ^{13}C NMR spectra 36
S1 36
13 38
S2 40
14 42
S3 44
S4 46
S5 48
S6 50
16 52
9 54
17 56
S8 58

S9 60
S10 62
18 64
S12 66
S13 68
20 70
S13 – major isomer 72
S14 77
S15 79
S16 85
21 87
S17 89
S18 91
S18 93
22 95
S20 97
28 99
29A 101
29B 103
5A 105
5B 107
S22 109
References 111

General Experimental

All chemicals were purchased from commercial suppliers and used as received, unless otherwise noted. Diethyl ether (ACS grade), dichloromethane (ACS grade), tetrahydrofuran (HPLC grade), and toluene (ACS grade) were dried for reactions using the MB-SPS solvent purification system containing activated alumina manufactured by MBRAUN. Unless otherwise noted, all reactions were carried out under an ambient atmosphere. Analytical thin-layer chromatography (TLC) was performed on Merck silica gel 60 F254 aluminum sheets. Visualization was accomplished with UV light and/or potassium permanganate ($KMnO_4$), Ninhydrin and Vanillin stains. Retention factor (R_f) values reported were measured using a 5 × 2 cm TLC plate in a developing chamber containing the solvent system described. Silicycle SiliaFlash® P60 (SiO_2, 40–63 μm particle size, 230–400 mesh) was used for flash column chromatography. ^{1}H NMR spectra were obtained at 400 MHz, ^{13}C NMR were obtained at either 101 MHz, NMR spectra were recorded using a Bruker 400 MHz spectrometer and were referenced to residual chloroform (7.26 ppm, ^{1}H; 77.16 ppm, ^{13}C), residual acetonitrile (1.94 ppm, ^{1}H; 118.26 ppm, ^{13}C), residual methanol (3.31 ppm, ^{1}H; 49.00 ppm, ^{13}C), residual dimethylsulfoxide (2.50 ppm, ^{1}H; 39.52 ppm, ^{13}C) or residual H_2O (4.76 ppm, ^{1}H). Chemical shifts are reported in parts per million (ppm) and multiplicities are indicated as: s (singlet), d (doublet), t (triplet), q (quartet), p (pentet), m (multiplet), and br (broad). Coupling constants, *J*, are reported in Hertz. Mass spectrometry (MS) was performed by the University of Pavia. Electrospray ionization (ES+) spectra were performed using a time-of-flight (TOF) mass analyzer. Data are reported in the form of m/z. Infrared (IR) spectra were measured neat on a Perkin-Elmer Spectrum Two FT-IR ATR spectrometer. Peaks are reported in cm^{-1}. Melting points were measured on a Buchi B-540 melting point apparatus and are uncorrected. Specific optical rotation was measured using a Jasco P-200 Polarimeter. PhysChem analysis was performed using a Sirius T3 automated pH-metric titrator.

Experimental Procedures

Optimization of benzyne Diels-Alder cycloaddition

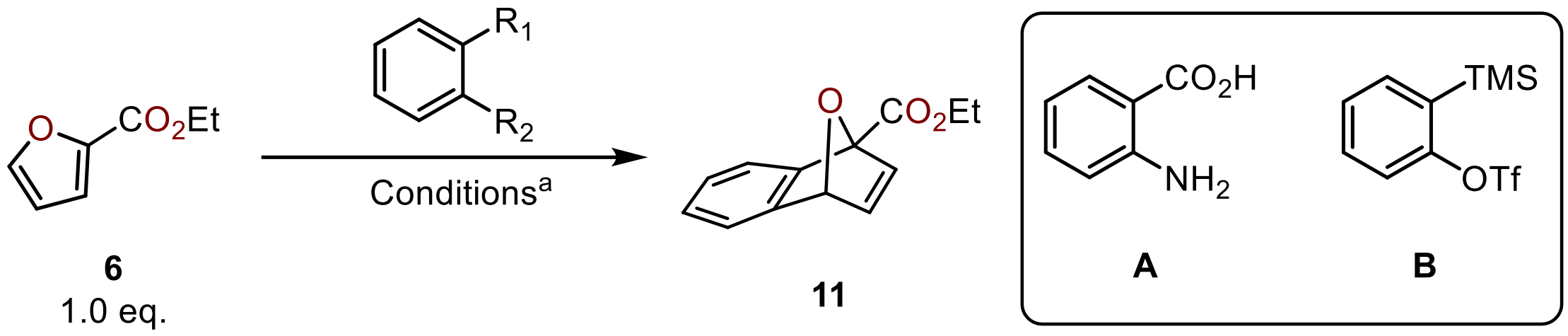

Entry	Benzyne Source [eq.]	Additive [eq.]	Solvent	Additions Time [h]	Temp. [°C]	Yield [%][c]
1	B [2.0]	CsF [2.0]	MeCN	/	25	0
2	B [4.0]	CsF [4.0]	MeCN	/	25	0
3[b]	B [1.0]	CsF [1.0]	MeCN	/	25	0
4	B [2.0]	CsF [2.0]	MeCN	/	50	38
5	B [2.0]	CsF [2.0]	MeCN	/	70	69
6	B [3.0]	CsF [5.0]	MeCN	/	70	75
7	A [2.0]	*i*AmONO [2.0]	THF	1	60	25
8	A [2.0]	*i*AmONO [2.0]	DCE	1	60	12
9	A [2.0]	*i*AmONO [2.0]	CH_2Cl_2/THF	1	50	0
10	A [2.0]	*i*AmONO [2.0]	DCE	1	70	21
11	A [2.0]	*i*AmONO [2.0]	DCE	1	80	46
12	A [2.0]	*i*AmONO [2.0]	DCE	2	80	51
13	A [2.0]	*i*AmONO [2.0]	DCE	4	80	52
14	A [2.0]	*i*AmONO [2.0]	THF	2	80	72
15	A [2.0]	*i*AmONO [2.0]	THF	2	100	69
16	A [4.0]	*i*AmONO [4.0]	THF	4	70	80
17	A [4.0]	*i*AmONO [4.0]	THF	8	70	82
18	A [3.0]	*i*AmONO [3.3]	THF	2	70	88
19[d]	A [3.0]	*i*AmONO [3.3]	THF	2	70	85

[a.] Reaction performed on a 1 mmol scale. [b.] 2.0 eq. of furan used. [c.] Isolated yields are reported. [d.] Reaction performed on 10 mmol scale.

Table S1. Optimization of benzyne Diels-Alder cycloaddition.

General Procedure for Diels-Alder cycloaddition using anthranilic acid A:

Solutions of anthranilic acid A (0.5 M) and isopentyl nitrite (0.5 M) were added dropwise and simultaneously to a refluxing solution of furan (1.0 eq.) in the appropriate solvent (1 M) over the appropriate period. The solution was refluxed for further 2 h, it was cooled to room temperature, aqueous sodium bicarbonate was added. The organic solvent was evaporated, and the aqueous phase was extracted with Et_2O. The organic layers were combined, washed with brine, dried over Na_2SO_4, and evaporated under reduced pressure. The residue was purified via flash column chromatography on silica gel to afford the corresponding known cycloadduct [1].

General Procedure for Diels-Alder cycloaddition using benzyne precursor B:

Benzyne precursor B was synthesized using a known procedure [2]. Cesium fluoride was added in one portion to a solution of the corresponding furan (1.00 equiv) in MeCN (0.1 M) at room temperature. The reaction mixture was heated to 70 °C and a solution of **B** in MeCN (0.1 M) was added dropwise. Upon complete addition, the reaction mixture was stirred for 16 hours, and it was allowed to cool to room temperature. Aqueous sodium bicarbonate was added, the organic solvent was evaporated, and the aqueous phase was extracted with Et_2O. The organic layers were combined, washed with brine, dried over Na_2SO_4, and evaporated under reduced pressure. The residue was purified via flash column chromatography on silica gel to afford the corresponding cycloadduct.

Synthesis of oxabicyclo[2.2.1]heptane derivatives

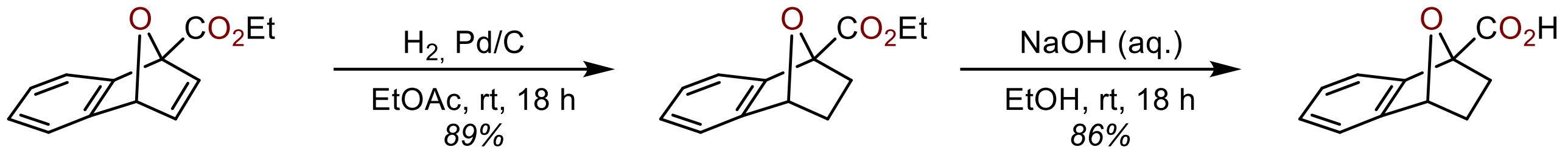

11 S1 13

Scheme S1. Synthesis of carboxylic acid **13.**

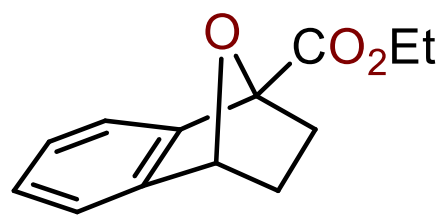

S1

Oxa-Bicyclic ester S1. Unsaturated cycloadduct **11** (1.00 g, 4.62 mmol, 1.0 eq.) was solubilized in 45 mL of EtOAc (0.1 M). The mixture was purged with Ar, then Pd/C (10% Pd on C, 490 mg, 0.10 eq.) was added to the mixture. The flask was filled with H_2 gas using a double balloon, and the reaction mixture was stirred for 18 h at room temperature under an atmosphere of H_2. Once complete, the mixture was filtered over a pad of celite, using EtOAc as the eluent. The solvent was evaporated under reduced pressure, and the residue was purified via flash column chromatography on silica gel (Hexane:Ethyl Acetate = 9:1) to afford the corresponding saturated cycloadduct **S1** (0.90 g, 89%) as a white solid.

R_f = 0.26 (SiO_2, Hex:EtOAc 9:1, UV, $KMnO_4$)

^{1}H NMR (400 MHz, $CDCl_3$) δ 7.57 – 7.55 (m, 1H), 7.37 – 7.27 (m, 3H), 5.57 (d, J = 4.7 Hz, 1H), 4.56 – 4.47 (m, 2H), 2.38 – 2.26 (m, 2H), 1.88 – 1.82 (m, 1H), 1.55 (ddd, J = 9.9, 8.6, 2.9 Hz, 1H), 1.49 (t, J = 7.1 Hz, 3H).

^{13}C NMR (101 MHz, $CDCl_3$) δ 169.57, 144.88, 143.30, 127.31, 126.79, 118.97, 118.93, 86.36, 79.04, 61.57, 31.16, 27.93, 14.30.

HRMS: (ESI-TOF, m/z) $[M+Na]^+$ calcd. for $C_{13}H_{14}O_3$ m/z 241.0835 and found m/z 241.0824.

IR: (ATR, neat, cm^{-1}) = 1744, 1436, 1370, 1343, 1317, 1261, 1220, 1127, 1105, 1017, 956.

m.p. = 60 – 63 °C.

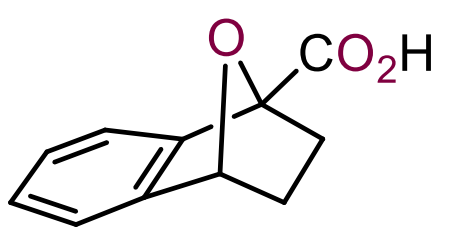

13

Oxa-Bicyclic acid 13. Ester-substituted cycloadduct **S1** (200 mg, 0.92 mmol, 1.0 eq.) was solubilized in 4 mL of EtOH (0.2 M) under an Ar atmosphere. An aqueous solution of NaOH (1 M, 1.83 mL, 2.0 eq.) was added to the mixture. The reaction mixture was stirred for 18 h at room temperature under an atmosphere of Ar. Once complete, the mixture was diluted with EtOAc and acidified with 1 M HCl. The aqueous layer was extracted with EtOAc. The organic layers were combined, washed with brine, dried over Na_2SO_4, and evaporated under reduced pressure. The residue was purified via flash column chromatography on silica gel (Hexane:Ethyl Acetate = 7:3) to afford the corresponding carboxylic acid **13** (150 mg, 86%) as a white solid.

R_f = 0.18 (SiO_2, Hex:EtOAc 8:2, UV, $KMnO_4$)

^{1}H NMR (400 MHz, CD_3CN) δ 7.45 – 7.43 (m, 1H), 7.33 – 7.30 (m, 1H), 7.26 – 7.20 (m, 2H), 5.47 (d, J = 4.7 Hz, 1H), 2.23 – 2.11 (m, 2H), 1.68 – 1.62 (m, 1H), 1.43 – 1.38 (m, 1H).

^{13}C NMR (101 MHz, CD_3CN) δ 170.96, 146.29, 144.56, 128.23, 127.67, 120.08, 119.54, 87.06, 79.70, 31.71, 28.55.

HRMS: (ESI-TOF, m/z) $[M+Na]^+$ calcd. for $C_{11}H_{10}O_3$ m/z 213.0522 and found m/z 213.0515.

IR: (ATR, neat, cm^{-1}) = 3498, 1683, 1344, 1325, 1277, 1207, 1167, 1140, 1079, 1043.

m.p. = 100 – 102 °C.

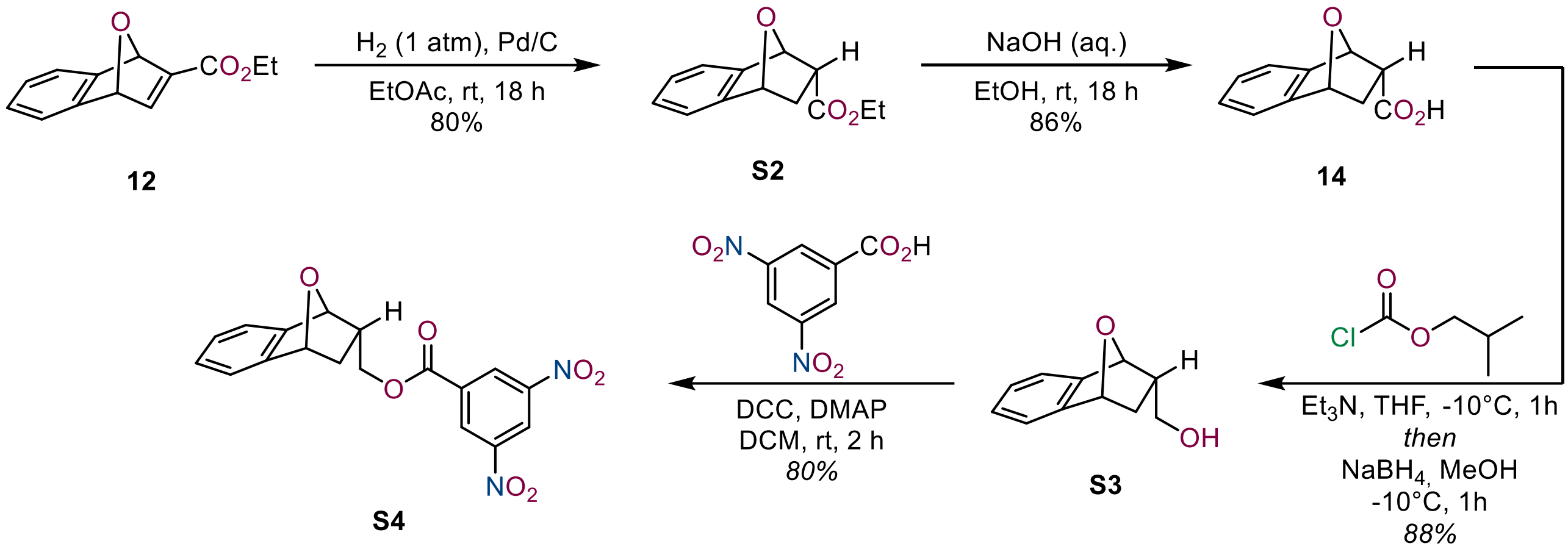

Scheme S2. Synthesis of carboxylic acid **14**.

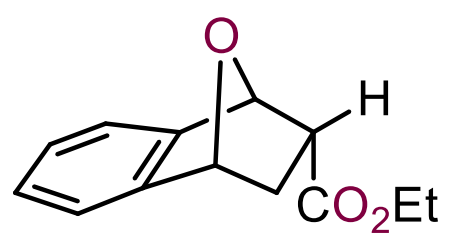

S2

Oxa-Bicyclic ester S2. Cycloadduct **12** was synthesized according to a known procedure [3]. Due to its instability, it was used without any purification as a crude product. Unsaturated cycloadduct **12** (500 mg, 2.31 mmol, 1.0 eq.) was solubilized in 23 mL of EtOAc (0.1 M). The mixture was purged with Ar, then Pd/C (10% Pd on C, 246 mg, 0.10 eq.) was added to the mixture. The flask was filled with H_2 gas using a double balloon, and the reaction mixture was stirred for 18 h at room temperature under an atmosphere of H_2. Once complete, the mixture was filtered over a pad of celite, using EtOAc as the eluent. The solvent was evaporated under reduced pressure, and the residue was purified via flash column chromatography on silica gel (Hexane:Ethyl Acetate = 9:1) to afford the corresponding saturated cycloadduct **S2** (405 mg, 80%) as a white solid, and as a single isomer.

R_f = 0.35 (SiO_2, Hex:EtOAc 9:1, UV, $KMnO_4$)

^{1}H NMR (400 MHz, $CDCl_3$) δ 7.28 – 7.26 (m, 1H), 7.21 – 7.12 (m, 3H), 5.56 (d, J = 5.3 Hz, 1H), 5.43 (d, J = 5.0 Hz, 1H), 3.96 (q, J = 7.1 Hz, 2H), 3.39 (ddd, J = 9.7, 5.3, 3.9 Hz, 1H), 2.32 (ddd, J = 11.6, 10.1, 5.1 Hz, 1H), 1.82 (dd, J = 11.8, 3.9 Hz, 1H), 1.17 (t, J = 7.1 Hz, 3H).

^{13}C NMR (101 MHz, $CDCl_3$) δ 171.15, 145.75, 141.95, 127.49, 126.58, 120.67, 118.93, 80.13, 79.99, 60.69, 45.08, 30.57, 14.30.

HRMS: (ESI-TOF, m/z) $[M+Na]^+$ calcd. for $C_{13}H_{14}O_3$ m/z 241.0835 and found m/z 241.0828.

IR: (ATR, neat, cm^{-1}) = 1723, 1459, 1332, 1218, 1187, 1058, 851, 765.

m.p. = 65 – 67 °C.

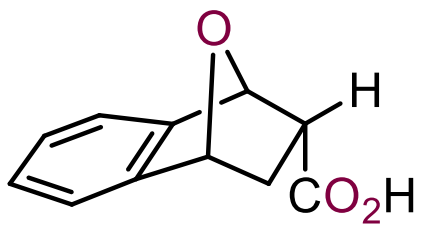

14

Oxa-Bicyclic acid 14. Ester-substituted cycloadduct **S2** (200 mg, 0.92 mmol, 1.0 eq.) was solubilized in 4 mL of EtOH (0.2 M) under an Ar atmosphere. An aqueous solution of NaOH (1 M, 1.83 mL, 2.0 eq.) was added to the mixture. The reaction mixture was stirred for 18 h at room temperature under an atmosphere of Ar. Once complete, the mixture was diluted with EtOAc and acidified with 1 M HCl. The aqueous layer was extracted with EtOAc. The organic layers were combined, washed with brine, dried over Na_2SO_4, and evaporated under reduced pressure. The residue was purified via flash column chromatography on silica gel (Hexane:Ethyl Acetate = 7:3) to afford the corresponding carboxylic acid **14** (150 mg, 86%) as a white solid.

R_f = 0.16 (SiO_2, Hex:EtOAc 8:2, UV, $KMnO_4$)

^{1}H NMR (400 MHz, $CDCl_3$) δ 9.88 (br s, 1H), 7.22 – 7.06 (m, 4H), 5.48 (d, J = 5.3 Hz, 1H), 5.38 (d, J = 5.0 Hz, 1H), 3.33 (ddd, J = 9.7, 5.3, 3.9 Hz, 1H), 2.25 (ddd, J = 11.7, 10.0, 5.1 Hz, 1H), 1.67 (dd, J = 11.9, 3.9 Hz, 1H).

^{13}C NMR (101 MHz, $CDCl_3$) δ 176.90, 145.60, 141.46, 127.63, 126.74, 120.96, 118.88, 80.05, 79.94, 45.00, 30.43.

HRMS: (ESI-TOF, m/z) $[M+Na]^+$ calcd. for $C_{11}H_{10}O_3$ m/z 213.0522 and found m/z 213.0519.

IR: (ATR, neat, cm^{-1}) = 3009, 1688, 1418, 1287, 1230, 1190, 1161, 1099.

m.p. = 102 – 104 °C.

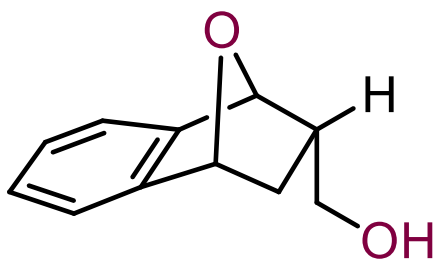

S3

Oxa-Bicyclic alcohol S3. To a solution of carboxylic acid **14** (150 mg, 0.79 mmol, 1.0 eq.) in 8 mL of THF (0.1 M), Et_3N (0.11 mL, 0.79 mmol, 1.0 eq.) was added. The mixture was cooled at -10°C and isobutyl chloroformate (0.10 mL, 0.79 mmol, 1.0 eq.) was added dropwise. The reaction was kept stirring at -10 °C for 1 h and then the salts were removed by filtration. The resulting clear solution was cooled at -10 °C, and $NaBH_4$ (59.7 mg, 1.58 mmol, 2.0 eq.) was added in one portion. MeOH (1.0 mL, 0.8 M) was added dropwise over 30 minutes, and the mixture was stirred at -10°C. Once complete, the mixture was diluted with EtOAc and acidified with 1 M HCl. The aqueous layer was extracted with EtOAc. The organic layers were combined, washed with brine, dried over Na_2SO_4, and evaporated under reduced pressure. The residue was purified via flash column chromatography on silica gel (Hexane:Ethyl Acetate = 7:3) to afford the corresponding alcohol **S3** (138 mg, 88%) as a colorless oil.

R_f = 0.26 (SiO_2, Hex:EtOAc 7:3, UV, $KMnO_4$)

^{1}H NMR (400 MHz, $CDCl_3$) δ 7.28 (dd, J = 6.3, 2.0 Hz, 1H), 7.21 – 7.12 (m, 3H), 5.37 (d, J = 3.9 Hz, 1H), 5.31 (d, J = 5.1 Hz, 1H), 3.34 – 3.23 (m, 1H), 2.70 – 2.61 (m, 2H), 2.28 – 2.17 (m, 1H), 1.93 (s, 1H), 0.79 (dd, J = 11.7, 3.7 Hz, 1H).

^{13}C NMR (101 MHz, $CDCl_3$) δ 146.01, 142.10, 127.06, 126.46, 120.95, 118.67, 81.03, 79.24, 64.29, 41.28, 30.75.

HRMS: (ESI-TOF, m/z) $[M+H]^+$ calcd. for $C_{11}H_{12}O_2$ m/z 177.0837 and found m/z 177.0830.

IR: (ATR, neat, cm^{-1}) = 3498, 3404, 1668, 1457, 1390, 1364, 1291, 1248, 1164, 1116, 1006, 909, 887.

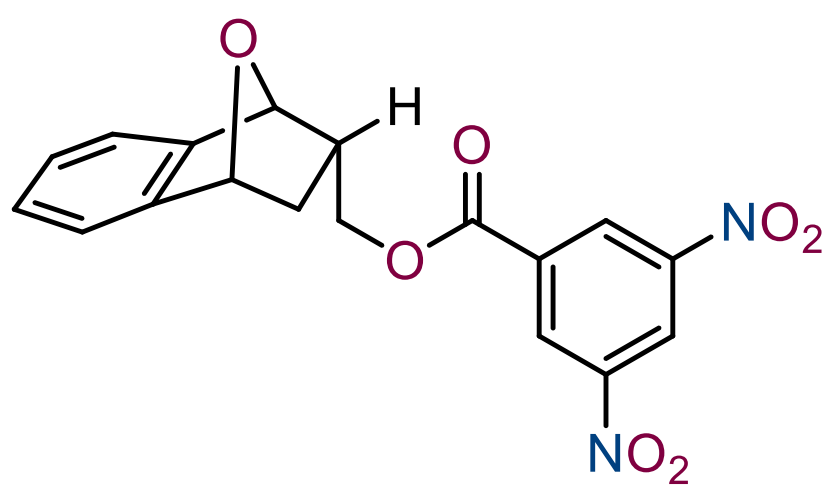

S4

Oxa-Bicyclic benzoate S4. To a stirred solution of alcohol **S3** (100 mg, 0.57 mmol, 1.0 equiv.) dissolved in dry DCM (5.7 mL, 0.1 M), were added sequentially 3,5-dinitrobenzoic acid (132 mg, 0.62 mmol, 1.1 equiv.), 4-DMAP (6.9 mg, 0.06 mmol, 0.1 equiv) and DCC (129 mg, 0.62 mmol, 1.1 equiv.). After 2 h of stirring the reaction was diluted with hexanes and washed with water, dried over Na_2SO_4, filtered and concentrated under reduced pressure. The residue was purified via flash column chromatography on silica gel (Hexane:Ethyl Acetate = 9:1) to afford the corresponding ester **S4** (168 mg, 80%) as a white crystalline solid.

R_f = 0.31(SiO_2, Hex:EtOAc 9:1, UV, $KMnO_4$)

^{1}H NMR (400 MHz, $CDCl_3$) δ 9.23 (t, J = 2.1 Hz, 1H), 9.09 (d, J = 2.1 Hz, 2H), 7.31 – 7.29 (m, 1H), 7.26 – 7.24 (m, 1H), 7.22 – 7.14 (m, 2H), 5.44 (dd, J = 4.9, 2.8 Hz, 2H), 4.16 (dd, J = 11.2, 6.0 Hz, 1H), 3.65 (dd, J = 11.2, 9.3 Hz, 1H), 3.02 – 2.96 (m, 1H), 2.44 (ddd, J = 11.6, 10.1, 5.1 Hz, 1H), 1.10 (dd, J = 11.7, 4.4 Hz, 1H).

^{13}C NMR (101 MHz, $CDCl_3$) δ 162.25, 148.85, 146.07, 141.52, 133.73, 129.46, 127.64, 126.90, 122.64, 120.83, 119.12, 80.64, 79.38, 68.01, 37.97, 31.26.

HRMS: (ESI-TOF, m/z) $[M+H]^+$ calcd. for $C_{18}H_{14}N_2O_7$ m/z 371.0801 and found m/z 371.0876.

IR: (ATR, neat, cm^{-1}) = 1731, 1664, 1600, 1479, 1449, 1408, 1369, 1313, 1237, 1166, 1026, 922.

m.p. = 142 – 143 °C.

Synthesis of azabicyclo[2.2.1]heptane derivatives

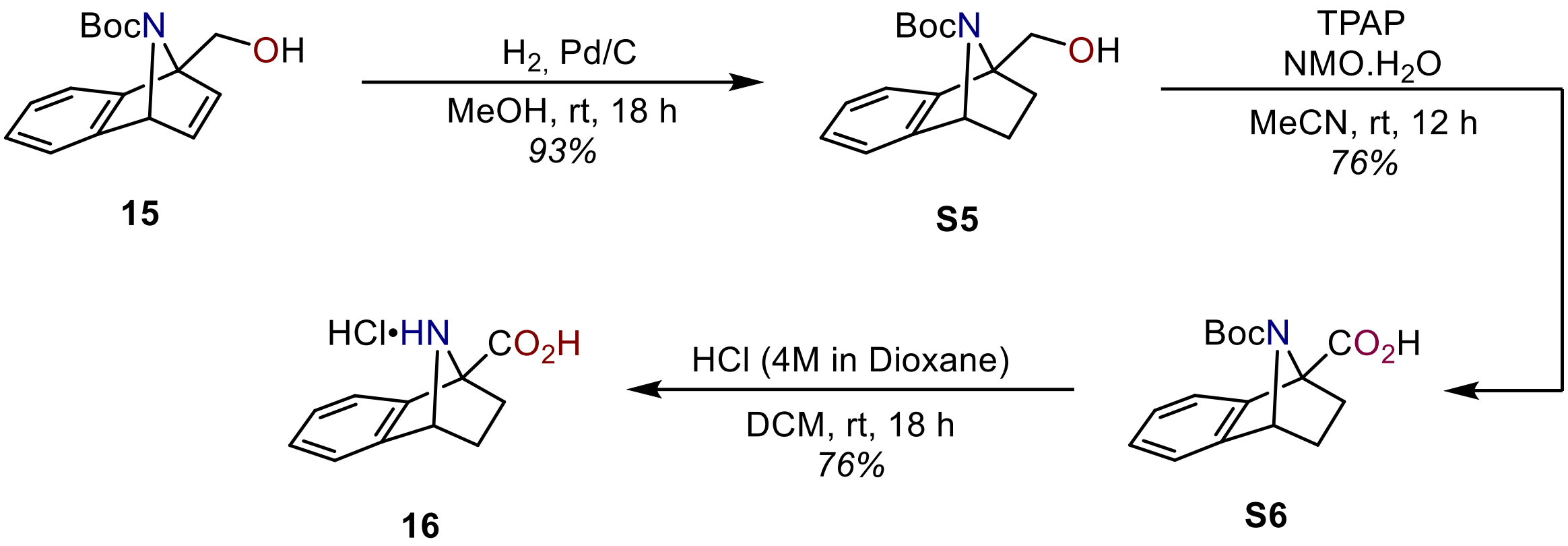

Scheme S3. Synthesis of α-amino acid derivative **16**.

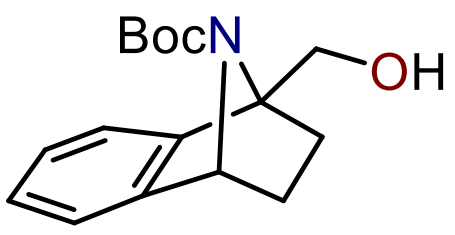

S5

Aza-Bicyclic alcohol S5. Alcohol **15** was prepared starting from pyrrole 2-carboxaldehyde according to a known literature procedure [4]. Unsaturated alcohol **15** (1.00 g, 3.66 mmol, 1.0 eq.) was solubilized in 36 mL of MeOH (0.1 M). The mixture was purged with Ar, then Pd/C (10% Pd on C, 389 mg, 0.10 eq.) was added to the mixture. The flask was filled with H_2 gas using a double balloon, and the reaction mixture was stirred for 18 h at room temperature under an atmosphere of H_2. Once complete, the mixture was filtered over a pad of celite, using MeOH as the eluent. The solvent was evaporated under reduced pressure, and the resulting residue was purified via flash column chromatography on silica gel (Hexane:Ethyl Acetate = 9:1 to 8:2) to afford pure saturated alcohol **S5** (940 mg, 93%) as a colorless oil.

$\mathbf{R_f}$ = 0.45 (SiO_2, Hex:EtOAc 8:2, UV-Vis, $KMnO_4$)

^{1}H NMR (400 MHz, $CDCl_3$) δ 7.32 – 7.19 (m, 4H), 5.21 – 5.20 (m, 1H), 4.57 – 4.44 (m, 2H), 2.26 – 2.15 (m, 2H), 1.47 (s, 9H), 1.40 – 1.35 (m, 1H), 1.32 – 1.25 (m, 1H).

^{13}C NMR (101 MHz, $CDCl_3$) δ 154.68, 145.04, 144.75, 126.67, 126.56, 119.33, 118.79, 80.91, 72.96, 61.94, 60.02, 29.25, 28.36, 27.64.

HRMS: (ESI-TOF, m/z) $[M+Na]^+$ calcd. for $C_{16}H_{21}NO_3$ m/z 298.1414 and found m/z 298.1403.

IR: (ATR, neat, cm^{-1}) = 3414, 1668, 1541, 1474, 1451, 1388, 1366, 1165, 1068, 1008, 943, 846.

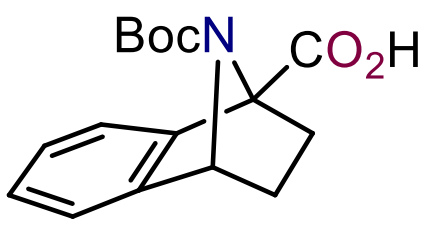

S6

Aza-Bicyclic acid S6. To a solution of alcohol **S5** (0.90 g, 3.27 mmol, 1.0 eq.) and N-methyl morpholine N-oxide (NMO) monohydrate (1.91 g, 16.3 mmol, 5.0 equiv.) in 32 mL of acetonitrile (0.1 M), TPAP (115 mg, 0.33 mmol, 0.1 eq.) is added and the mixture is stirred at room temperature for 12 hours. On completion the reaction is quenched with an excess of 2-propanol. The solvent is evaporated, and the residue is filtered over a pad of silica using ethyl acetate containing 1% of acetic acid. The filtrate is concentrated in vacuo and the crude product is subjected to column chromatography on silica gel (Hexane:Ethyl Acetate = 8:2) to afford carboxylic acid **S6** (0.72 g, 76%) as a colorless oil.

R_f = 0.33 (SiO_2, Hex:EtOAc 7:3, UV, $KMnO_4$)

^{1}H NMR (400 MHz, $CDCl_3$) δ 10.33 (br s, 1H), 7.64 – 7.62 (m, 1H), 7.35 – 7.26 (m, 3H), 5.31 (d, J = 4.5 Hz, 1H), 2.60 (td, J = 11.1, 3.8 Hz, 1H), 2.35 (ddt, J = 15.3, 10.4, 4.3 Hz, 1H), 1.80 (ddd, J = 12.4, 8.9, 4.0 Hz, 1H), 1.48 – 1.43 (m, 10H).

^{13}C NMR (101 MHz, $CDCl_3$) 156.60, 143.66, 143.36, 127.39, 127.04, 120.22, 119.65, 82.59, 63.34, 32.18, 28.54, 28.02, 27.81, 27.23.

HRMS: (ESI-TOF, m/z) $[M+Na]^+$ calcd. for $C_{16}H_{19}NO_4$ m/z 312.1206 and found m/z 312.1198.

IR: (ATR, neat, cm^{-1}) = 3049, 1705, 1366, 1325, 1250, 1157, 1120, 900, 840.

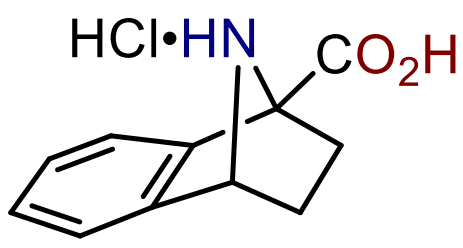

16

Aza-Bicyclic hydrochloride salt 16. Boc-protected derivative **S6** (500 mg, 1.73 mmol, 1.0 eq.) was solubilized in 5 mL of DCM (0.15 M). The mixture was purged with Ar, then HCl (4 M in Dioxane, 8.64 mL, 20 eq.) was added to the mixture. The reaction mixture was stirred for 18 h at room temperature under an atmosphere of Ar. Once complete, the mixture was filtered under vacuum, using DCM as the eluent. The corresponding hydrochloride salt **16** was obtained (250 mg, 76%) as a white solid.

R_f = 0.23 (SiO_2, EtOAc:MeOH 8:2, UV, $KMnO_4$)

^{1}H NMR (400 MHz, D_2O) δ 7.65 – 7.62 (m, 1H), 7.56 – 7.53 (m, 1H), 7.52 – 7.47 (m, 2H), 5.33 – 5.32 (m, 1H), 2.58 – 2.49 (m, 2H), 2.00 – 1.95 (m, 1H), 1.78 – 1.73 (m, 1H).

^{13}C NMR (101 MHz, D_2O) δ 170.55, 138.69, 137.75, 129.35, 128.99, 121.44, 120.20, 74.92, 60.81, 28.09, 25.11.

HRMS: (ESI-TOF, m/z) $[M+H]^+$ calcd. for $C_{11}H_{11}NO_2$ m/z 190.0863 and found m/z 190.0860.

IR: (ATR, neat, cm^{-1}) = 3395, 2853, 1733, 1593, 1459, 1407, 1360, 1321, 1250.

m.p. = 194-196 °C.

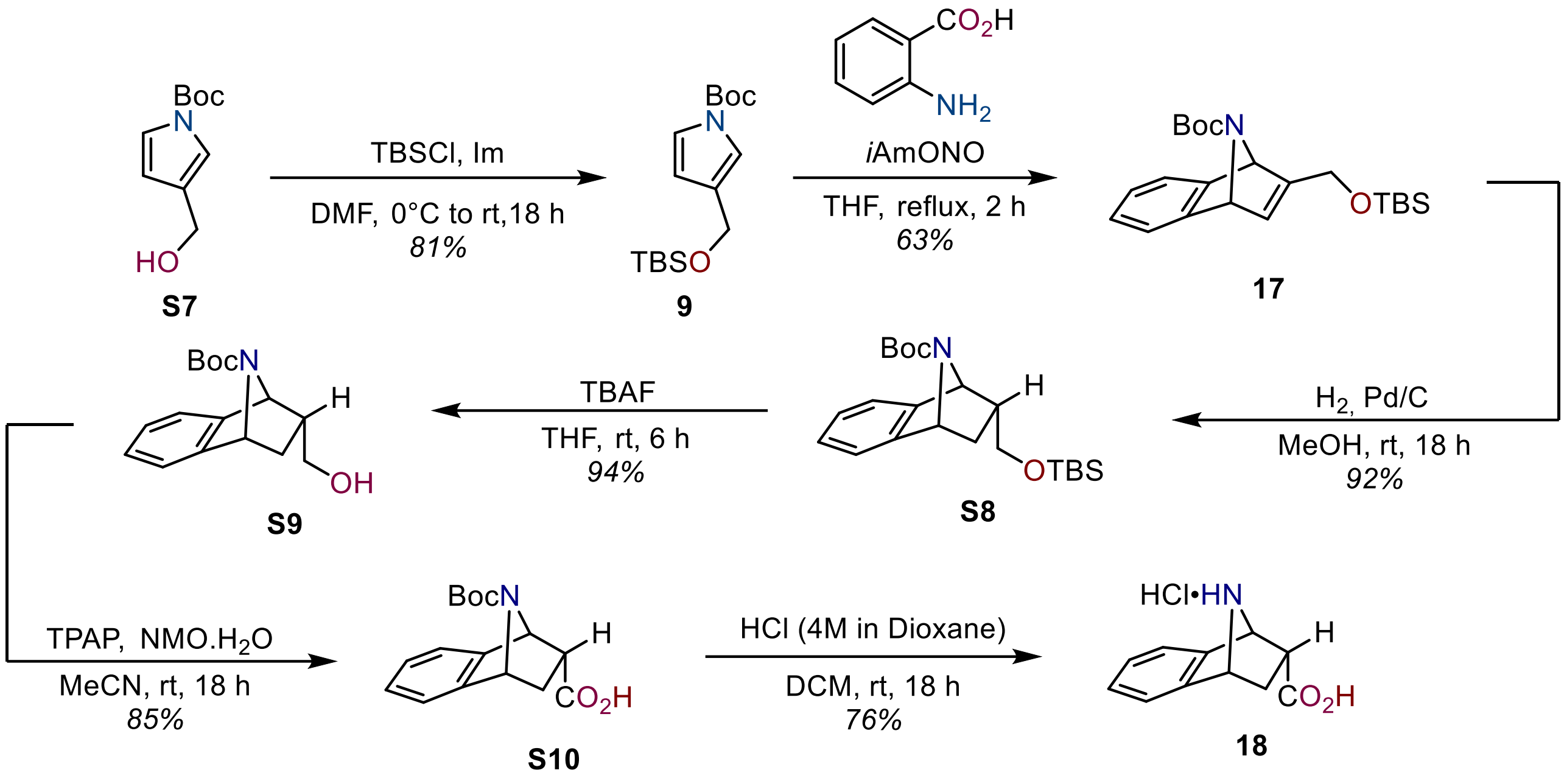

Scheme S4. Synthesis of β-amino acid derivative **18**.

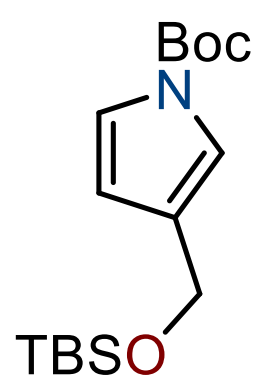

9

Pyrrole 9. Alcohol **S7** was prepared according to a known literature procedure [5]. To a solution of alcohol **S7** (5.00 g, 25.4 mmol, 1.0 eq.) and imidazole (3.40 g, 50.7 mmol, 2.0 eq.) in 60 mL of anhydrous DMF (0.4 M) was added *tert*-butylchlorodimethylsilane (5.73 g, 38.0 mmol, 1.5 eq.) at 0°C. The reaction mixture was allowed to warm to room temperature and stirred for 18 h. The reaction mixture was quenched with sat. aq. $NaHCO_3$ solution and extracted with DCM. The combined organic layers were washed with brine, dried over Na_2SO_4 and concentrated under reduced pressure. The resulting residue was purified via flash column chromatography on silica gel (Hexane:Ethyl Acetate = 99:1 to 95:5) to afford protected alcohol **9** (6.40 g, 81%) as a yellow liquid.

$\mathbf{R_f}$ = 0.69 (SiO_2, Hex:EtOAc 95:5, UV-Vis, $KMnO_4$).

^{1}H NMR (400 MHz, $CDCl_3$) δ 7.18 – 7.13 (m, 2H), 6.18 (dd, J = 3.3, 1.7 Hz, 1H), 4.57 (d, J = 1.0 Hz, 2H), 1.58 (s, 9H), 0.92 (s, 9H), 0.09 (s, 6H).

^{13}C NMR (101 MHz, $CDCl_3$) δ 149.07, 127.95, 120.49, 117.14, 111.51, 83.53, 59.15, 28.14, 26.11, 18.58, -5.05.

HRMS: (ESI-TOF, m/z) $[M+H]^+$ calcd. for $C_{16}H_{29}NO_3Si$ m/z 312.1917 and found m/z 312.1921.

IR: (ATR, neat, cm^{-1}) = 1742, 1474, 1409, 1347, 1246, 1157, 1064, 971, 835, 770.

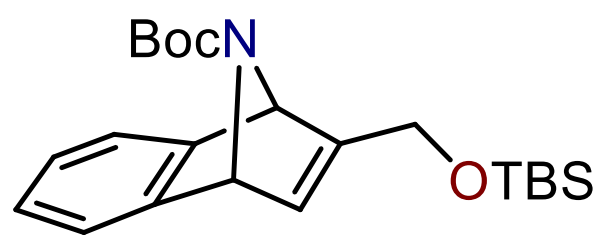

17

Aza-Bicyclic silyl ether 17. Solutions of anthranilic acid (6.60 g, 48.2 mmol, 3.0 eq.) in tetrahydrofuran (96 mL, 0.5 M) and isopentyl nitrite (7.01 mL, 52.2 mmol, 3.25 eq.) in tetrahydrofuran (104 mL, 0.5 M) were added dropwise and simultaneously to a refluxing solution of tert-butyl 3-(((tert-butyldimethylsilyl)oxy)methyl)-1H-pyrrole-1-carboxylate **9** (5.00 g, 16.1 mmol, 1.0 eq.) in tetrahydrofuran (16 mL, 1 M) over a period of about 2 h. The solution was refluxed for further 2 h, it was cooled to room temperature, aqueous sodium bicarbonate was added. The organic solvent was evaporated, and the aqueous phase was extracted with Et_2O. The organic layers were combined, washed with brine, dried over Na_2SO_4, and evaporated under reduced pressure. The residue was purified via flash column chromatography on silica gel (Hexane:Ethyl Acetate = 95:5 to 9:1) to afford the corresponding cycloadduct **17** (3.90 g, 63%) as a yellow oil.

R_f = 0.52 (SiO_2, Hex:EtOAc 9:1, UV-Vis, $KMnO_4$)

^{1}H NMR (400 MHz, DMSO-d6) δ 7.32 – 7.30 (m, 1H), 7.28 – 7.26 (m, 1H), 6.96 – 6.94 (m, 2H), 6.60 (q, J = 2.2 Hz, 1H), 5.42 (t, J = 1.9 Hz, 1H), 5.27 (d, J = 1.4 Hz, 1H), 4.38 – 4.26 (m, 2H), 1.32 (s, 9H), 0.87 (s, 9H), 0.01 (s, 6H).

^{13}C NMR (101 MHz, DMSO-d6) δ 156.97, 154.07, 148.61, 147.88, 134.47, 124.53, 124.14, 120.28, 119.90, 79.34, 66.42, 66.05, 59.93, 27.56, 25.43, 17.62, -5.80, -5.83.

HRMS: (ESI-TOF, m/z) $[M+Na]^+$ calcd. for $C_{22}H_{33}NO_3Si$ m/z 410.2122 and found m/z 410.2114.

IR: (ATR, neat, cm^{-1}) = 1707, 1455, 1366, 1329, 1250, 1164, 1075, 835, 761.

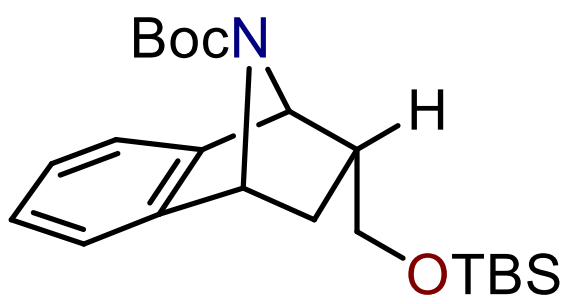

S8

Aza-Bicyclic silyl ether S8. TBS-protected unsaturated alcohol **17** (2.00 g, 5.16 mmol, 1.0 eq.) was solubilized in 51 mL of MeOH (0.1 M). The mixture was purged with Ar, then Pd/C (10% Pd on C, 549 mg, 0.10 eq.) was added to the mixture. The flask was filled with H_2 gas using a double balloon, and the reaction mixture was stirred for 18 h at room temperature under an atmosphere of H_2. Once complete, the mixture was filtered over a pad of celite, using MeOH as the eluent. The solvent was evaporated under reduced pressure, and the resulting residue was purified via flash column chromatography on silica gel (Hexane:Ethyl Acetate = 98:2 to 95:5) to afford pure saturated cycloadduct **S8** (1.85 g, 92%) as a colorless oil and as a single isomer.

R_f = 0.35 (SiO_2, Hex:EtOAc 95:5, UV-Vis, $KMnO_4$)

^{1}H NMR (400 MHz, $CDCl_3$) δ 7.31 – 7.28 (m, 1H), 7.22 (dd, J = 5.8, 2.7 Hz, 1H), 7.18 – 7.13 (m, 2H), 5.14 – 5.13 (m, 1H), 5.05 (d, J = 3.9 Hz, 1H), 3.27 (dd, J = 10.1, 5.7 Hz, 1H), 2.72 – 2.67 (m, 1H), 2.50 (t, J = 10.2 Hz, 1H), 2.25 (ddd, J = 11.7, 9.9, 4.8 Hz, 1H), 1.41 (s, 9H), 0.92 (s, 9H), 0.66 (dd, J = 11.8, 4.5 Hz, 1H), 0.00 (s, 3H), -0.02 (s, 3H).

^{13}C NMR (101 MHz, $CDCl_3$) δ 155.32, 145.28, 141.40, 126.68, 125.98, 122.15, 119.08, 79.94, 64.31, 63.48, 61.30, 41.30, 30.27, 28.23, 25.87, 18.15, -5.40.

HRMS: (ESI-TOF, m/z) $[M+H]^+$ calcd. for $C_{22}H_{35}NO_3Si$ m/z 412.2278 and found m/z 412.2272.

IR: (ATR, neat, cm^{-1}) = 2953, 1701, 1629, 1459, 1388, 1364, 1248, 1164, 1079, 1006, 970, 907, 837.

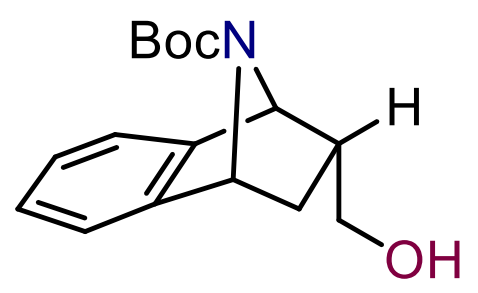

S9

Aza-Bicyclic alcohol S9. To a solution of protected alcohol **S8** (1.50 g, 3.85 mmol, 1.0 eq.) in 38 mL of anhydrous THF (0.1 M) under Ar, was added tetra n-butylammonium fluoride (TBAF, 1 M in THF, 5.78 mL, 5.78 mmol, 1.5 eq.) dropwise at room temperature and the mixture was stirred for 6 h. Once complete, the mixture was extracted with EtOAc and washed with water. The organic layers were combined, washed with brine, dried over Na_2SO_4, and evaporated under reduced pressure. The residue was purified via flash column chromatography on silica gel (Hexane:Ethyl Acetate = 7:3) to afford the free alcohol **S9** (0.99 g, 94%) as a colourless oil.

R_f = 0.33 (SiO_2, Hex:EtOAc 7:3, UV, $KMnO_4$).

^{1}H NMR (400 MHz, $CDCl_3$) δ 7.32 – 7.29 (m, 1H), 7.26 – 7.20 (m, 1H), 7.18 – 7.11 (m, 2H), 5.16 – 5.15 (m, 1H), 5.05 (d, J = 4.7 Hz, 1H), 3.22 (q, J = 4.3 Hz, 1H), 2.74 – 2.64 (m, 2H), 2.28 (ddd, J = 11.6, 9.5, 4.8 Hz, 1H), 2.00 (s, 1H), 1.38 (s, 9H), 0.73 (dd, J = 11.5, 3.7 Hz, 1H).

^{13}C NMR (101 MHz, $CDCl_3$) δ 155.38, 145.33, 141.32, 126.96, 126.33, 121.81, 119.43, 80.27, 64.21, 63.24, 61.30, 41.23, 30.76, 28.32.

HRMS: (ESI-TOF, m/z) $[M+Na]^+$ calcd. for $C_{16}H_{21}NO_3$ m/z 298.1414 and found m/z 298.1405.

IR: (ATR, neat, cm^{-1}) = 3432, 1700, 1675, 1364, 1254, 1162, 1090, 1051, 1023, 905.

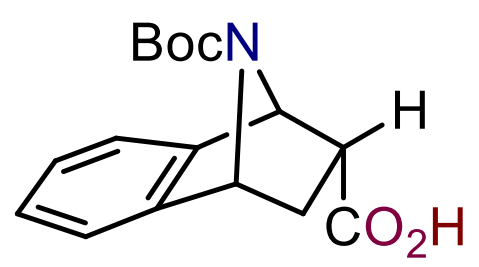

S10

Aza-Bicyclic acid S10. Alcohol **S9** (450 mg, 1.63 mmol, 1.0 eq.) and N-methyl morpholine N-oxide monohydrate (2.23 g, 16.3 mmol, 10.0 equiv.) are dissolved in 15 mL of acetonitrile (0.1 M). TPAP (57 mg, 0.16 mmol, 0.1 eq.) is added and the mixture is stirred at room temperature. On completion the reaction is quenched with an excess of 2-propanol. The solvent is evaporated, and the residue is filtered over a pad of silica using ethyl acetate containing 1% of acetic acid. The filtrate is concentrated in vacuo and the resulting residue was purified via flash column chromatography on silica gel (Hexane:Ethyl Acetate = 8:2 to 7:3) to afford the corresponding carboxylic acid **S10** (400 mg, 85%) as a pale yellow oil.

R_f = 0.43 (SiO_2, Hex:EtOAc 7:3, UV, $KMnO_4$).

^{1}H NMR (400 MHz, $CDCl_3$) δ 7.32 – 7.15 (m, 4H), 5.35 (d, J = 4.6 Hz, 1H), 5.19 (d, J = 4.4 Hz, 1H), 3.46 (dt, J = 9.6, 4.4 Hz, 1H), 2.39 (td, J = 11.2, 4.6 Hz, 1H), 1.72 (dd, J = 12.1, 4.0 Hz, 1H), 1.45 (d, J = 1.2 Hz, 9H).

^{13}C NMR (101 MHz, $CDCl_3$) δ 176.76, 154.94, 145.03, 140.72, 127.50, 126.61, 121.75, 119.61, 80.89, 62.89, 61.72, 44.51, 30.61, 28.30.

HRMS: (ESI-TOF, m/z) $[M+H]^+$ calcd. for $C_{16}H_{19}NO_4$ m/z 312.1206 and found m/z 312.1203.

IR: (ATR, neat, cm^{-1}) = 3070, 1675, 1520, 1480, 1420, 1385, 1162, 920, 830.

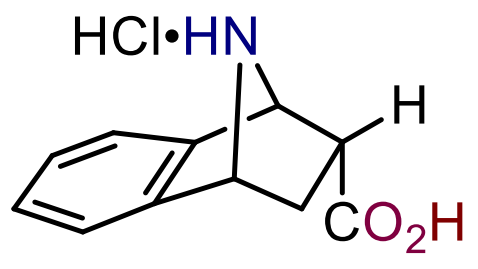

18

Aza-Bicyclic hydrochloride salt 18. Boc-protected derivative **S10** (250 mg, 0.86 mmol, 1.0 eq.) was solubilized in 3 mL of DCM (0.15 M). The mixture was purged with Ar, then HCl (4 M in Dioxane, 4.32 mL, 20 eq.) was added to the mixture. The reaction mixture was stirred for 18 h at room temperature under an atmosphere of Ar. Once complete, the mixture was filtered under vacuum, using DCM as the eluent. The corresponding hydrochloride salt **18** was obtained (125 mg, 76%) as a white solid.

$\mathbf{R_f}$ = 0.26 (SiO_2, EtOAc:MeOH 8:2, UV, $KMnO_4$)

^{1}H NMR (400 MHz, D_2O) δ 7.53 – 7.39 (m, 4H), 5.48 (dt, J = 4.8, 0.8 Hz, 1H), 5.35 (d, J = 4.6 Hz, 1H), 3.83 – 3.78 (m, 1H), 2.61 (ddd, J = 13.3, 10.1, 4.7 Hz, 1H), 1.91 (dd, J = 13.4, 4.1 Hz, 1H).

^{13}C NMR (101 MHz, D_2O) δ 173.26, 139.08, 135.22, 129.69, 128.82, 122.94, 121.12, 62.54, 62.27, 41.87, 27.51.

HRMS: (ESI-TOF, m/z) $[M+H]^+$ calcd. for $C_{11}H_{11}NO_2$ m/z 190.0863 and found m/z 190.0856.

IR: (ATR, neat, cm^{-1}) = 3090, 1700, 1614, 1347, 1213, 1161, 854, 766.

m.p. = 214 - 215 °C.

Synthesis of fulvene-based bicyclic derivatives

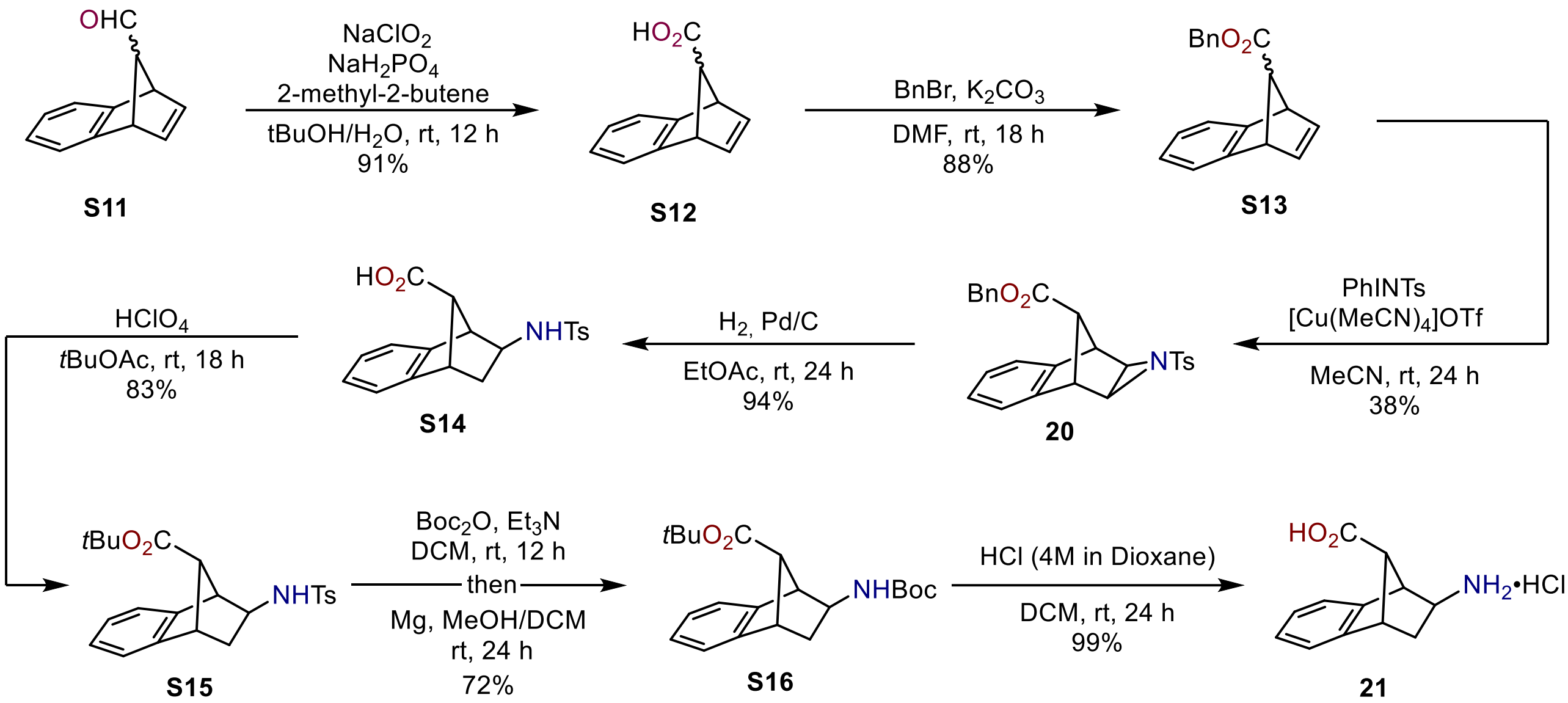

Scheme S5. Synthesis of γ-amino acid **21**.

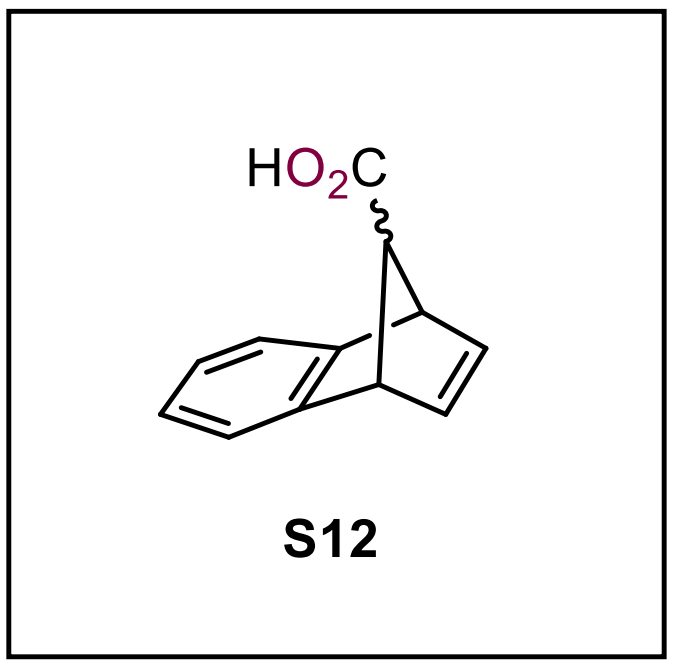

Bicyclic acid S12. To a solution of known aldehyde [6] **S11** (5.00 g, 29.4 mmol, 1.0 eq.) in 500 mL of *t*BuOH (0.05 M) and 500 mL of H_2O (0.05 M) at room temperature, 2-methyl-2-butene (94 mL, 881 mmol, 30 eq.) was added, together with $NaH_2PO_4.2H_2O$ (68.8 g, 441 mmol, 15 eq.) and $NaClO_2$ (26.6 g, 294 mmol, 10 eq.). The reaction mixture was stirred for 12 h at room temperature. The reaction was quenched with the addition of sat. $NaHCO_3$ solution. The aqueous phase was extracted with DCM, and the organic layers were combined. Then, 1 M NaOH was added, and the aqueous phase was washed with DCM. Finally, the aqueous layer was acidified with 1 M HCl, and it was extracted with DCM. The organic layers were combined, washed with brine, dried over Na_2SO_4, and evaporated under reduced pressure to afford carboxylic acid **S12** (4.98 g, 91%) as a white solid and as a 1.5:1 mixture of inseparable isomers.

For ^{1}H-NMR and ^{13}C-NMR, peaks of both isomers are reported. Fractional ^{1}H-NMR integration is due to a 1.5:1 ratio of isomers.

R$_f$ = 0.29 (SiO_2, Hex:EtOAc 8:2, UV, $KMnO_4$)

^{1}H NMR (400 MHz, $CDCl_3$) δ 10.14 (br s, 2.5H), 7.26 – 7.20 (m, 5H), 6.99 – 6.92 (m, 5H), 6.78 (t, *J* = 2.1 Hz, 3H), 6.73 (t, *J* = 1.9 Hz, 2H), 4.16 (q, *J* = 1.9 Hz, 2H), 4.09 (q, *J* = 2.0 Hz, 3H), 3.41 (t, *J* = 1.7 Hz, 1.5H), 3.31 (t, *J* = 1.7 Hz, 1H).

^{13}C NMR (101 MHz, $CDCl_3$) δ 176.77, 175.98, 149.72, 148.23, 142.86, 141.08, 125.24, 124.99, 122.87, 121.88, 80.46, 80.33, 51.72, 51.52.

HRMS: (ESI-TOF, m/z) $[M+Na]^+$ calcd. for $C_{12}H_{10}O_2$ m/z 209.0573 and found m/z 209.0568.

IR: (ATR, neat, cm^{-1}) = 3051, 1698, 1414, 1258, 1174, 949, 915, 837.

m.p. = 153 – 156 °C.

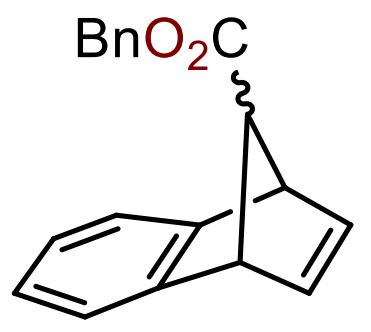
S13

Bicyclic benzyl ester S13. To a solution of carboxylic acid **S12** (2.00 g, 10.7 mmol, 1.0 eq.) in 30 mL of DMF (0.35 M) at room temperature, K_2CO_3 (2.97 g, 21.5 mmol, 2 eq.) was added, together with benzyl bromide (1.53 mL, 12.9 mmol, 1.2 eq.). The reaction mixture was stirred for 18 h at room temperature. The reaction was quenched with the addition of water, and the aqueous phase was extracted with Et_2O. The organic layers were combined, washed with brine, dried over Na_2SO_4, and evaporated under reduced pressure. The resulting residue was purified via flash column chromatography on silica gel (Hexane:Ethyl Acetate = 95:5) to afford benzyl ester **S13** (2.60 g, 88%) as a white solid and as a mixture of inseparable isomers.

For ^{1}H-NMR and ^{13}C-NMR, peaks of both isomers are reported. Fractional ^{1}H-NMR integration is due to a 2.5:1 ratio of isomers.

R_f = 0.32 (SiO_2, Hex:EtOAc 95:5, UV, $KMnO_4$)

^{1}H NMR (400 MHz, $CDCl_3$) δ 7.37 – 7.21 (m, 20H), 7.02 – 6.93 (m, 11.5H), 6.79 (t, J = 2.1 Hz, 5H), 6.74 (t, J = 1.9 Hz, 2H), 5.11 (s, 2H), 4.88 (s, 5H), 4.23 (q, J = 1.9 Hz, 2H), 4.15 (q, J = 2.0 Hz, 5H), 3.46 (t, J = 1.8 Hz, 2.5H), 3.37 (q, J = 2.6, 1.7 Hz, 1H).

^{13}C NMR (101 MHz, $CDCl_3$) δ 170.23, 169.87, 149.84, 148.42, 142.69, 140.99, 136.10, 135.96, 128.65, 128.61, 128.43, 128.39, 128.34, 128.33, 128.24, 128.02, 127.96, 127.73, 125.12, 124.90, 122.81, 121.80, 80.94, 80.78, 66.34, 65.98, 51.98, 51.83.

HRMS: (ESI-TOF, m/z) $[M+Na]^+$ calcd. for $C_{19}H_{16}O_2$ m/z 299.1043 and found m/z 299.1035.

IR: (ATR, neat, cm^{-1}) = 1724, 1694, 1450, 1213, 1180, 1012, 937, 890, 743, 699.

m.p. = 84 – 88 °C.

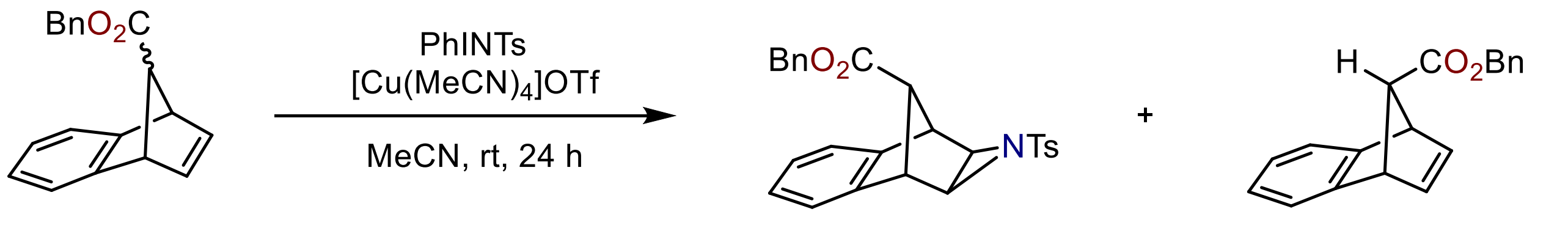

Scheme S6. Aziridination reaction on **S13**, with the recovery of major isomer.

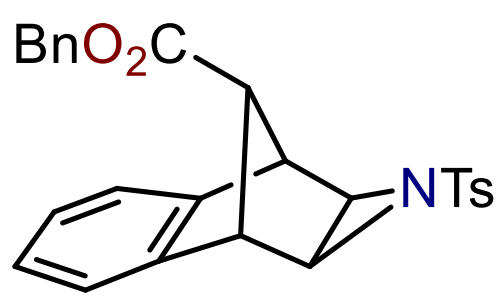

20

Bicyclic tosyl aziridine 20. To a solution of olefin **S13** (1.00 g, 4.99 mmol, 1.0 eq.) in 50 mL of MeCN (0.10 M) at room temperature, PhINTs (2.24 g, 5.99 mmol, 1.2 eq.) was added, together with Cu catalyst (188 mg, 0.49 mmol, 0.1 eq.). The reaction mixture was stirred for 24 h at room temperature. Once complete, it was filtered over a pad of silica gel using EtOAc as the eluent, and the solvent was evaporated. The resulting residue was purified via flash column chromatography on silica gel (Hexane:Ethyl Acetate = 8:2) to afford aziridinated bicycle **20** (0.70 g, 38%) as a light yellow solid and as a single isomer.

R_f = 0.24 (SiO_2, Hex:EtOAc 8:2, UV, Vanillin, $KMnO_4$)

^{1}H NMR (400 MHz, $CDCl_3$) δ 7.74 – 7.72 (m, 2H), 7.26 – 7.24 (m, 2H), 7.16 – 7.14 (m, 5H), 7.03 – 7.01 (m, 2H), 6.89 – 6.87 (m, 2H), 4.75 (s, 2H), 3.71 (s, 2H), 3.23 (d, J = 1.8 Hz, 1H), 3.13 (s, 2H), 2.36 (s, 3H).

^{13}C NMR (101 MHz, $CDCl_3$) δ 170.94, 144.76, 143.93, 135.63, 135.05, 131.52, 130.44, 129.86, 128.40, 128.04, 127.93, 127.72, 127.05, 123.60, 66.18, 55.45, 46.01, 45.97, 21.69.

HRMS: (ESI-TOF, m/z) $[M+Na]^+$ calcd. for $C_{26}H_{23}NO_4S$ m/z 468.1240 and found m/z 468.1223.

IR: (ATR, neat, cm^{-1}) = 1726, 1597, 1347, 1327, 1213, 1194, 1153, 1086, 1016, 990, 889, 725, 695.

m.p. = 120 – 123 °C.

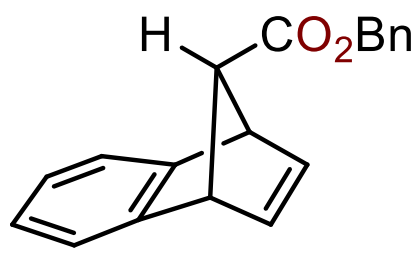

S13-major isomer

R_f = 0.32 (SiO_2, Hex:EtOAc 95:5, UV, $KMnO_4$)

^{1}H NMR (400 MHz, $CDCl_3$) δ 7.33 – 7.21 (m, 5H), 7.19 – 7.09 (m, 2H), 6.88 (dd, J = 5.3, 3.1 Hz, 2H), 6.66 (t, J = 1.9 Hz, 2H), 5.03 (s, 2H), 4.15 (q, J = 1.9 Hz, 2H), 3.29 (q, J = 1.9 Hz, 1H).

^{13}C NMR (101 MHz, $CDCl_3$) δ 170.31, 149.90, 141.04, 136.13, 128.66, 128.64, 128.29, 128.07, 124.94, 121.85, 80.83, 66.41, 52.02.

HRMS: (ESI-TOF, m/z) $[M+Na]^+$ calcd. for $C_{19}H_{16}O_2$ m/z 299.1043 and found m/z 299.1038.

IR: (ATR, neat, cm^{-1}) = 1724, 1694, 1450, 1213, 1180, 1012, 937, 890, 743, 699.

m.p. = 84 – 88 °C.

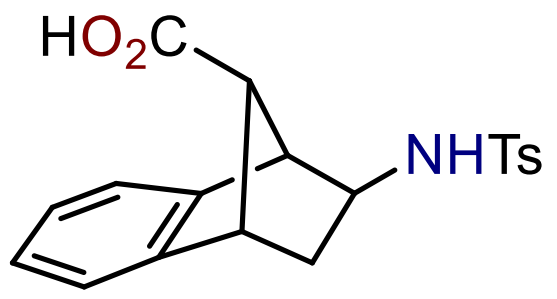

S14

Bicyclic acid S14. Aziridine **20** (450 mg, 1.01 mmol, 1.0 eq.) was solubilized in 10 mL of EtOAc (0.1 M). The mixture was purged with Ar, then Pd/C (10% Pd on C, 106 mg, 0.10 eq.) was added to the mixture. The flask was filled with H_2 gas using a double balloon, and the reaction mixture was stirred for 24 h at room temperature under an atmosphere of H_2. Once complete, the mixture was filtered over a pad of celite, using EtOAc as the eluent. The solvent was evaporated under reduced pressure, and the ring-opened bicycle **S14** was obtained (340 mg, 94%) as a white foam.

R_f = 0.44 (SiO_2, Hex:EtOAc 1:1, UV, Vanillin)

^{1}H NMR (400 MHz, MeOD) δ 7.74 (d, J = 8.3 Hz, 2H), 7.37 (d, J = 8.0 Hz, 2H), 7.12 – 7.03 (m, 4H), 3.48 – 3.47 (m, 1H), 3.35 (s, 1H), 3.16 (t, J = 1.6 Hz, 1H), 3.07 (dd, J = 8.1, 3.6 Hz, 1H), 2.42 (s, 3H), 1.69 (dt, J = 12.8, 3.8 Hz, 1H), 1.56 (dd, J = 12.8, 8.1 Hz, 1H).

^{13}C NMR (101 MHz, MeOD) δ 175.27, 147.08, 144.80, 143.61, 139.26, 130.80, 128.09, 128.03, 127.53, 123.23, 122.53, 61.98, 56.36, 53.70, 46.54, 37.95, 21.47.

HRMS: (ESI-TOF, m/z) $[M+Na]^+$ calcd. for $C_{19}H_{19}NO_4S$ m/z 380.0927 and found m/z 380.0897.

IR: (ATR, neat, cm^{-1}) = 1701, 1687, 1342, 1159, 1087, 993, 894, 814, 777, 723.

m.p. = 173 – 175 °C.

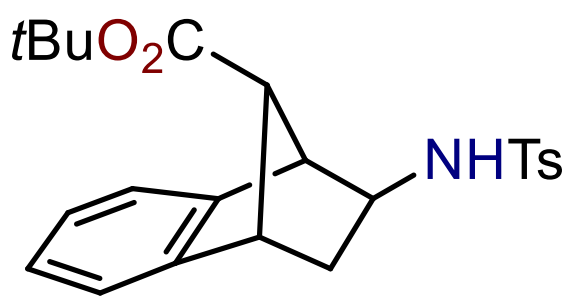

S15

Bicyclic tert-butyl ester S15. A solution of carboxylic acid **S14** (500 mg, 1.40 mmol, 1.0 eq.) in 14 mL *t*BuOAc (0.1 M) was prepared. Under stirring, 0.2 mL (1.82 mmol, 1.3 eq.) of $HClO_4$ (65% in H_2O) were added slowly at 0 °C. The mixture was then stirred at room temperature for 18 h. After adjusting the pH-value to 6 by addition of solid $NaHCO_3$, the excess of $NaHCO_3$ was filtered off and the solution was concentrated under reduced pressure. The residue was dissolved in 30% aqueous NH_3 and the solution extracted three times with M*t*BE. The combined organic phases were dried over Na_2SO_4, filtered and the solvent was removed under reduced pressure. The crude product was purified via flash column chromatography on silica gel (Hexane:Ethyl Acetate = 7:3) to afford *t*-butyl ester **S15** (579 mg, 83%) as a white solid.

R_f = 0.38 (SiO_2, Hex:EtOAc 7:3, UV, Vanillin)

^{1}H NMR (400 MHz, $CDCl_3$) δ 7.80 (d, J = 8.3 Hz, 2H), 7.33 (d, J = 8.3 Hz, 2H), 7.18 - 7.07 (m, 4H), 5.38 (d, J = 6.5 Hz, 1H), 3.50 - 3.46 (m, 2H), 3.28 - 3.24 (m, 1H), 3.06 - 3.04 (m, 1H), 2.44 (s, 3H), 1.79 - 1.73 (m, 1H), 1.69 - 1.64 (m, 1H), 1.14 (s, 9H).

^{13}C NMR (101 MHz, $CDCl_3$) δ 170.7, 145.7, 143.7, 142.2, 137.2, 129.9, 127.2, 126.9, 126.4, 122.4, 121.5, 80.5, 62.1, 54.9, 52.7, 45.7, 37.6, 27.6, 21.6.

HRMS: (ESI-TOF, m/z) $[M+Na]^+$ calcd. for $C_{23}H_{27}NO_4S$ m/z 436.1553 and found m/z 436.1538.

IR: (ATR, neat, cm^{-1}) = 3250, 2970, 1715, 1446, 1334, 1148, 1088, 813.

m.p. = 132 – 134 °C.

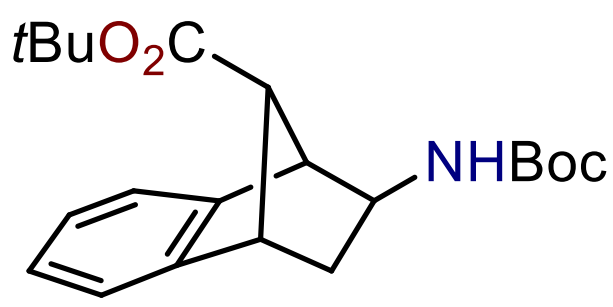

S16

Bicyclic Boc-amine S16. Tosyl protected amino acid **S15** (500 mg, 1.10 mmol, 1.0 eq.) was solubilized in 11 mL of DCM (0.1 M). Triethylamine was added to the mixture (0.51 mL, 3.30 mmol, 3.0 eq.), followed by Boc anhydride (0.98 mL, 3.85 mmol, 3.5 eq.). The mixture was stirred for 12 h, and the solvent was evaporated under reduced pressure. The resulting residue was solubilized in a mixture of 4 mL of MeOH and 8 mL of DCM (0.1 M). Magnesium turnings (0.31 g, 22.00 mmol, 20 eq.) were added, and the mixture was sonicated for 1 hour. It was stirred at room temperature for 24 hours, then saturated NH_4Cl was added. The aqueous phase was extracted with DCM, the organic phases were reunited, washed with brine, dried over Na_2SO_4, and evaporated under reduced pressure. The resulting residue was purified via flash column chromatography on silica gel (Hexane:Ethyl Acetate = 9:1 to 8:2) to afford the desired product **S16** (284 mg, 72%) as a white solid.

R_f = 0.36 (SiO_2, Hex:EtOAc 9:1, UV-Vis, Vanillin)

^{1}H NMR (400 MHz, $CDCl_3$) δ 7.26 (d, J = 3.4 Hz, 1H), 7.15 (dd, J = 5.4, 3.1 Hz, 1H), 7.07 (dd, J = 5.3, 3.1 Hz, 2H), 4.75 (d, J = 6.5 Hz, 1H), 3.61 – 3.49 (m, 3H), 2.96 (s, 1H), 1.90 (dd, J = 12.8, 8.1 Hz, 1H), 1.57 – 1.43 (m, 11H), 1.15 (s, 9H).

^{13}C NMR (101 MHz, $CDCl_3$) δ 171.32, 145.83, 142.86, 126.74, 126.44, 122.72, 121.46, 80.58, 62.41, 52.93, 52.59, 45.91, 37.62, 28.57, 27.80.

HRMS: (ESI-TOF, m/z) $[M+Na]^+$ calcd. for $C_{21}H_{29}NO_4$ m/z 382.1989 and found m/z 382.1979.

IR: (ATR, neat, cm^{-1}) = 3373, 2978, 1692, 1520, 1360, 1237, 1155, 730.

m.p. = 128 °C.

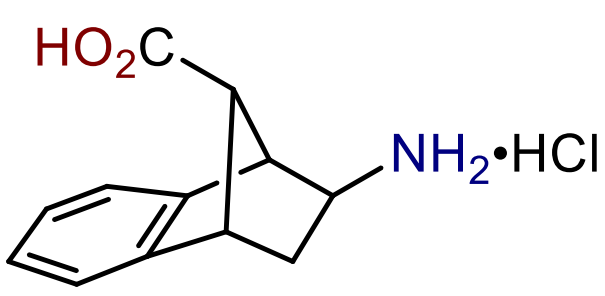

21

Bicyclic hydrochloride salt 21. Boc-protected derivative **S16** (200 mg, 0.56 mmol, 1.0 eq.) was solubilized in 5 mL of DCM (0.15 M). The mixture was purged with Ar, then HCl (4 M in Dioxane, 2.78 mL, 20 eq.) was added to the mixture. The reaction mixture was stirred for 24 h at room temperature under an atmosphere of Ar. Once complete, the mixture was filtered under vacuum, using DCM as the eluent. The corresponding hydrochloride salt **21** was obtained (133 mg, 99%) as a white solid.

R_f = 0.18 (SiO_2, EtOAc:MeOH 8:2, UV-Vis, Vanillin)

^{1}H NMR (400 MHz, D_2O) δ 7.40 – 7.38 (m, 1H), 7.34 – 7.32 (m, 1H), 7.27 – 7.20 (m, 2H), 3.83 (d, J = 1.7 Hz, 1H), 3.78 – 3.76 (m, 1H), 3.43 (t, J = 1.6 Hz, 1H), 3.30 (dd, J = 8.0, 3.9 Hz, 1H), 2.02 (dt, J = 13.4, 3.8 Hz, 1H), 1.94 (ddd, J = 13.4, 8.0, 0.9 Hz, 1H).

^{13}C NMR (101 MHz, D_2O) δ 175.24, 144.83, 141.00, 127.87, 127.12, 122.80, 122.14, 60.30, 52.10, 49.76, 45.52, 34.04.

HRMS: (ESI-TOF, m/z) $[M+H]^+$ calcd. for $C_{12}H_{13}NO_2$ m/z 204.1019 and found m/z 204.1012.

IR: (ATR, neat, cm^{-1}) = 2929, 1725, 1528, 1222, 1185, 920, 823, 697.

m.p. = Decomp. 250°C.

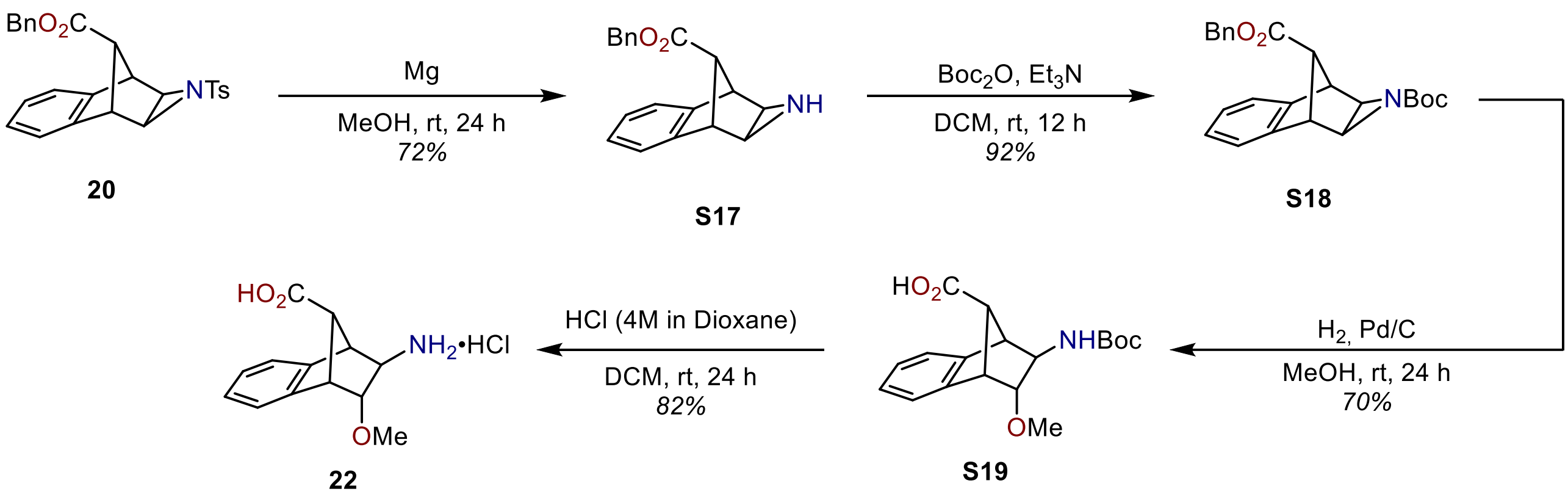

Scheme S7. Synthesis of γ-amino acid **22**.

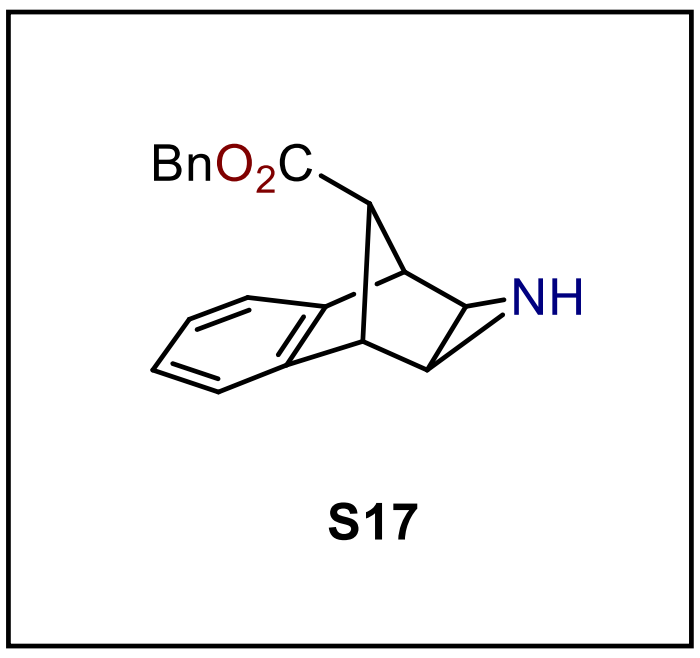

Bicyclic free aziridine S17. Tosyl-protected aziridine **20** (350 mg, 0.79 mmol, 1.0 eq.) was solubilized in 8 mL of MeOH (0.1 M). Magnesium turnings (382 mg, 15.7 mmol, 20 eq.) were added, and the mixture was sonicated for 1 hour. It was stirred at room temperature for 24 hours, then saturated NH_4Cl was added. The aqueous phase was extracted with DCM, the organic phases were reunited, washed with brine, dried over Na_2SO_4, and evaporated under reduced pressure. The resulting residue was purified via flash column chromatography on silica gel (Hexane:Ethyl Acetate = 6:4) to afford free aziridine **S17** (170 mg, 74%) as a colorless liquid.

R_f = 0.46 (SiO_2, Hex:EtOAc 1:1, UV, Ninhydrin)

^{1}H NMR (400 MHz, $CDCl_3$) δ 7.27 – 7.22 (m, 6H), 7.08 (dd, J = 5.3, 3.1 Hz, 2H), 7.02 – 6.99 (m, 2H), 4.89 (s, 2H), 3.68 (d, J = 1.8 Hz, 2H), 3.37 (s, 1H), 2.47 (s, 2H).

^{13}C NMR (101 MHz, $CDCl_3$) δ 172.82, 146.69, 136.16, 128.41, 127.89, 127.64, 127.62, 126.20, 122.85, 65.90, 53.16, 46.32, 36.77.

HRMS: (ESI-TOF, m/z) $[M+H]^+$ calcd. for $C_{19}H_{17}NO_2$ m/z 292.1332 and found m/z 292.1330.

IR: (ATR, neat, cm^{-1}) = 3293, 1724, 1456, 1316, 1247, 1210, 1195, 1174, 1155, 1012, 842.

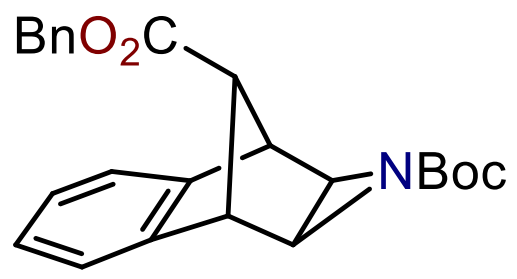

S18

Bicyclic Boc-aziridine S18. Free aziridine **S17** (150 mg, 0.52 mmol, 1.0 eq.) was solubilized in 5 mL of DCM (0.1 M). Triethylamine was added to the mixture (0.15 mL, 1.03 mmol, 2.0 eq.), followed by Boc anhydride (0.18 mL, 0.77 mmol, 1.5 eq.). The mixture was stirred for 12 h, and the solvent was evaporated under reduced pressure. The resulting residue was purified via flash column chromatography on silica gel (Hexane:Ethyl Acetate = 95:5) to afford Boc-protected aziridine **S18** (185 mg, 92%) as a colorless oil.

R_f = 0.51 (SiO_2, Hex:EtOAc 8:2, UV, Ninhydrin)

^{1}H NMR (400 MHz, $CDCl_3$) δ 7.25 (dd, J = 5.6, 2.8 Hz, 5H), 7.10 (dd, J = 5.3, 3.1 Hz, 2H), 7.03 – 6.98 (m, 2H), 4.88 (s, 2H), 3.95 – 3.94 (m, 2H), 3.30 (t, J = 1.8 Hz, 1H), 2.91 (s, 2H), 1.44 (s, 9H).

^{13}C NMR (101 MHz, $CDCl_3$) δ 171.83, 159.88, 144.79, 136.01, 128.46, 128.00, 127.70, 126.72, 123.40, 81.55, 66.08, 55.30, 46.28, 43.65, 28.06.

HRMS: (ESI-TOF, m/z) $[M+Na]^+$ calcd. for $C_{24}H_{25}NO_4$ m/z 414.1676 and found m/z 414.1656.

IR: (ATR, neat, cm^{-1}) = 1715, 1366, 1290, 1254, 1217, 1150, 833, 747.

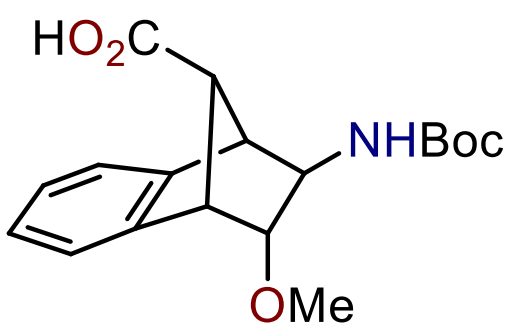

S19

Bicyclic acid S19. Boc-protected aziridine **S18** (150 mg, 0.38 mmol, 1.0 eq.) was solubilized in 4 mL of MeOH (0.1 M). The mixture was purged with Ar, then Pd/C (10% Pd on C, 40 mg, 0.10 eq.) was added to the mixture. The flask was filled with H_2 gas using a double balloon, and the reaction mixture was stirred for 24 h at room temperature under an atmosphere of H_2. Once complete, the mixture was filtered over a pad of celite, using MeOH as the eluent. The solvent was evaporated under reduced pressure, and the ring-opened methoxy-substituted carboxylic acid **S19** was obtained (90 mg, 70%) as a white solid.

R_f = 0.49 (SiO_2, Hex:EtOAc 1:1, UV, Vanillin)

^{1}H NMR (400 MHz, MeOD) δ 7.26 – 7.24 (m, 1H), 7.18 – 7.08 (m, 3H), 3.89 (s, 1H), 3.77 (t, J = 2.3 Hz, 1H), 3.62 (dq, J = 3.2, 1.6 Hz, 1H), 3.35 (s, 4H), 3.19 – 3.17 (m, 1H), 1.47 (s, 10H).

^{13}C NMR (101 MHz, MeOD) δ 175.44, 157.14, 143.84, 142.65, 128.08, 127.82, 124.42, 123.06, 87.15, 80.83, 68.58, 58.02, 52.27, 51.97, 51.31, 28.67.

HRMS: (ESI-TOF, m/z) $[M+H]^+$ calcd. for $C_{18}H_{23}NO_5$ m/z 334.1576 and found m/z 334.1581.

IR: (ATR, neat, cm^{-1}) = 3427, 3017, 1705, 1500, 1459, 1401, 1362, 1254, 1159, 1090, 930.

m.p. = 142 – 143 °C.

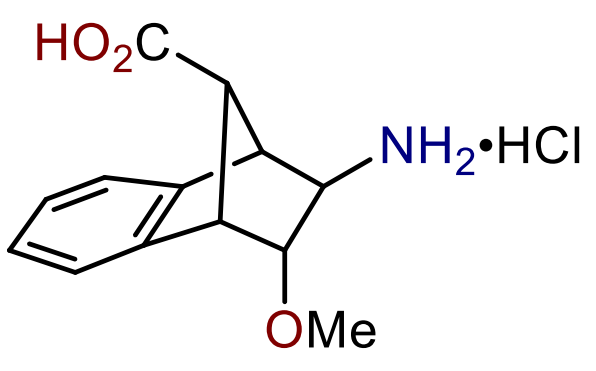

22

Bicyclic hydrochloride salt 22. Boc-protected derivative **S19** (75 mg, 0.22 mmol, 1.0 eq.) was solubilized in 2 mL of 1,4-dioxane (0.1 M). The mixture was purged with Ar, then HCl (4 M in Dioxane, 1.1 mL, 20 eq.) was added to the mixture. The reaction mixture was stirred for 24 h at room temperature under an atmosphere of Ar. Once complete, the solvent was evaporated, and the residue was triturated with Et_2O. The corresponding hydrochloride salt **22** was obtained (50 mg, 82%) as a white solid.

R_f = 0.22 (SiO_2, EtOAc:MeOH 8:2, UV, Vanillin)

^{1}H NMR (400 MHz, D_2O) δ 7.40 – 7.38 (m, 1H), 7.32 – 7.23 (m, 3H), 3.97 (dt, J = 4.3, 2.3 Hz, 2H), 3.89 (d, J = 1.7 Hz, 1H), 3.70 (q, J = 1.7 Hz, 1H), 3.51 (dd, J = 4.3, 2.4 Hz, 1H), 3.46 (s, 3H).

^{13}C NMR (101 MHz, D_2O) δ 174.59, 141.00, 139.09, 128.08, 127.74, 123.30, 123.00, 84.07, 66.55, 63.98, 57.13, 50.45, 49.36, 48.05.

HRMS: (ESI-TOF, m/z) $[M+H]^+$ calcd. for $C_{13}H_{15}NO_3$ m/z 234.1125 and found m/z 234.1118.

IR: (ATR, neat, cm^{-1}) = 3373, 2838, 1716, 1459, 1385, 1256, 1206, 1187, 1142, 1118.

m.p. = 180 – 182 °C.

Synthesis of Enalapril bicyclic derivatives

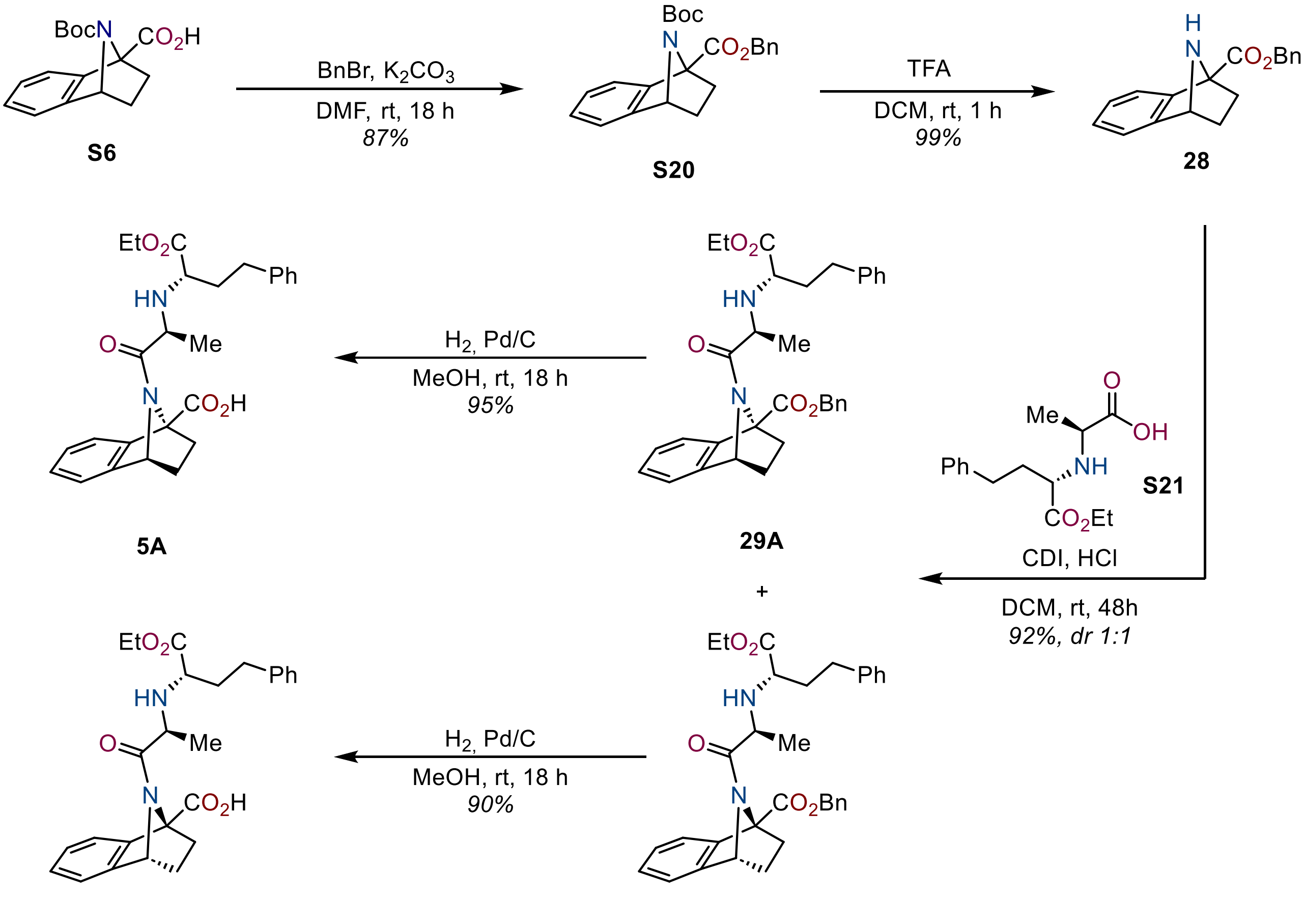

Scheme S8. Synthesis of Enalapril bicyclic analogues **5A** and **5B**.

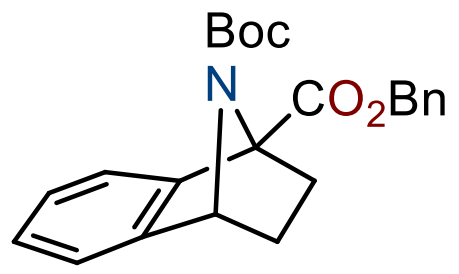

S20

Aza-bicyclic benzyl ester S20. To a solution of carboxylic acid **S6** (500 mg, 1.73 mmol, 1.0 eq.) in 9 mL of DMF (0.2 M) at room temperature, K_2CO_3 (1.19 g, 8.64 mmol, 5.0 eq.) was added, together with benzyl bromide (0.62 mL, 5.18 mmol, 3.0 eq.). The reaction mixture was stirred for 18 h at room temperature. The reaction was quenched with the addition of water, and the aqueous phase was extracted with Et_2O. The organic layers were combined, washed with brine, dried over Na_2SO_4, and evaporated under reduced pressure. The resulting residue was purified via flash column chromatography on silica gel (Hexane:Ethyl Acetate = 9:1) to afford benzyl ester **S20** (570 mg, 87%) as a colorless oil.

R_f = 0.25 (SiO_2, Hex:EtOAc 9:1, UV, Vanillin)

^{1}H NMR (400 MHz, $CDCl_3$) δ 7.46 – 7.43 (m, 2H), 7.39 – 7.32 (m, 4H), 7.26 – 7.23 (m, 1H), 7.20 – 7.13 (m, 2H), 5.38 (q, J = 12.4 Hz, 2H), 5.19 (d, J = 4.5 Hz, 1H), 2.51 (ddd, J = 11.7, 10.6, 3.9 Hz, 1H), 2.31 – 2.23 (m, 1H), 1.62 (ddd, J = 11.7, 8.9, 4.1 Hz, 1H), 1.40 – 1.34 (m, 10H).

^{13}C NMR (101 MHz, $CDCl_3$) δ 169.54, 156.05, 143.89, 143.80, 135.86, 128.56, 128.44, 128.29, 127.13, 126.84, 119.93, 119.61, 81.32, 72.60, 67.00, 63.29, 30.72, 28.04, 27.71.

HRMS: (ESI-TOF, m/z) $[M+Na]^+$ calcd. for $C_{23}H_{25}NO_4$ m/z 402.1676 and found m/z 402.1660.
IR: (ATR, neat, cm^{-1}) = 2976, 1705, 1628, 1537, 1498, 1457, 1390, 1340, 1310, 1265, 1075, 1028, 916.

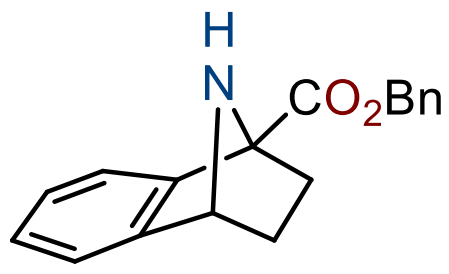

28

Aza-bicyclic benzyl ester 28. Boc-protected amino acid **S20** (250 mg, 0.66 mmol, 1.0 eq.) was solubilized in 7 mL of DCM (0.1 M). Under stirring, 0.66 mL of trifluoroacetic acid (1.0 M) were added slowly at 0 °C. The mixture was then stirred at room temperature for 1 h. After adjusting the pH-value to 9 by addition of solid $NaHCO_3$, the solution was extracted three times with DCM. The combined organic phases were dried over Na_2SO_4, filtered and the solvent was removed under reduced pressure. Free amine **28** (182 mg, 99%) was obtained as a colorless oil and it was used without further purification.
R_f = 0.21 (SiO_2, Hex:EtOAc 3:7, UV, Ninhydrin)
^{1}H NMR (400 MHz, $CDCl_3$) δ 7.44 – 7.30 (m, 6H), 7.26 – 7.21 (m, 1H), 7.16 – 7.09 (m, 2H), 5.36 (s, 2H), 4.58 (d, J = 3.7 Hz, 1H), 2.63 (s, 1H), 2.28 – 2.18 (m, 2H), 1.66 – 1.59 (m, 1H), 1.59 – 1.27 (m, 1H).
^{13}C NMR (101 MHz, $CDCl_3$) δ 171.25, 147.92, 146.23, 135.72, 128.70, 128.47, 128.43, 126.86, 126.30, 119.51, 119.13, 72.45, 66.93, 60.70, 30.82, 28.03.
HRMS: (ESI-TOF, m/z) $[M+H]^+$ calcd. for $C_{18}H_{17}NO_2$ m/z 280.1332 and found m/z 280.1323.
IR: (ATR, neat, cm^{-1}) = 3210, 2948, 1727, 1684, 1602, 1455, 1340, 1312, 1127, 1105, 922.

Enalapril Bicyclic Benzyl ester 29. Mixed anhydride was prepared according to a known procedure [7]. N-[(S)-1-ethoxycarbonyl-3-phenylpropyl]-L-alanine **S21** (200 mg, 0.72 mmol, 2.0 eq.) was solubilized in 7 mL of DCM (0.1 M). CDI (116 mg, 0.72 mmol, 2.0 eq.) was added, followed by the addition of HCl (4 M in Dioxane, 36 µL, 0.4 eq.). The mixture was stirred for 10 minutes at room temperature, until complete consumption of **S21** and formation of mixed anhydride **27** (monitored via TLC). Then, benzyl ester analogue **28** (100 mg, 0.36 mmol, 1.0 eq.) was added as a solution of DCM (0.18 mL, 2 M). The mixture was stirred for 48 hours at room temperature. Once complete, the solvent was removed under reduced pressure, and the crude product was purified via flash column chromatography on silica gel (Hexane:Ethyl Acetate = 1:1) to afford Enalapril bicyclic analogue **29** as a 1:1 mixture of diastereomers (180 mg, 92%, both colorless oil).

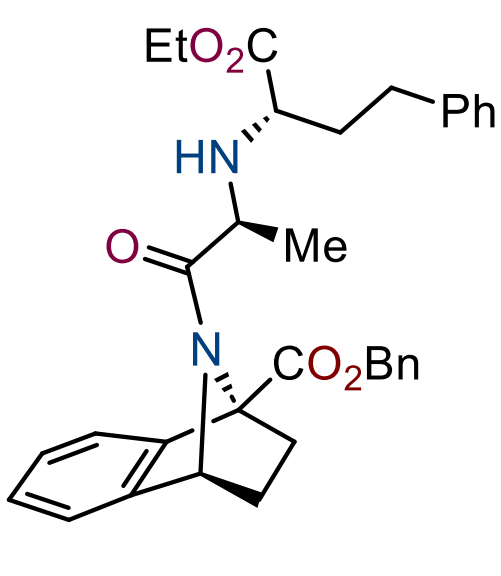

29A

Diastereomer 29A:

R$_f$ = 0.52 (SiO_2, Hex:EtOAc 1:1, UV, $KMnO_4$).

^{1}H NMR (400 MHz, $CDCl_3$) δ 7.34 – 7.32 (m, 2H), 7.28 – 7.04 (m, 13H), 5.33 – 5.23 (m, 3H), 4.11 (td, *J* = 7.2, 6.5 Hz, 2H), 3.45 (q, *J* = 6.9 Hz, 1H), 3.13 (t, *J* = 6.5 Hz, 1H), 2.56 – 2.46 (m, 3H), 2.15 – 2.10 (m, 1H), 1.91 – 1.86 (m, 2H), 1.52 (ddd, *J* = 12.1, 8.8, 3.8 Hz, 1H), 1.36 (ddd, *J* = 11.6, 8.8, 3.9 Hz, 1H), 1.20 (t, *J* = 7.1 Hz, 3H), 1.12 (d, *J* = 6.9 Hz, 3H).

^{13}C NMR (101 MHz, $CDCl_3$) δ 174.61, 174.56, 168.81, 143.52, 143.40, 141.29, 135.90, 128.44, 128.43, 128.41, 128.16, 127.29, 127.21, 125.98, 119.86, 119.03, 71.47, 67.31, 62.06, 60.85, 59.83, 54.44, 35.23, 32.00, 29.80, 28.77, 19.60, 14.34.

HRMS: (ESI-TOF, m/z) $[M+H]^+$ calcd. for $C_{33}H_{36}N_2O_5$ m/z 541.2697 and found m/z 541.2710.

IR: (ATR, neat, cm^{-1}) = 2937, 1729, 1664, 1450, 1407, 1247, 1165, 1027, 874, 841.

$α_D^{25.0}$ = -32.6 (c=0.07, MeOH).

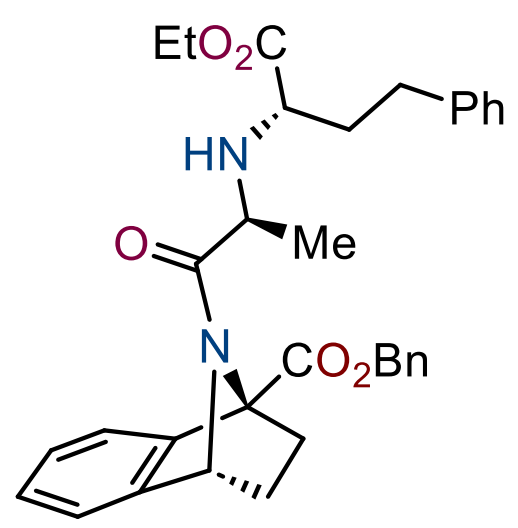

29B

Diastereomer 29B:

R$_f$ = 0.34 (SiO_2, Hex:EtOAc 1:1, UV, $KMnO_4$).

^{1}H NMR (400 MHz, $CDCl_3$) δ 7.40 (dd, J = 6.0, 2.5 Hz, 1H), 7.26 – 7.24 (m, 2H), 7.20 – 6.91 (m, 12H), 5.35 (d, J = 4.4 Hz, 1H), 5.19 (q, J = 12.4 Hz, 2H), 3.96 – 3.89 (m, 2H), 3.36 (q, J = 6.8 Hz, 1H), 2.92 (t, J = 6.4 Hz, 1H), 2.40 – 2.32 (m, 3H), 2.10 – 2.09 (m, 1H), 1.73 – 1.71 (m, 2H), 1.43 (ddd, J = 11.5, 8.9, 3.9 Hz, 1H), 1.27 (ddd, J = 11.5, 8.9, 3.9 Hz, 1H), 1.08 (d, J = 6.9 Hz, 3H), 1.03 (t, J = 7.1 Hz, 3H).

^{13}C NMR (101 MHz, $CDCl_3$) δ 175.52, 174.58, 169.00, 143.88, 143.83, 141.42, 135.94, 128.52, 128.44, 128.41, 128.21, 127.17, 127.14, 125.98, 120.95, 118.95, 71.02, 67.29, 62.20, 60.79, 59.72, 55.29, 35.45, 31.97, 30.43, 29.29, 19.65, 14.29.

HRMS: (ESI-TOF, m/z) $[M+H]^+$ calcd. for $C_{33}H_{36}N_2O_5$ m/z 541.2697 and found m/z 541.2713.

IR: (ATR, neat, cm^{-1}) = 2980, 1727, 1665, 1535, 1496, 1451, 1388, 1315, 1246, 1185, 1075, 1026, 948.

$α_D^{25.0}$ = -40.1 (c=0.07, MeOH).

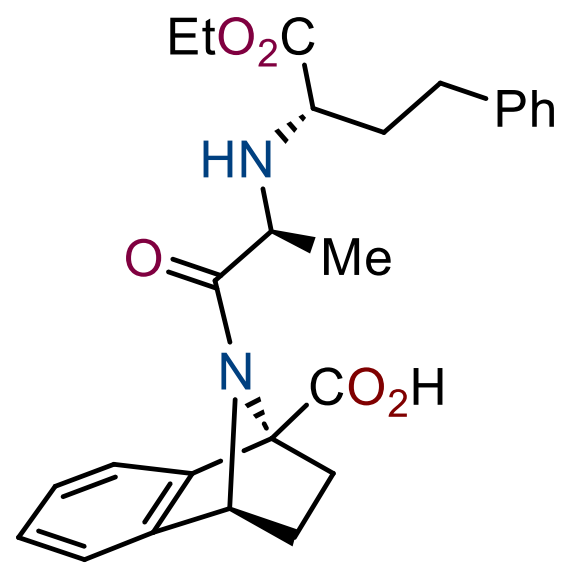

5A

Enalapril Bicyclic analogue 5A. Benzyl protected enalapril isomer A **29A** (50 mg, 0.09 mmol, 1.0 eq.) was solubilized in 1 mL of MeOH (0.1 M). The mixture was purged with Ar, then Pd/C (10% Pd on C, 10 mg, 0.10 eq.) was added to the mixture. The flask was filled with H_2 gas using a double balloon, and the reaction mixture was stirred for 24 h at room temperature under an atmosphere of H_2. Once complete, the mixture was filtered over a pad of celite, using MeOH as the eluent. The solvent was evaporated under reduced pressure, and the Enalapril hybrid Analogue **5A** was obtained (39.5 mg, 95%) as a white solid.

R_f = 0.40 (SiO_2, Hex:EtOAc 3:7, UV, $KMnO_4$).

^{1}H NMR (400 MHz, MeOD) δ 7.49 – 7.47 (m, 1H), 7.39 – 7.37 (m, 1H), 7.34 – 7.20 (m, 7H), 5.57 (d, J = 4.4 Hz, 1H), 4.46 (q, J = 6.9 Hz, 1H), 4.35 (q, J = 7.1 Hz, 2H), 3.89 (dd, J = 6.7, 5.6 Hz, 1H), 2.79 (dddd, J = 37.0, 13.9, 9.1, 6.7 Hz, 2H), 2.49 (ddd, J = 11.6, 10.5, 3.8 Hz, 1H), 2.27 (dddd, J = 15.5, 14.1, 7.1, 4.1 Hz, 3H), 1.60 – 1.56 (m, 1H), 1.49 – 1.41 (m, 4H), 1.36 (t, J = 7.1 Hz, 3H).

^{13}C NMR (101 MHz, MeOD) δ 171.36, 169.67, 168.55, 144.68, 144.37, 140.86, 129.72, 129.56, 128.75, 128.45, 127.64, 121.23, 120.56, 73.33, 64.15, 63.47, 59.78, 55.79, 32.85, 31.87, 30.81, 30.15, 16.22, 14.40.

HRMS: (ESI-TOF, m/z) $[M-H]^-$ calcd. for $C_{26}H_{30}N_2O_5$ m/z 449.2082 and found m/z 449.2103.

IR: (ATR, neat, cm^{-1}) = 3150, 2581, 1742, 1712, 1636, 1552, 1455, 1438, 1407, 1380, 1362, 1246, 1213, 1053, 840.

m.p. = 168 - 171 °C.

$\alpha_D^{25.0}$ = -20.2 (c=0.80, MeOH).

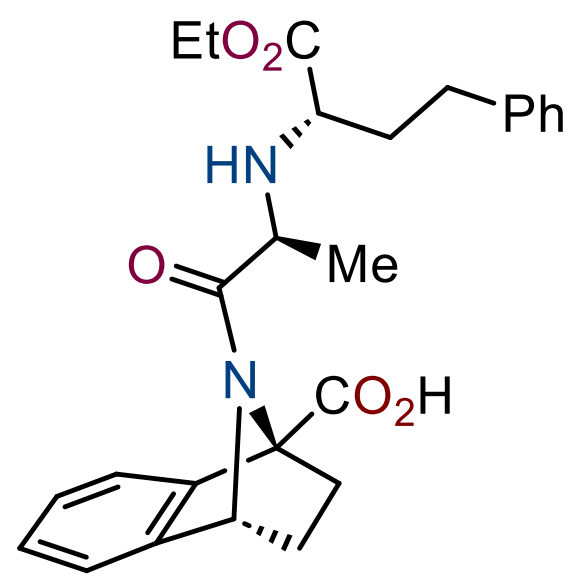

5B

Enalapril Bicyclic analogue 5B. Benzyl protected enalapril isomer B **29B** (50 mg, 0.09 mmol, 1.0 eq.) was solubilized in 1 mL of MeOH (0.1 M). The mixture was purged with Ar, then Pd/C (10% Pd on C, 10 mg, 0.10 eq.) was added to the mixture. The flask was filled with H_2 gas using a double balloon, and the reaction mixture was stirred for 24 h at room temperature under an atmosphere of H_2. Once complete, the mixture was filtered over a pad of celite, using MeOH as the eluent. The solvent was evaporated under reduced pressure, and the Enalapril hybrid analogue **5B** was obtained (37.5 mg, 90%) as a white solid.

R_f = 0.29 (SiO_2, Hex:EtOAc 3:7, UV, $KMnO_4$).

^{1}H NMR (400 MHz, $CDCl_3$) δ 7.61 – 7.59 (m, 1H), 7.25 – 6.99 (m, 8H), 5.49 (d, *J* = 4.3 Hz, 1H), 4.23 – 4.13 (m, 2H), 3.62 (q, *J* = 7.0 Hz, 1H), 3.49 – 3.45 (m, 1H), 2.59 – 2.55 (m, 3H), 2.41 (dddd, *J* = 12.0, 10.6, 8.7, 6.2 Hz, 1H), 2.18 – 2.11 (m, 2H), 1.81 – 1.80 (m, 1H), 1.47 (d, *J* = 7.0 Hz, 3H), 1.41 (ddd, *J* = 10.5, 8.5, 3.1 Hz, 1H), 1.22 (t, *J* = 7.1 Hz, 3H).

^{13}C NMR (101 MHz, $CDCl_3$) δ 169.69, 164.80, 162.13, 143.56, 142.10, 140.40, 128.61, 128.20, 127.55, 127.17, 126.28, 119.80, 119.50, 66.75, 61.50, 60.55, 59.82, 58.19, 32.61, 32.56, 30.17, 27.50, 20.41, 14.18.

HRMS: (ESI-TOF, m/z) $[M+H]^+$ calcd. for $C_{26}H_{30}N_2O_5$ m/z 451.2227 and found m/z 451.2232.

IR: (ATR, neat, cm^{-1}) = 3421, 2933, 1735, 1671, 1647, 1541, 1438, 1410, 1241, 1216, 1172, 1021, 932.
m.p. = 163 - 165 °C.
$\alpha_D^{25.0}$ = +7.6 (c=0.75, MeOH).

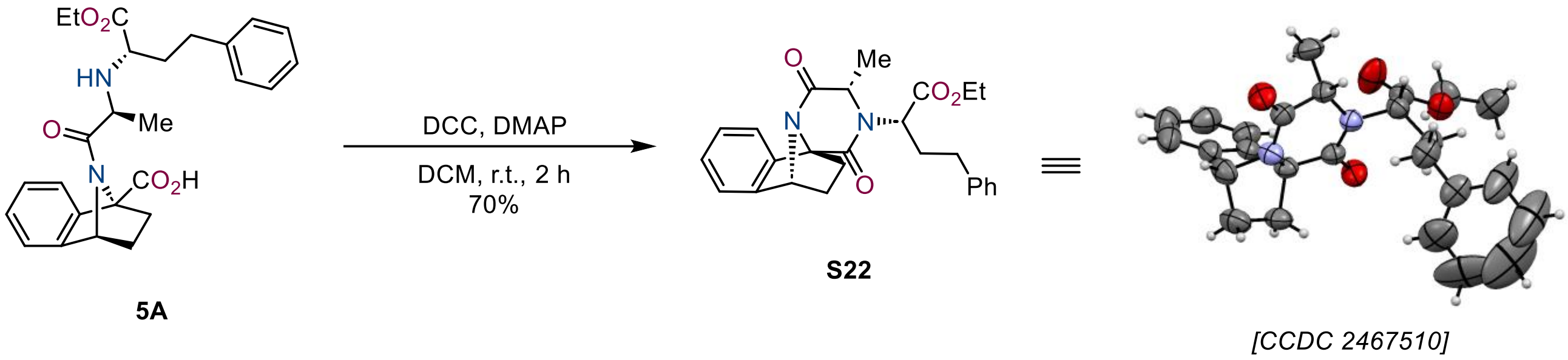

Scheme S9. Derivatization of Enalapril hybrid analogue **5A.**

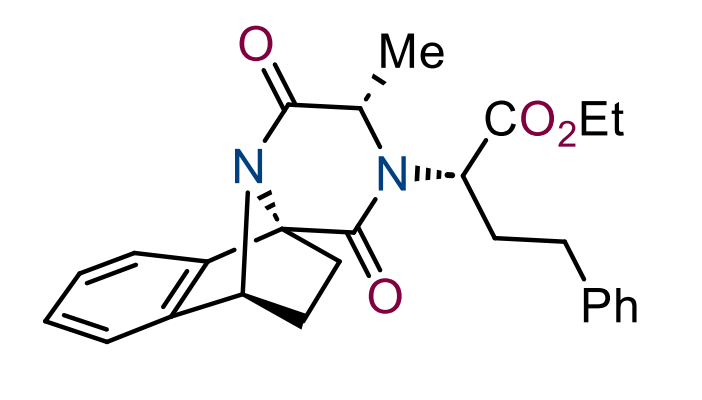

S22

Enalapril Bicyclic Analogue Derivatization S22. To a stirred solution of acid **5A** (25 mg, 0.055 mmol, 1.0 equiv.) dissolved in dry DCM (0.6 mL, 0.1 M), were added sequentially 4-DMAP (0.68 mg, 0.006 mmol, 0.1 equiv) and DCC (13 mg, 0.063 mmol, 1.1 equiv.). After 2 h of stirring the reaction was diluted with hexanes and washed with water, dried over Na_2SO_4, filtered and concentrated under reduced pressure. The residue was purified via flash column chromatography on silica gel (Hexane:Ethyl Acetate = 8:2) to afford the corresponding amide **S22** (17 mg, 70%) as a white crystalline solid.
R_f = 0.23 (SiO_2, Hex:EtOAc 8:2, UV, $KMnO_4$).
^{1}H NMR (400 MHz, $CDCl_3$) δ 7.38 – 7.36 (m, 1H), 7.32 – 7.26 (m, 3H), 7.23 – 7.17 (m, 5H), 5.54 – 5.53 (m, 1H), 4.26 (q, J = 7.1 Hz, 2H), 3.76 (q, J = 7.0 Hz, 1H), 3.60 (dd, J = 9.1, 5.3 Hz, 1H), 2.79 – 2.74 (m, 2H), 2.63 – 2.60 (m, 1H), 2.50 (ddt, J = 14.5, 9.1, 7.2 Hz, 1H), 2.25 – 2.21 (m, 2H), 1.90 (dd, J = 8.9, 7.8 Hz, 1H), 1.44 – 1.39 (m, 1H), 1.29 (t, J = 7.1 Hz, 3H), 1.11 (d, J = 7.0 Hz, 3H).
^{13}C NMR (101 MHz, $CDCl_3$) δ 169.70, 165.65, 165.47, 144.54, 143.77, 140.58, 128.84, 128.37, 127.57, 127.22, 126.48, 120.54, 118.58, 67.80, 61.62, 61.14, 60.05, 59.68, 33.05, 32.70, 30.25, 26.22, 18.85, 14.25.
HRMS: (ESI-TOF, m/z) $[M+H]^+$ calcd. for $C_{26}H_{28}N_2O_4$ m/z 433.2122 and found m/z 433.2115.
IR: (ATR, neat, cm^{-1}) = 1731, 1662, 1494, 1477, 1448, 1407, 1369, 1237, 1211, 1192, 1166, 1112, 1026, 963, 747.
m.p. = 110 - 113 °C.
$\alpha_D^{25.0}$ = +31.5 (c=0.60, MeOH).

PhysChem Analysis

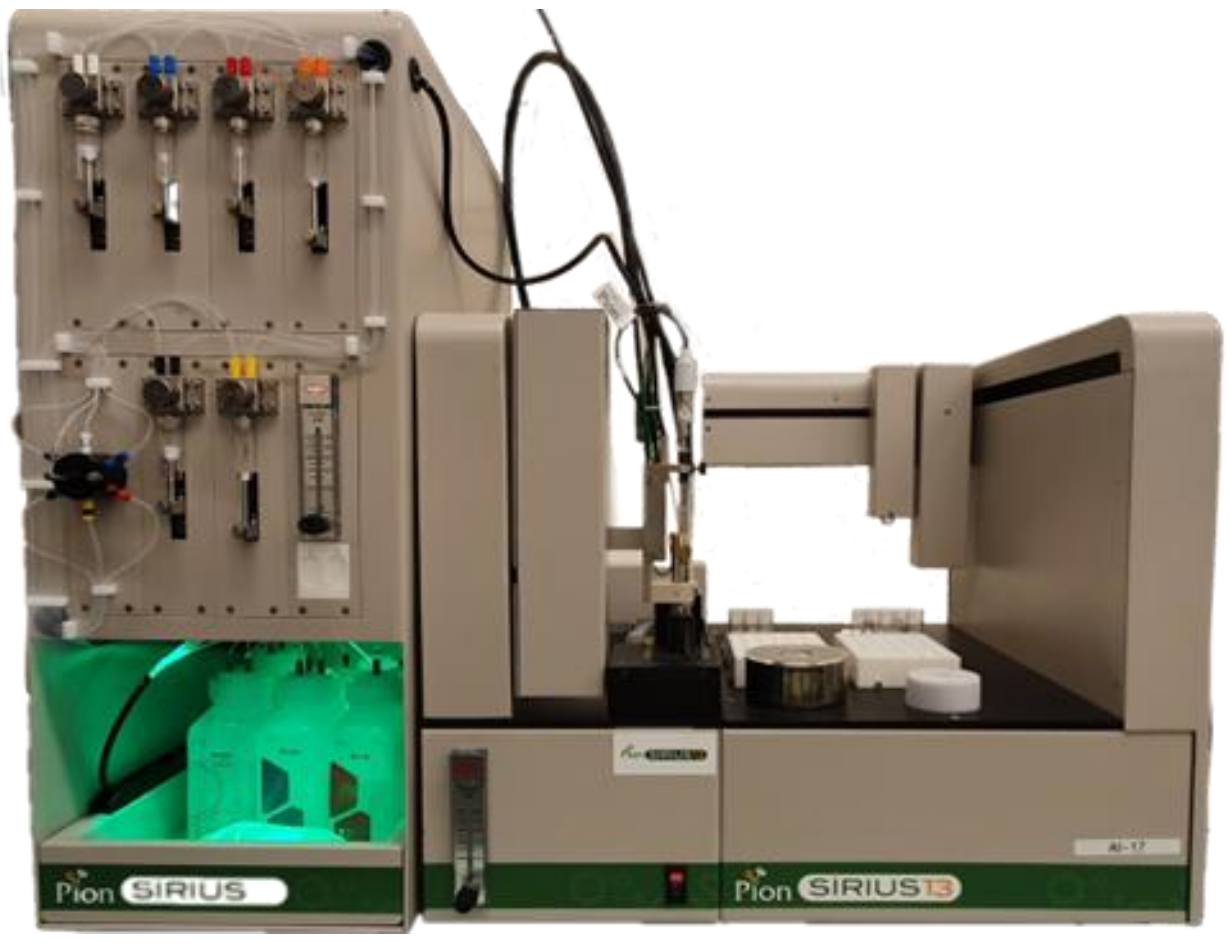

Figure **S1**. Sirius T3 automated pH-metric titrator.

Sirius T3 is an automated instrument that allows the screening of compounds and the preparation of detailed physical chemical profiles. The device consists of a pH-meter electrode, a robot that prepares the solutions needed to perform the measurements, and a UV-VIS spectrophotometer. If basic titration is needed, basic titration will be performed with a 0.1 M KOH buffer. If acidic titration is needed, this titration will be performed with a 0.1 M HCl buffer.

pKa Measurement

To obtain the pKa value of our molecules, a pH-metric titration experiment was carried out. Potentiometric measurement was done from pH 2 to pH 12 by adding 0.5 M KCl solution. Measurement point was done every 0.2 pH value. After each addition, the sample is stirred for 60 seconds, and the pH value is then collected. The titration experiment was done 3 times. The given value is an average value of the 3 measurements.

Log P/Log D Measurement

Log P was determined potentiometrically by using the "Shake flask" method, which consists of dissolving part of the solute in question in a volume of octanol and water, then performing pH titration. LogD was determined as the value of log P at a pH where the molecule was completely not ionised.

Solubility Measurement

Solubility measurements were performed using the CheqSol method developed by Pion. As in most cases, compounds are more soluble at pH where they ionized, the analyte was solubilized in acidic water, then pH-titration was performed, measuring the UV-Vis spectrum after each addition. The precipitation point was detected from the reduction of the light transmission during UV-VIS measurement, or manually when not obvious. The pH-titration was repeated several times around the precipitation point to determine the solubility value by averaging.

Results

Sample	Structure	Theoretical pKa[a] (in water) value	pKa (in water) value	Theoretical Log P[a] value	Log P Value	Theoretical Solubility[a] value	Solubility value
**1**	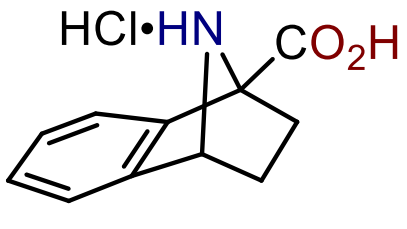 **16**	1.98<br>8.72	1.75 ± 0.02<br>9.25 ± 0.01	0.54	0.64 ± 0.03	61.26 mM	19.18 mM at pH 7.4
**2**	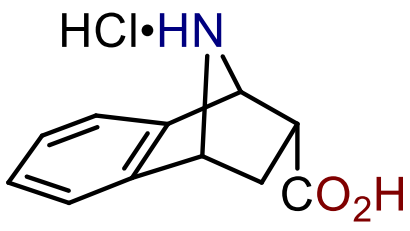 **18**	3.62<br>9.35	3.26 ± 0.01<br>8.89 ± 0.01	0.97	-0.08 ± 0.04	34.92 mM	18.13 mM at pH 7.4
**3**	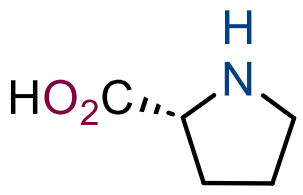	2.35<br>11.11	2.17 ± 0.02<br>10.55 ± 0.01	-0.57	-0.17 ± 0.02	3.74 M	Odl
**4**	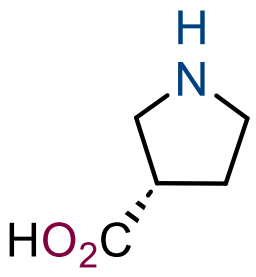	3.86<br>11.14	3.39 ± 0.01<br>10.48 ± 0.01	-0.47	-0.84 ± 0.08	3.45 M	Odl
**5**	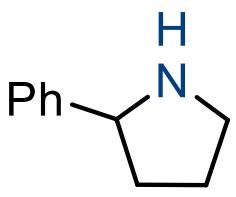	1.91<br>10.75	2.48 ± 0.03<br>10.05 ± 0.01	0.96	1.50 ± 0.05	124.10 mM	155.20 mM at pH 7.4
**6**	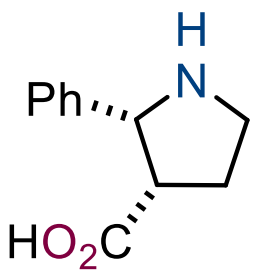	3.70<br>10.77	3.22 ± 0.02<br>9.38 ± 0.01	1.23	1.01 ± 0.03	73.10 mM	165.10 mM at pH 7.4

a. Theoretical value predicted by ChemDraw. Odl = Over Detection Limit.

Table S2. Physicochemical measurements of proline-based compounds and hybrid analogues.

Crystallographic Data

Diffraction data for colourless single crystals of the two compounds were collected on a Rigaku XtaLab SuperNova four circle diffractometer (Rigaku Europe SE, Neu-Isenburg, Germany) equipped by a microfocus X-ray source (λ = 0.7107 Å) and a Dectris Pilatus 200K hybrid pixel array detector (DECTRIS AG, Baden-Daettwil, Switzerland). 2D diffraction frames were processed with the CrysAlisPro software suite, ver. 1.171.41.[8] Empirical absorption-effect correction was applied to the data based on a multi-scan approach using spherical harmonics (SCALE3 ABSPACK algorithm of the CrysAlisPro suite). Crystal structures were solved by direct methods (SIR 97) [9] and refined by full-matrix least-square procedures on F2 using all reflections (SHELXL 2019/2).[10] Crystal data and plot showing thermal ellipsoids are reported on Table **S3**. CCDC deposition numbers 2467510 and 2467511 contain the supplementary crystallographic data for this paper. These data are provided free of charge by the joint Cambridge Crystallographic Data Centre and Fachinformationszentrum Karlsruhe Access Structures service.

Table S3. Crystal data of compounds **S22** and **(±)-S4**, respectively.

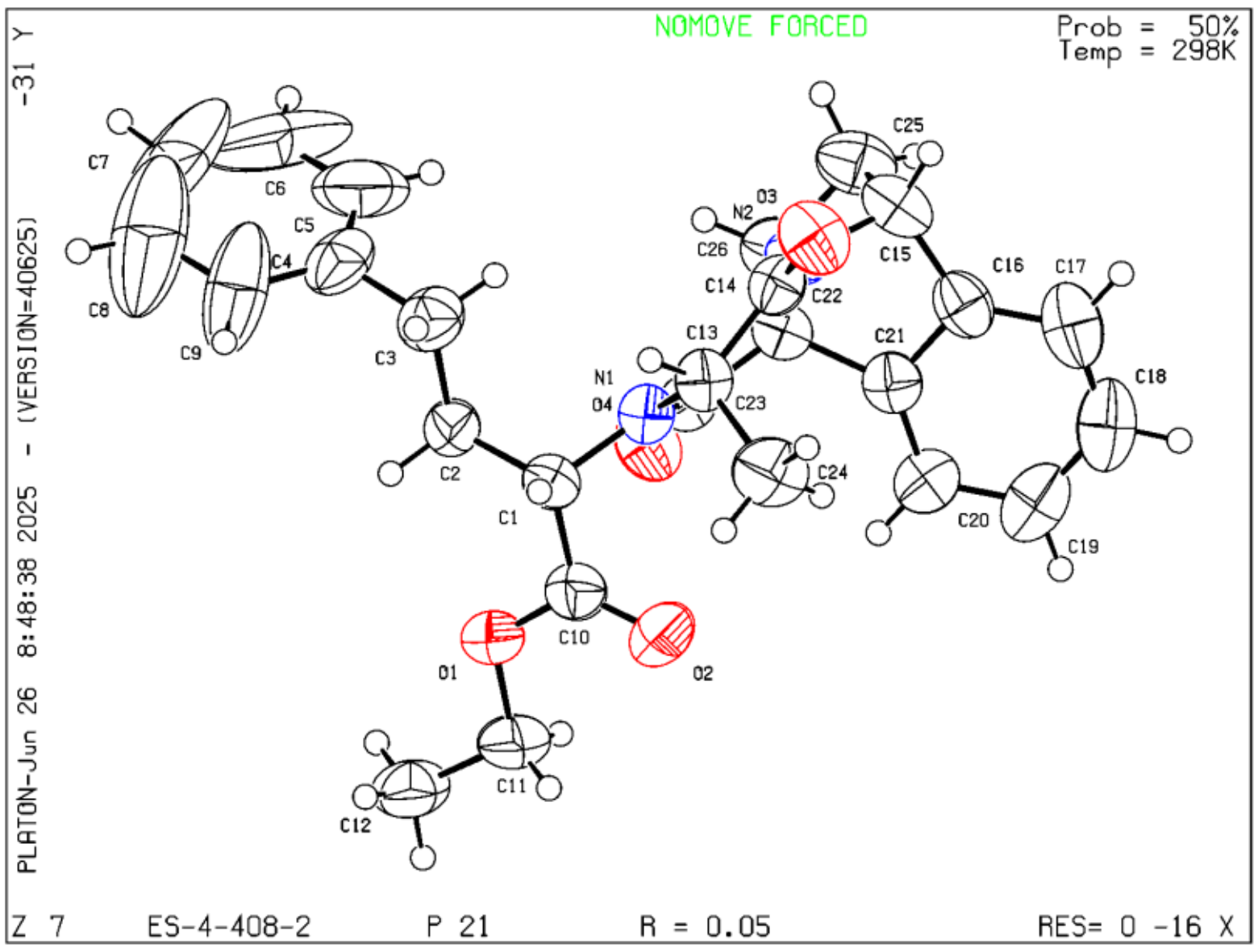

Identification Code	ES-4-408	
CCDC code	2467510	
Empirical formula	C_{26} H_{28} N_2 O_4	
Formula weight	432.50	
Temperature	298(2) K	
Wavelength	0.7107 Å	
Crystal system	Monoclinic	
Space group	*P*2$_1$ (no. 4)	
Unit cell dimensions	a = 10.5698(9) Å	α = 90°
	b = 10.0448(7) Å	β = 105.071(8)°

	c = 11.4750(10) Å	γ = 90°
Volume	1176.4(2) $Å^3$	
Z	2	
Density (calculated)	1.221 g/cm^3	
Absorption coefficient	0.083 mm^{-1}	
F(000)	460 e	
Crystal size	0.34 x 0.34 x 0.11 mm^3	
Theta range for data collection	1.84 to 25.05°.	
Index ranges	–12<=h<=12, –11<=k<=11, -13<=l<=13	
Reflections collected	13137	
Independent reflections	4114 [R(int) = 0.0249]	
Strong data [$I_O>2\sigma(I_O)$]	3453	
Completeness	100.0 %	
Absorption correction	empirical (multi-scan)	
Max. and min. transmission	0.990 and 0.721	
Refinement method	Full-matrix least-squares on F^2	
Data / restraints / parameters	4114 / 16 / 289	
Goodness-of-fit on F^2	1.089	
Final R indices (strong data)	R1 = 0.0519, wR2 = 0.1405	
Final R indices (all data)	R1 = 0.0610, wR2 = 0.1475	
Largest diff. peak and hole	0.329 and –0.252 e/$Å^3$	
Flack parameter	0.1(5)	

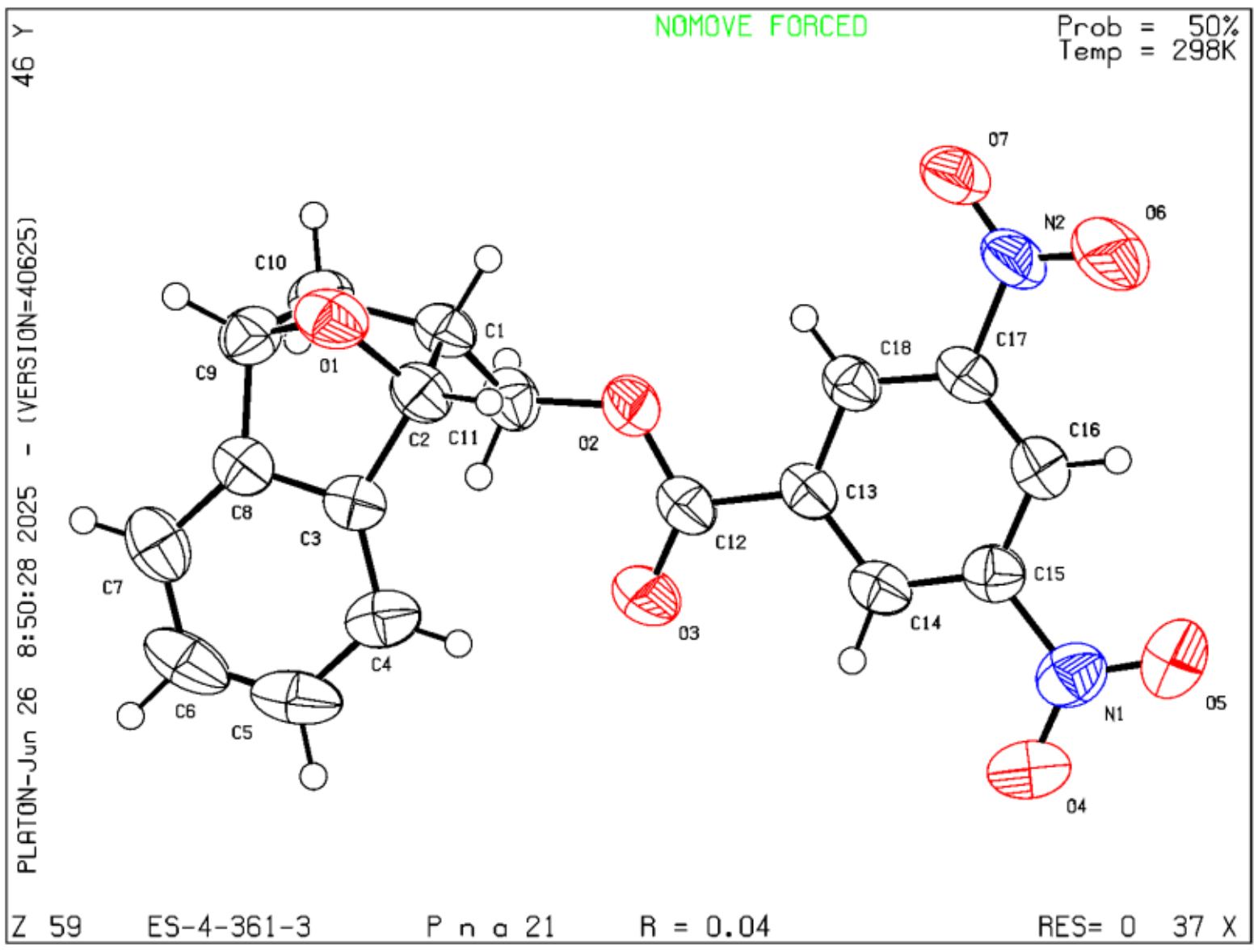

Identification Code	ES-4-361
CCDC code	2467511
Empirical formula	$C_{18} H_{14} N_2 O_7$
Formula weight	370.31
Temperature	298(2) K
Wavelength	0.7107 Å
Crystal system	Orthorhombic

Space group	*Pna*2$_1$ (no. 33)
Unit cell dimensions	a = 18.3818(8) Å α = 90°
	b = 9.3612(4) Å β = 90°
	c = 9.8194(4) Å γ = 90°
Volume	1689.68(12) Å^3
Z	4
Density (calculated)	1.456 g/cm^3
Absorption coefficient	0.114 mm^{-1}
F(000)	768 e
Crystal size	0.36 x 0.31 x 0.11 mm^3
Theta range for data collection	2.22 to 30.06°.
Index ranges	–25<=h<=25, –13<=k<=12, -13<=l<=13
Reflections collected	17421
Independent reflections	4889 [R(int) = 0.0199]
Strong data [$I_O > 2\sigma(I_O)$]	3997
Completeness	100.0 %
Absorption correction	empirical (multi-scan)
Max. and min. transmission	0.988 and 0.761
Refinement method	Full-matrix least-squares on F^2
Data / restraints / parameters	4889 / 1 / 244
Goodness-of-fit on F^2	1.056
Final R indices (strong data)	R1 = 0.0353, wR2 = 0.0941
Final R indices (all data)	R1 = 0.0448, wR2 = 0.0996
Largest diff. peak and hole	0.161 and –0.119 e/Å^3
Flack parameter	-0.1(3)

^{1}H and ^{13}C NMR spectra

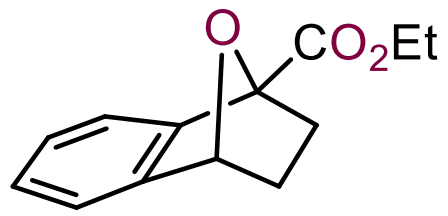

S1

^{1}H NMR, 400 MHz, $CDCl_3$

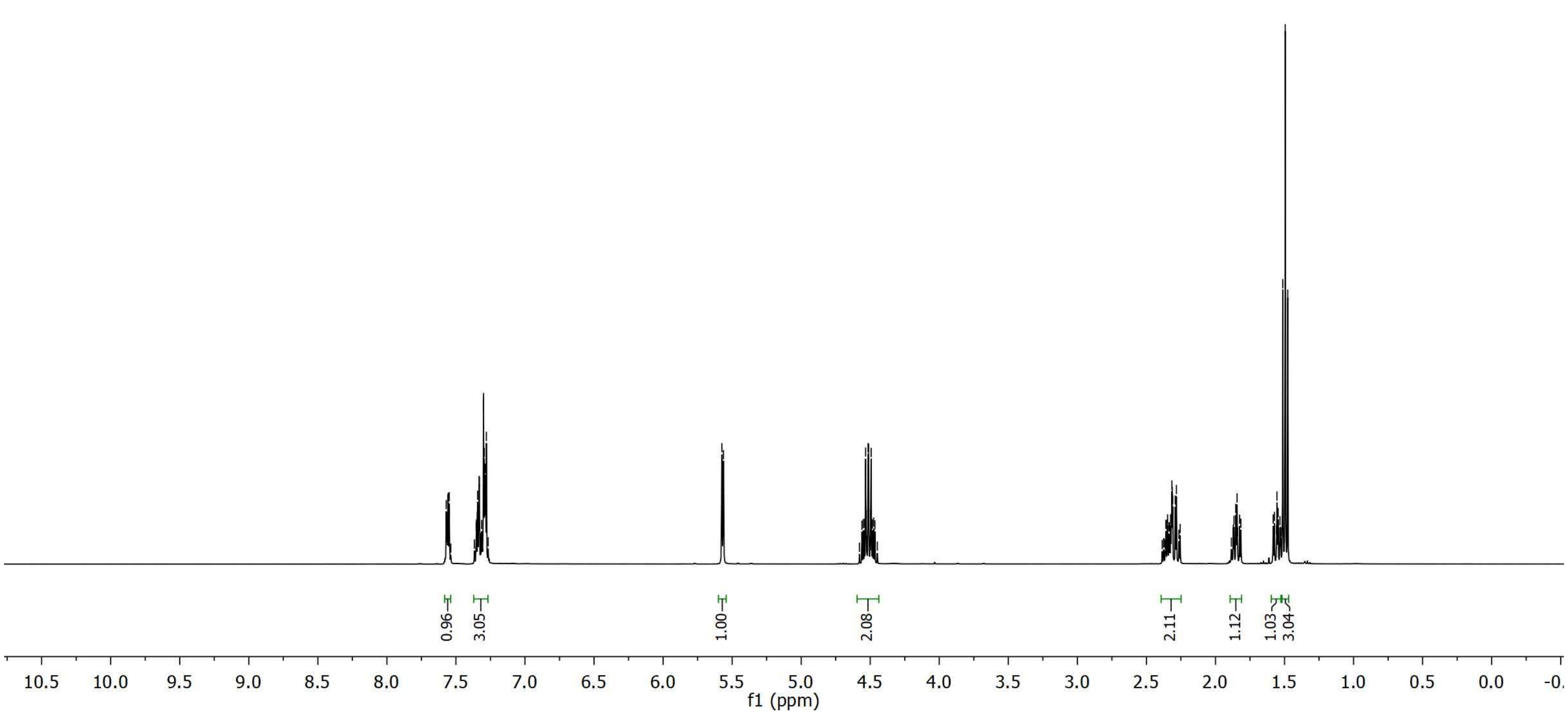

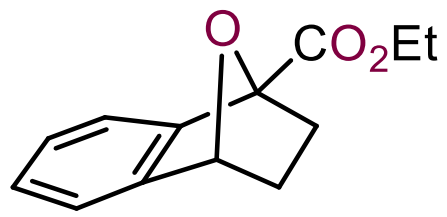

S1

^{13}C NMR, 101 MHz, $CDCl_3$

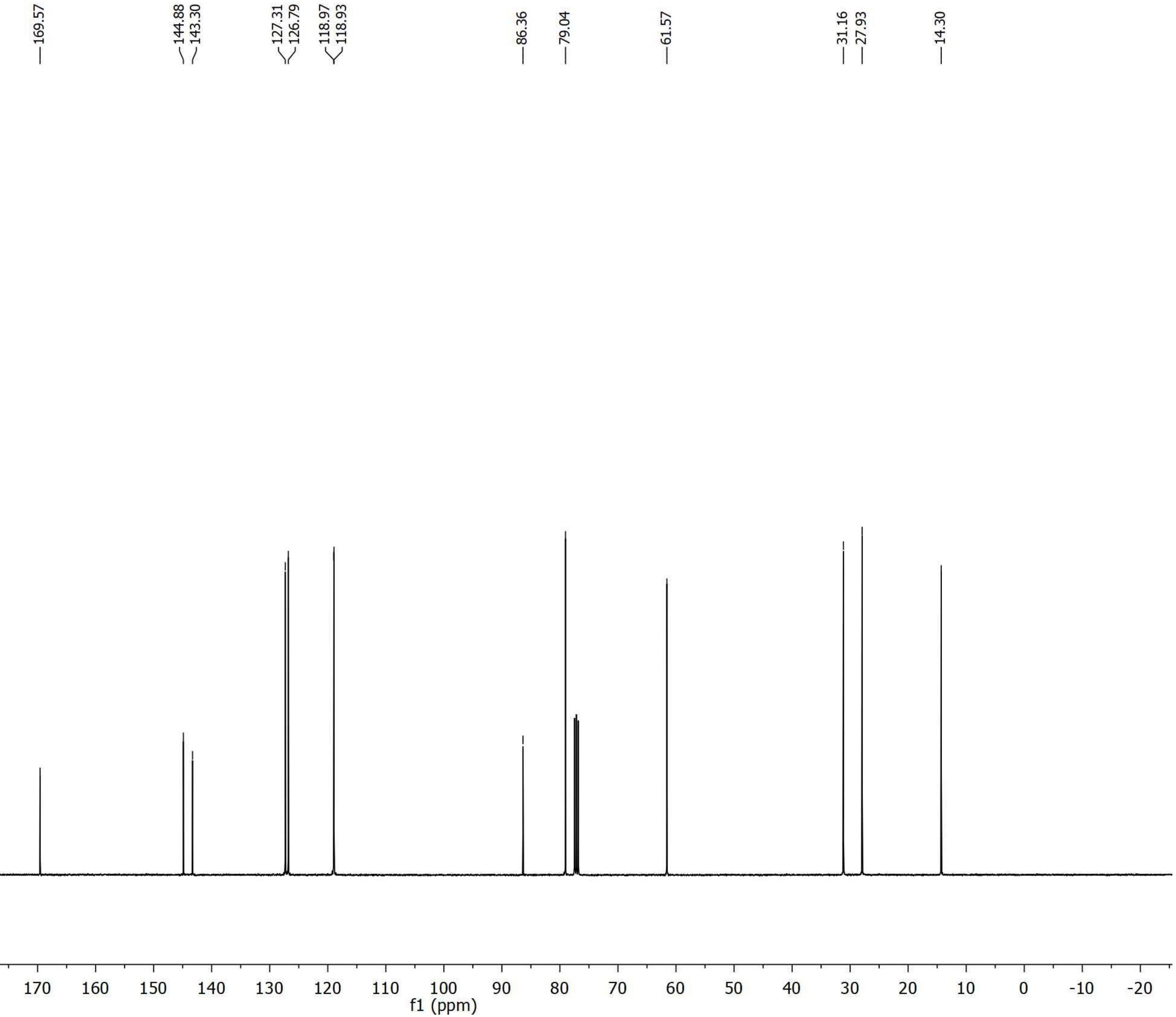

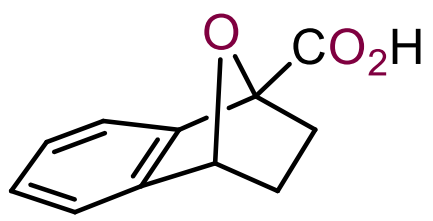

13

^{1}H NMR, 400 MHz, CD_3CN

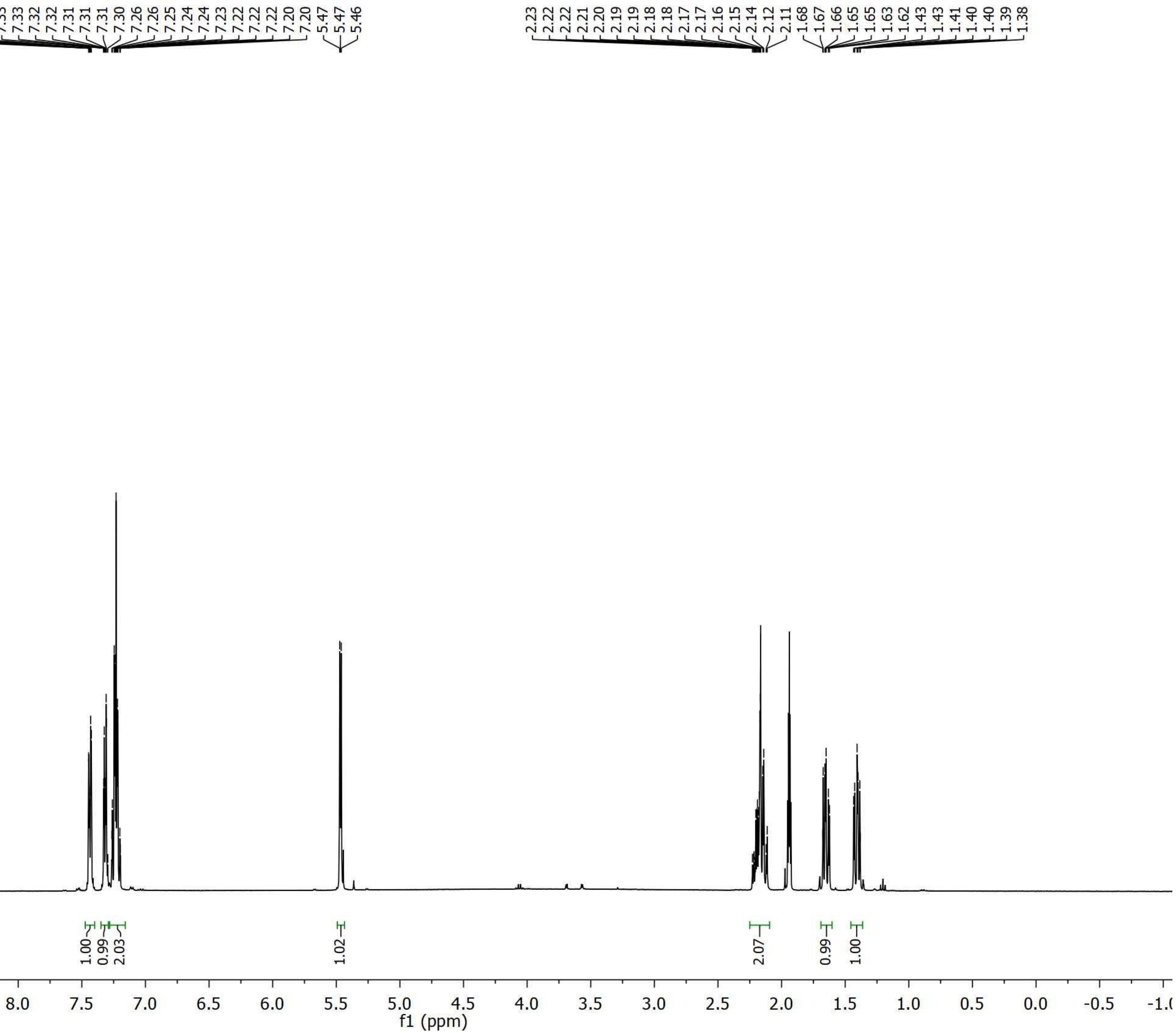

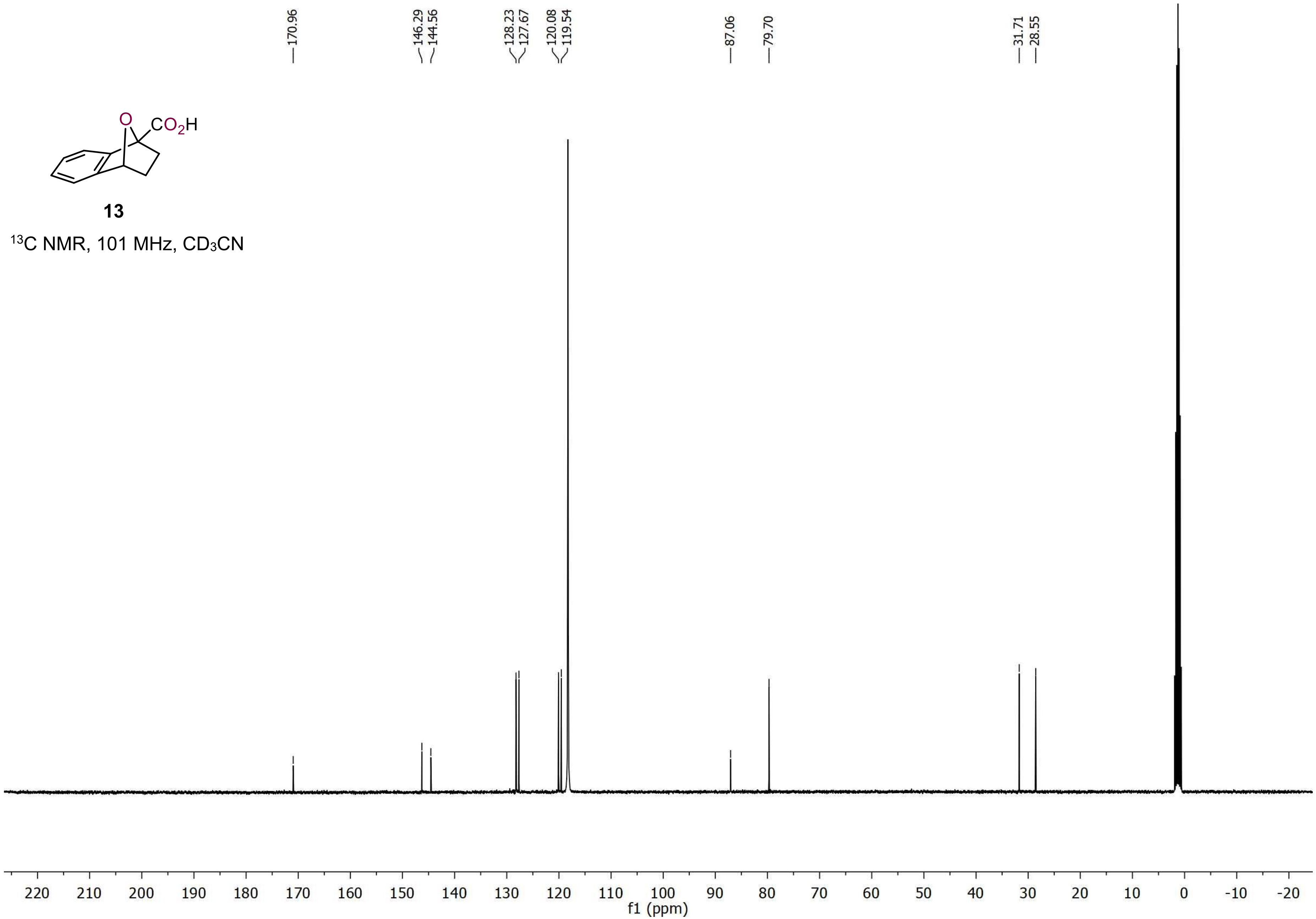

13

^{13}C NMR, 101 MHz, CD_3CN

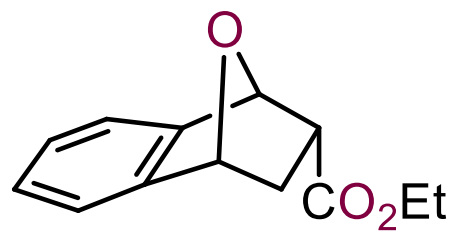

S2

^{1}H NMR, 400 MHz, $CDCl_3$

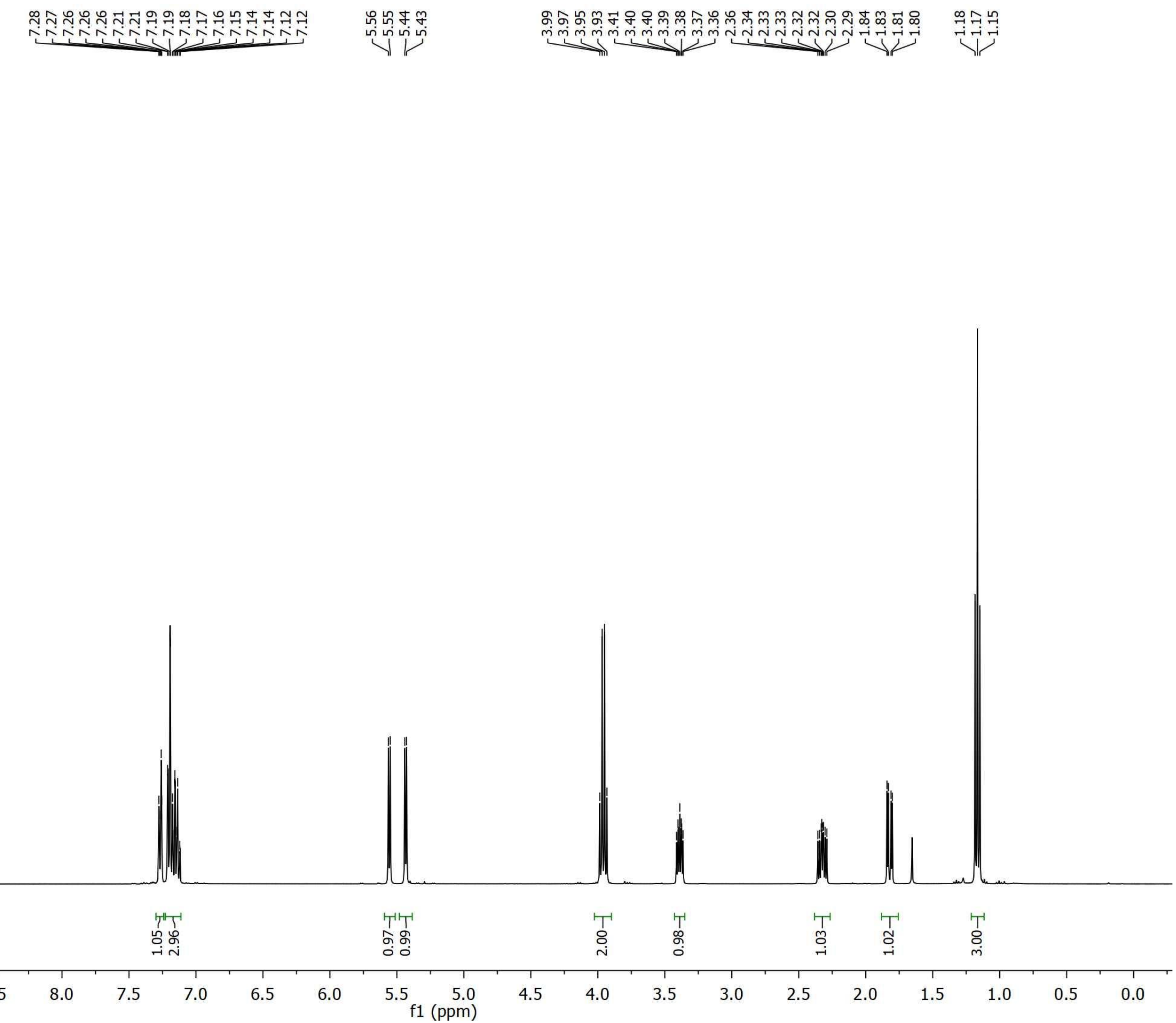

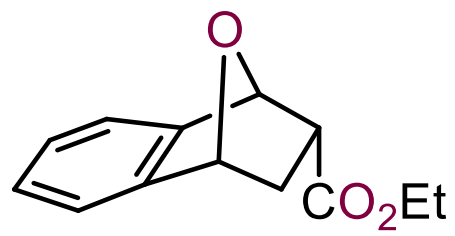

S2

^{13}C NMR, 101 MHz, $CDCl_3$

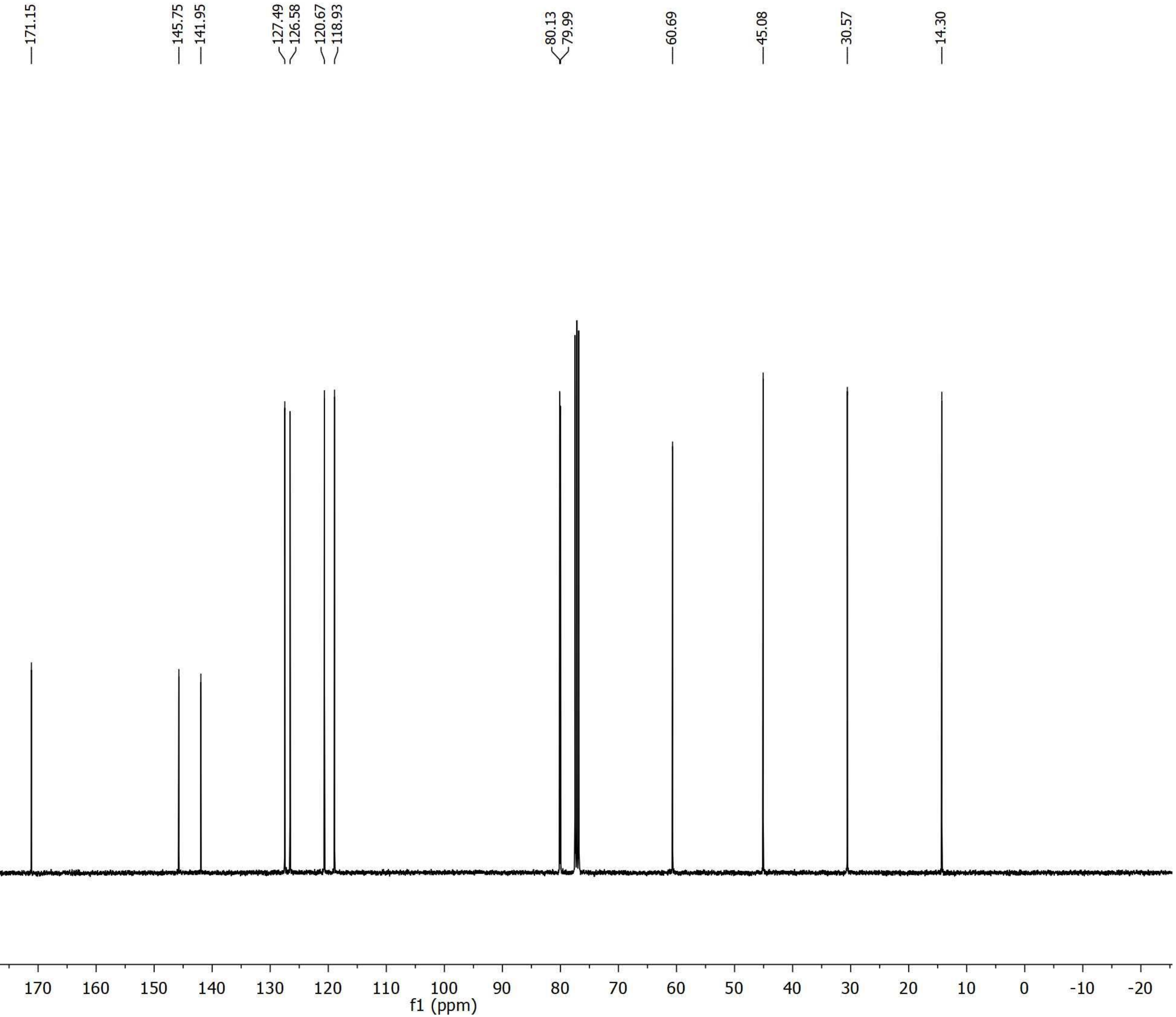

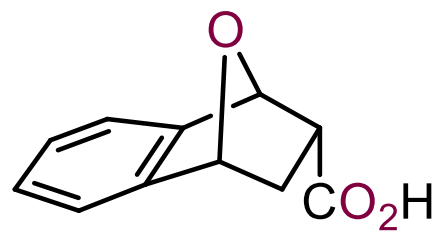

14

^{1}H NMR, 400 MHz, $CDCl_3$

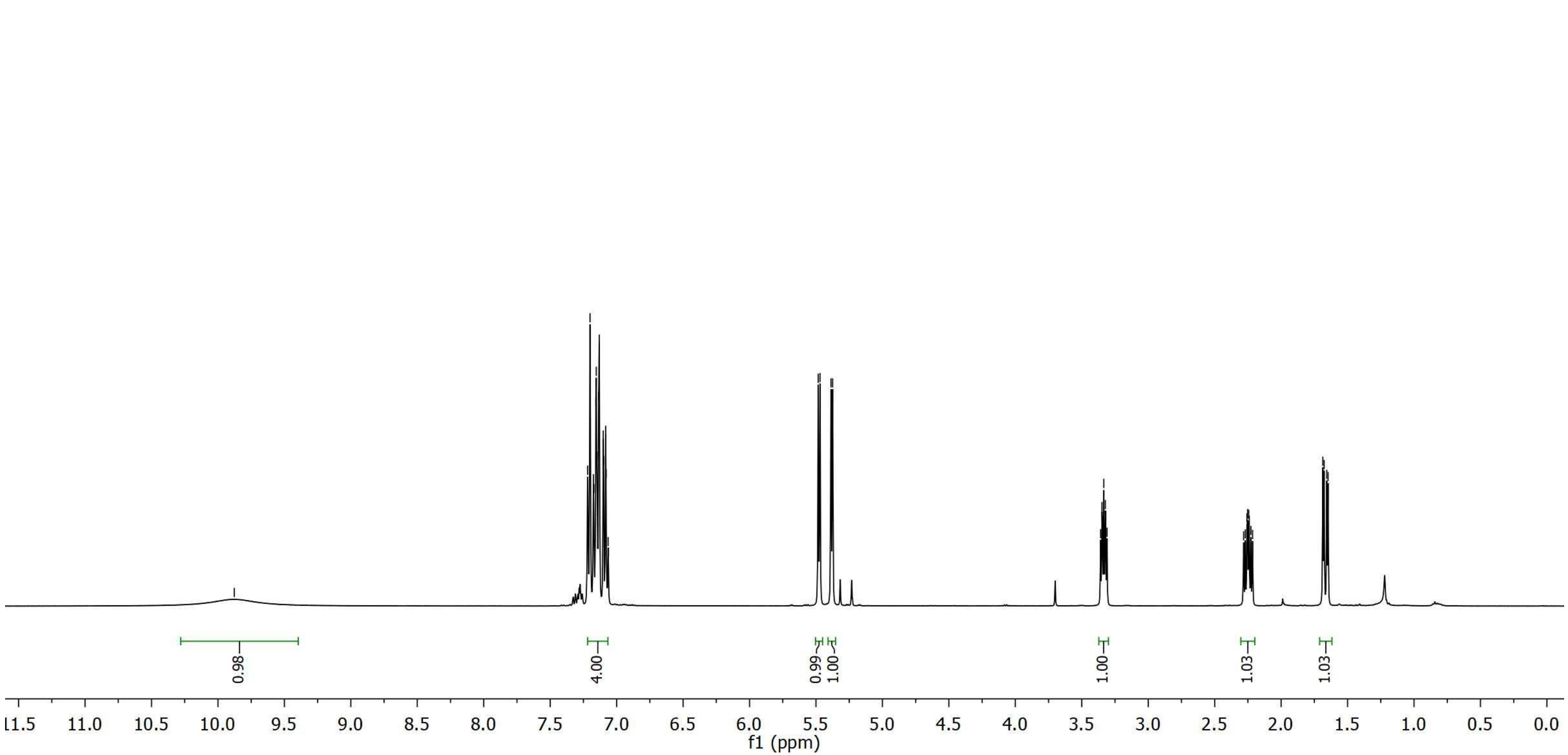

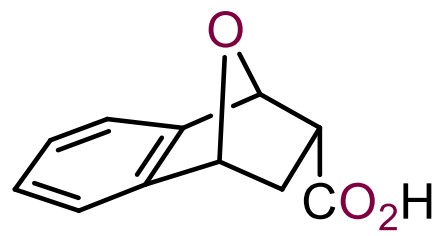

14

^{13}C NMR, 101 MHz, $CDCl_3$

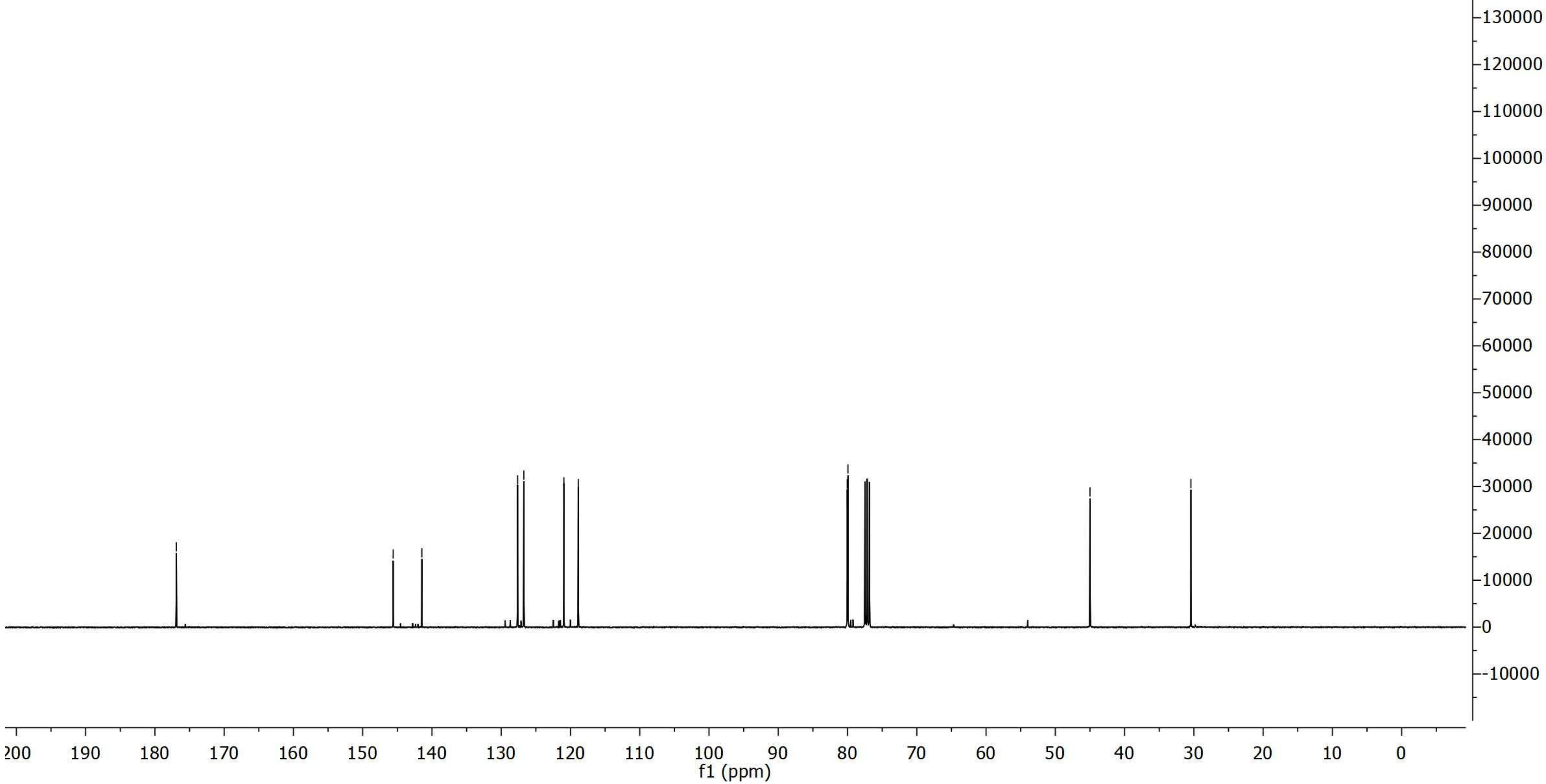

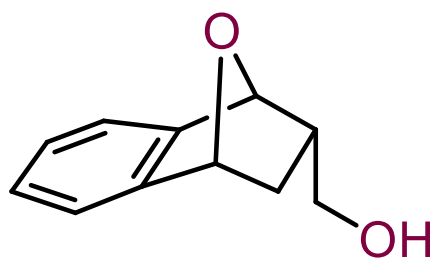

S3

^{1}H NMR, 400 MHz, CDCl3

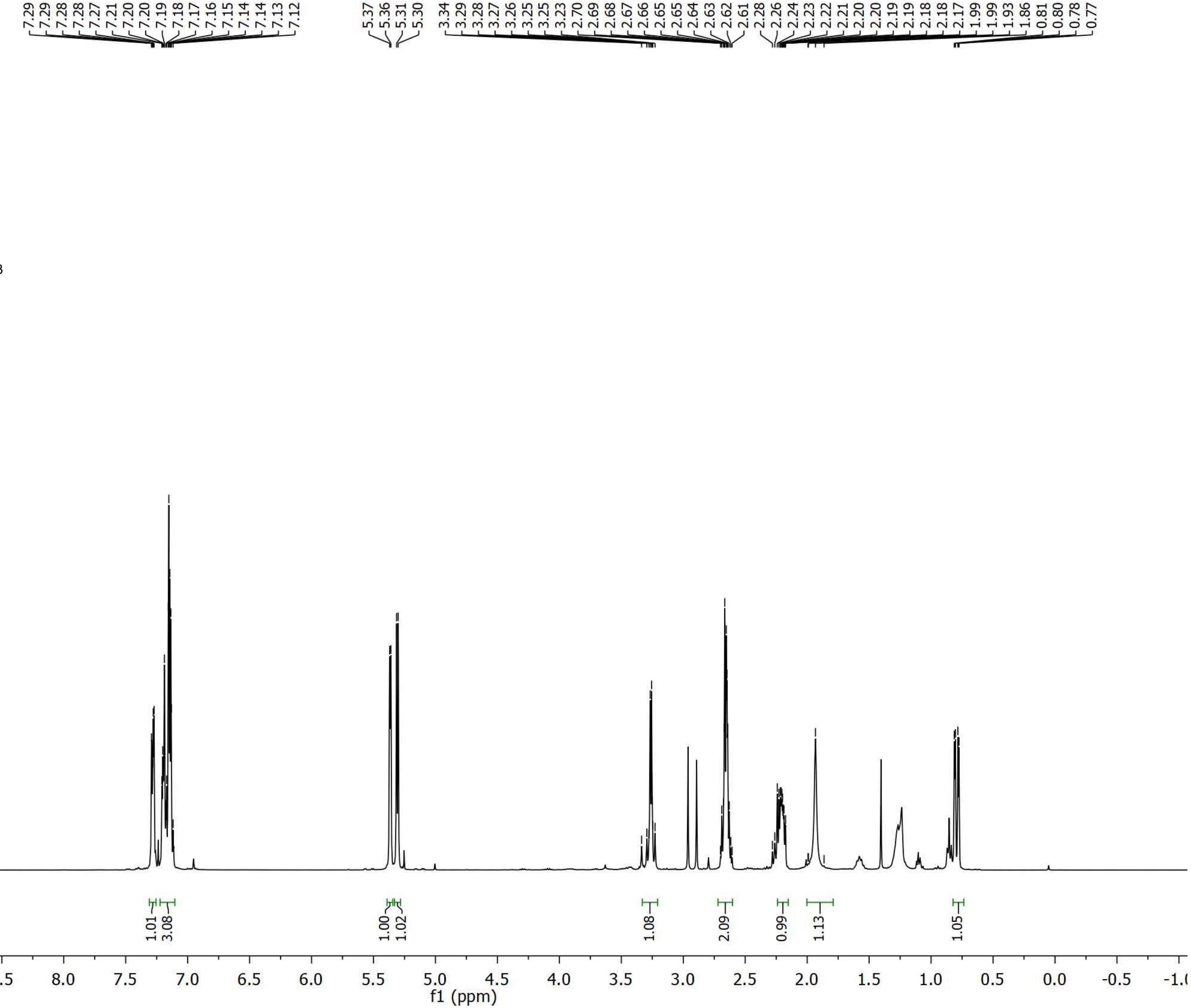

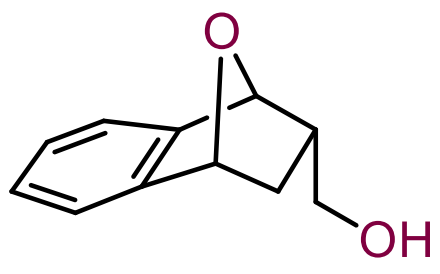

S3

^{13}C NMR, 101 MHz, $CDCl_3$

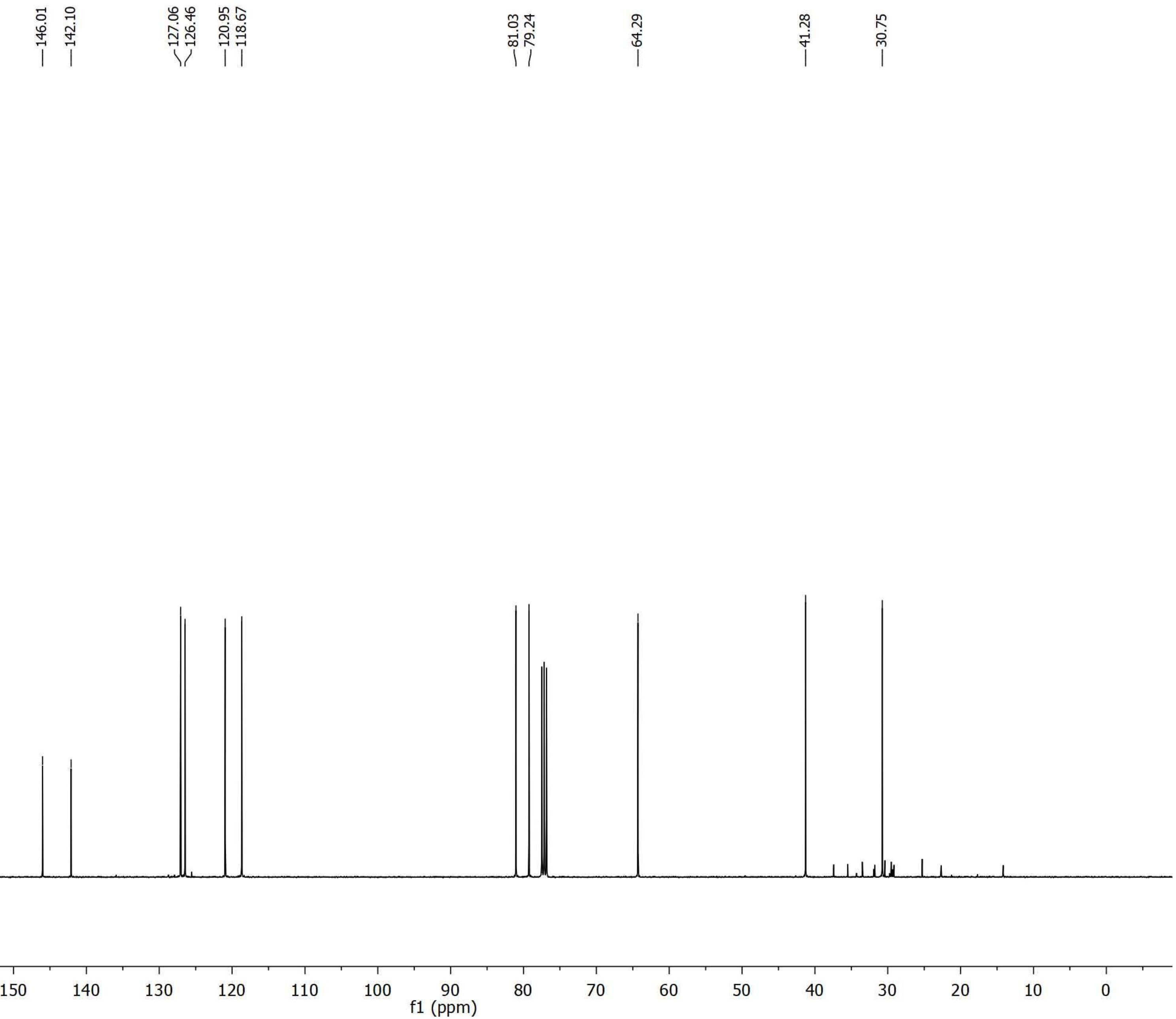

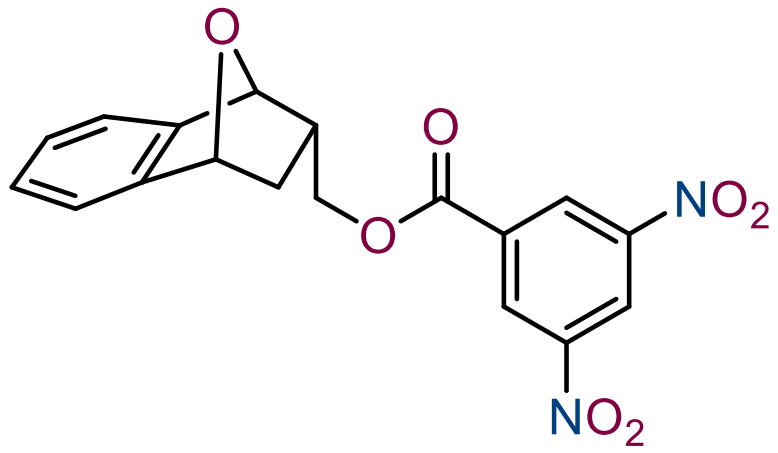

S4

^1H NMR, 400 MHz, $CDCl_3$

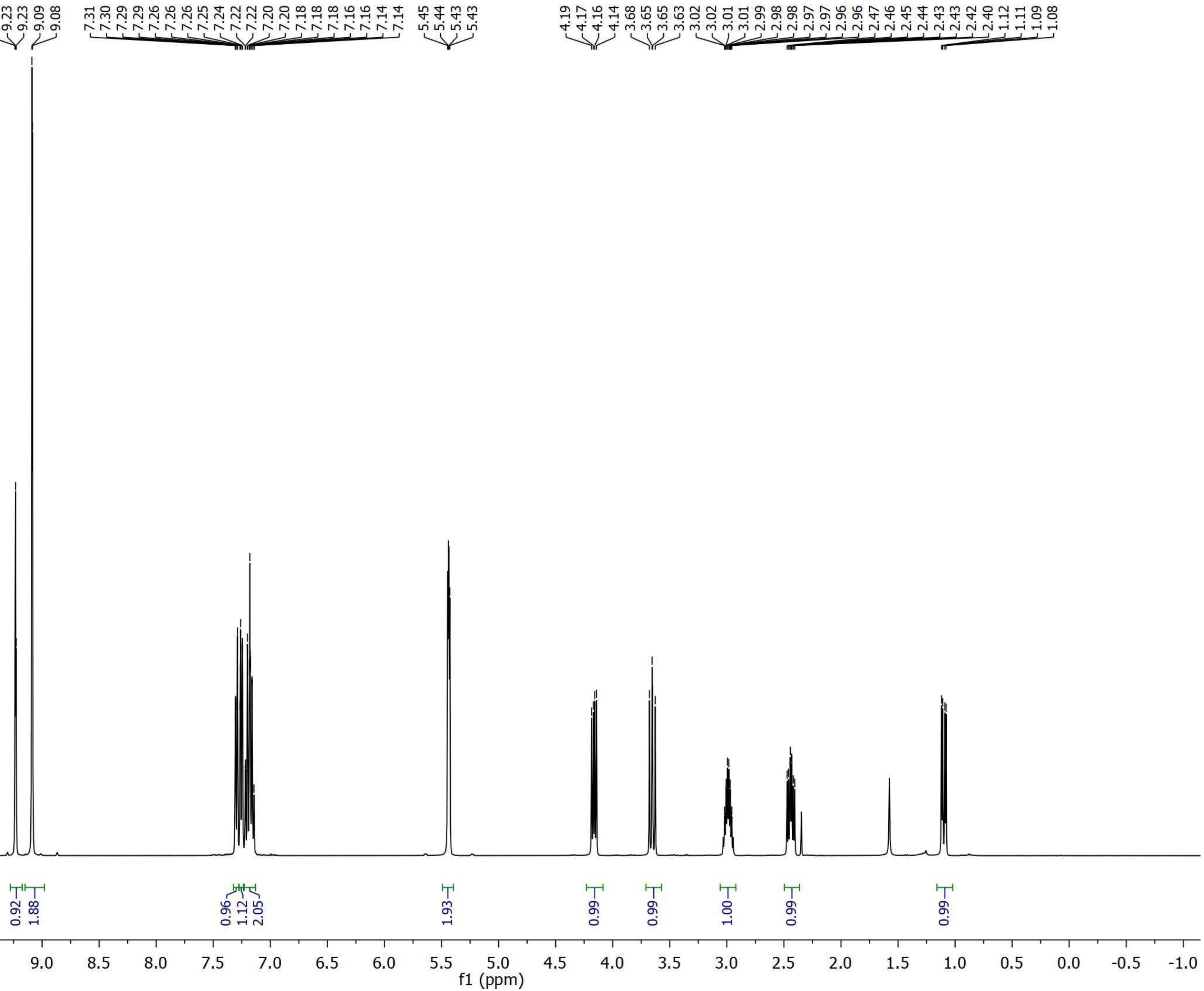

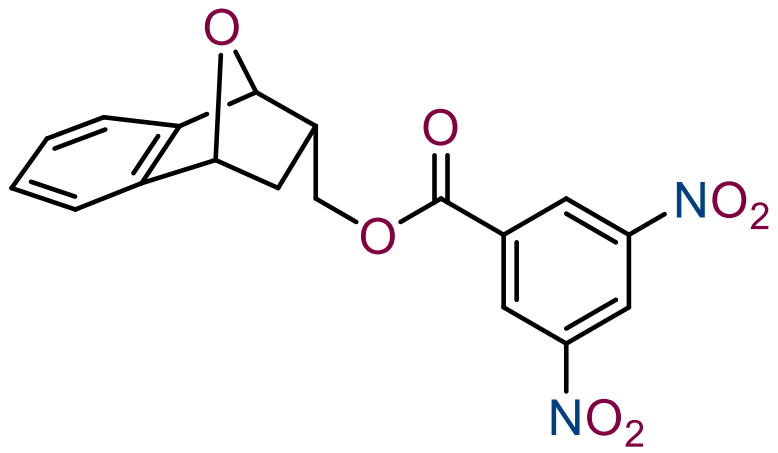

S4

^{13}C NMR, 101 MHz, $CDCl_3$

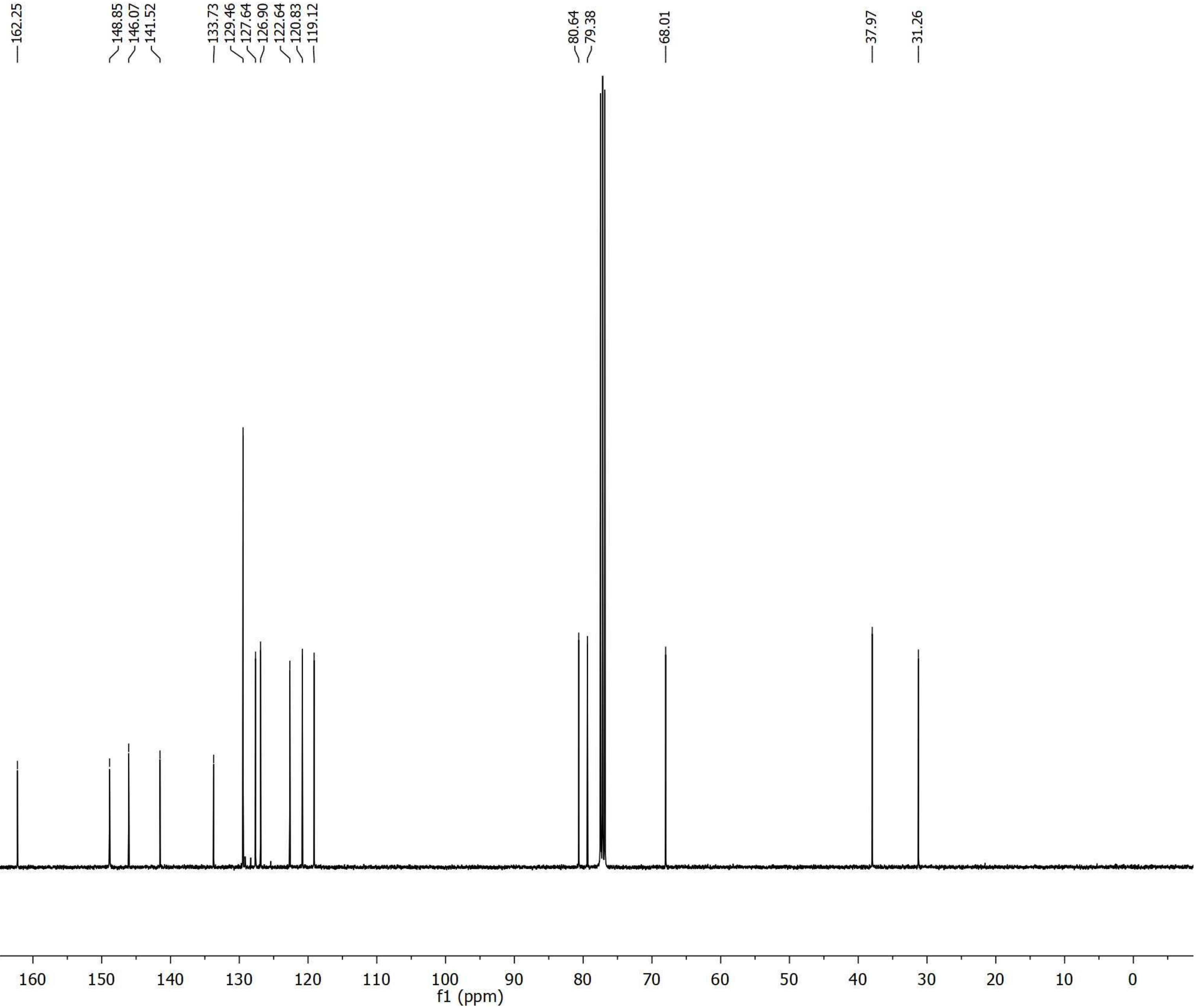

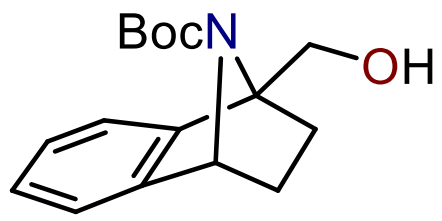

S5

^{1}H NMR, 400 MHz, $CDCl_3$

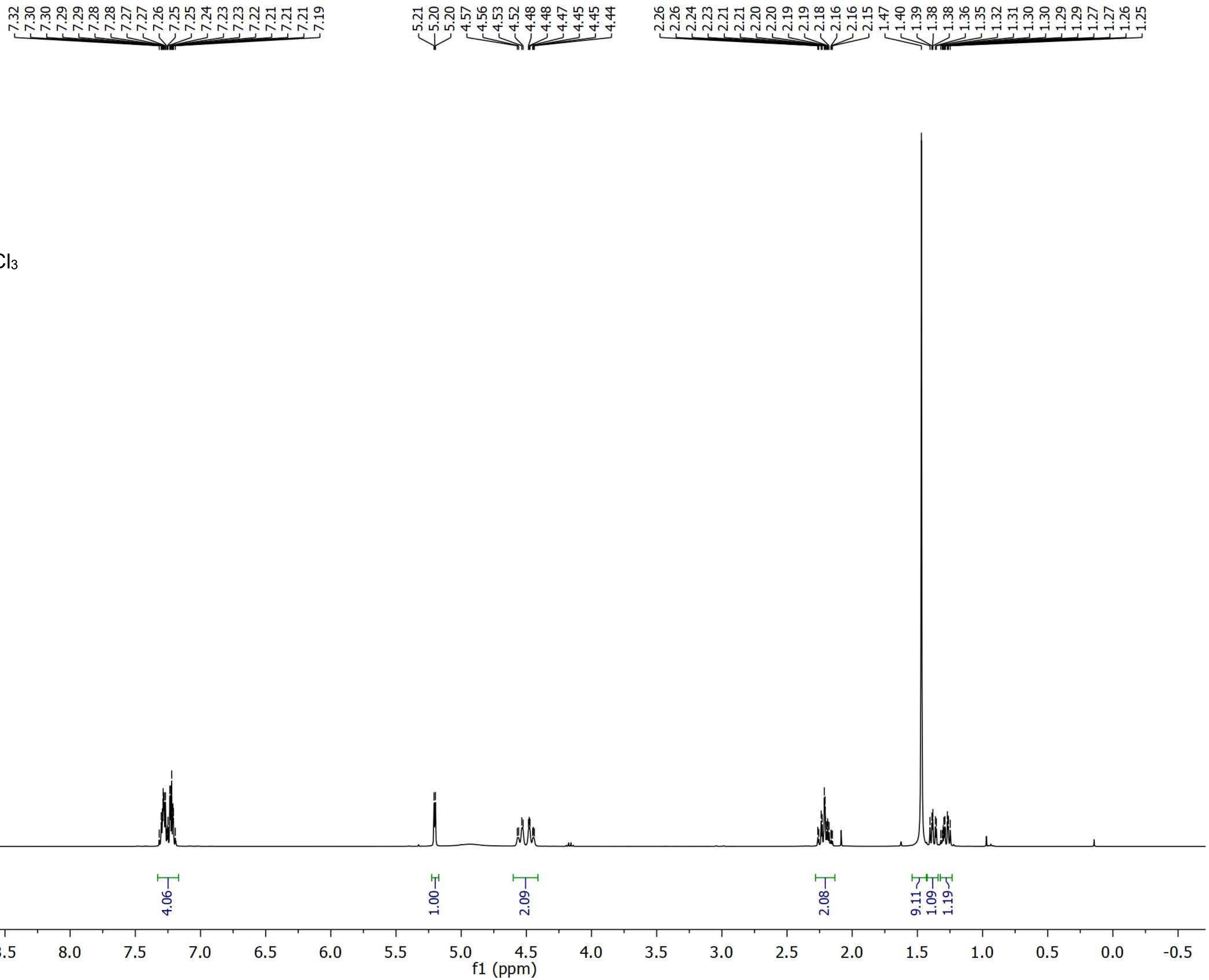

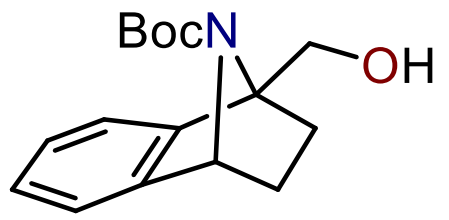

S5

^{13}C NMR, 101 MHz, $CDCl_3$

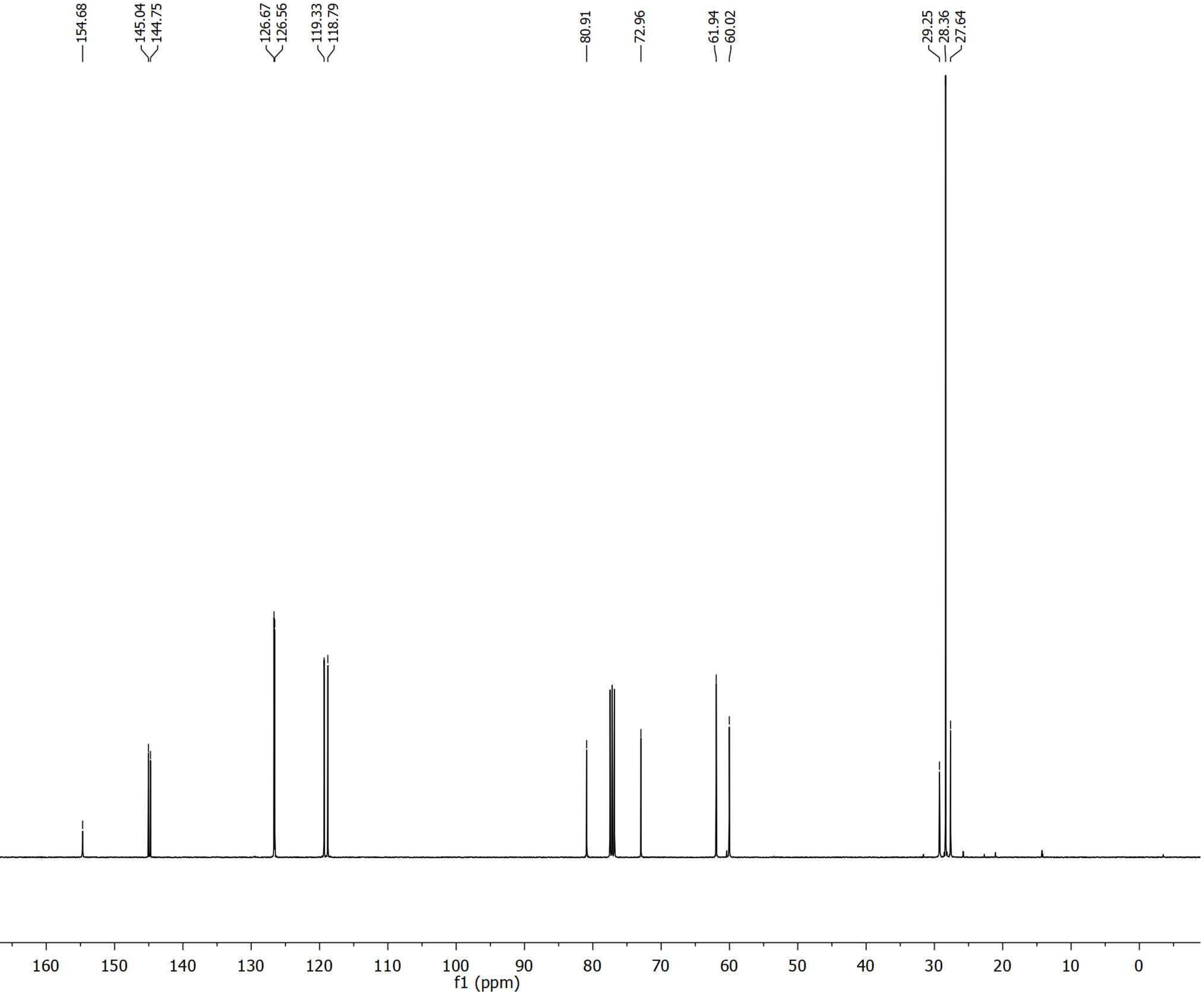

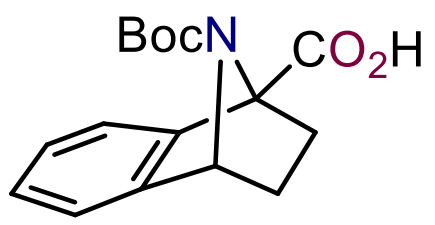

S6

^{1}H NMR, 400 MHz, $CDCl_3$

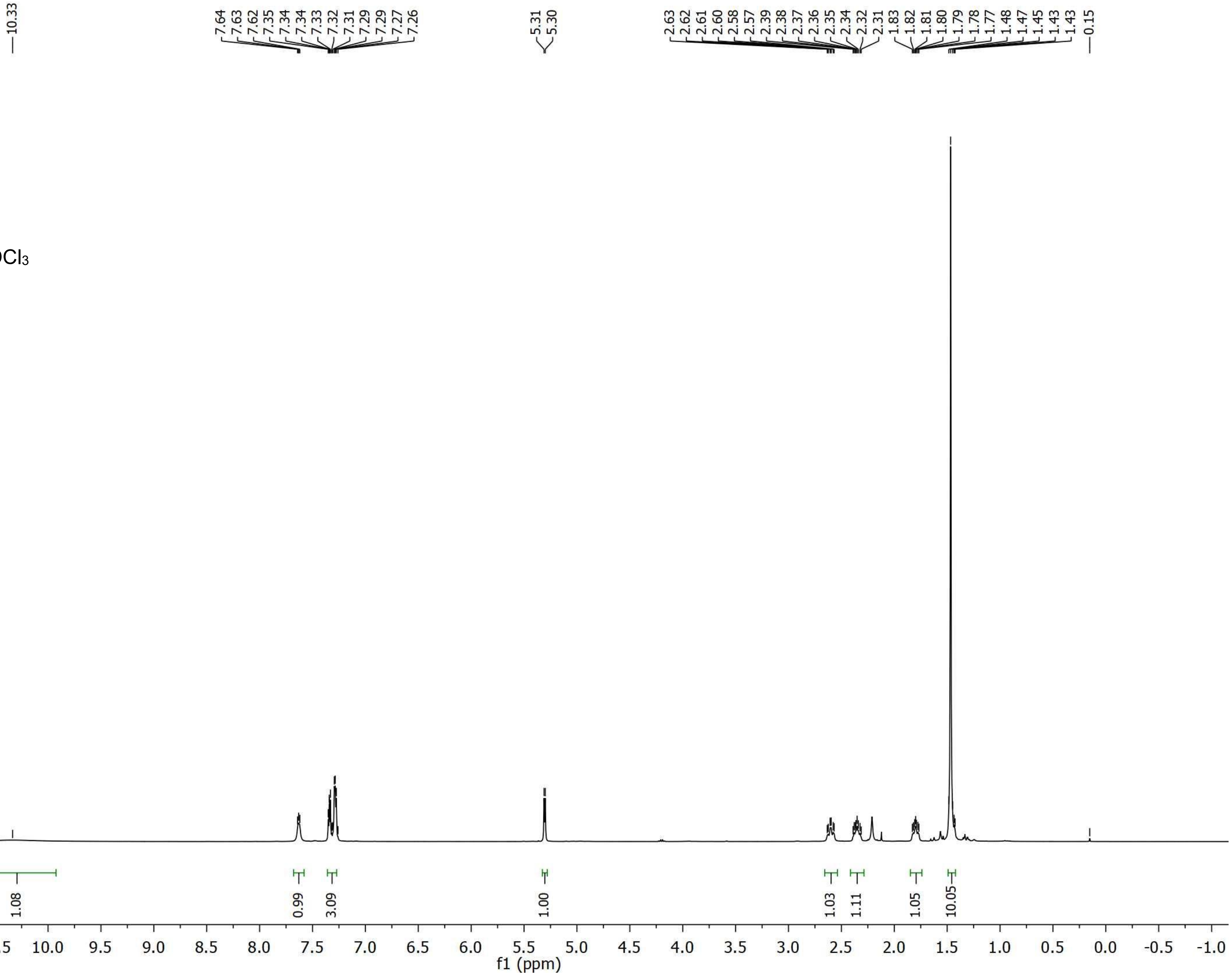

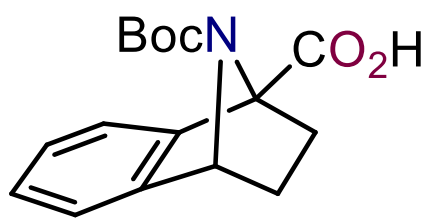

S6

^{13}C NMR, 101 MHz, $CDCl_3$

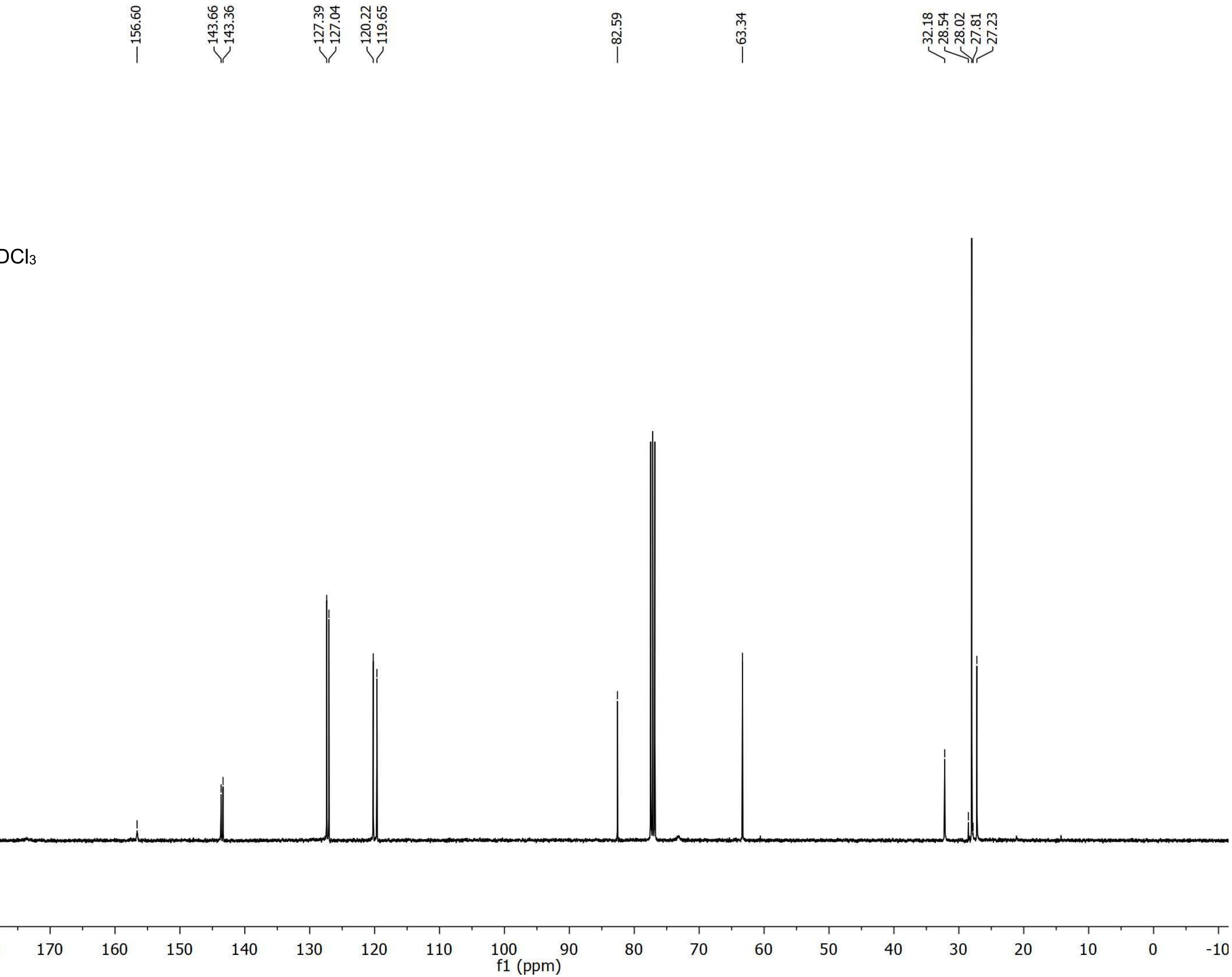

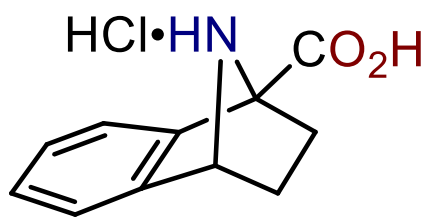

16

^{1}H NMR, 101 MHz, D_2O

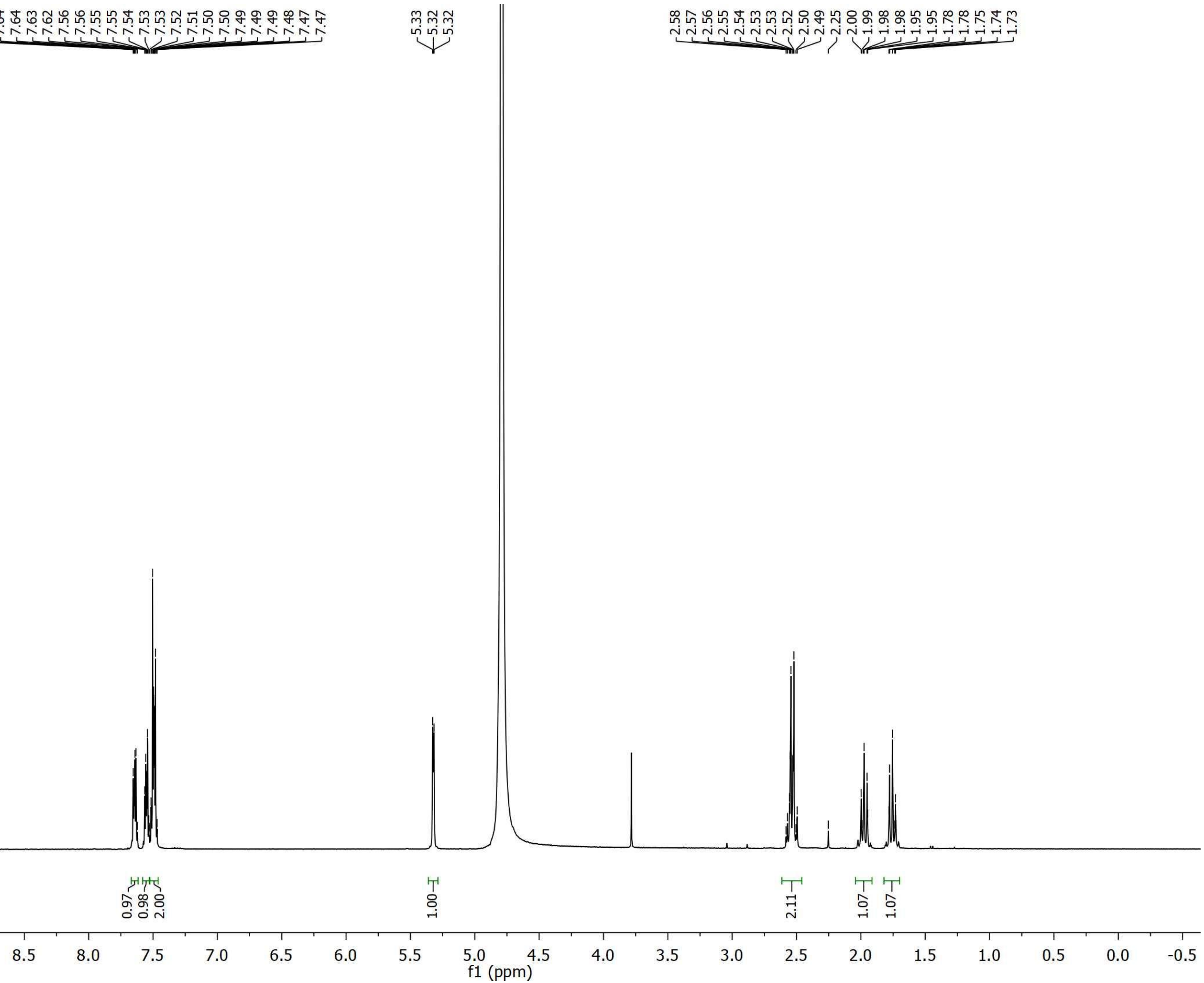

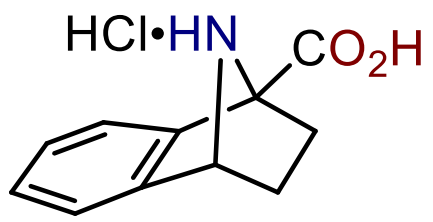

16

^{13}C NMR, 101 MHz, D_2O

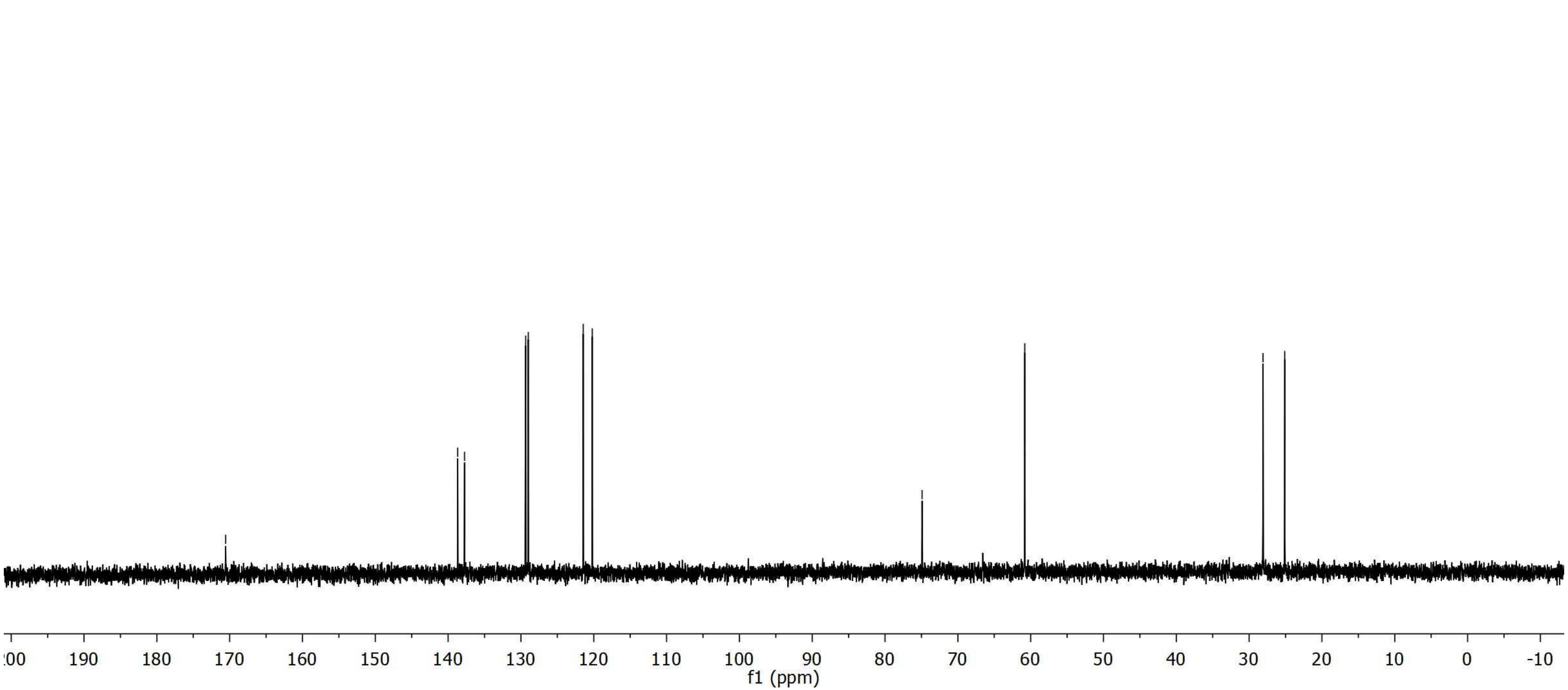

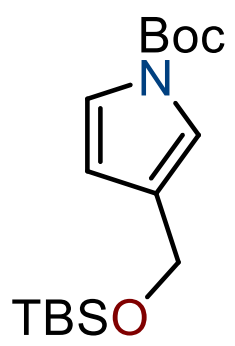

9

^{1}H NMR, 400 MHz, $CDCl_3$

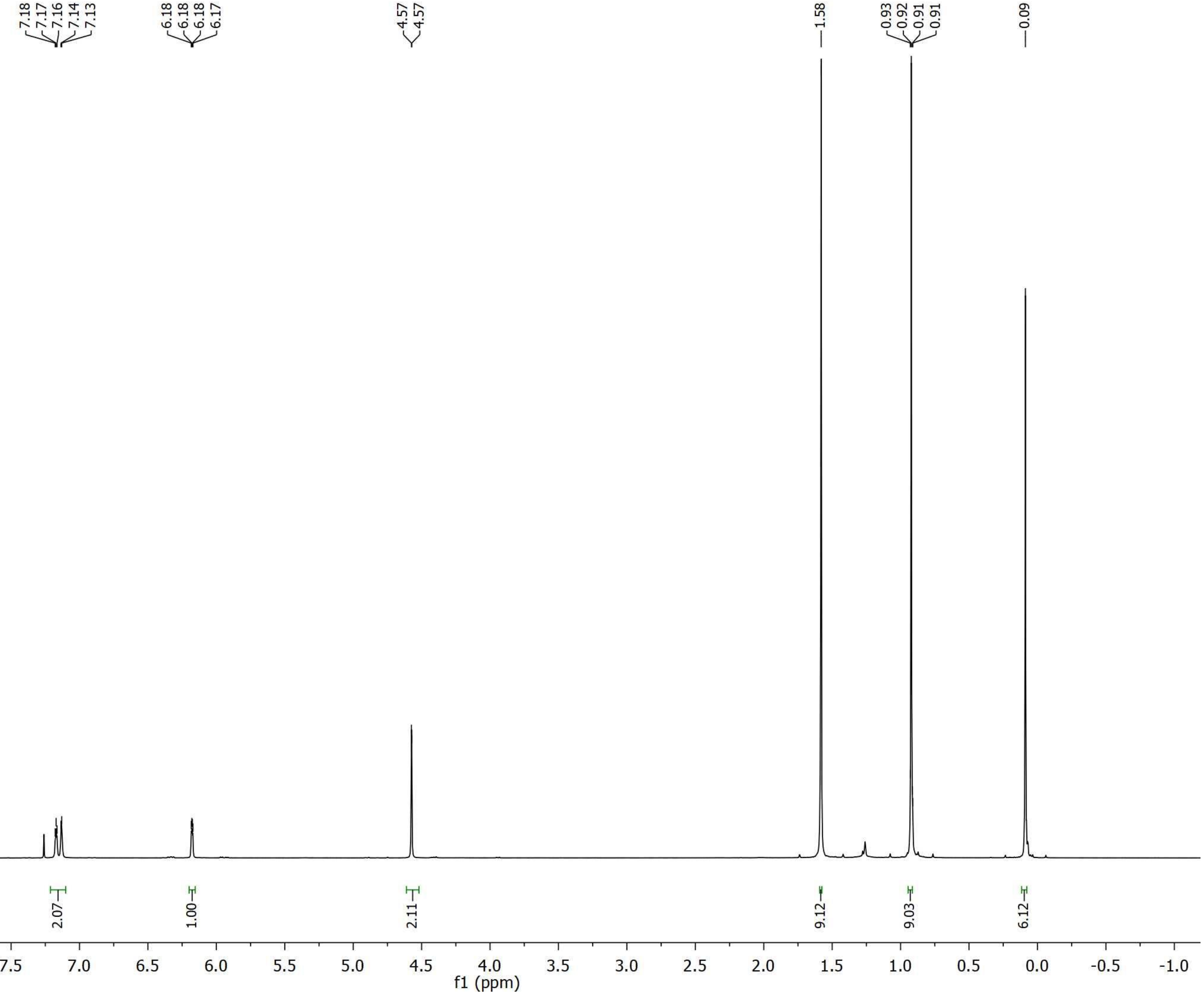

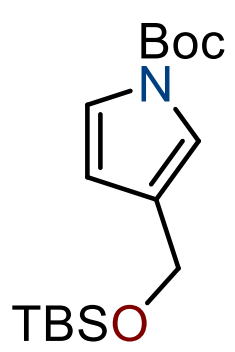

9

^{13}C NMR, 101 MHz, $CDCl_3$

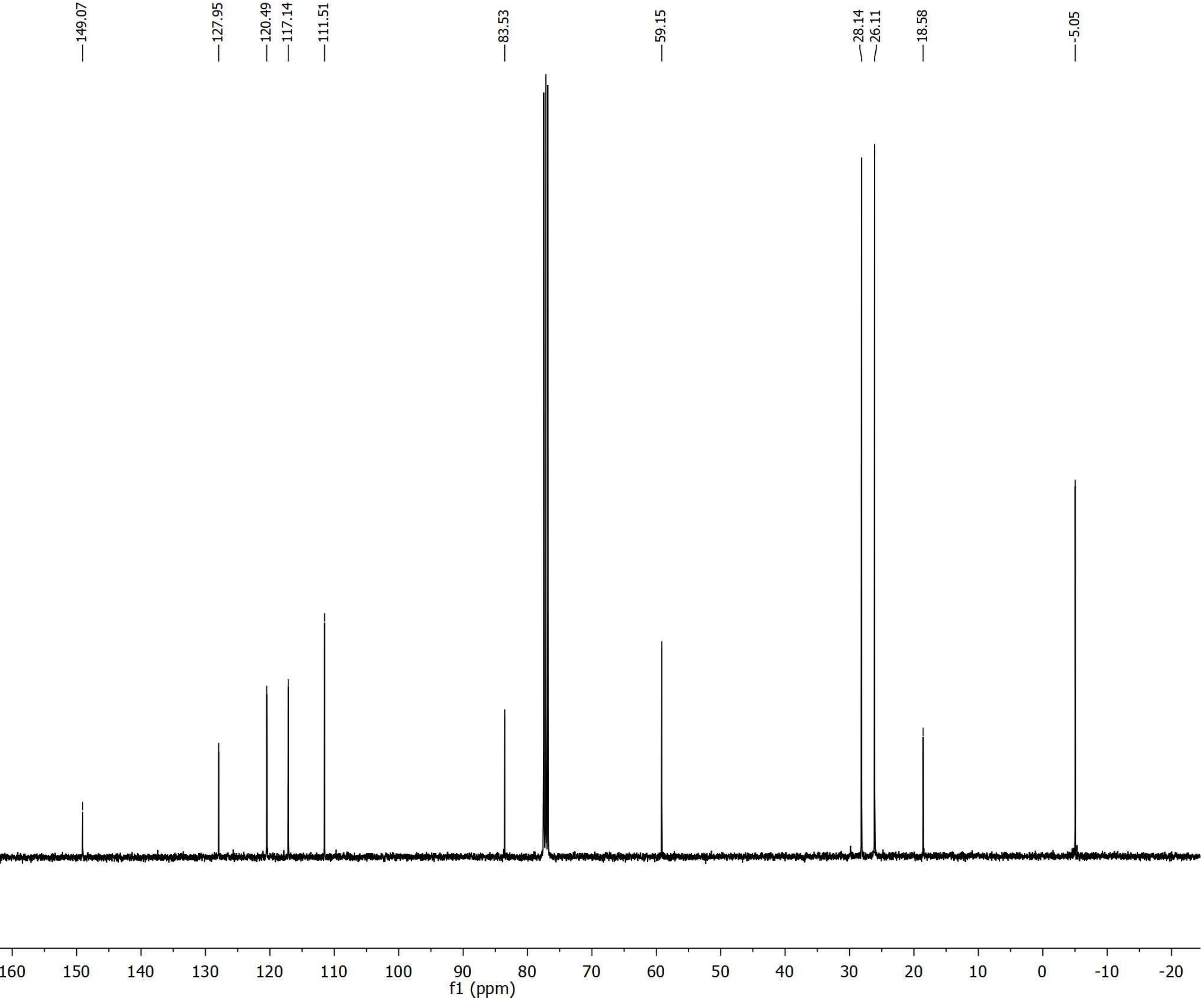

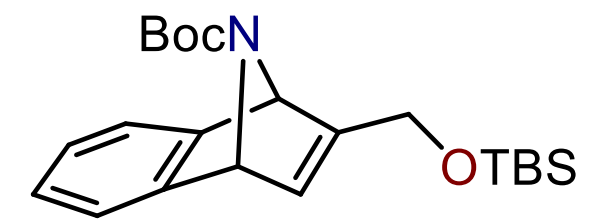

17

^{1}H NMR, 400 MHz, DMSO *d*6

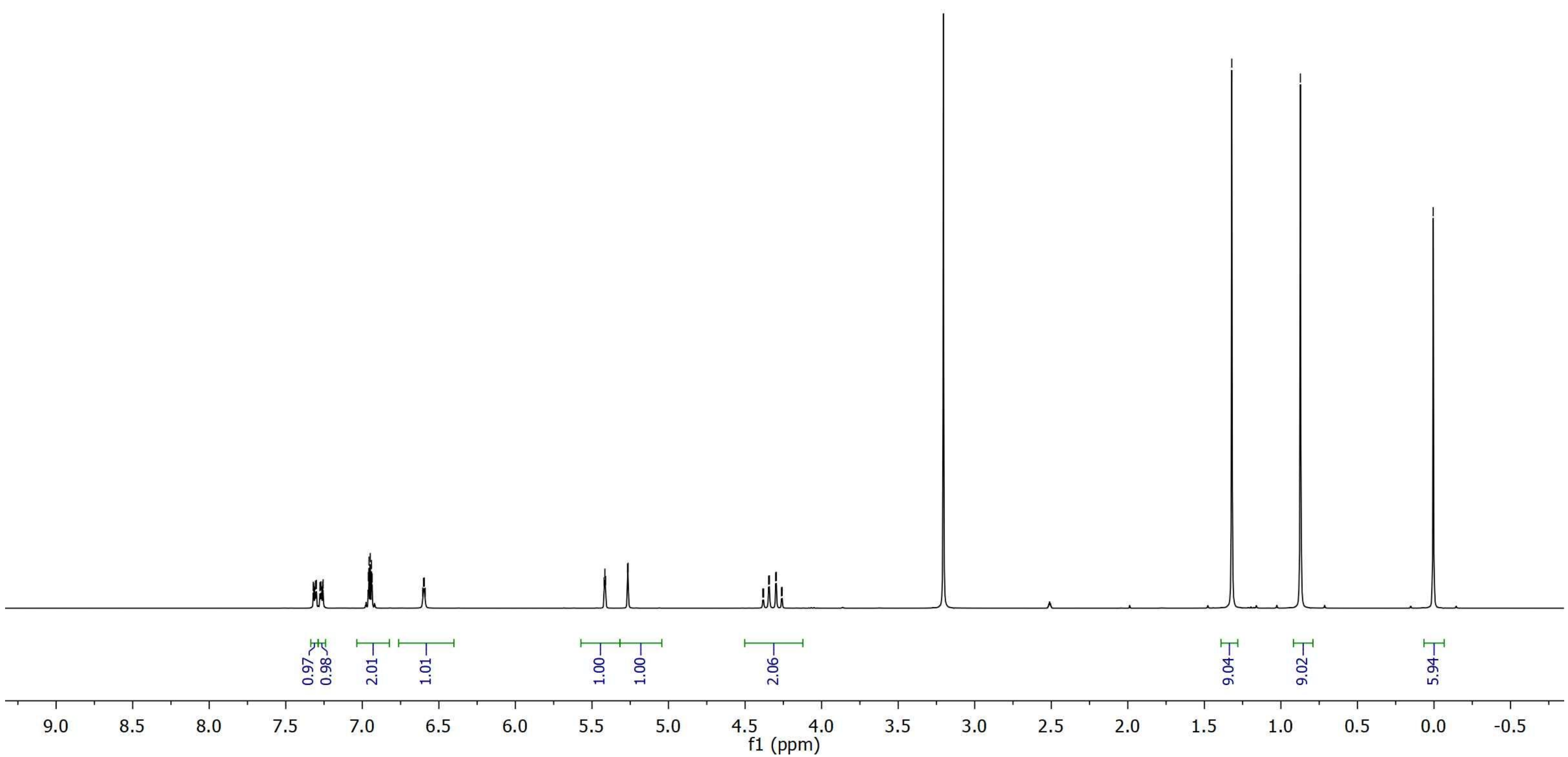

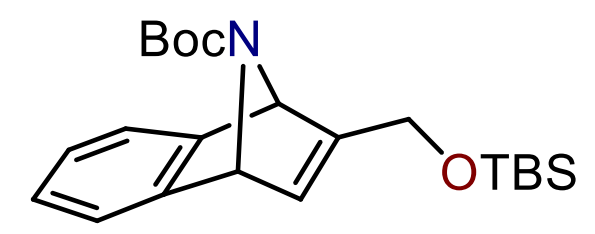

17

^{13}C NMR, 101 MHz, DMSO *d*6

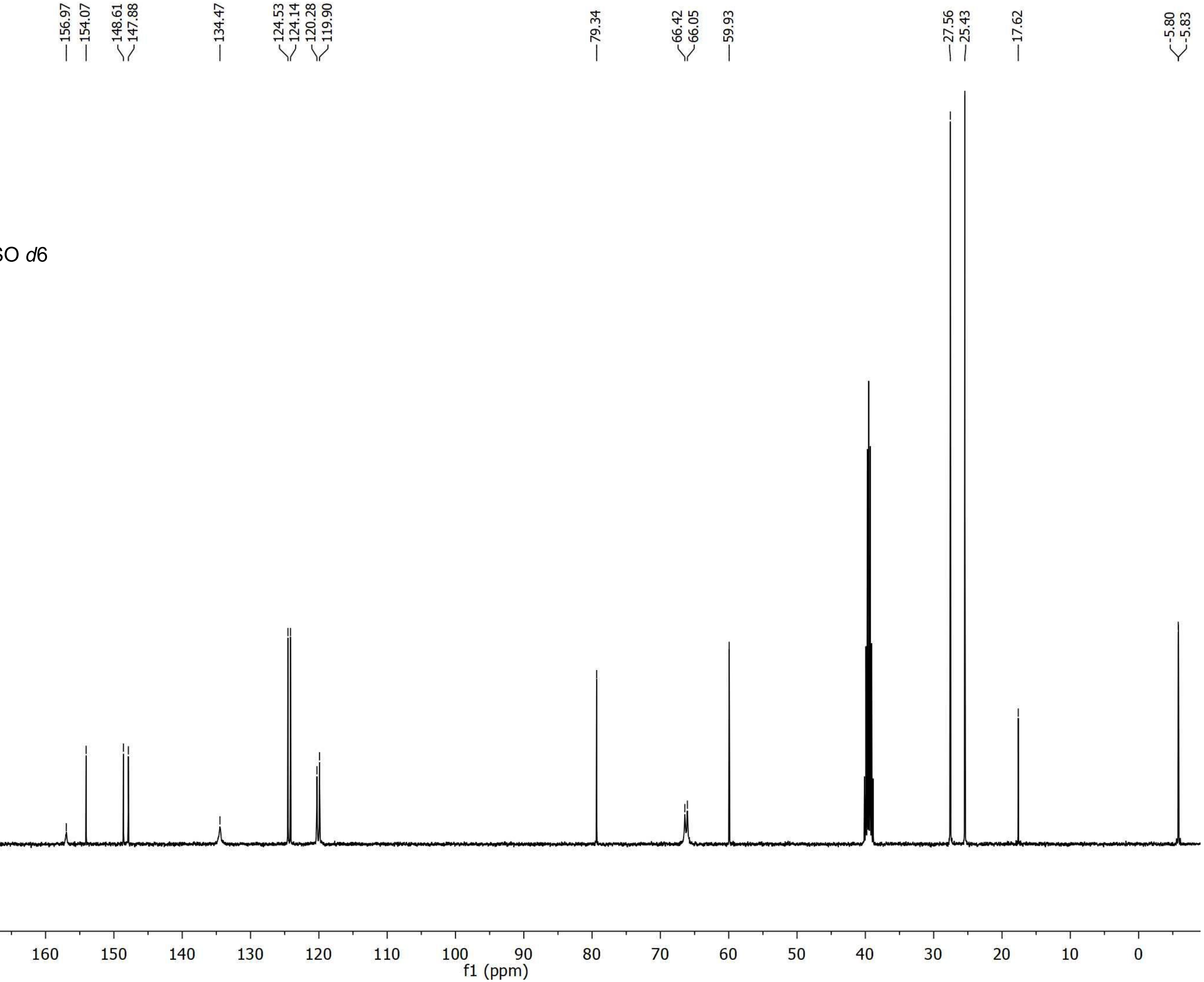

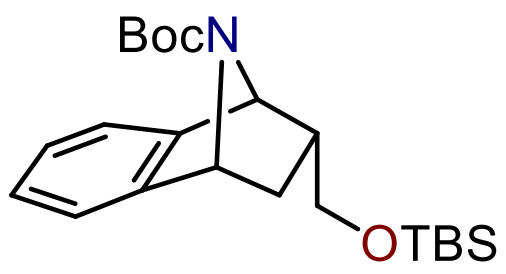

S8

^{1}H NMR, 400 MHz, $CDCl_3$

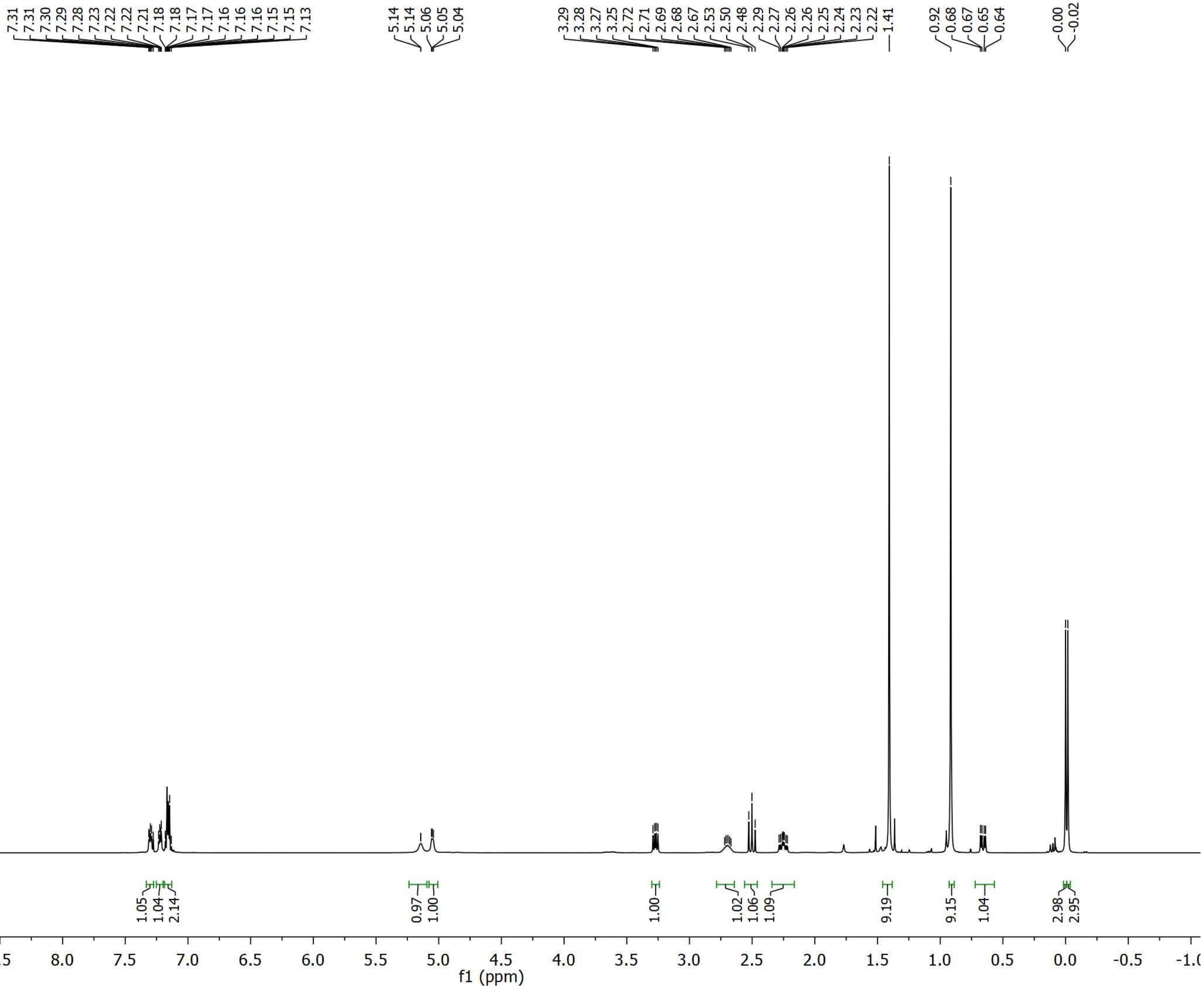

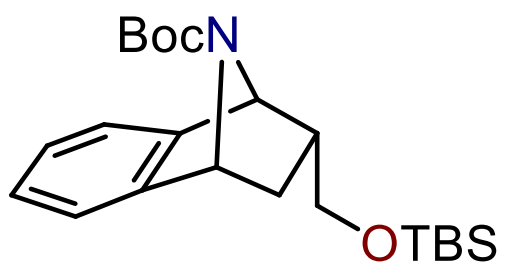

S8

^{13}C NMR, 101 MHz, $CDCl_3$

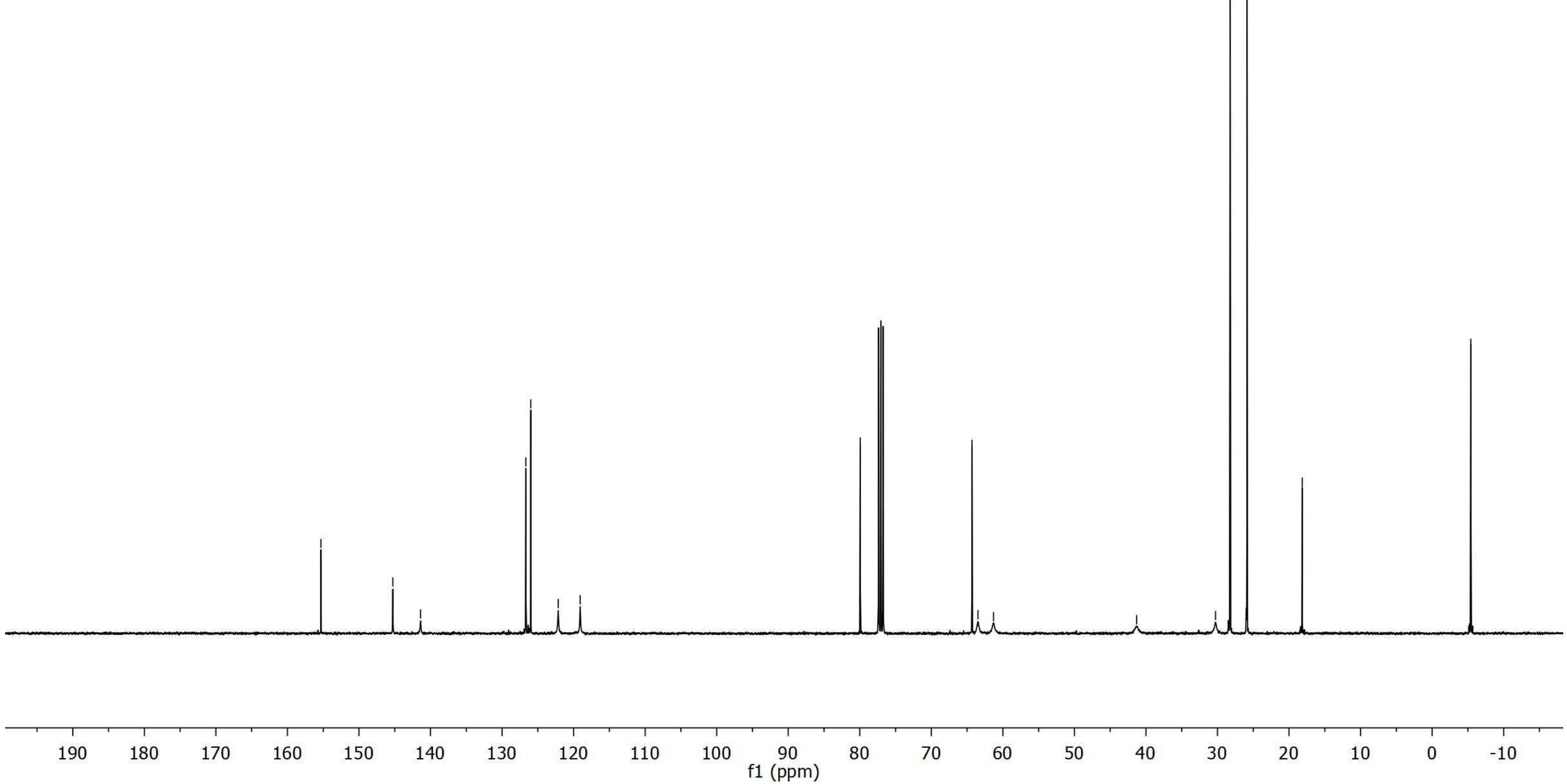

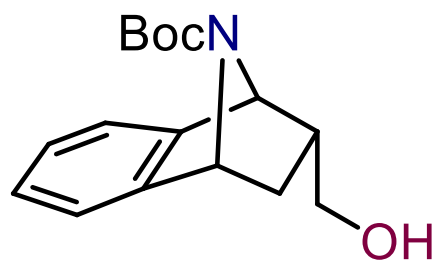

S9

^1H NMR, 400 MHz, $CDCl_3$

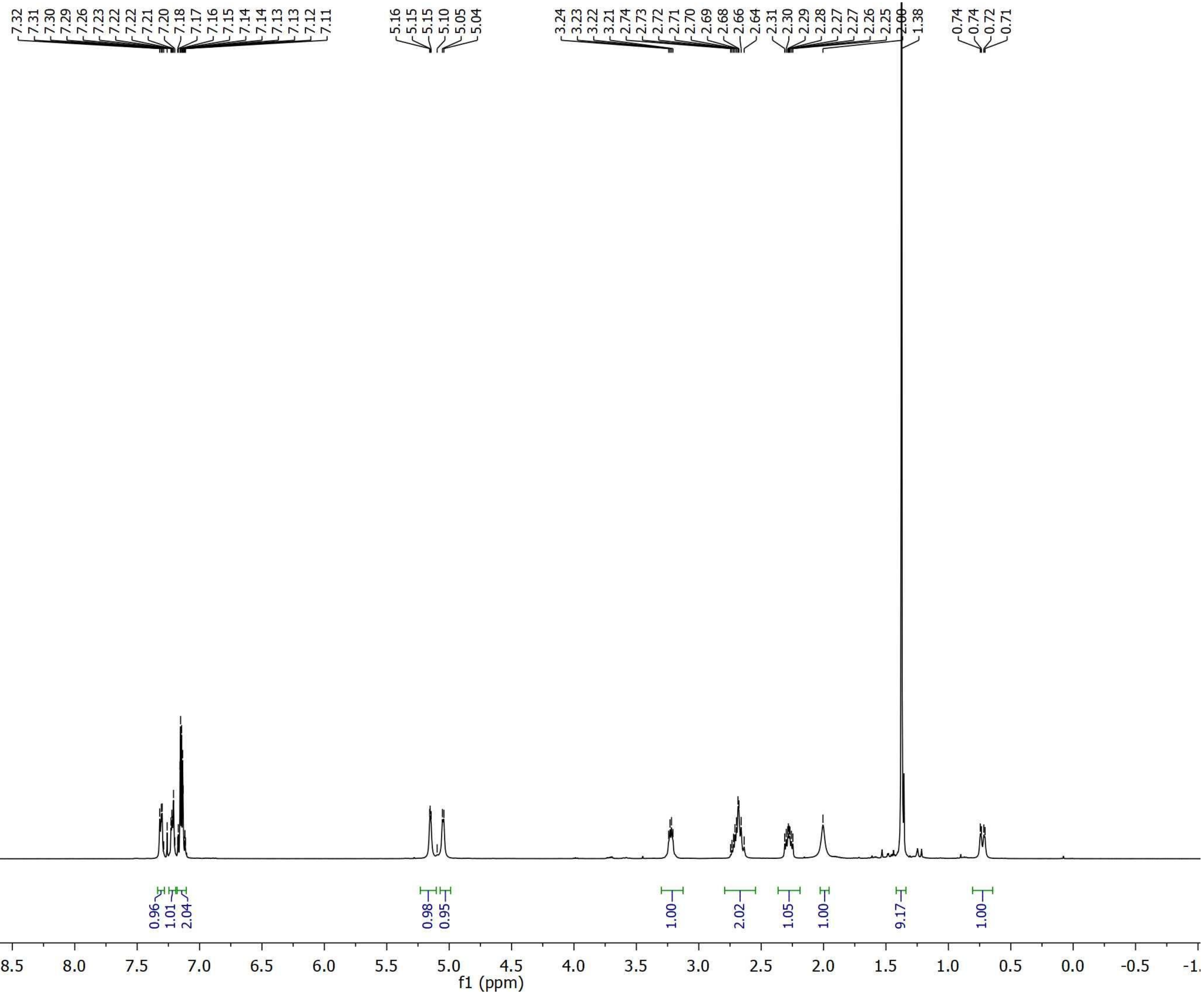

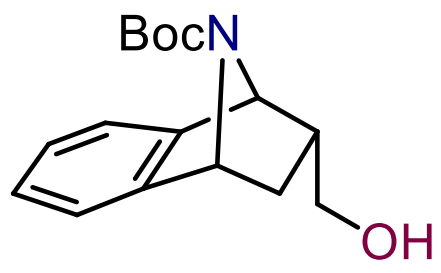

S9

^{13}C NMR, 101 MHz, $CDCl_3$

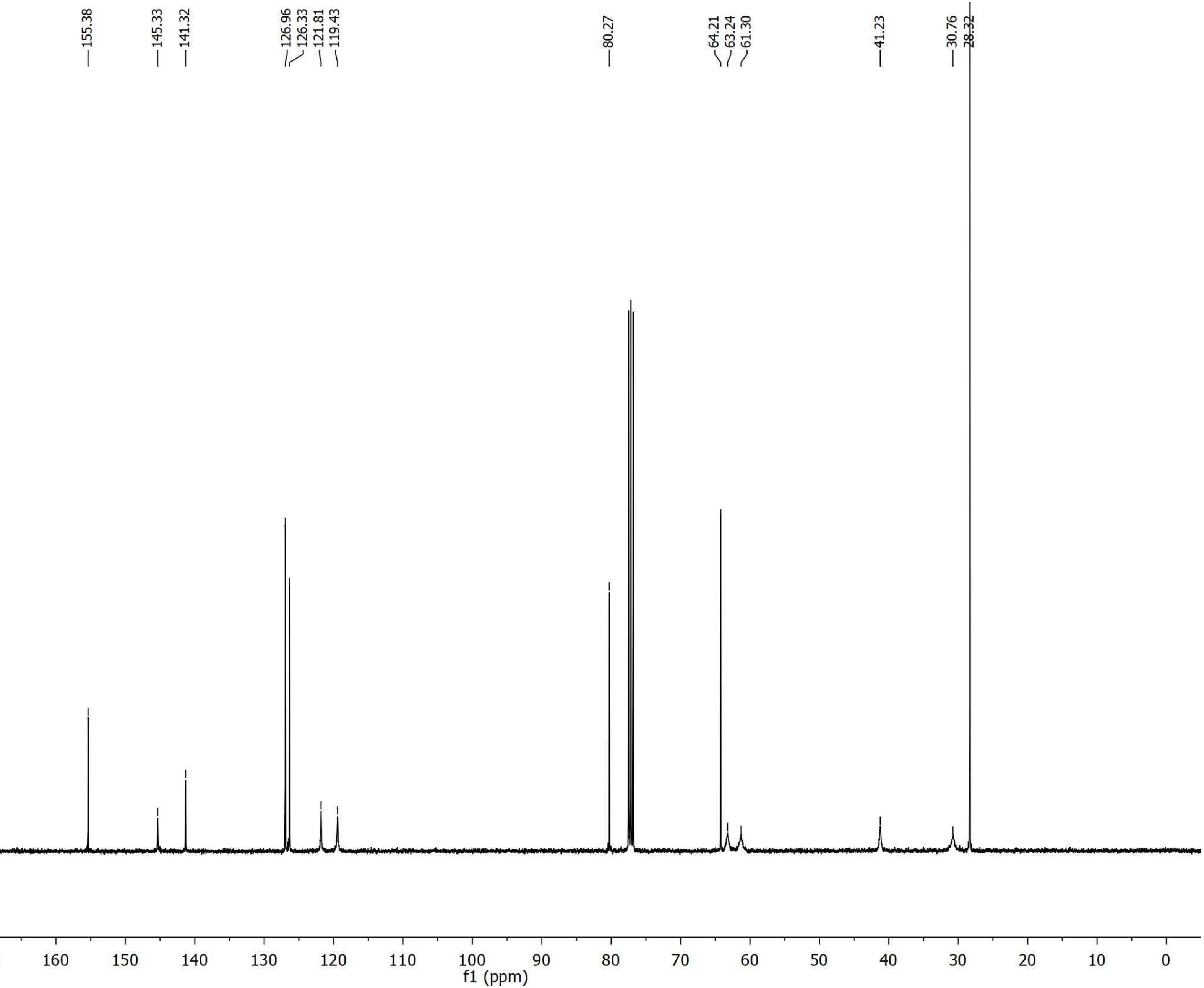

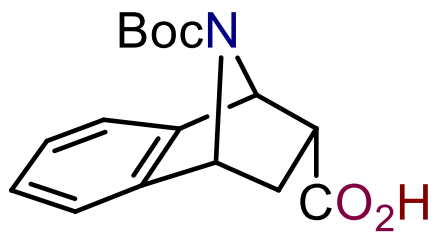

S10

^{1}H NMR, 400 MHz, $CDCl_3$

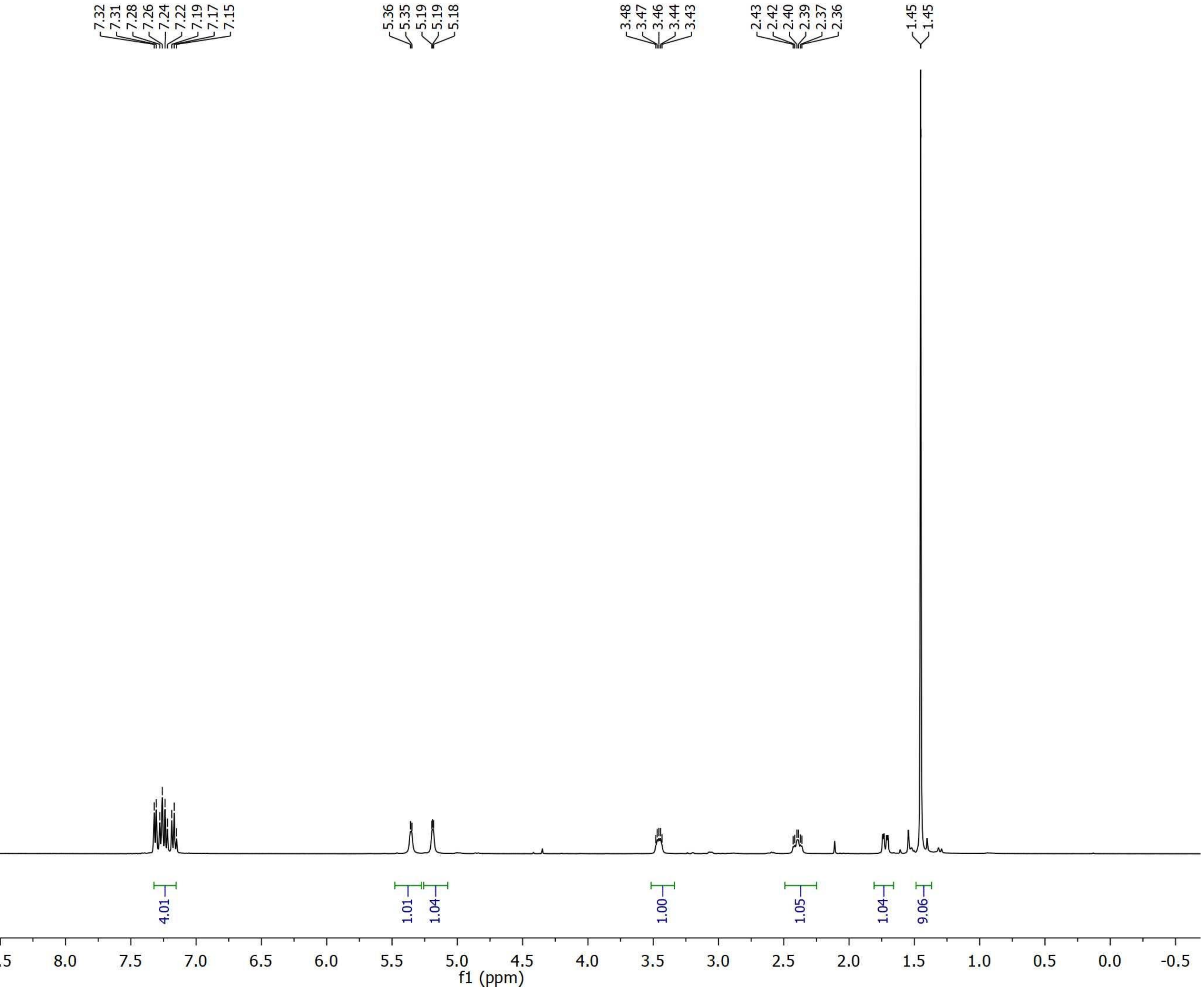

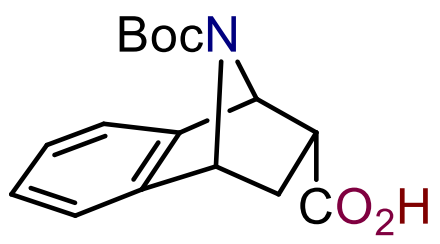

S10

^{13}C NMR, 101 MHz, $CDCl_3$

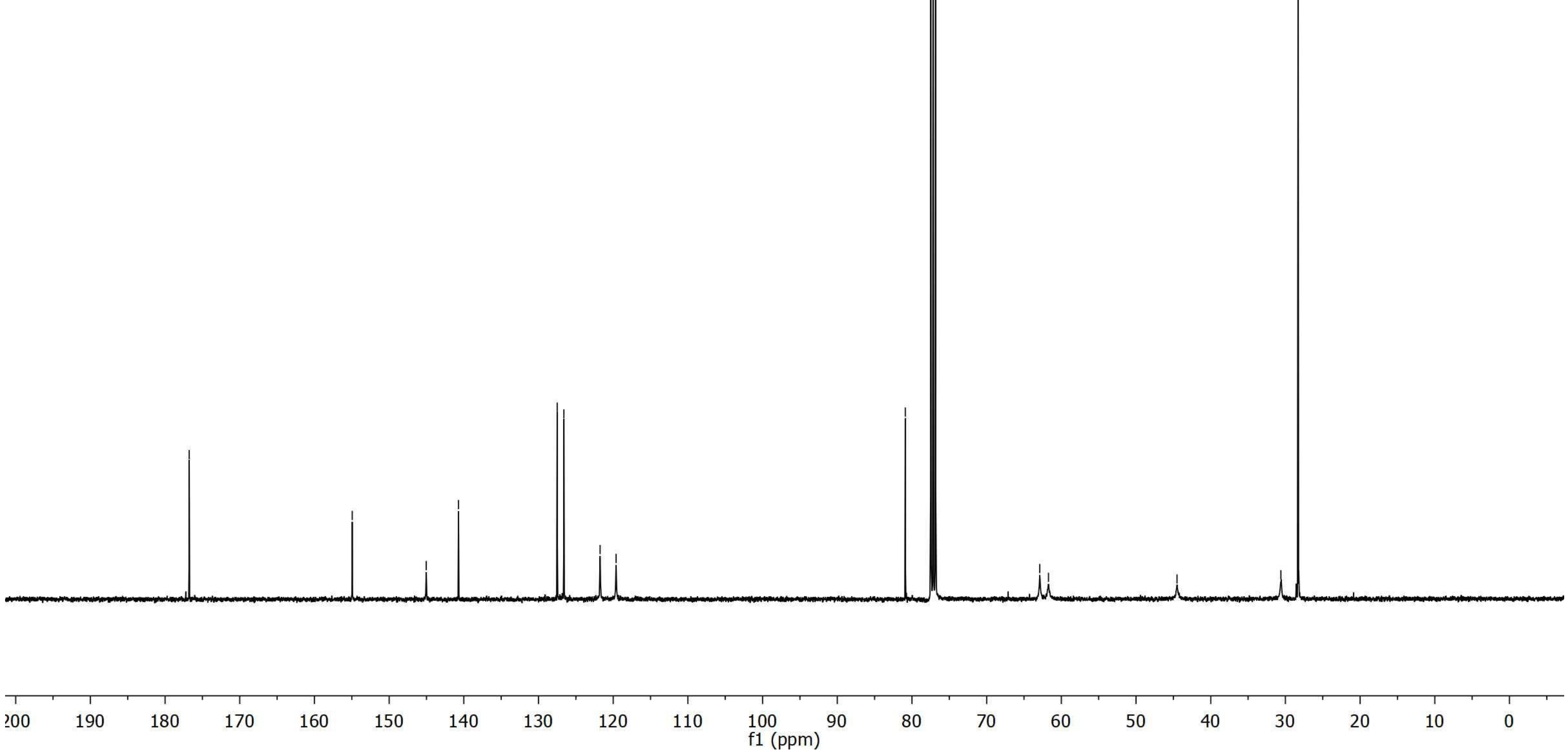

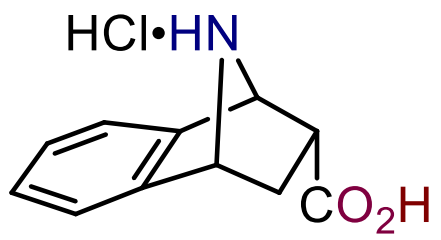

18

^1H NMR, 400 MHz, D_2O

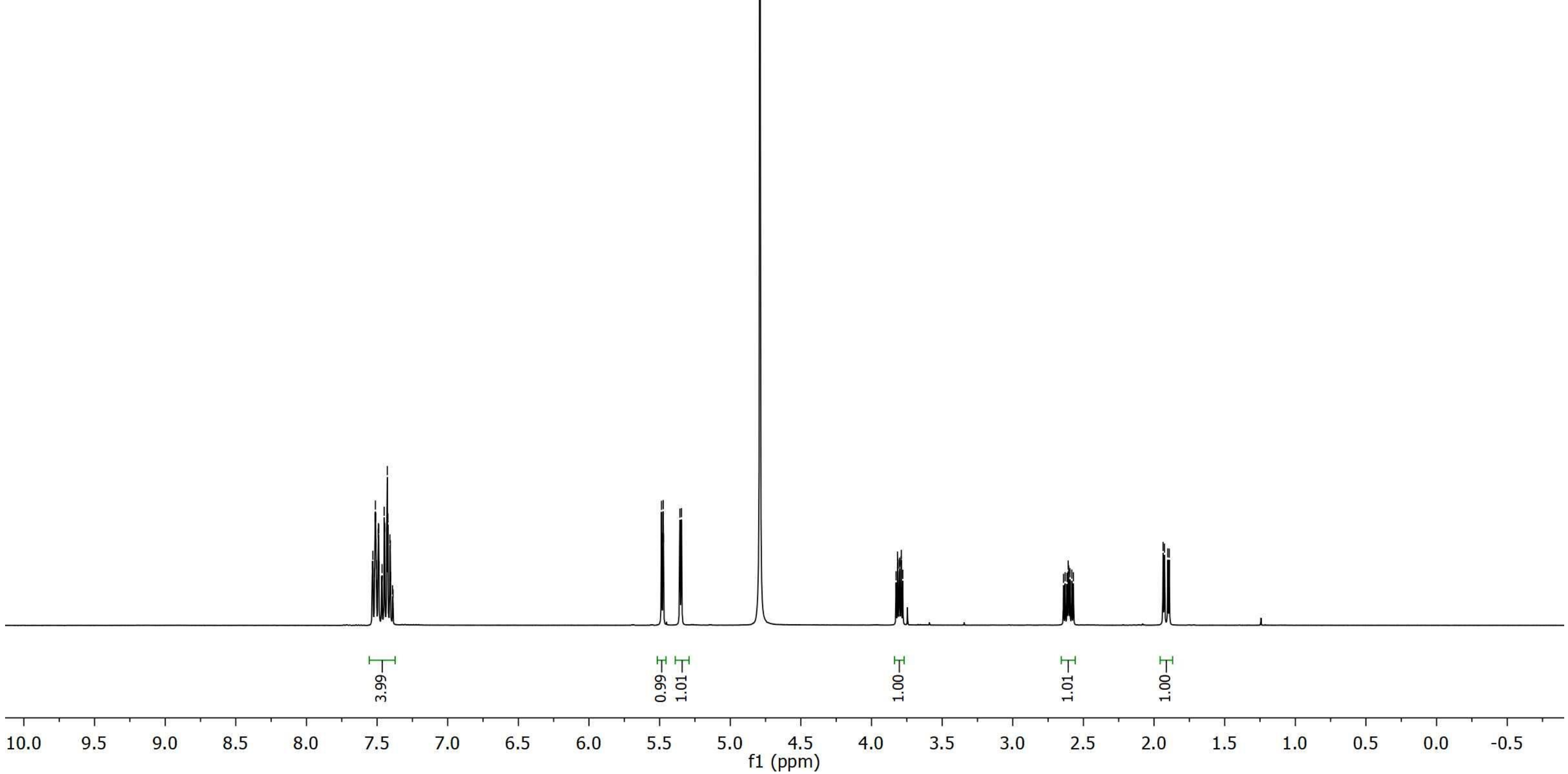

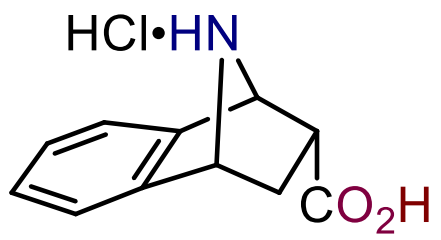

18

^{13}C NMR, 101 MHz, D_2O

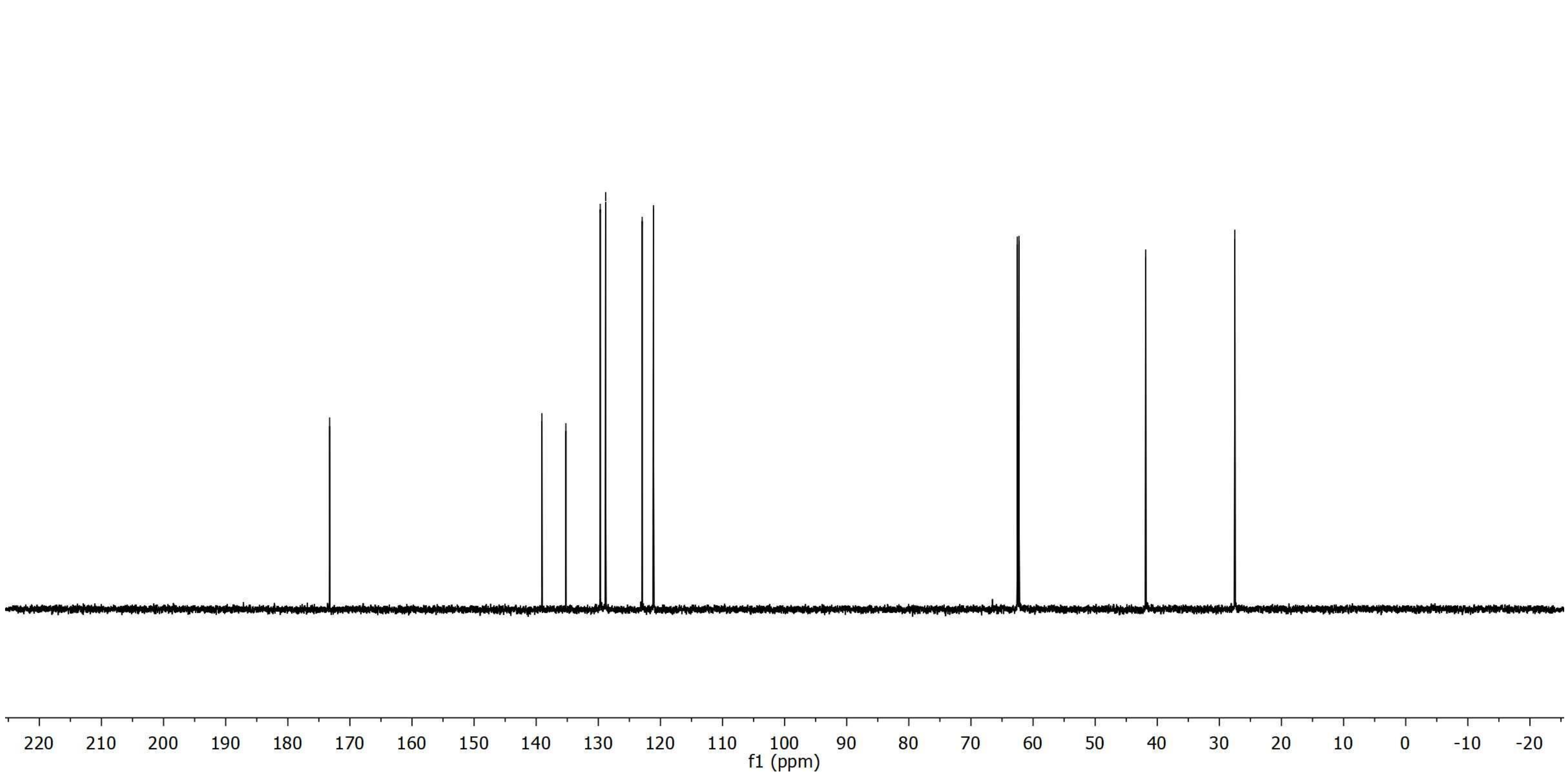

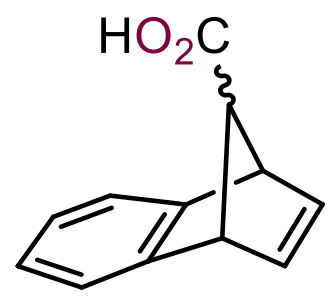

S12

^{1}H NMR, 400 MHz, $CDCl_3$

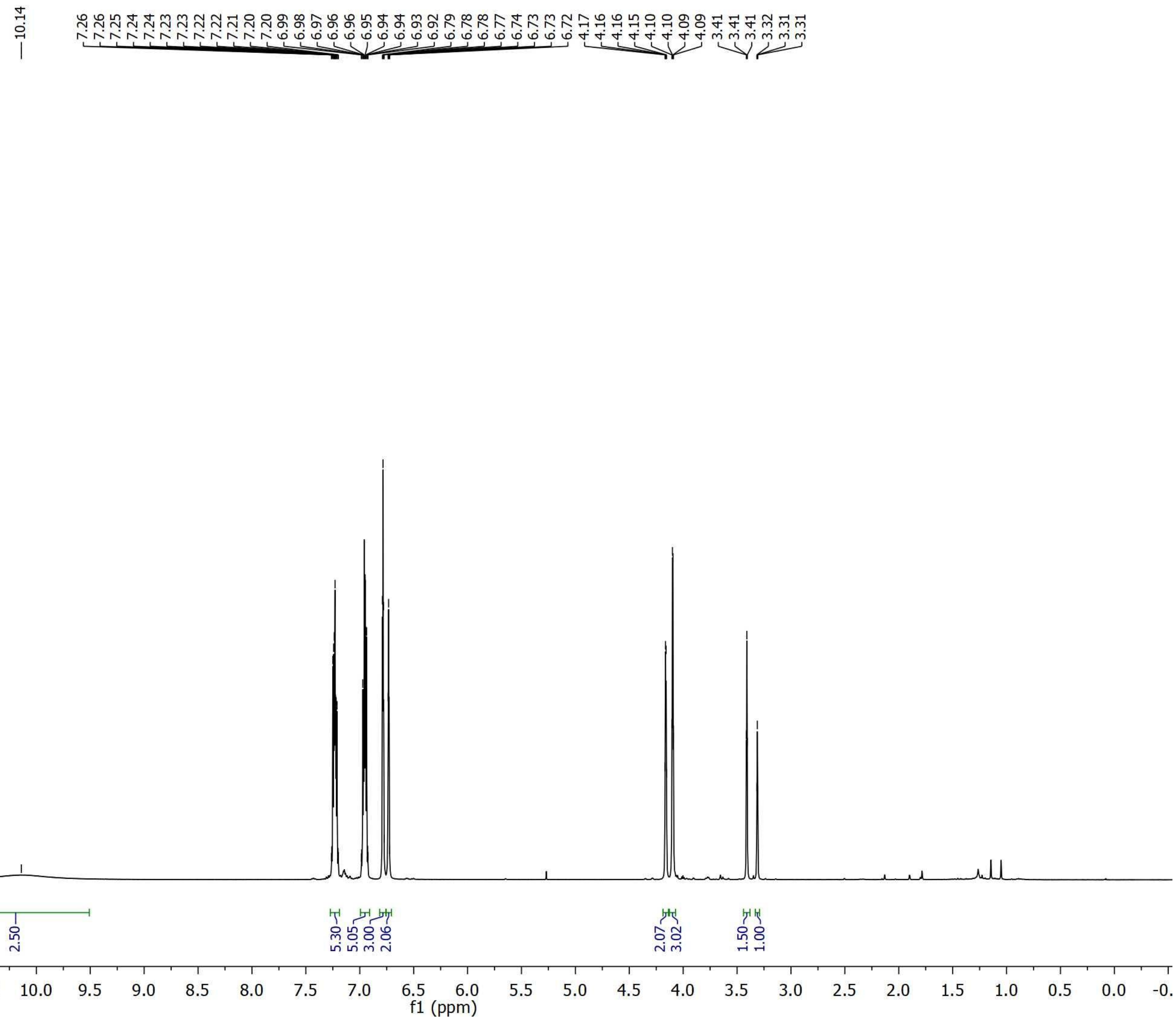

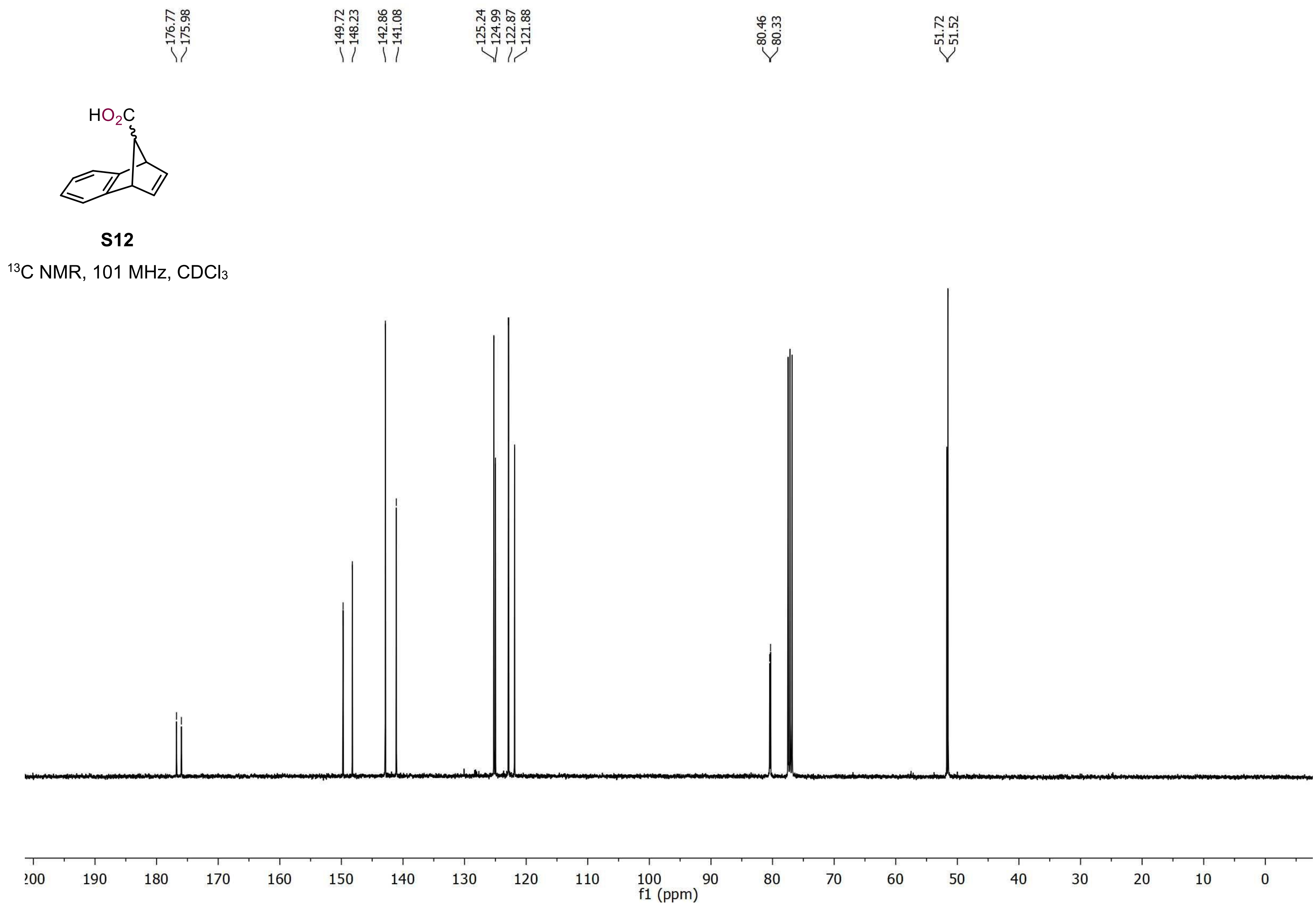
176.77
175.98
149.72
148.23
142.86
141.08
125.24
124.99
122.87
121.88
80.46
80.33
51.72
51.52
HO2C
S12
13C NMR, 101 MHz, CDCl3
200
190
180
170
160
150
140
130
120
110
100
90
80
70
60
50
40
30
20
10
0
f1 (ppm)

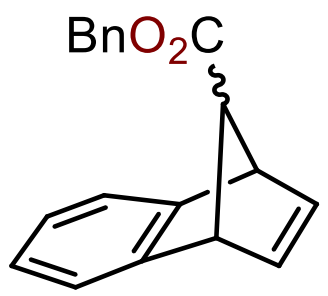

S13

^{1}H NMR, 400 MHz, $CDCl_3$

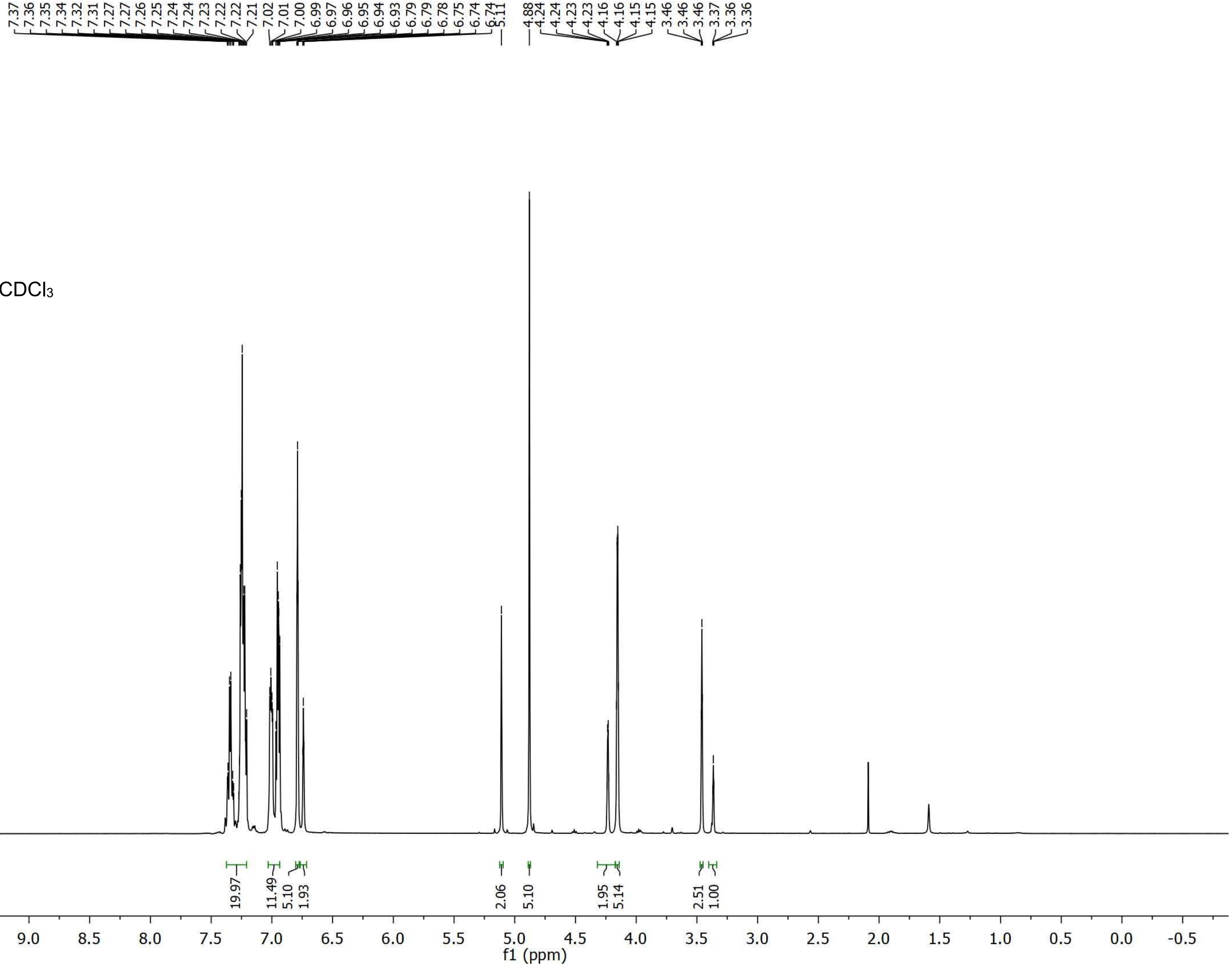

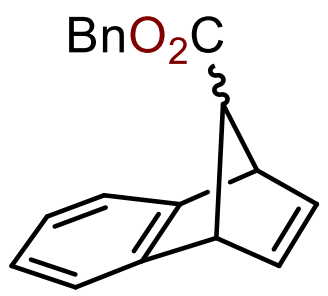

S13

^{13}C NMR, 101 MHz, $CDCl_3$

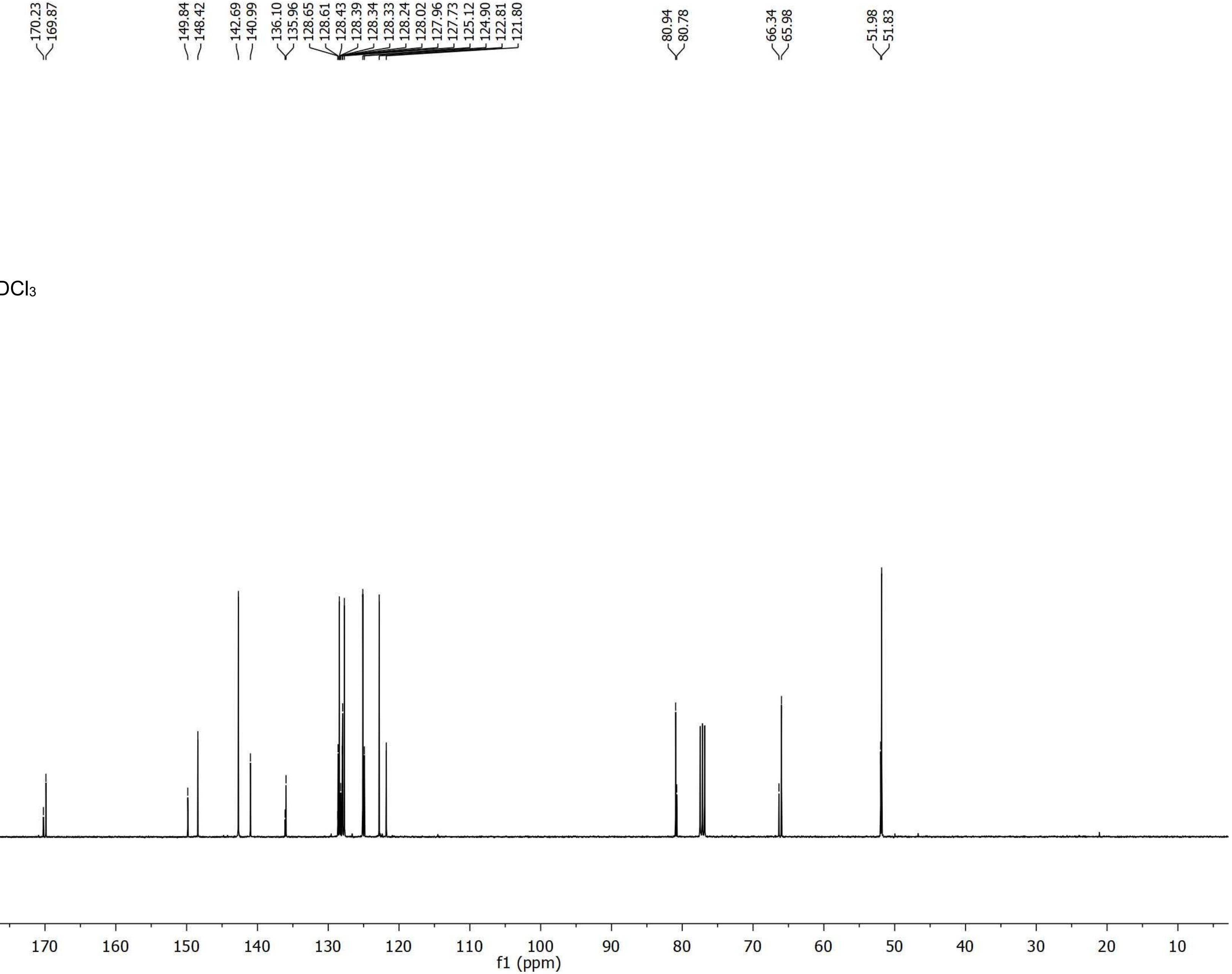

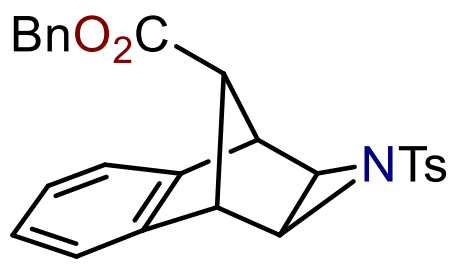

20

^{1}H NMR, 400 MHz, $CDCl_3$

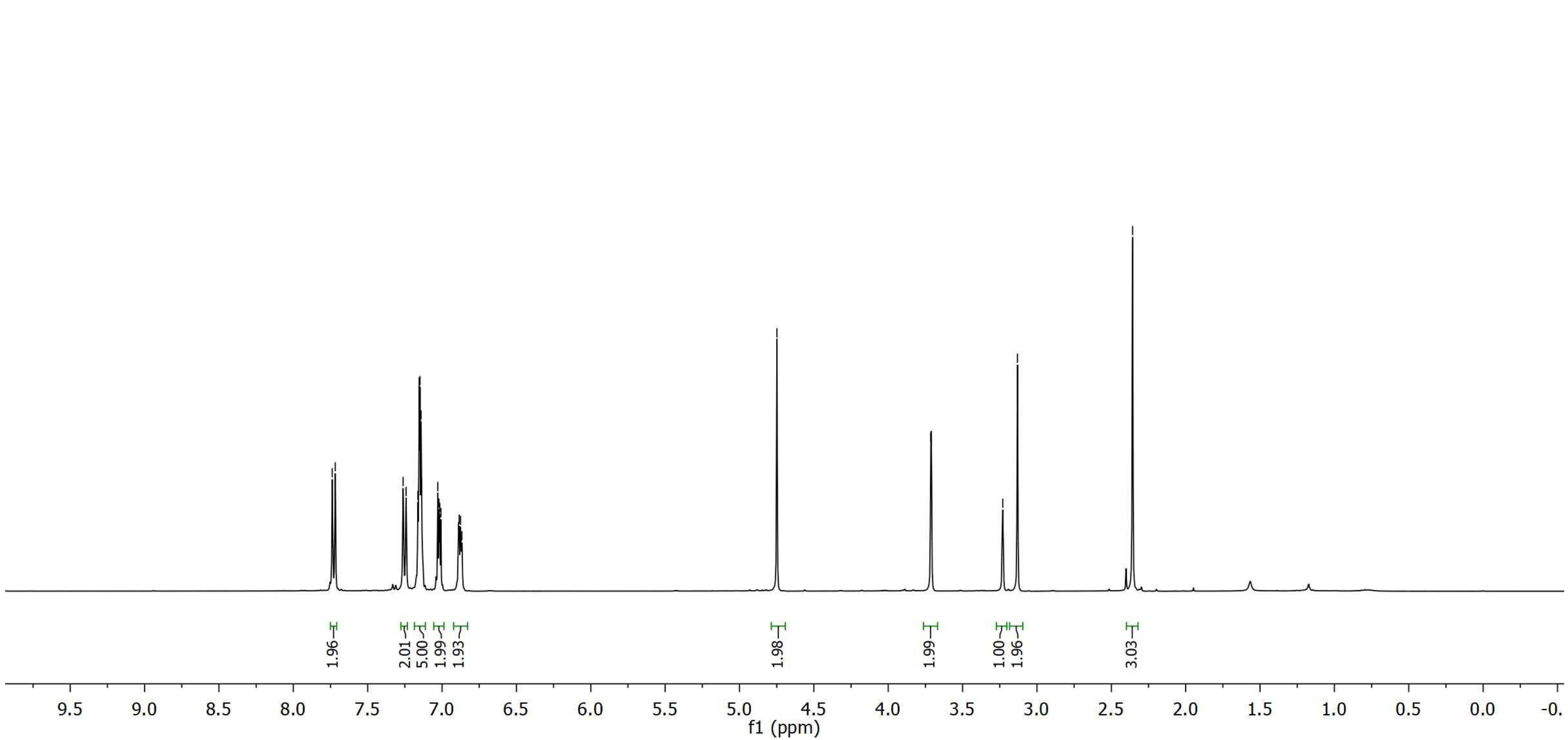

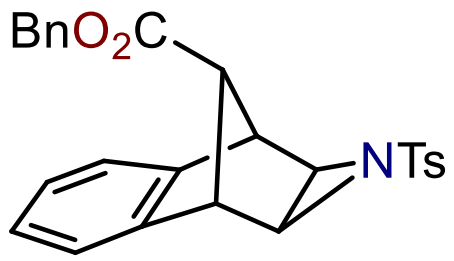

20

^{13}C NMR, 101 MHz, $CDCl_3$

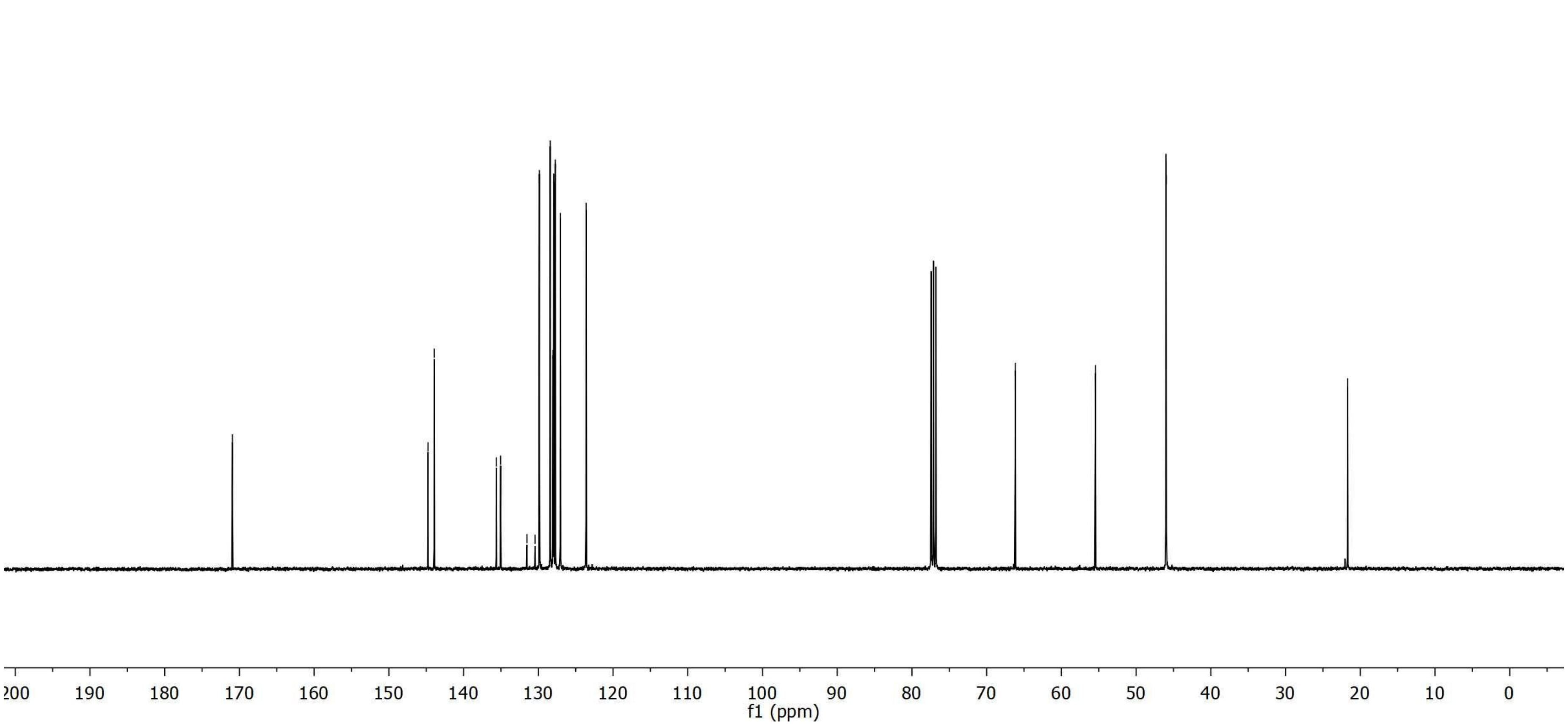

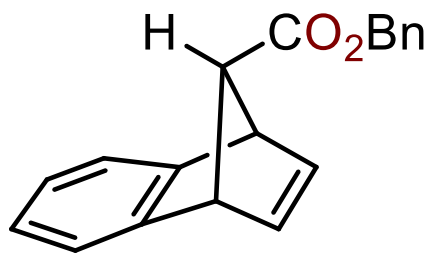

S13 – major isomer

^{1}H NMR, 400 MHz, $CDCl_3$

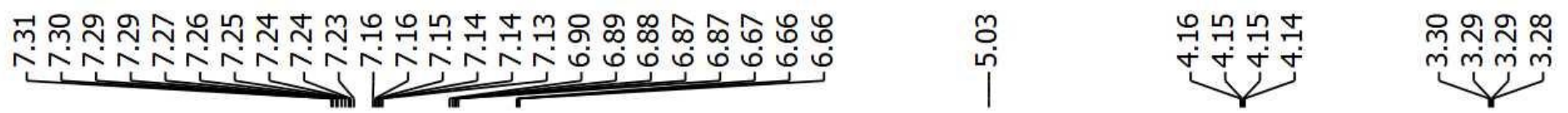

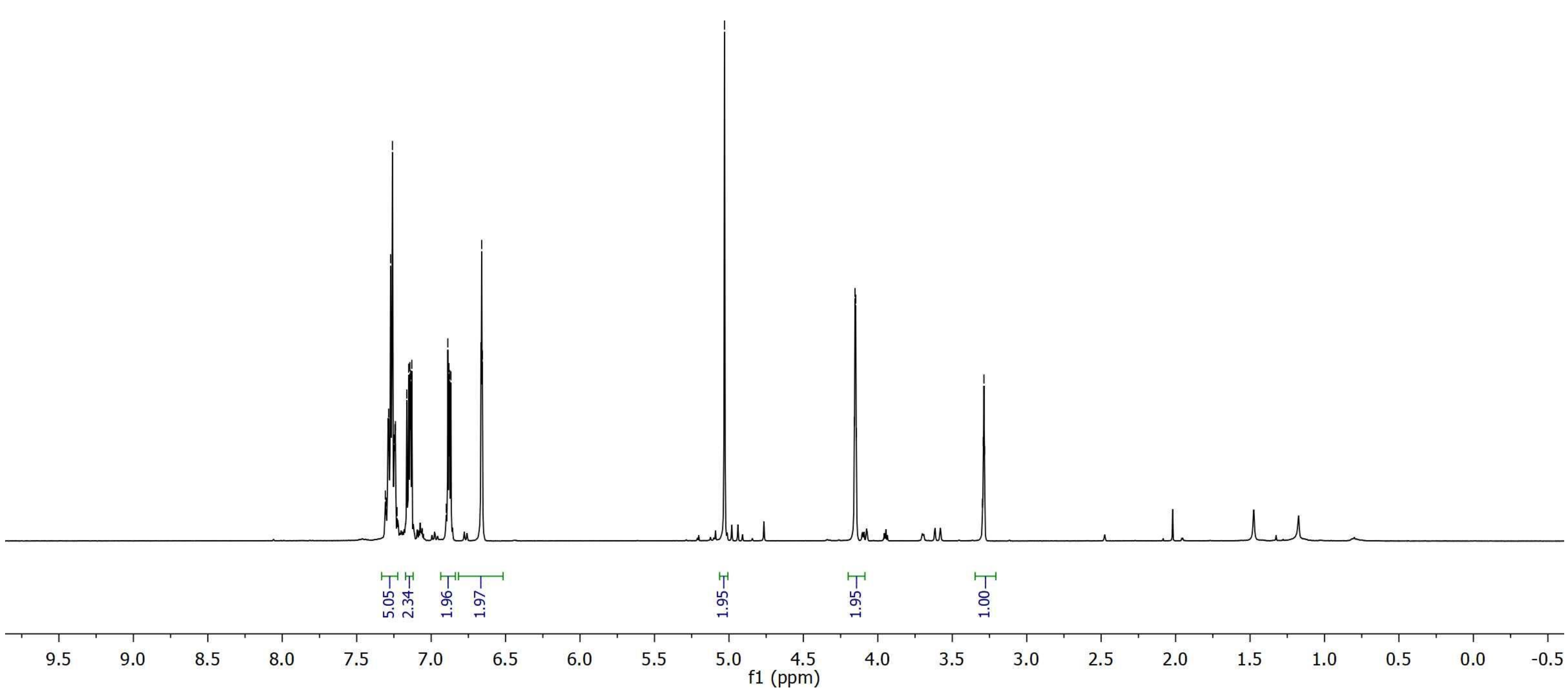

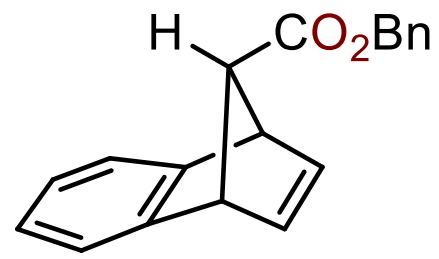

S13 – major isomer

^{13}C NMR, 101 MHz, $CDCl_3$

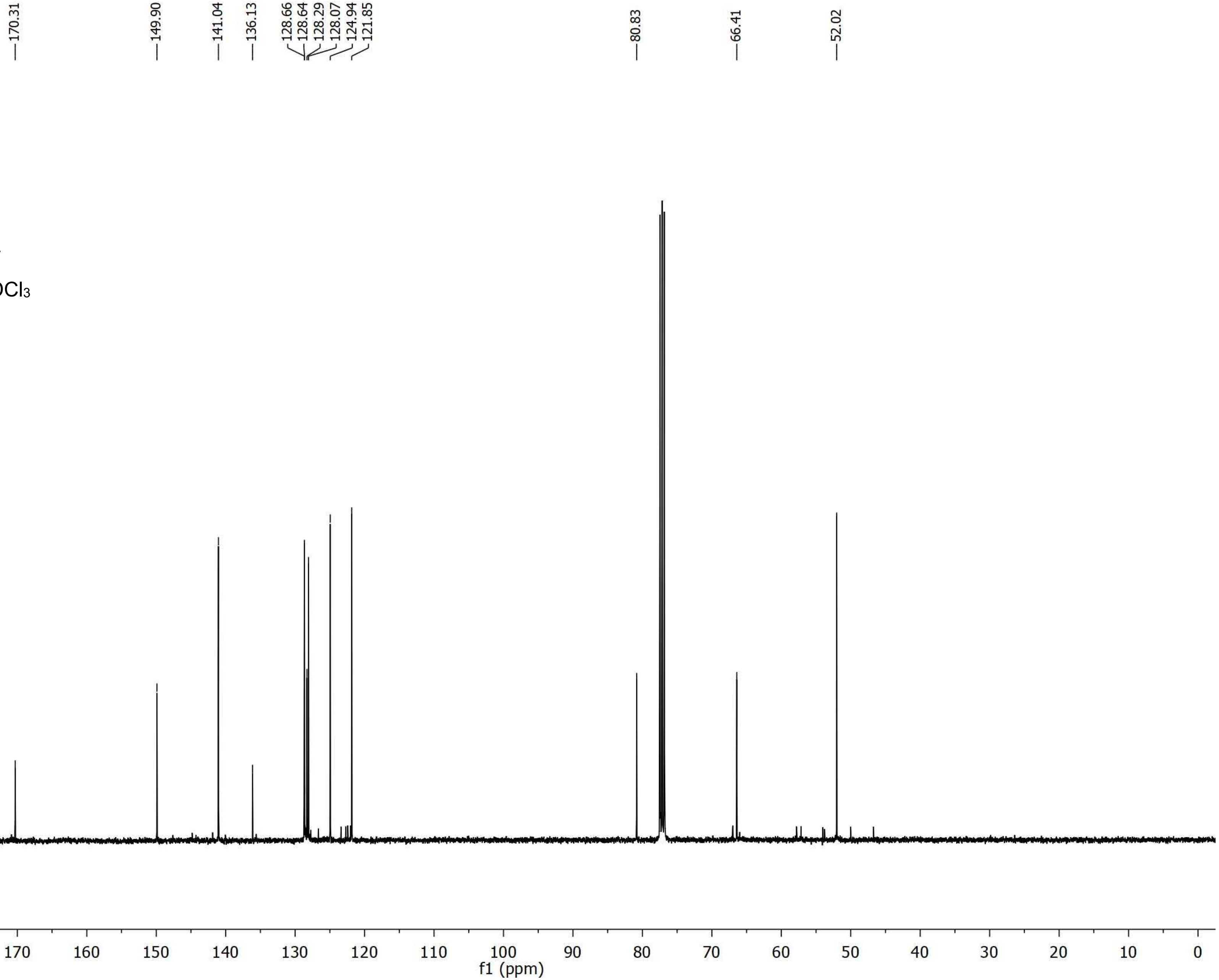

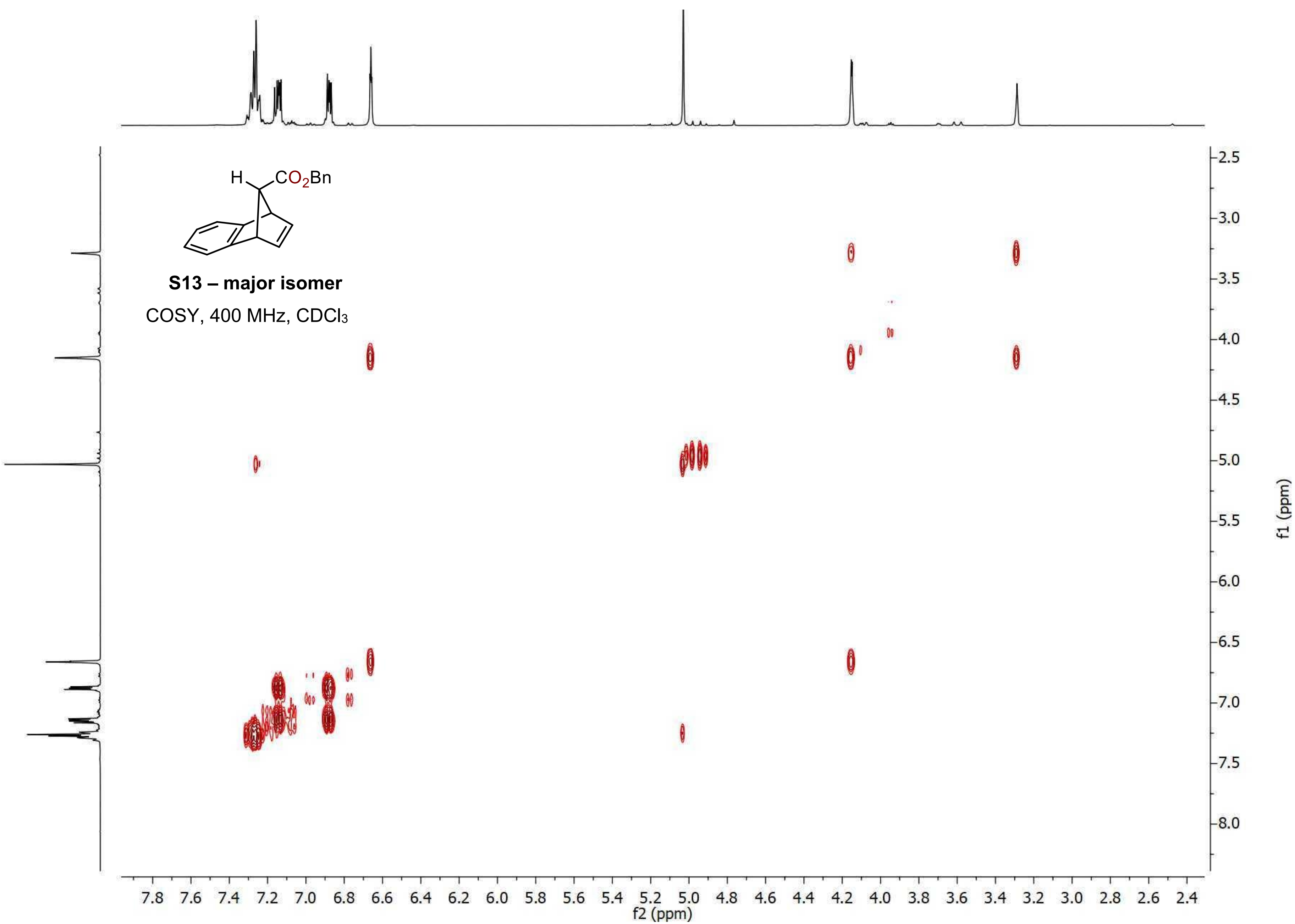
H
CO2Bn
S13 – major isomer
COSY, 400 MHz, CDCl3
f2 (ppm)
f1 (ppm)
7.8 7.6 7.4 7.2 7.0 6.8 6.6 6.4 6.2 6.0 5.8 5.6 5.4 5.2 5.0 4.8 4.6 4.4 4.2 4.0 3.8 3.6 3.4 3.2 3.0 2.8 2.6 2.4
2.5 3.0 3.5 4.0 4.5 5.0 5.5 6.0 6.5 7.0 7.5 8.0

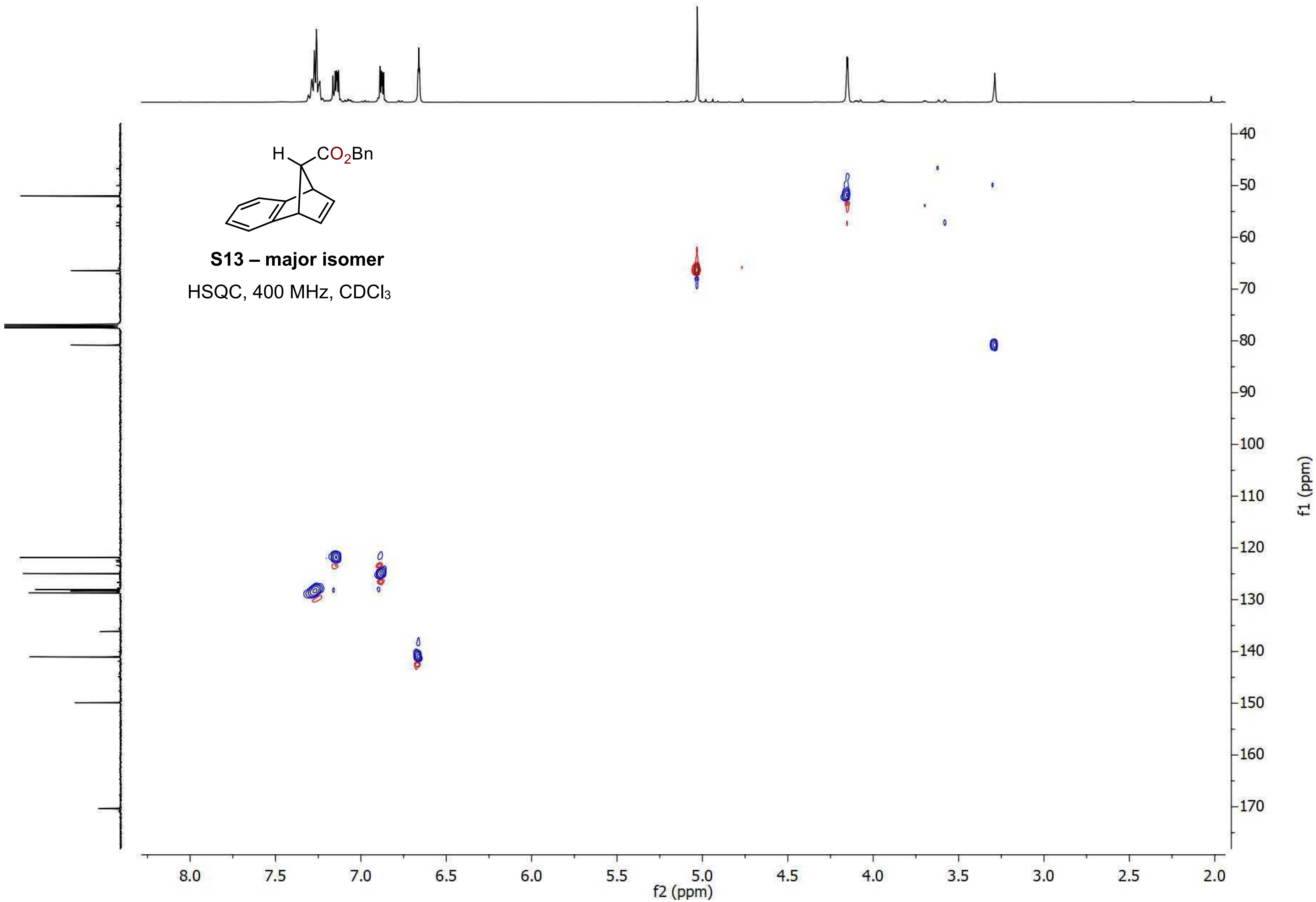

S13 – major isomer

HSQC, 400 MHz, $CDCl_3$

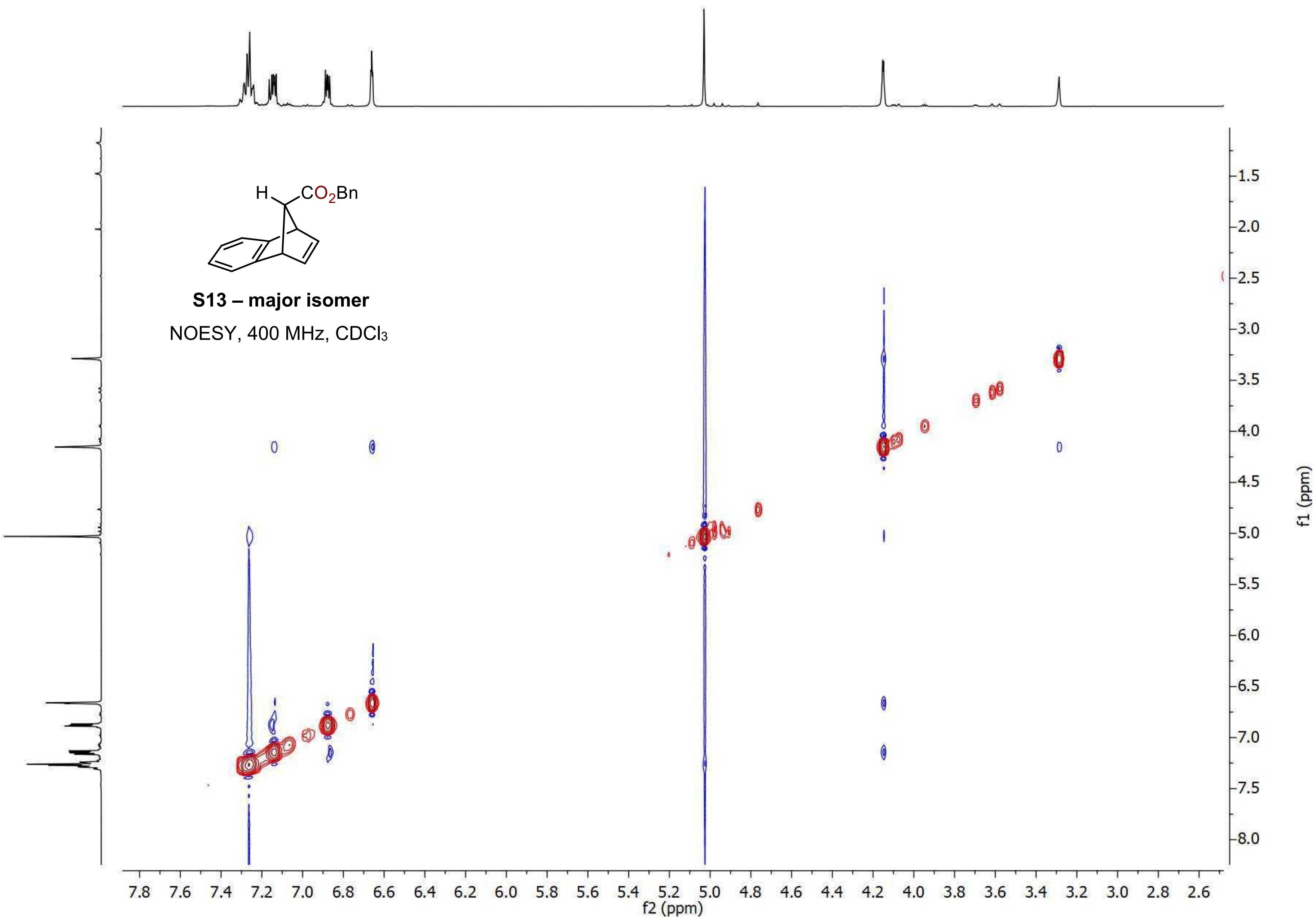

H
CO2Bn
S13 – major isomer
NOESY, 400 MHz, CDCl3
f2 (ppm)
f1 (ppm)

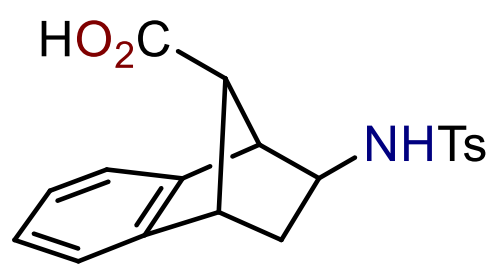

S14

^1H NMR, 400 MHz, MeOD

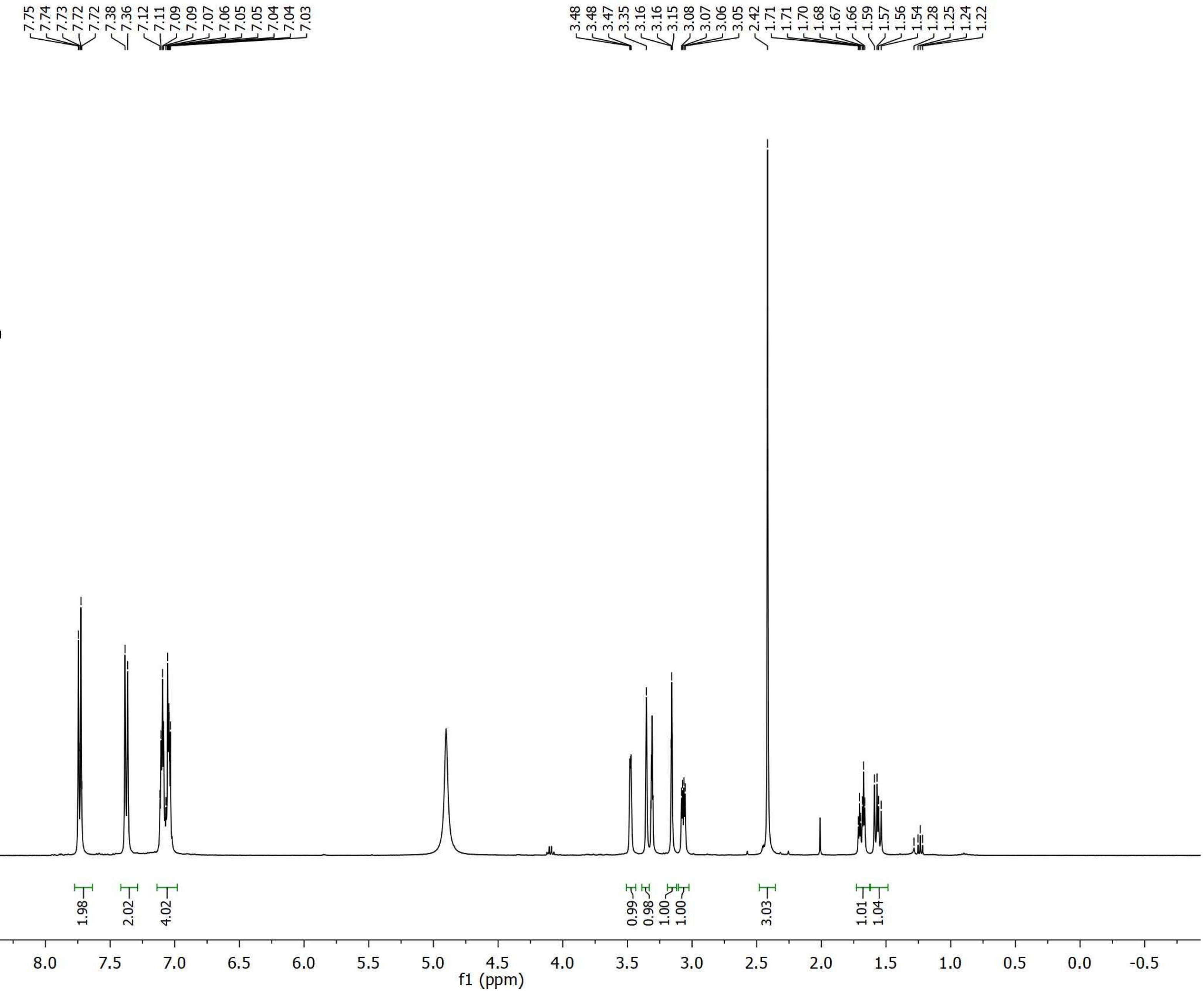

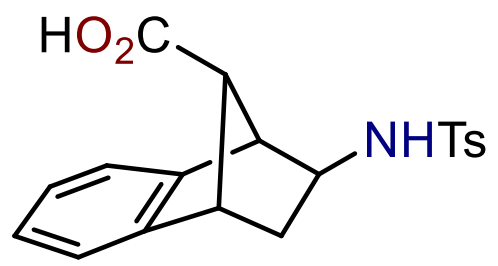

S14

^{13}C NMR, 101 MHz, MeOD

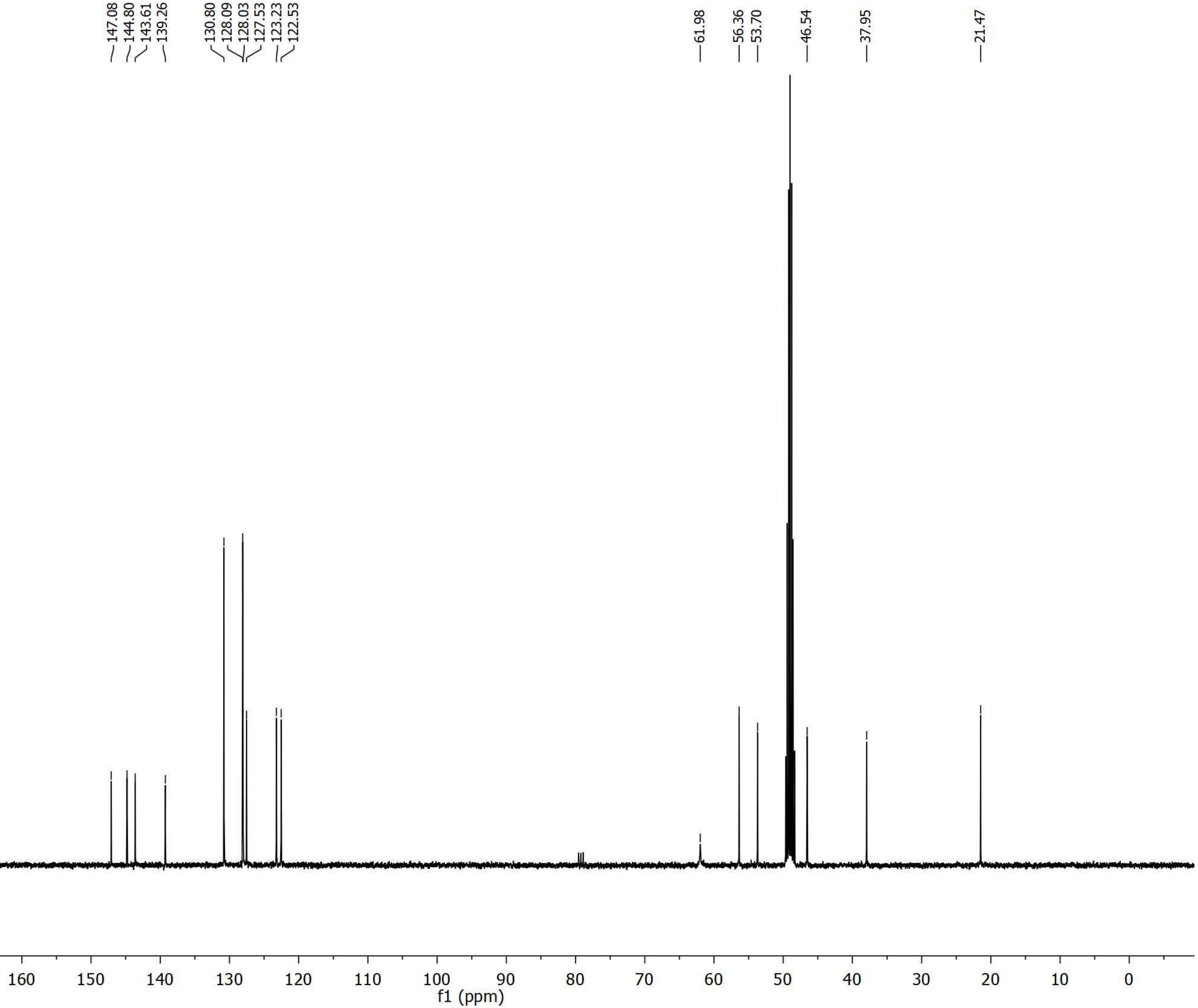

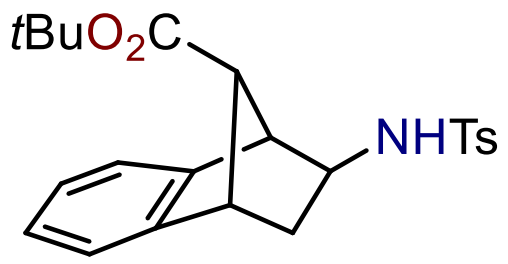

S15

^{1}H NMR, 400 MHz, $CDCl_3$

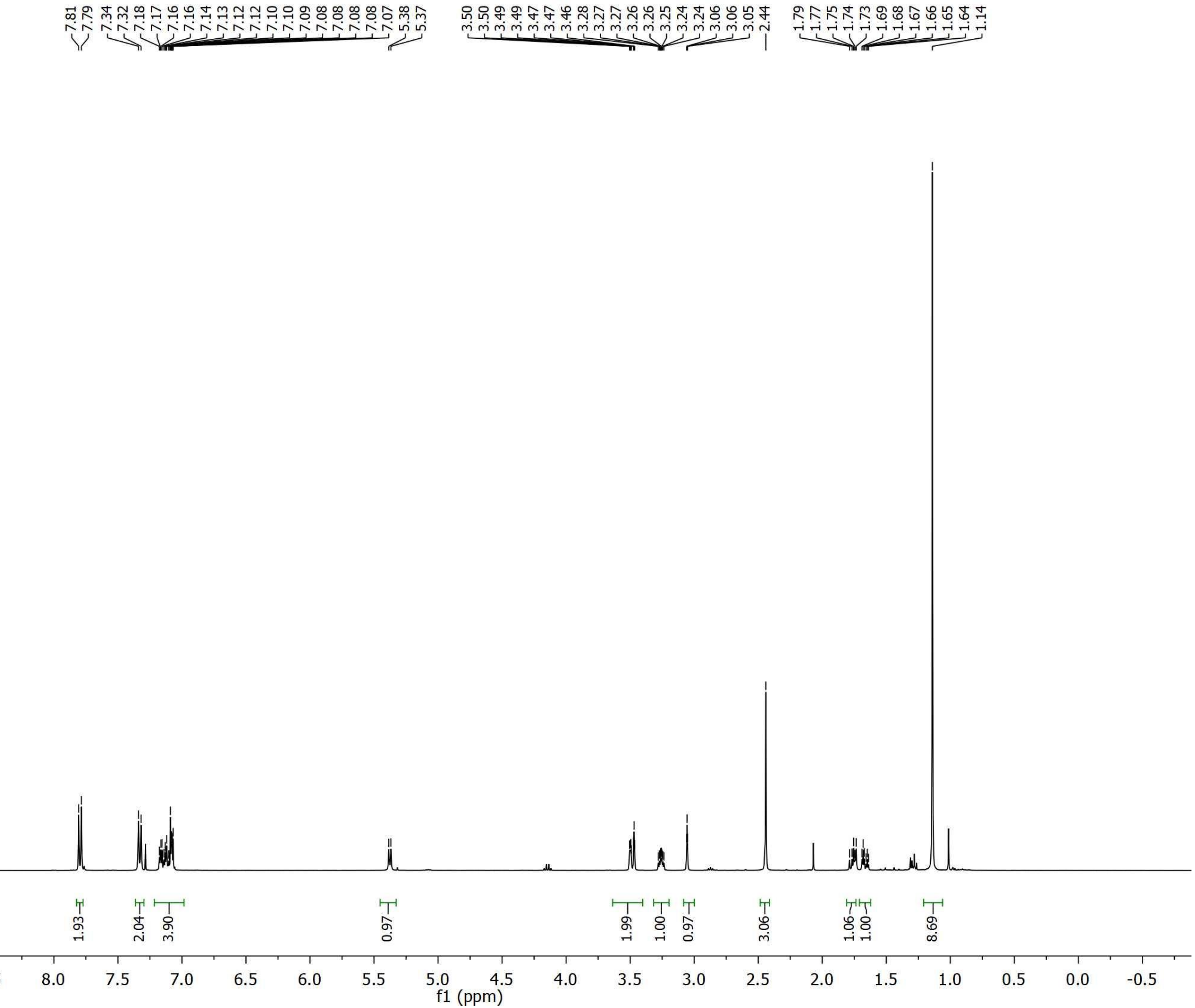

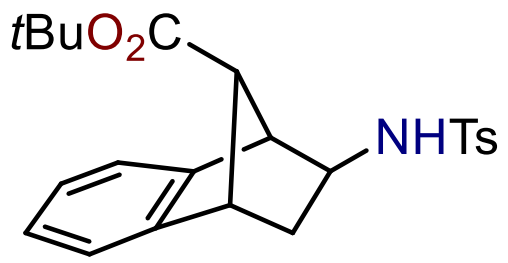

S15

^{13}C NMR, 101 MHz, $CDCl_3$

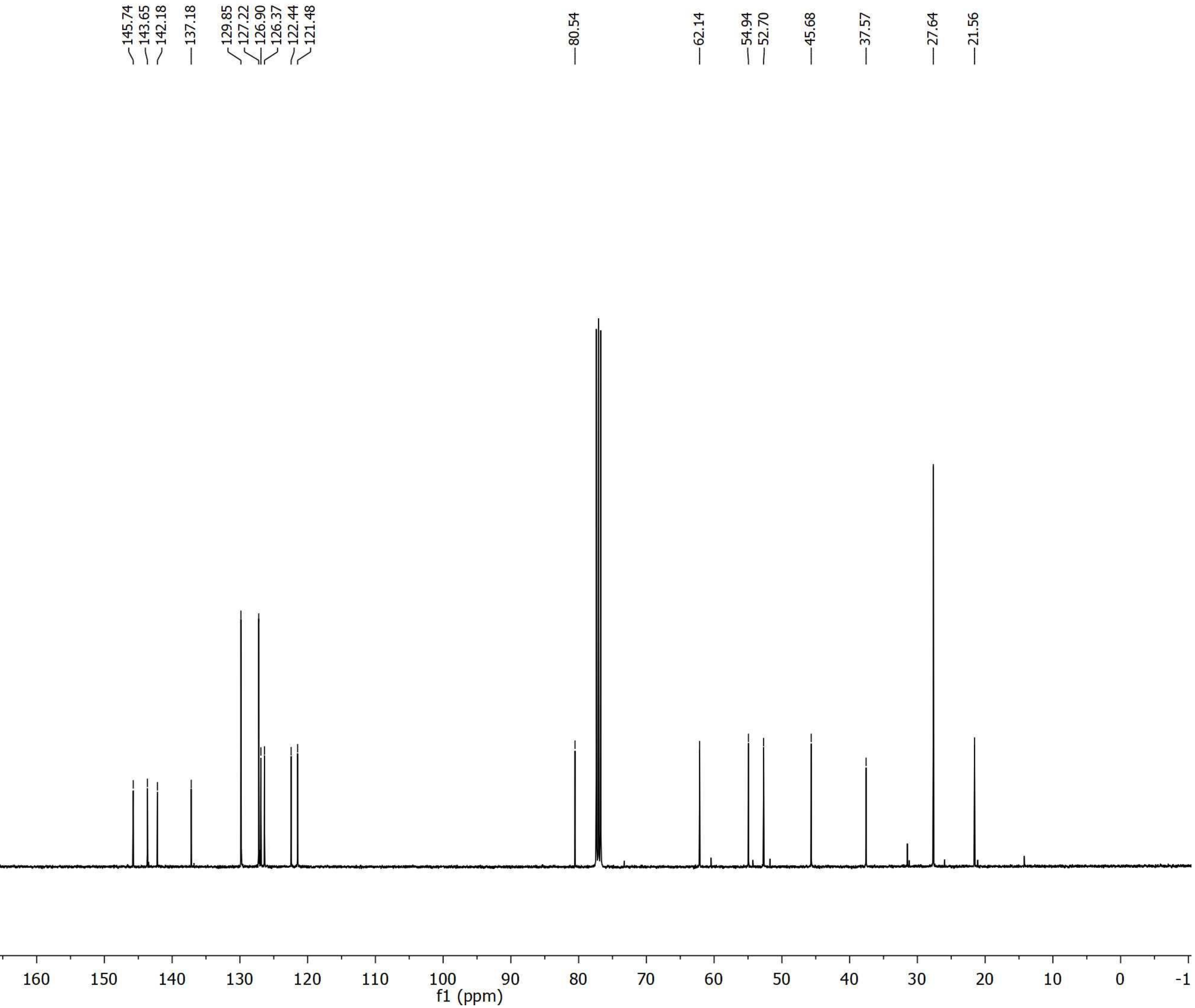

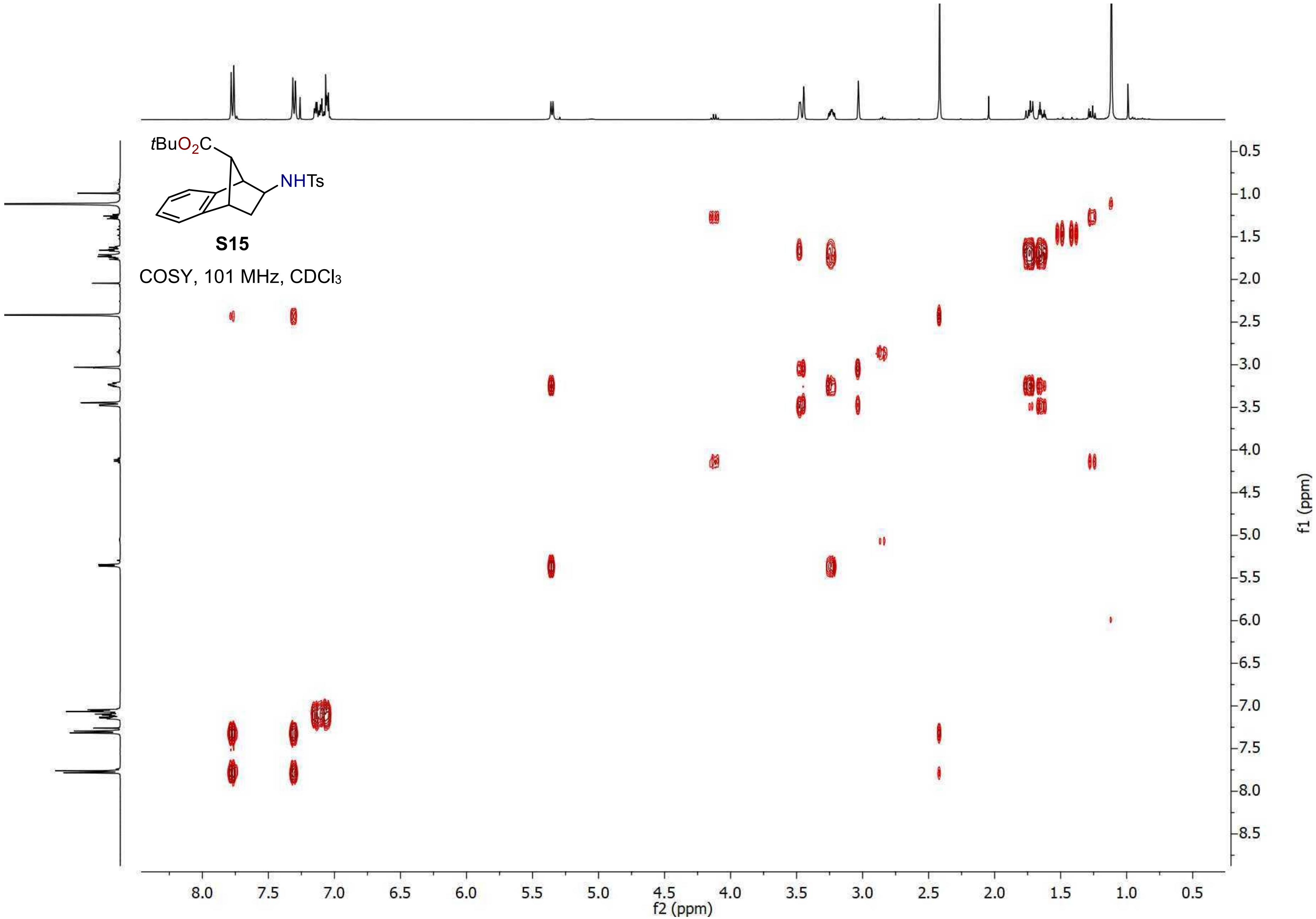

tBuO2C
NHTs
S15
COSY, 101 MHz, CDCl3
f1 (ppm)
f2 (ppm)

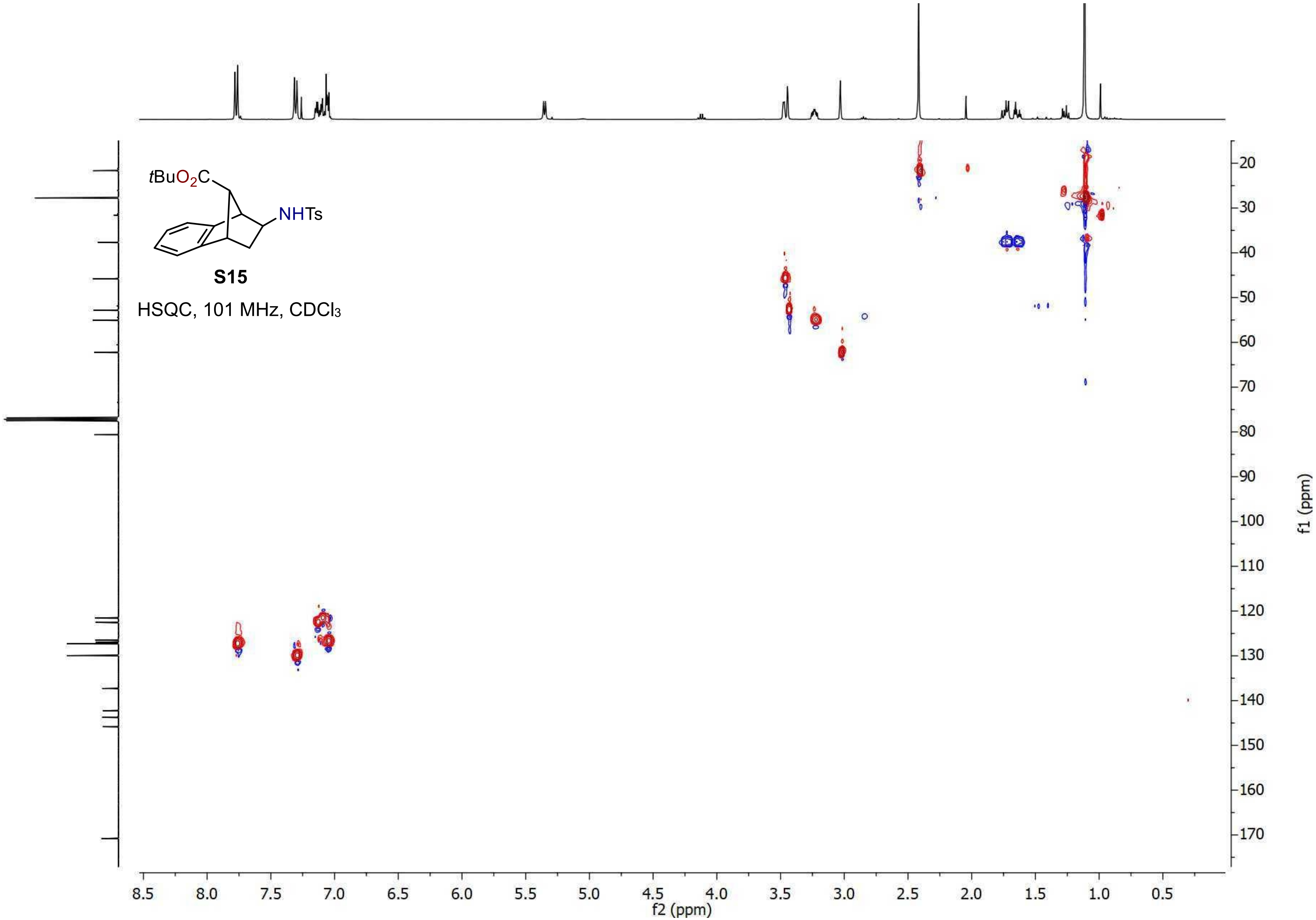

tBuO2C
NHTs
S15
HSQC, 101 MHz, CDCl3
f2 (ppm)
f1 (ppm)

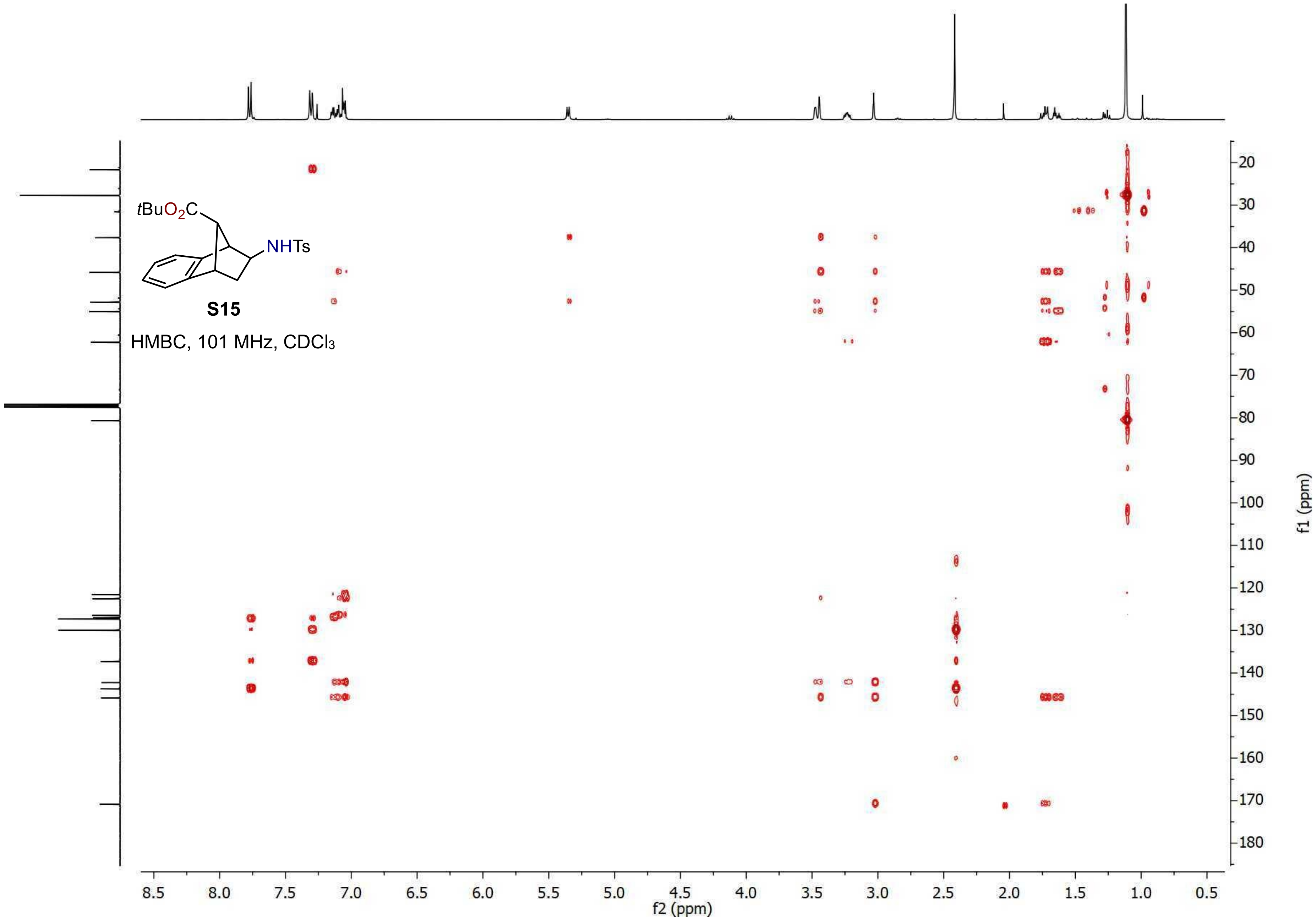

tBuO2C
NHTs
S15
HMBC, 101 MHz, CDCl3
f2 (ppm)
f1 (ppm)

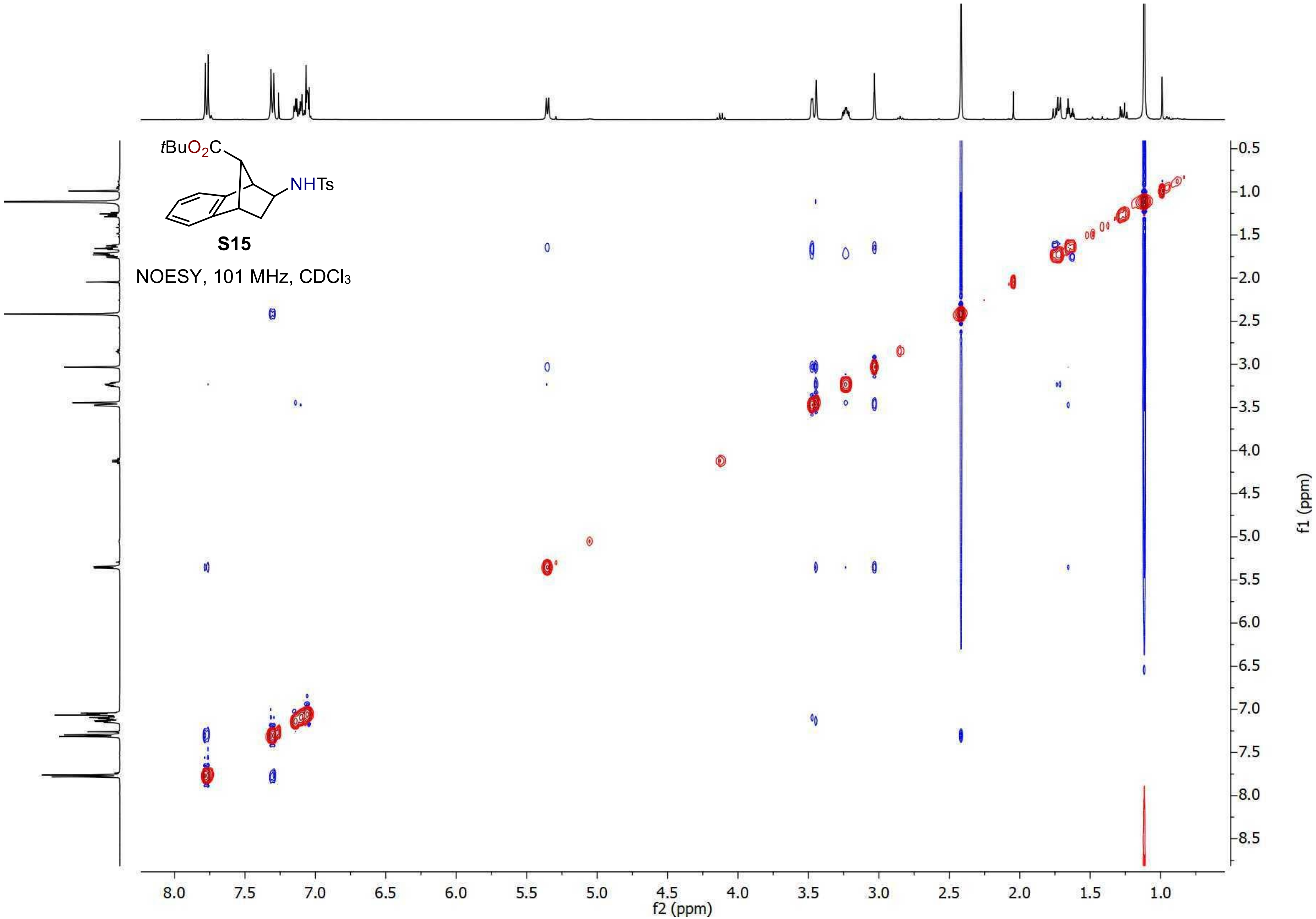

tBuO2C
NHTs
S15
NOESY, 101 MHz, CDCl3
f1 (ppm)
f2 (ppm)
8.0
7.5
7.0
6.5
6.0
5.5
5.0
4.5
4.0
3.5
3.0
2.5
2.0
1.5
1.0
0.5
8.5

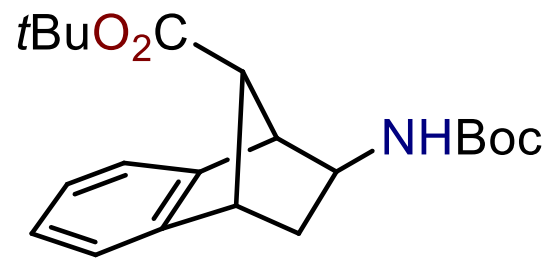

S16

^1H NMR, 400 MHz, $CDCl_3$

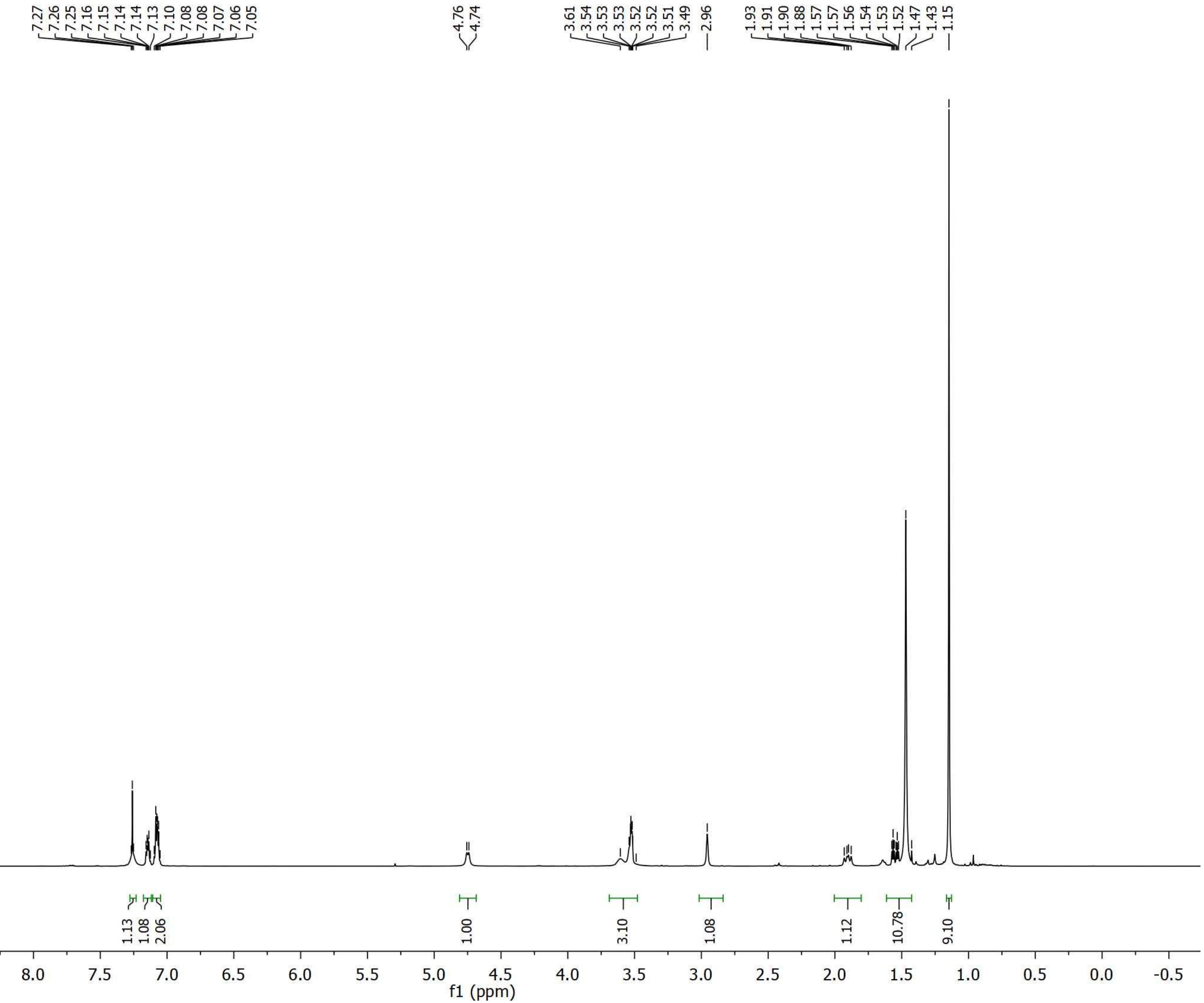

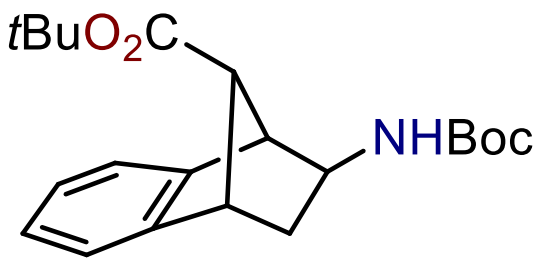

S16

^{13}C NMR, 101 MHz, $CDCl_3$

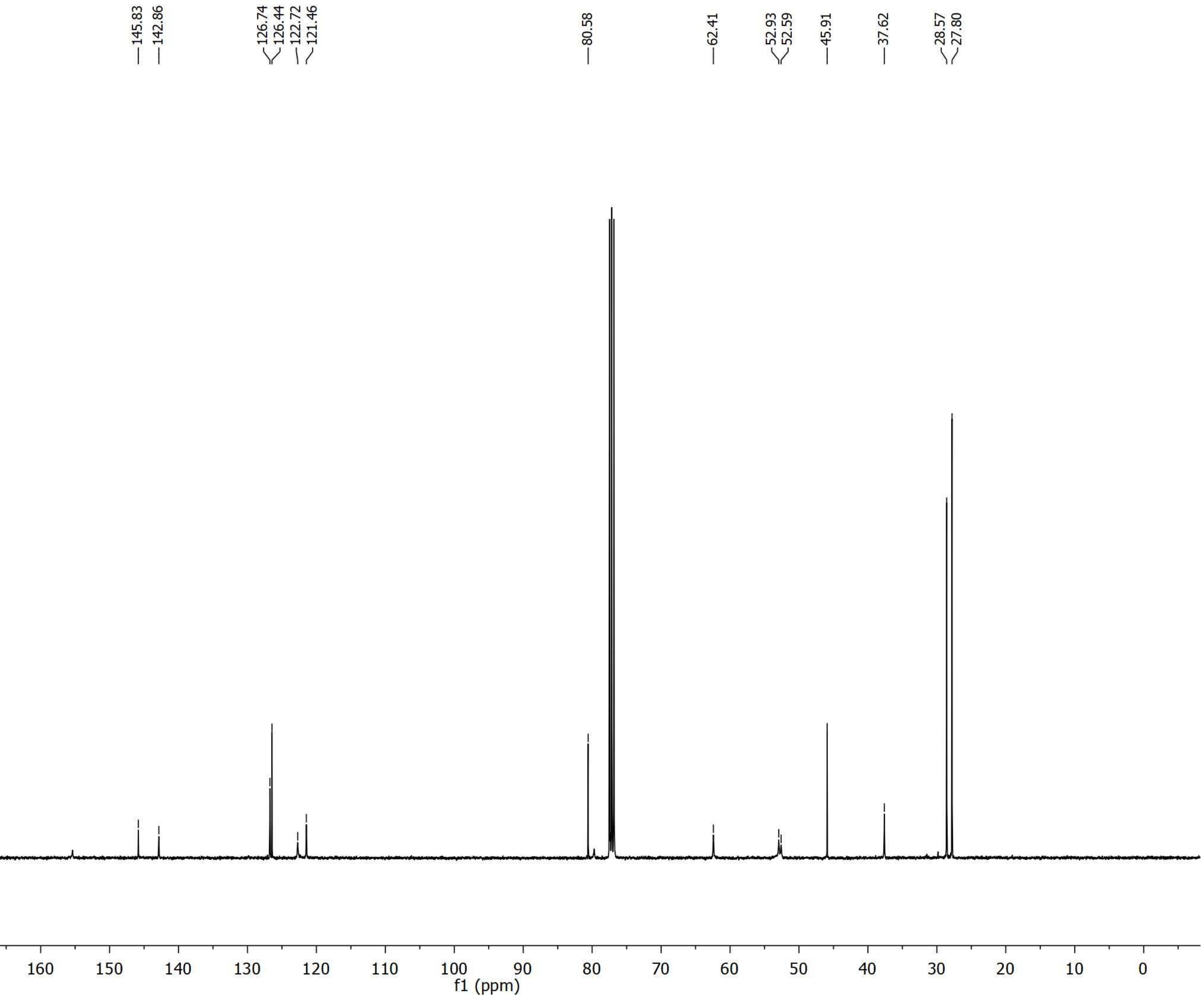

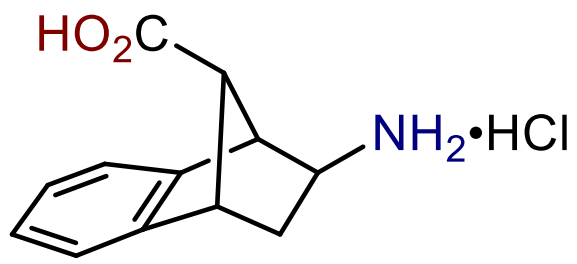

21

^{1}H NMR, 400 MHz, D_2O

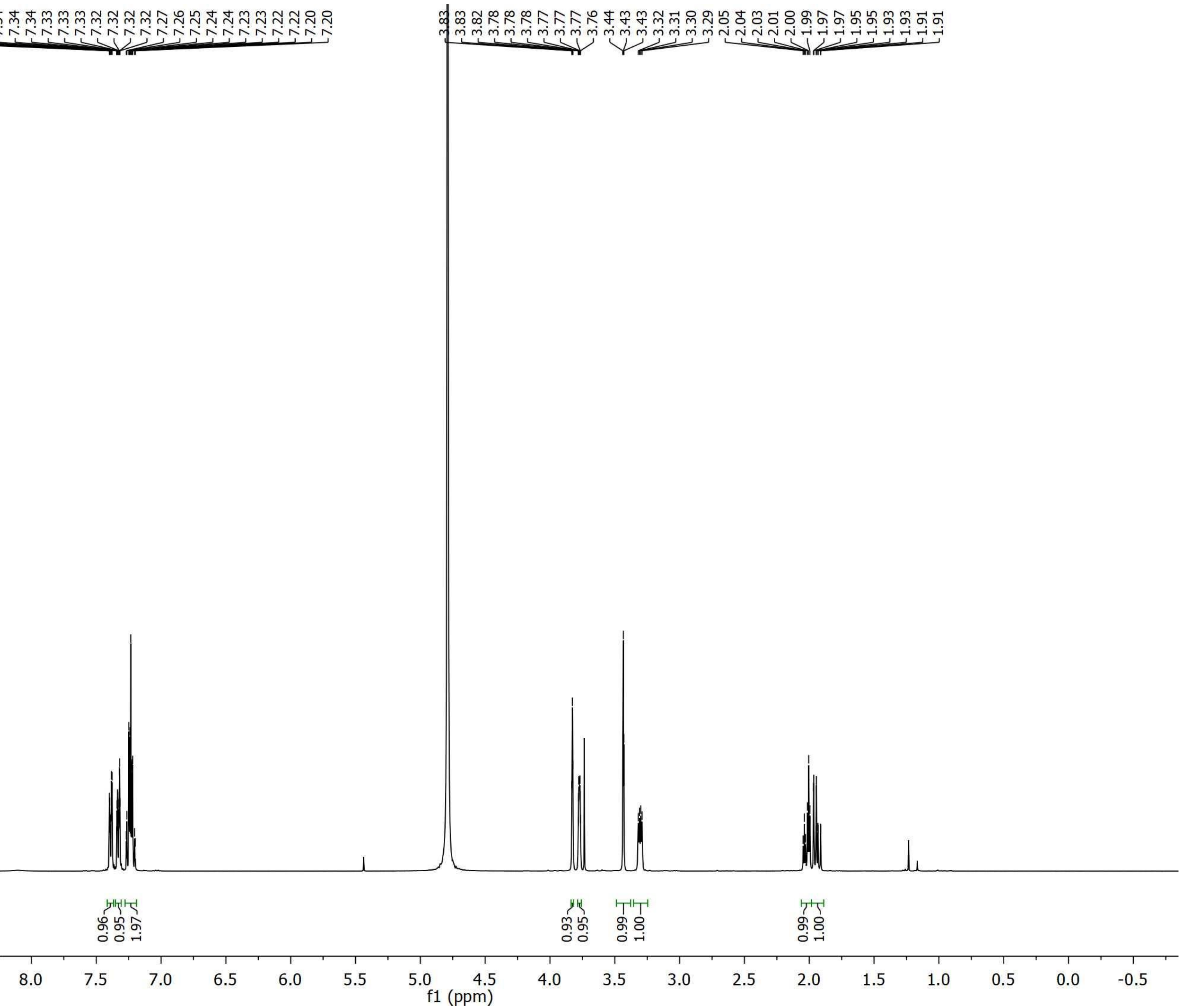

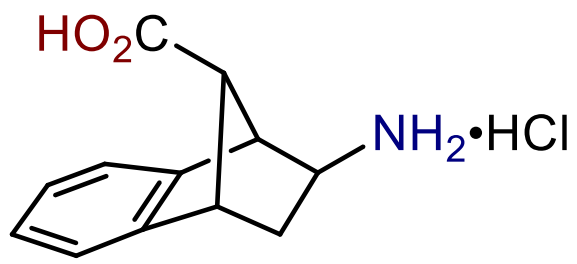

21

^{13}C NMR, 101 MHz, D_2O

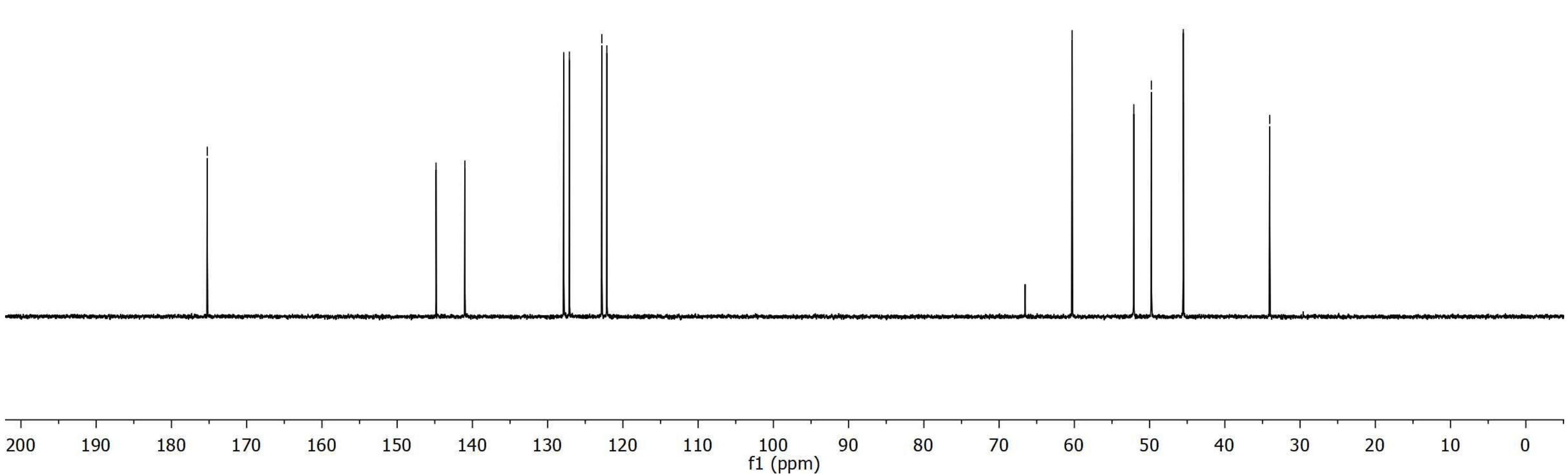

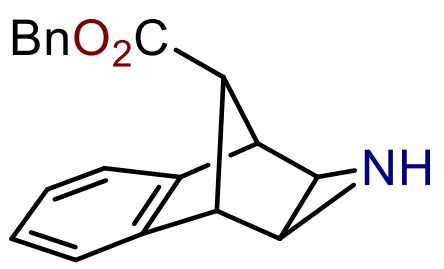

S17

^{1}H NMR, 400 MHz, $CDCl_3$

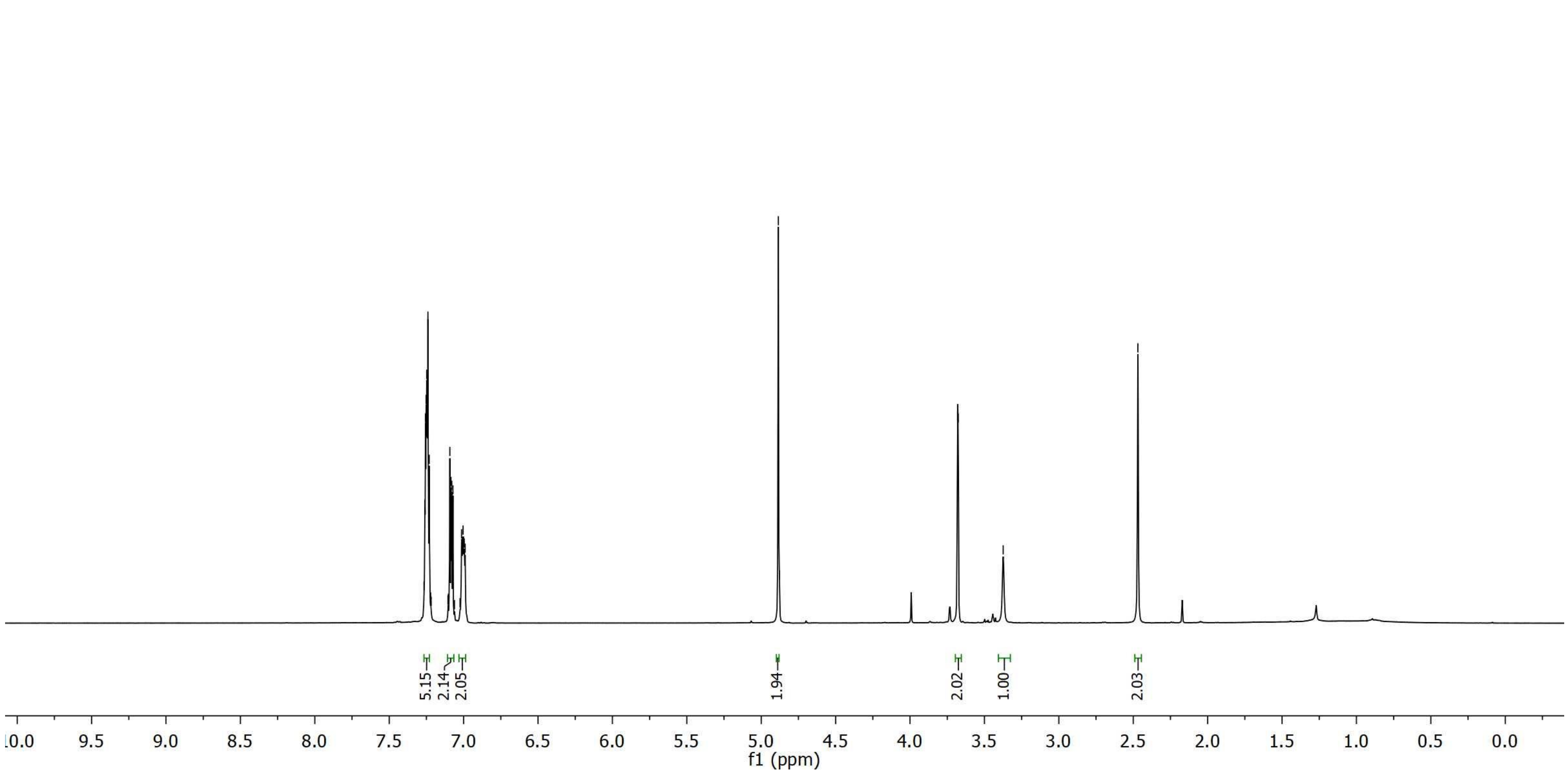

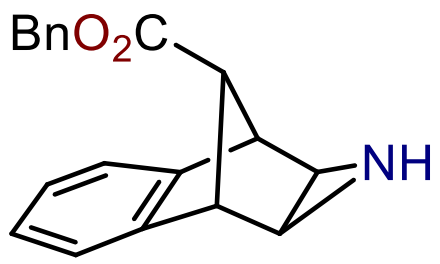

S17

^{13}C NMR, 101 MHz, $CDCl_3$

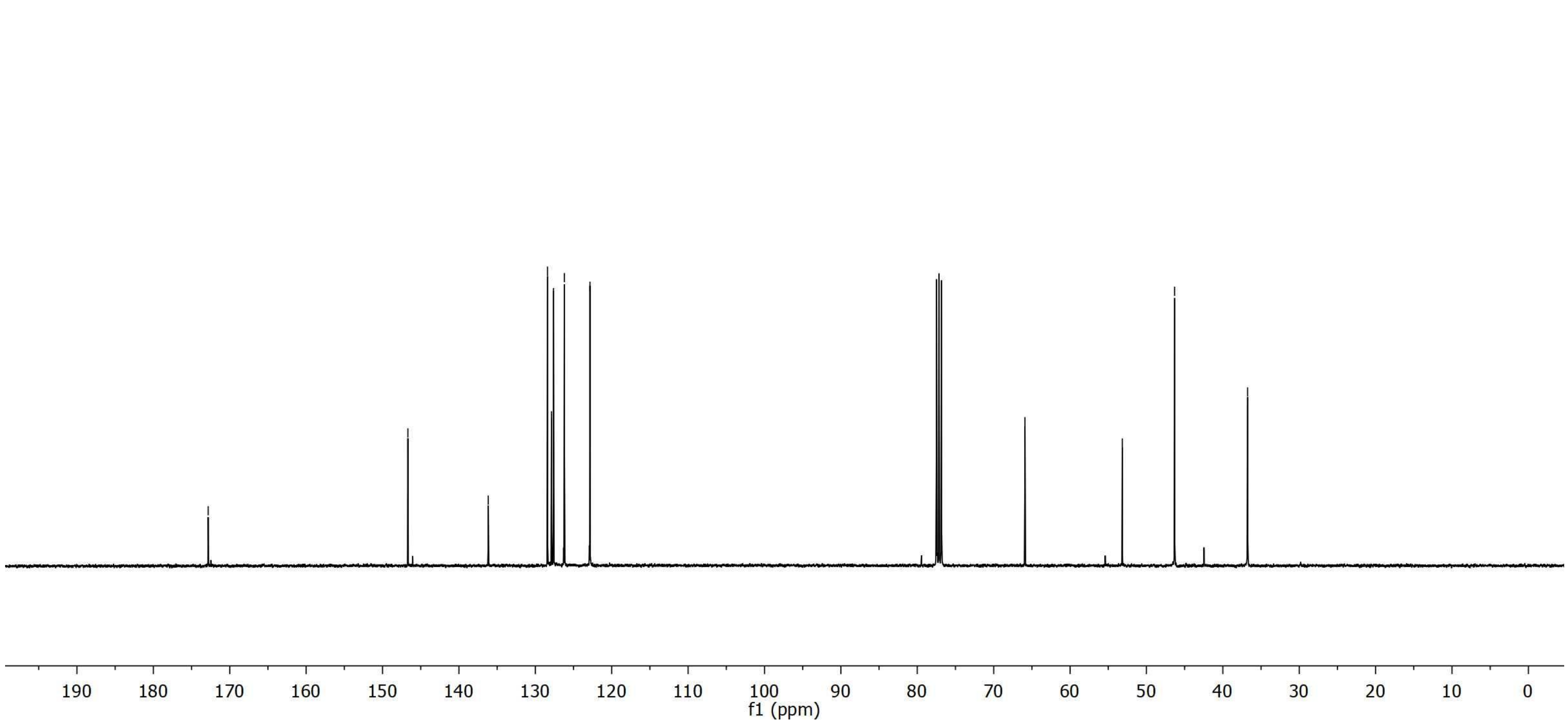

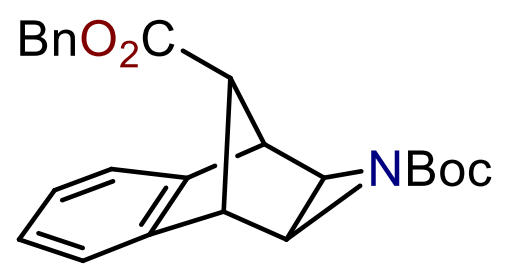

S18

^{1}H NMR, 400 MHz, $CDCl_3$

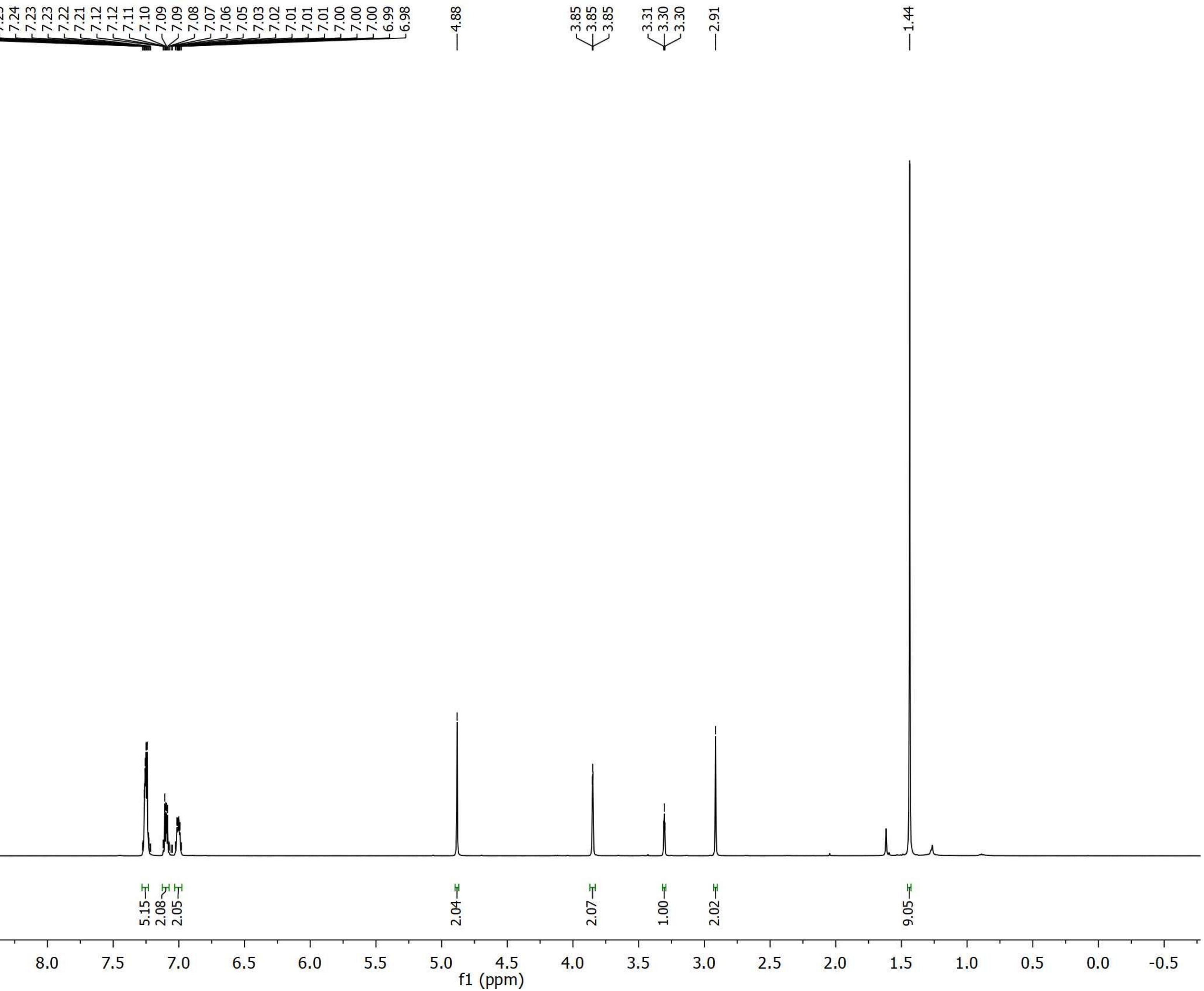

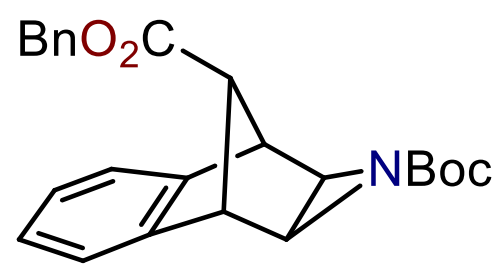

S18

^{13}C NMR, 101 MHz, $CDCl_3$

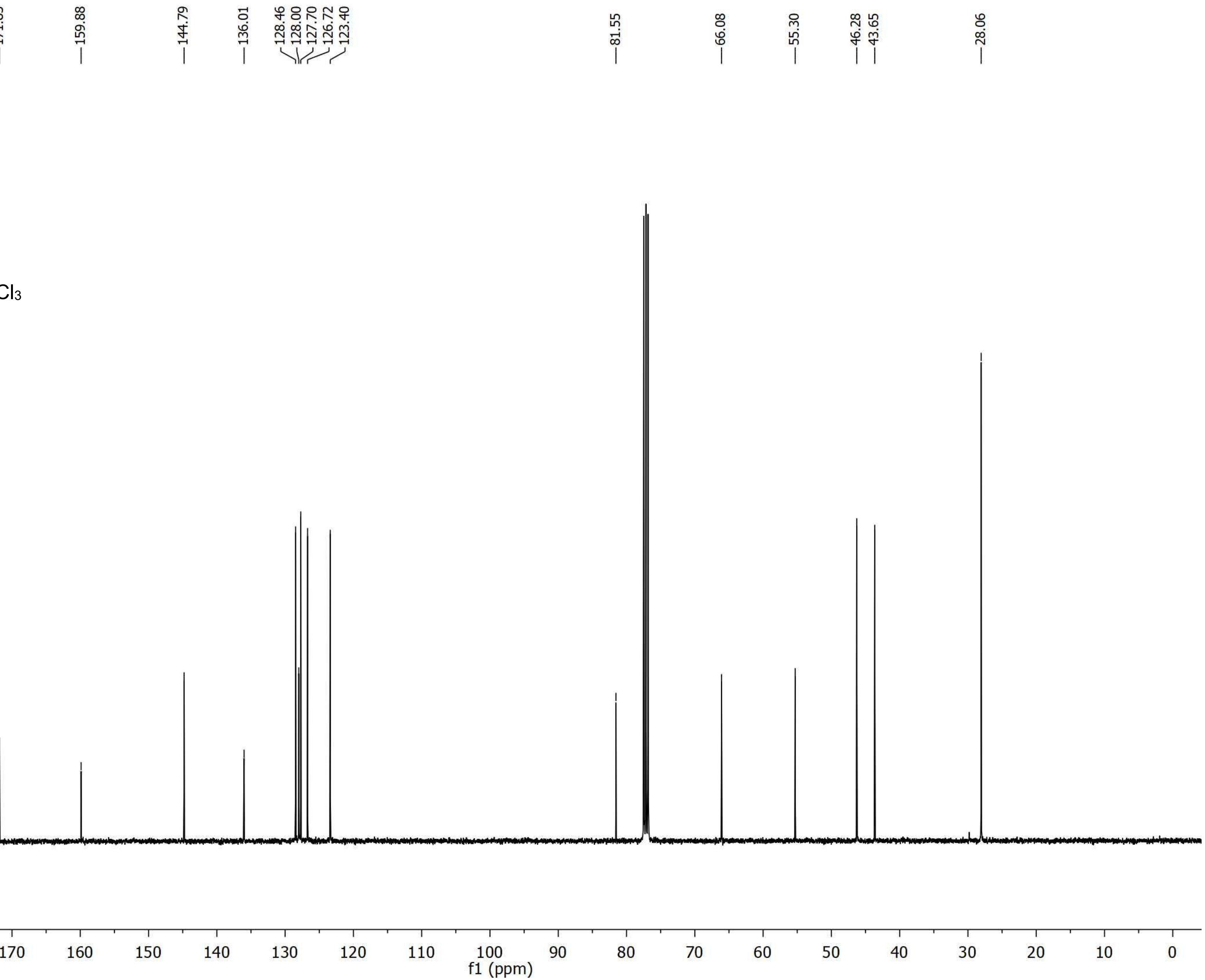

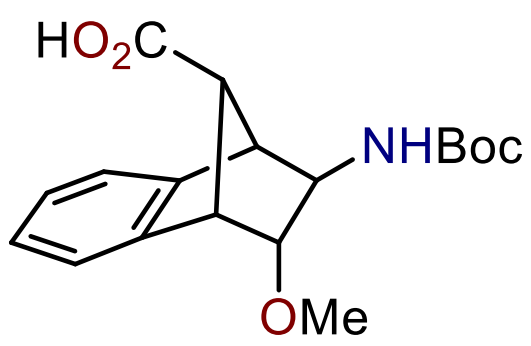

S19

^{1}H NMR, 400 MHz, MeOD

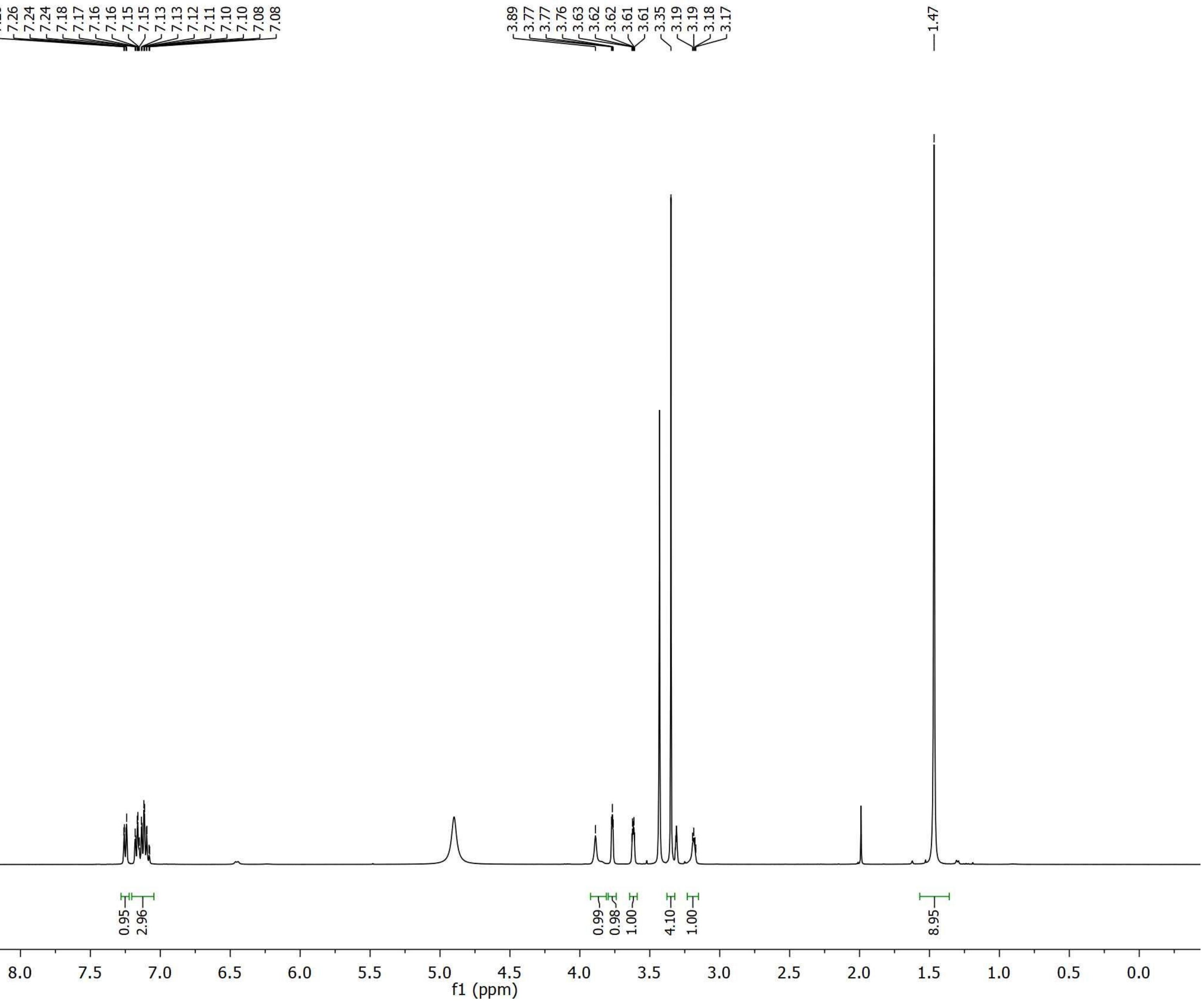

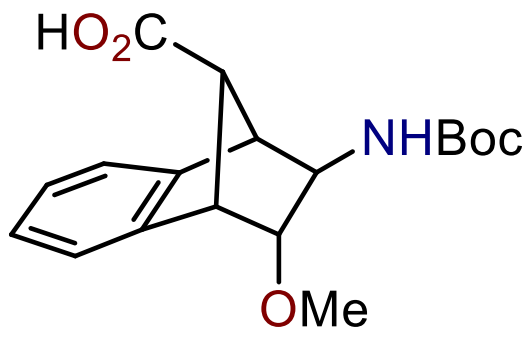

S19

^{13}C NMR, 101 MHz, MeOD

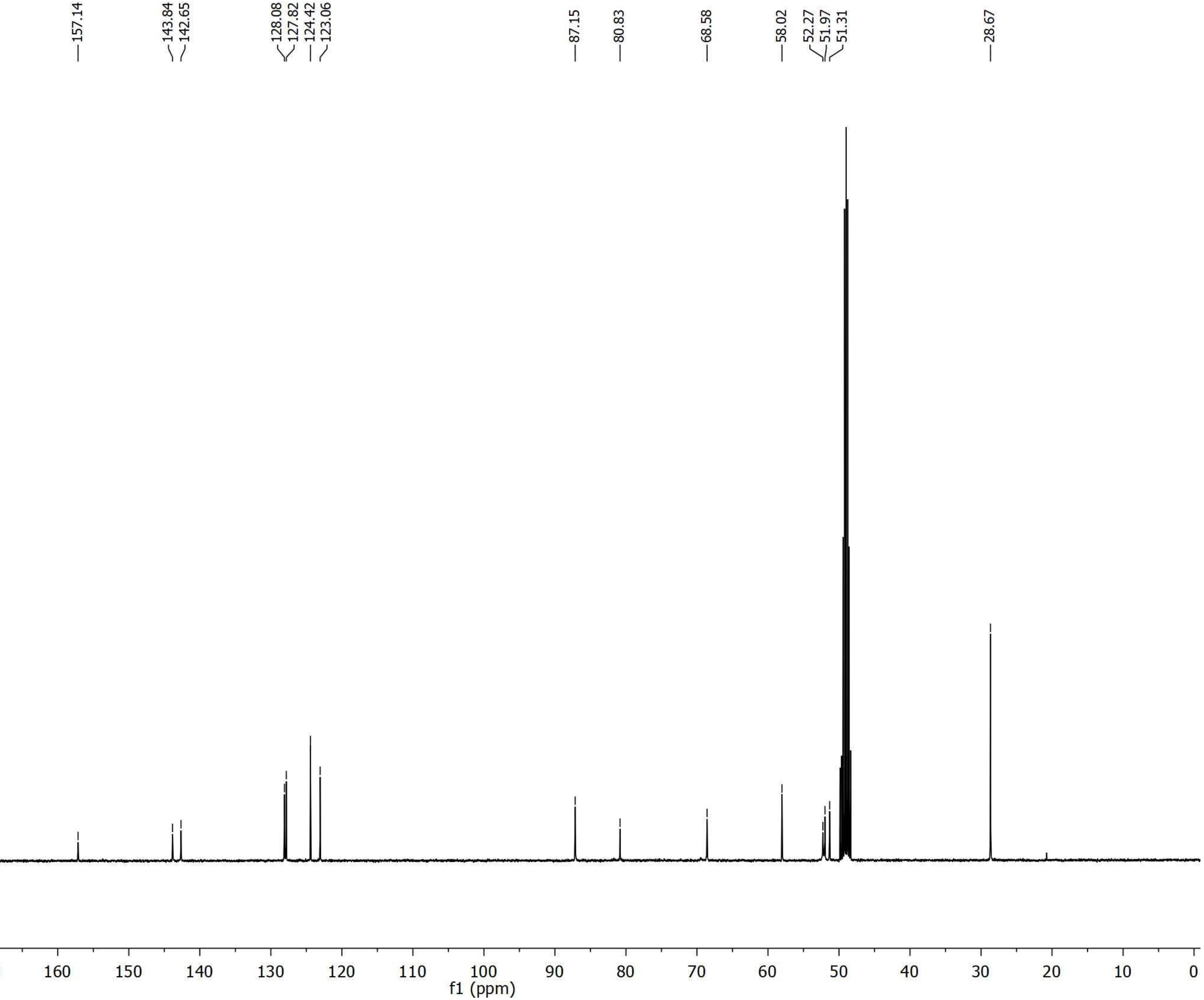

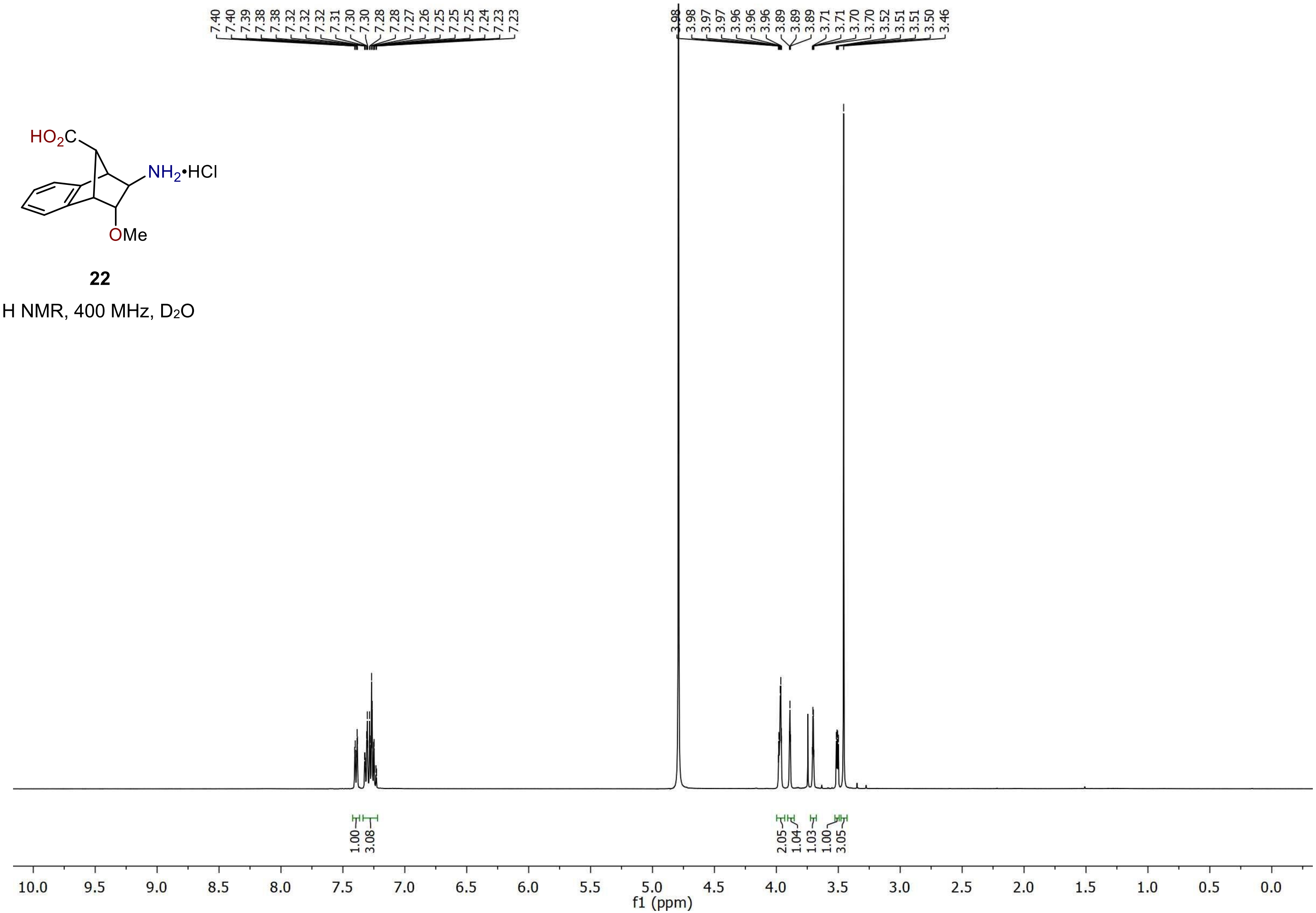

22

^{1}H NMR, 400 MHz, D_2O

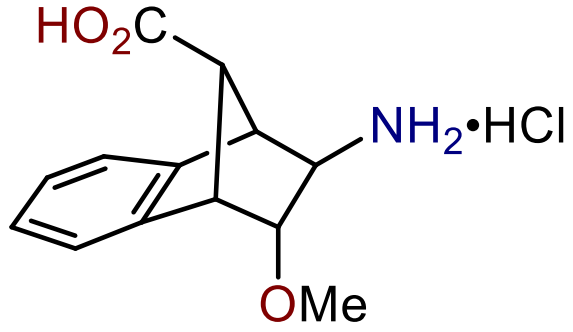

22

^{13}C NMR, 101 MHz, D_2O

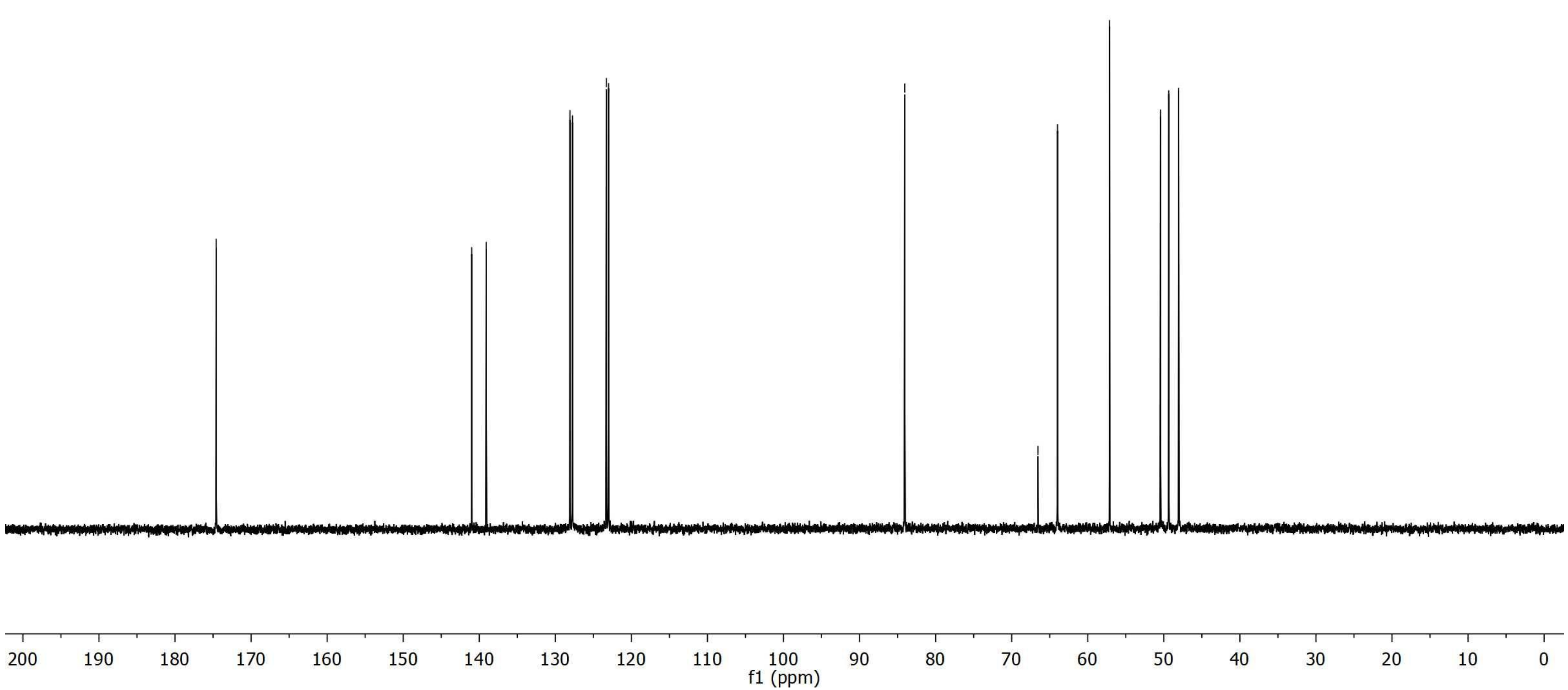

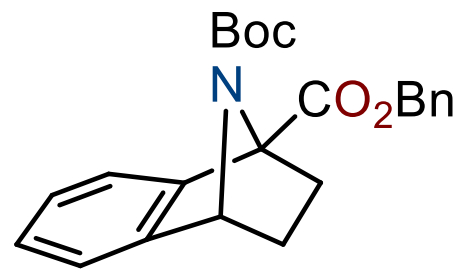

S20

^{1}H NMR, 400 MHz, $CDCl_3$

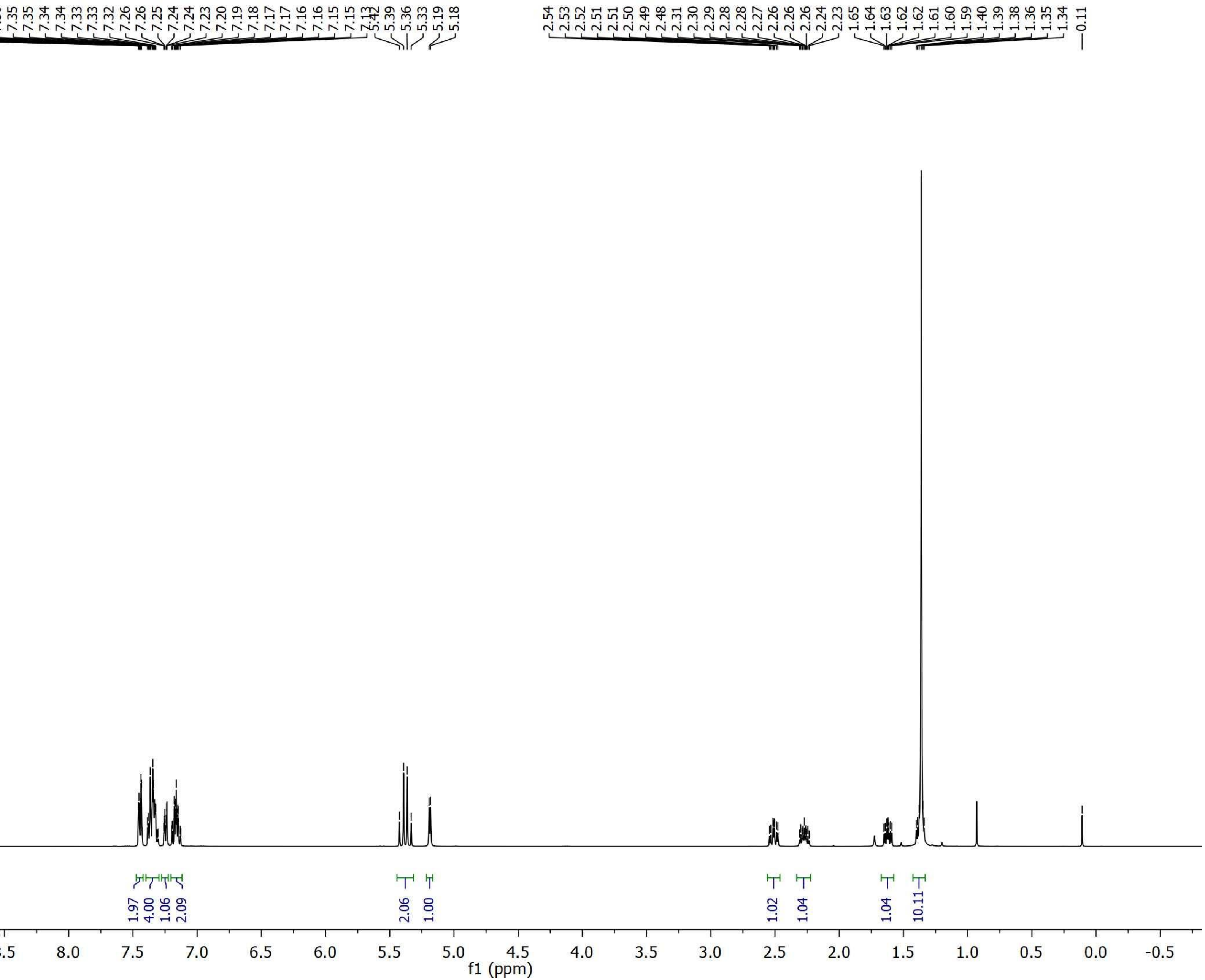

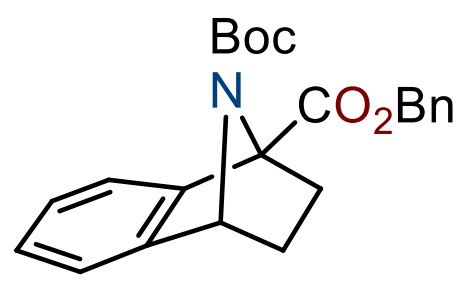

S20

^{13}C NMR, 101 MHz, $CDCl_3$

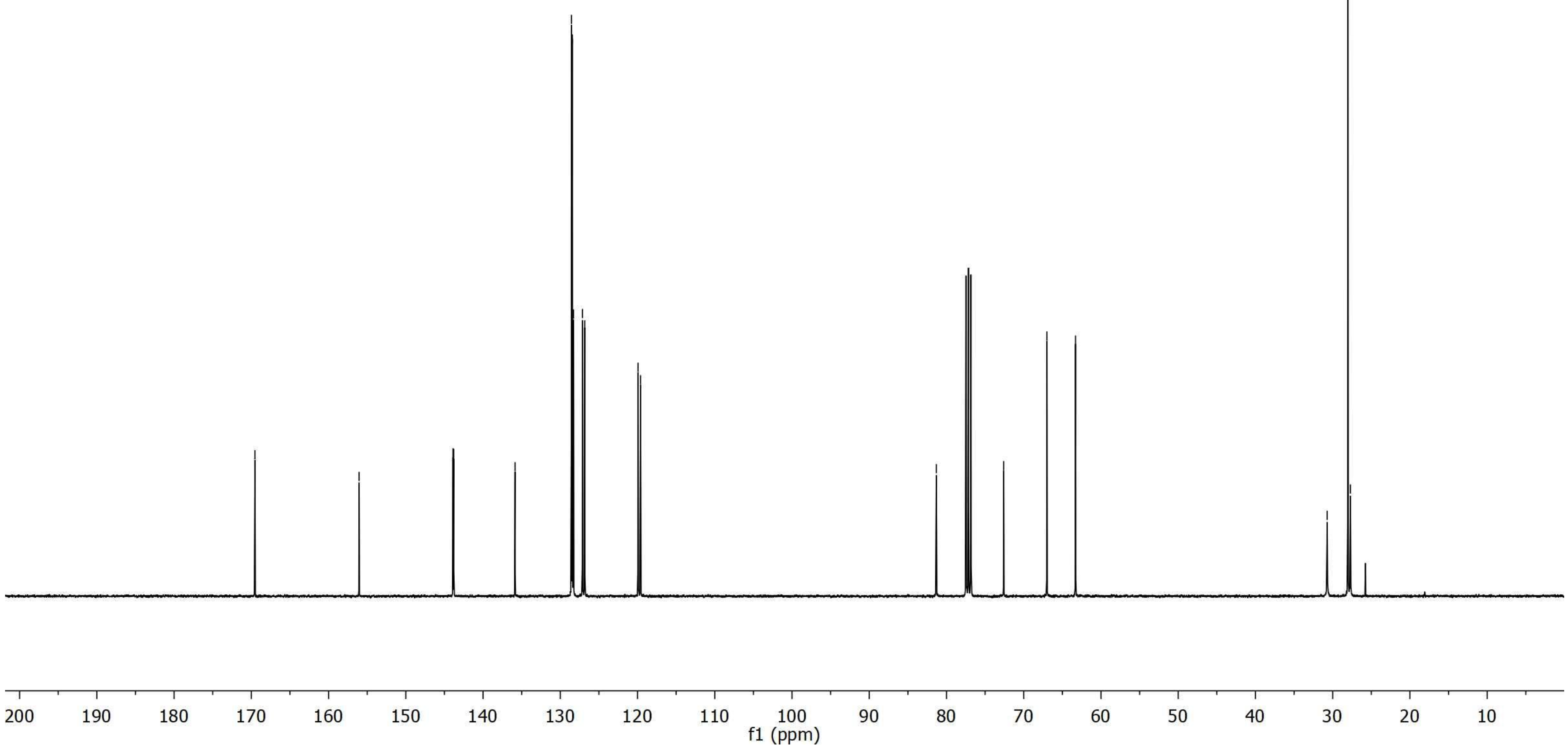

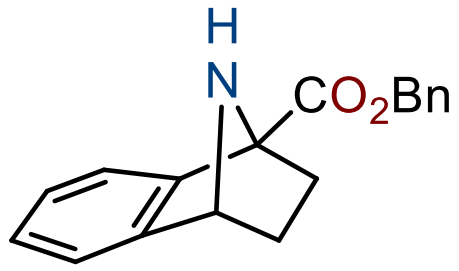

28

^{1}H NMR, 400 MHz, $CDCl_3$

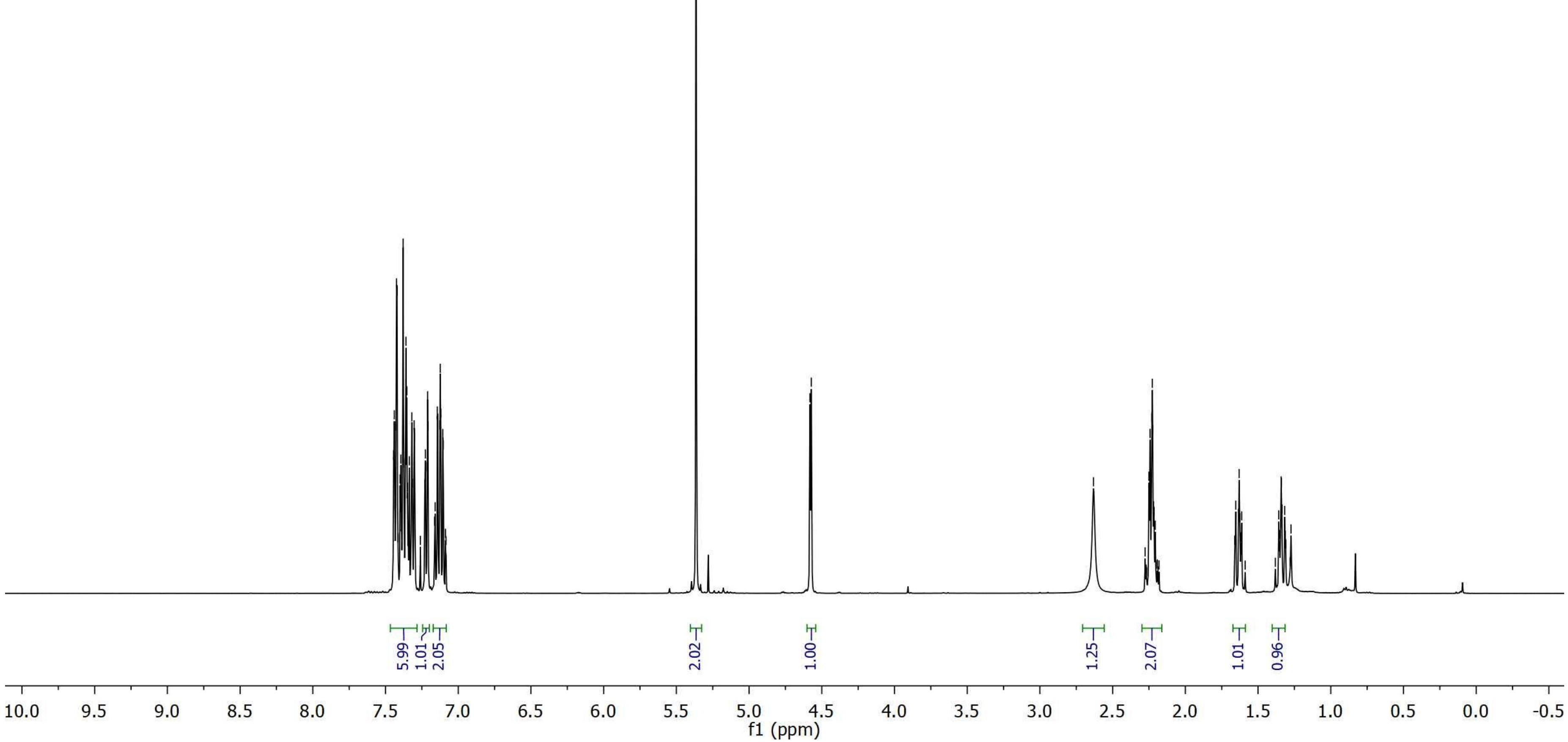

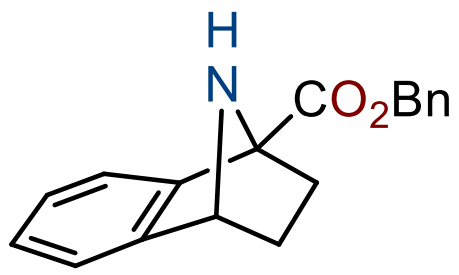

28

^{13}C NMR, 101 MHz, $CDCl_3$

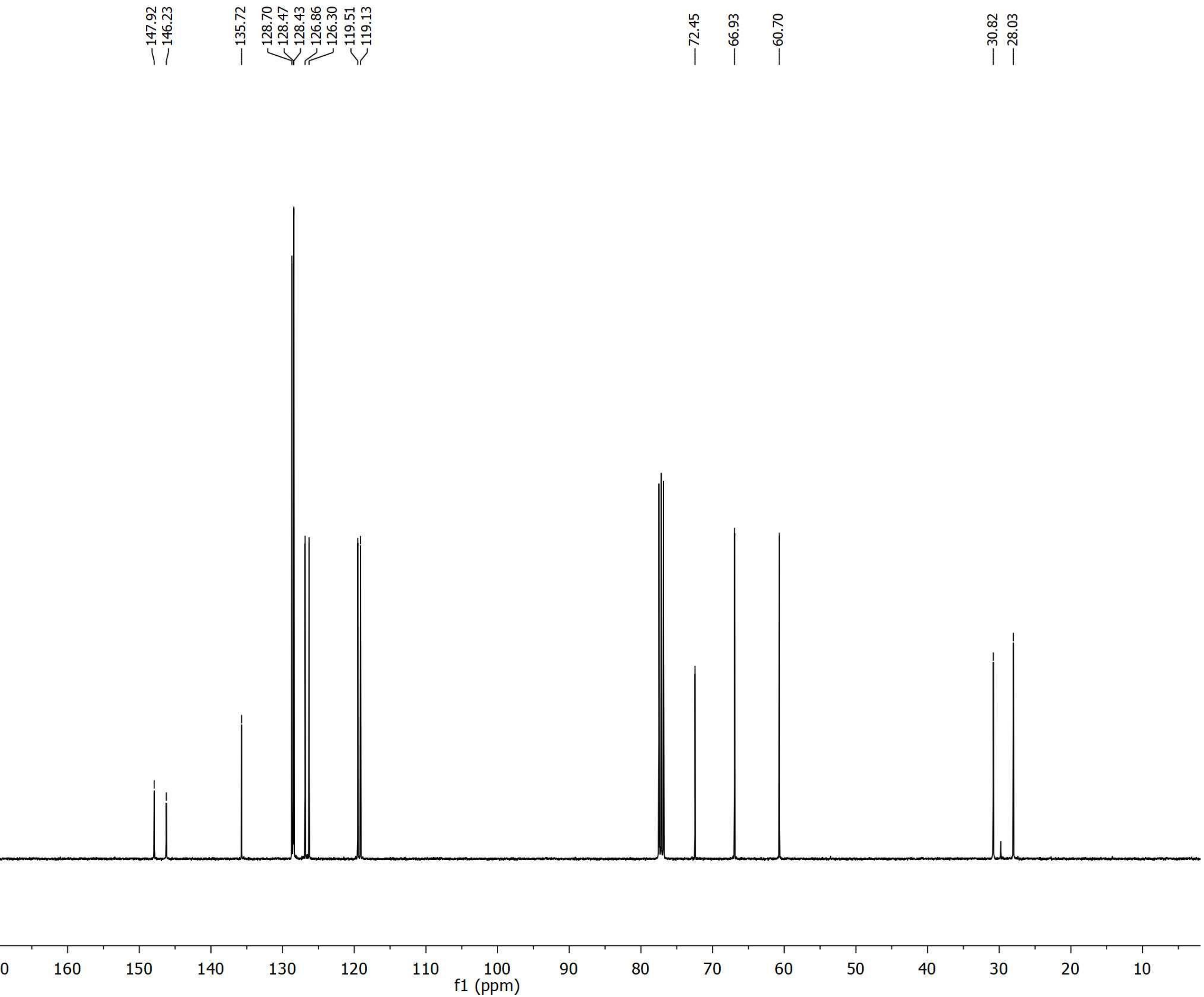

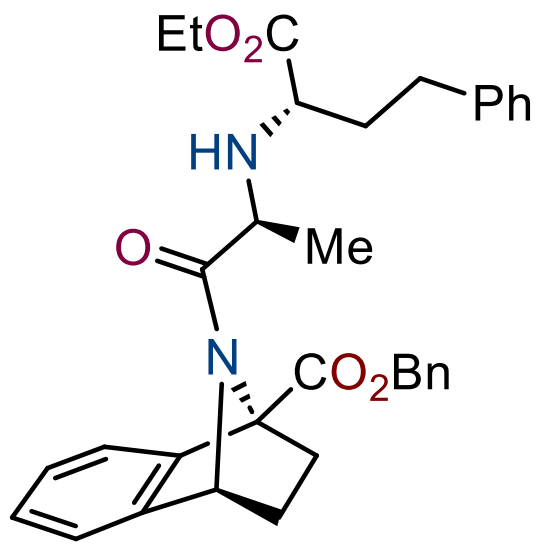

29A

^{1}H NMR, 400 MHz, CDCl$_3$

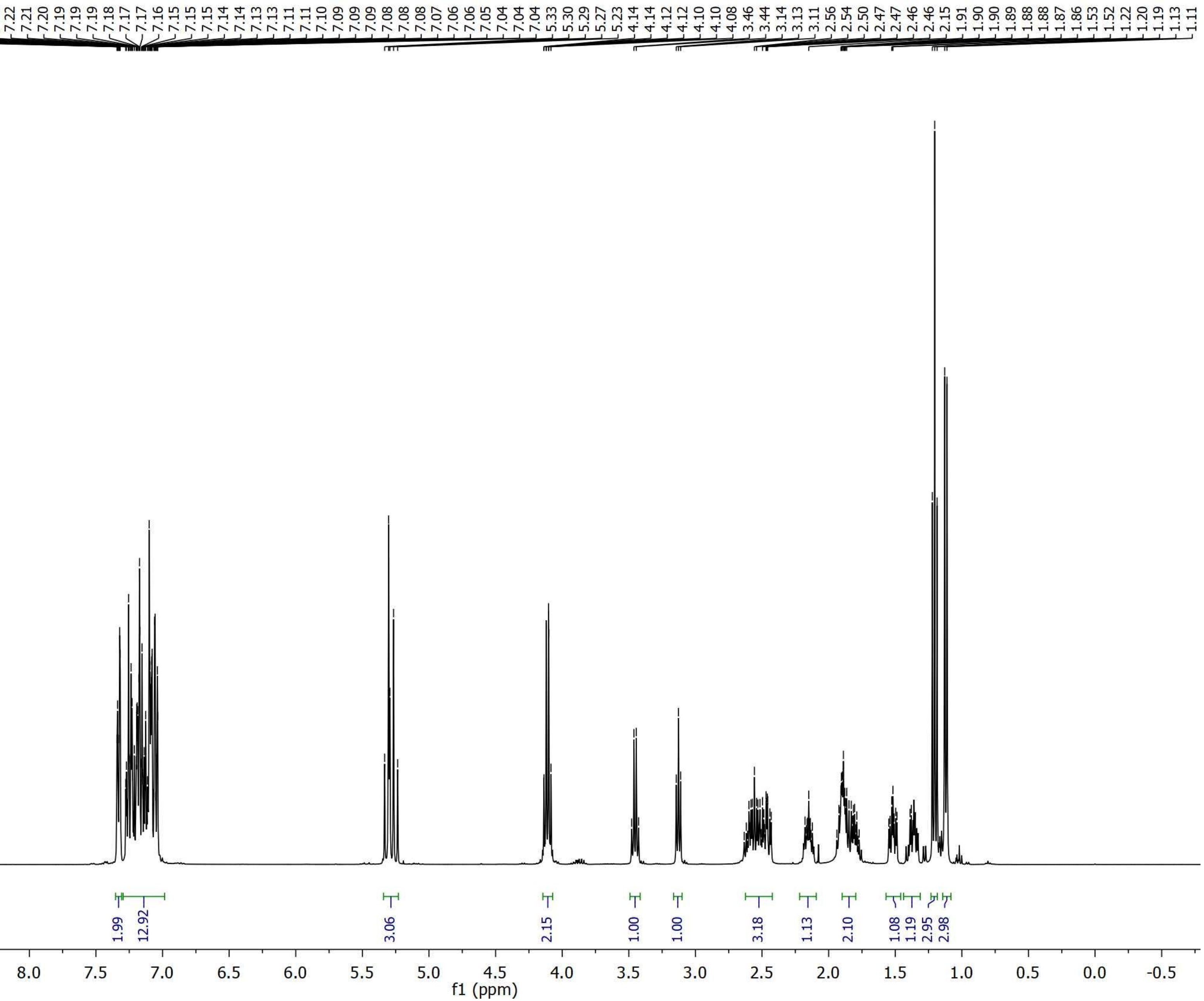

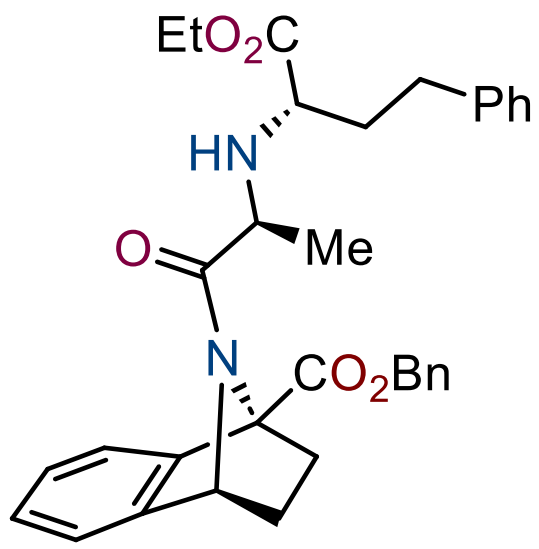

29A

^{13}C NMR, 101 MHz, $CDCl_3$

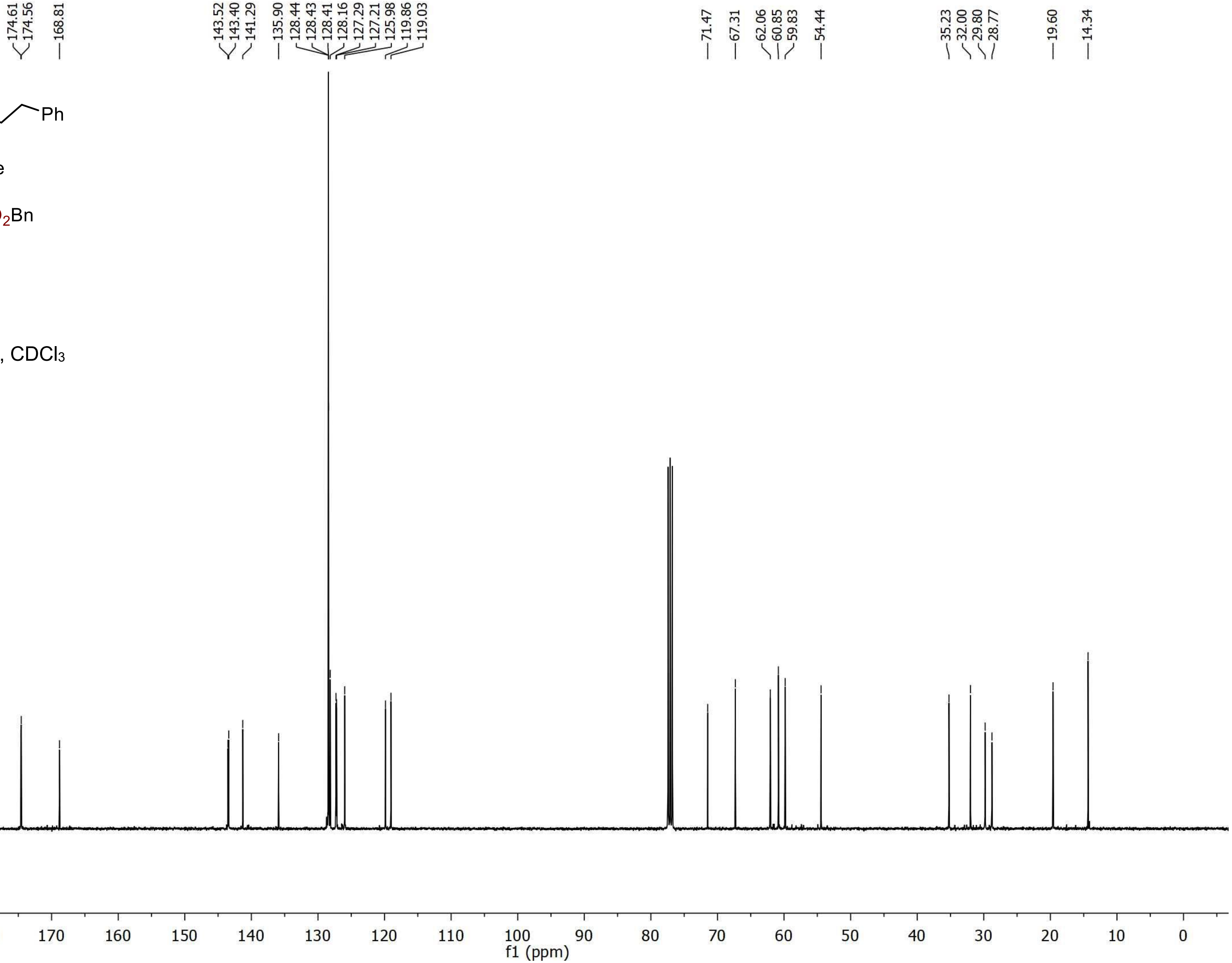

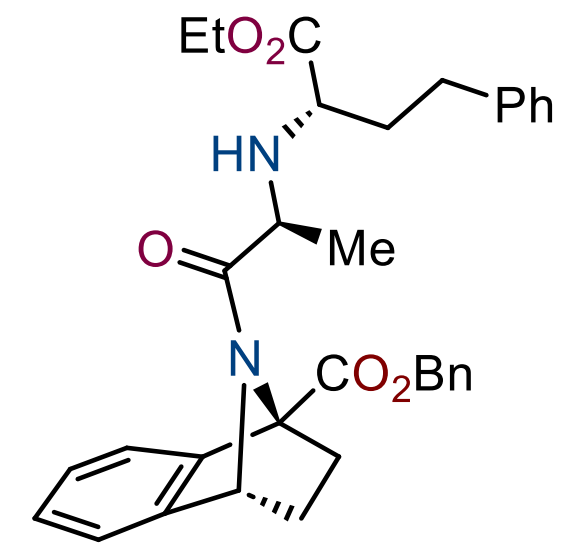

29B

^{1}H NMR, 400 MHz, $CDCl_3$

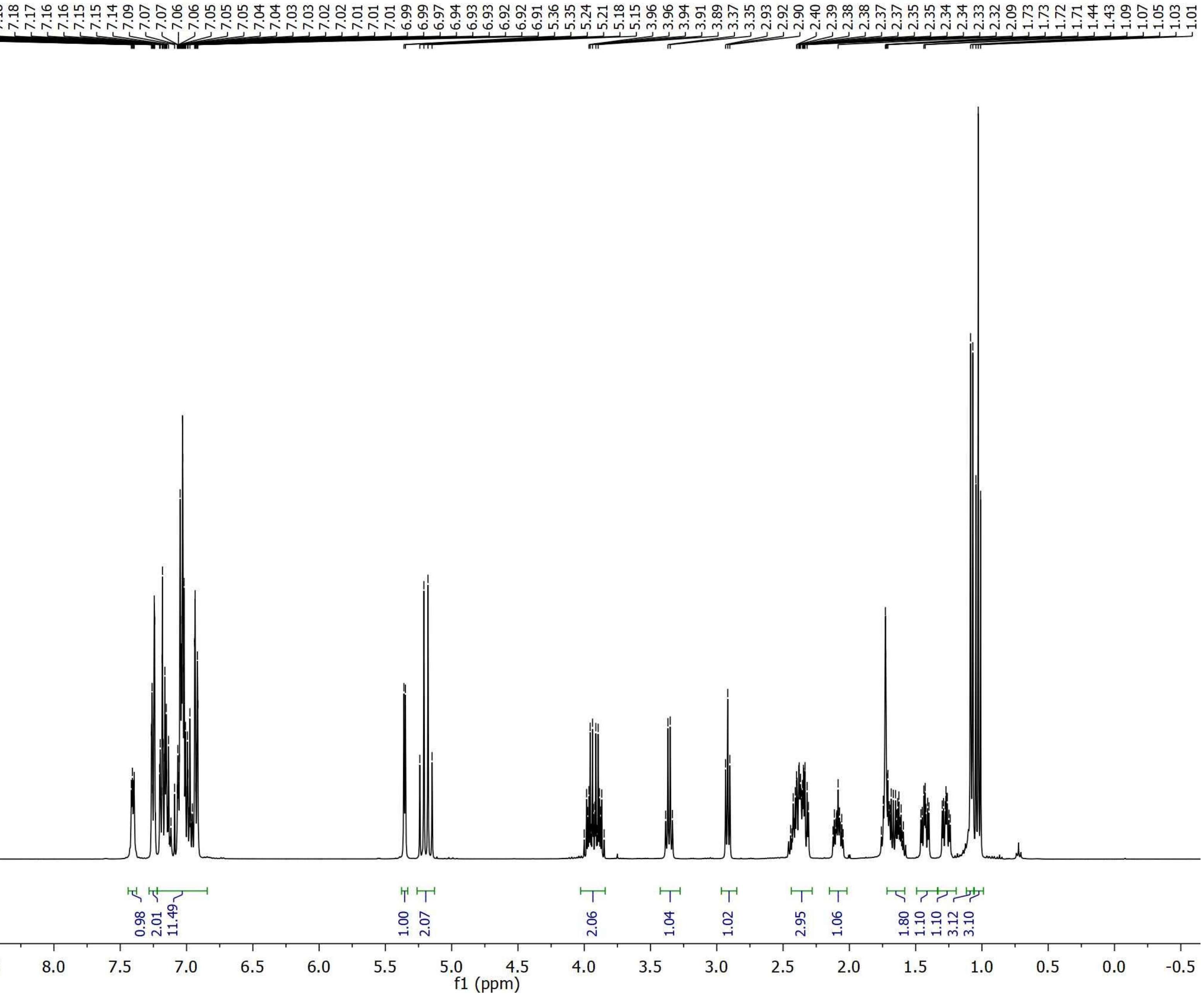

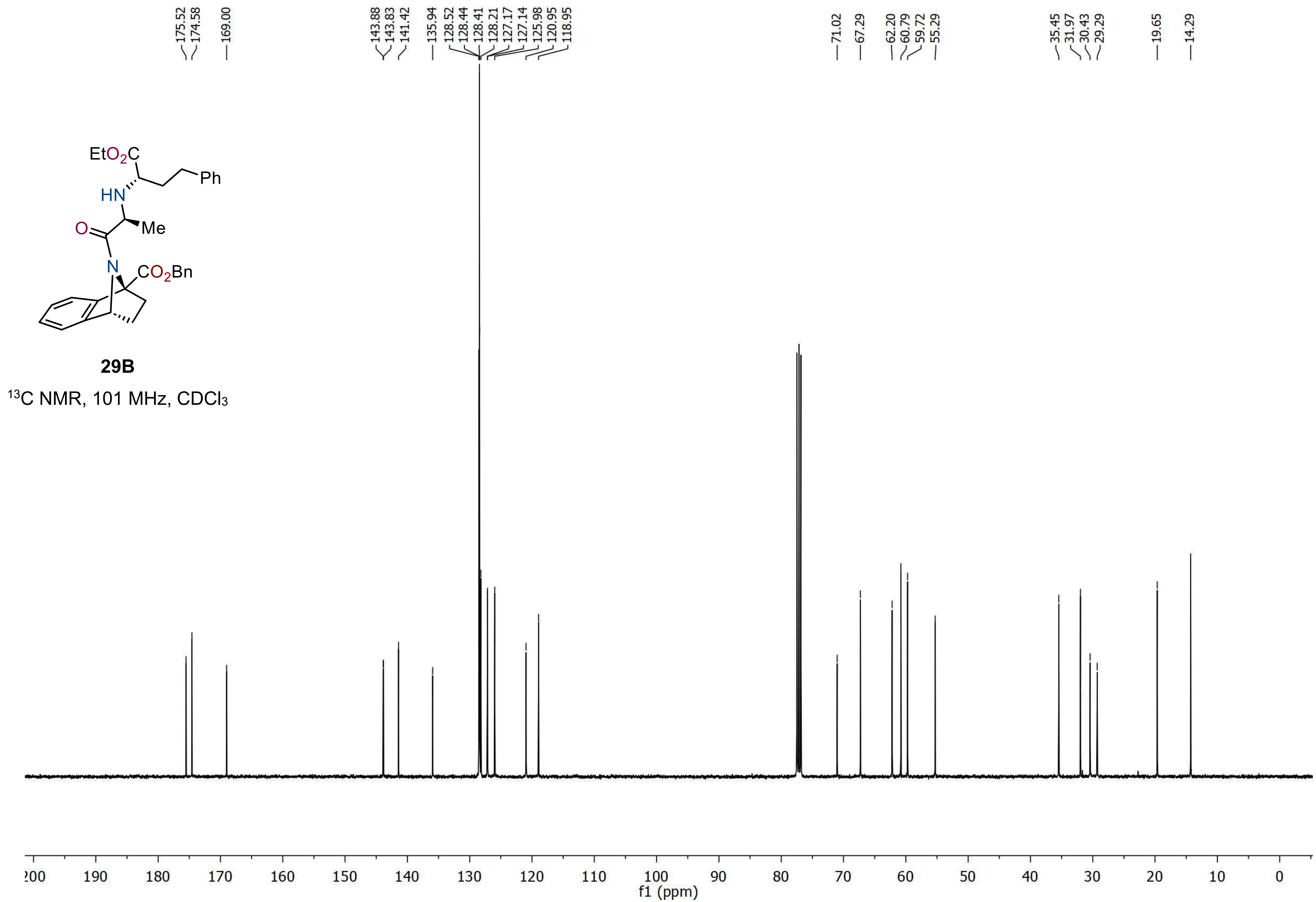

29B

^{13}C NMR, 101 MHz, $CDCl_3$

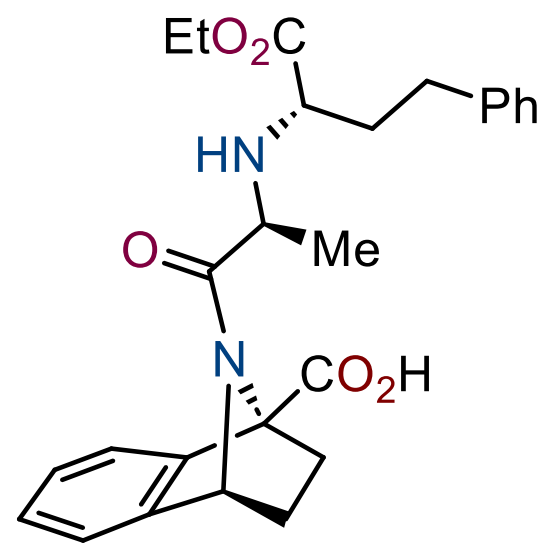

5A

^{1}H NMR, 400 MHz, MeOD

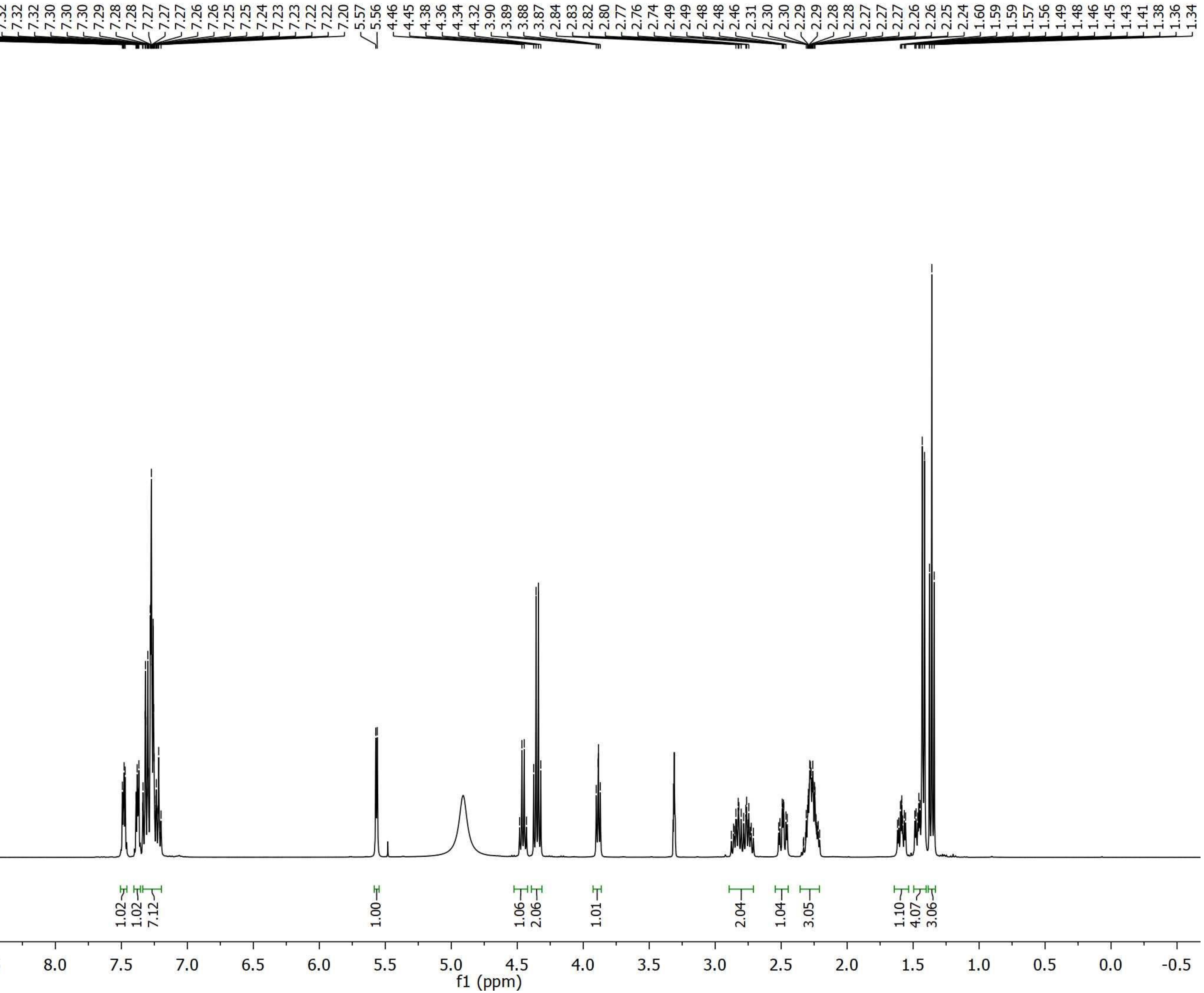

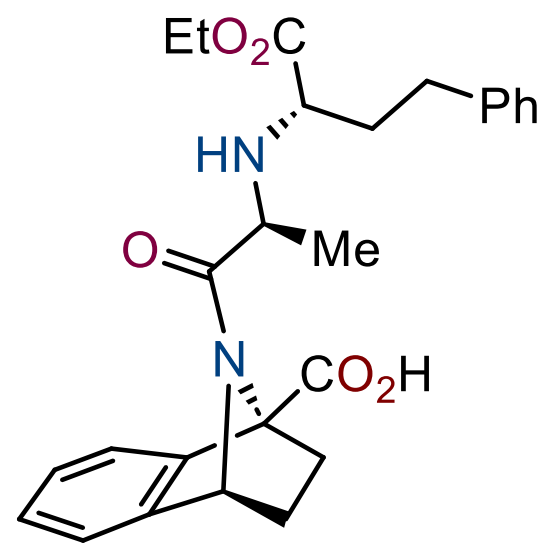

5A

^{13}C NMR, 101 MHz, MeOD

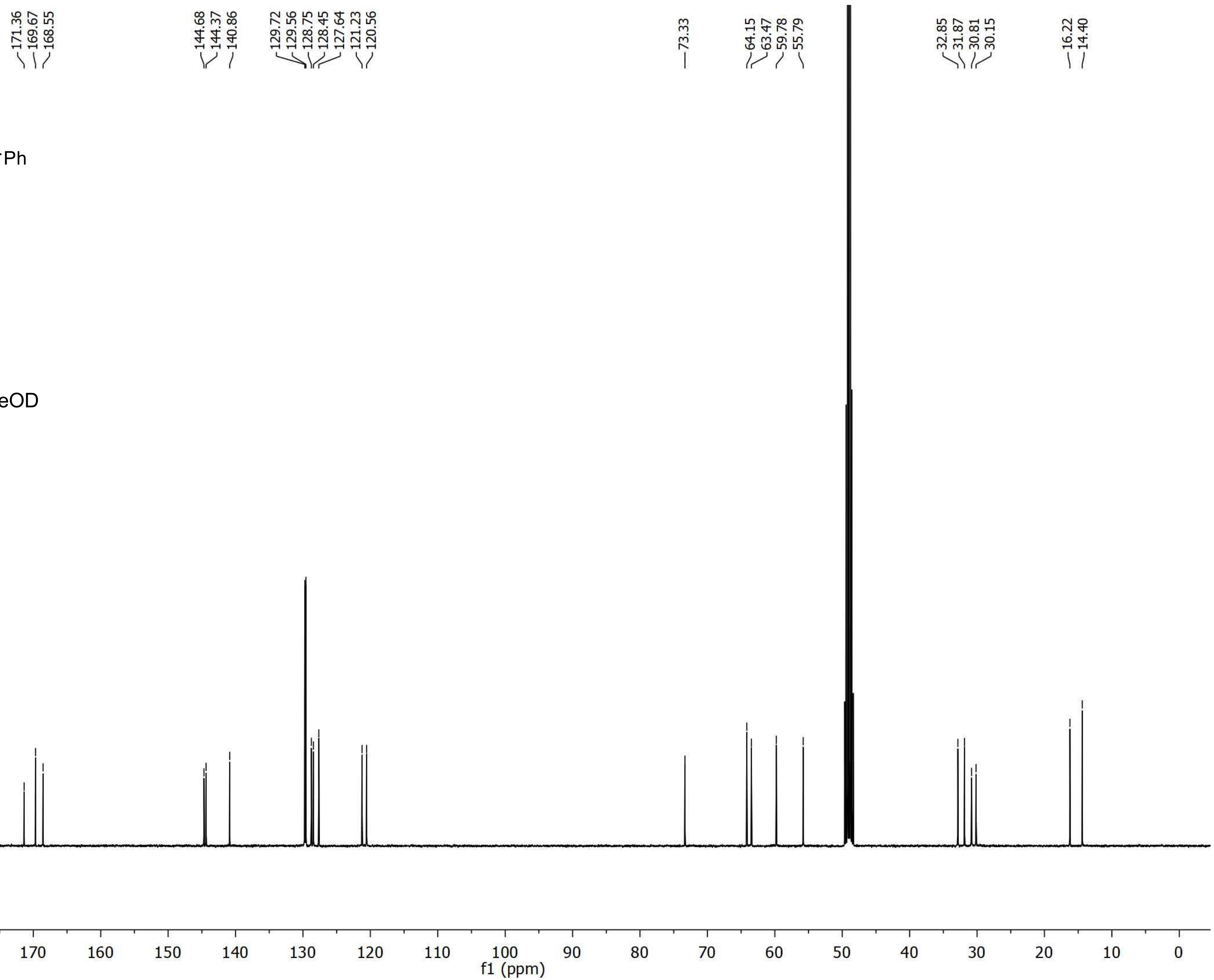

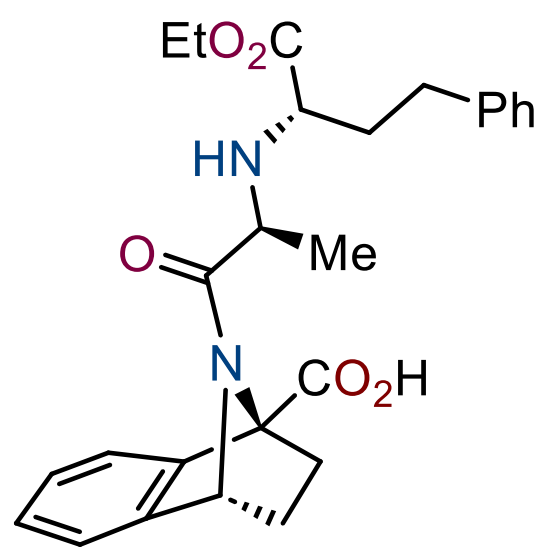

5B

^{1}H NMR, 400 MHz, $CDCl_3$

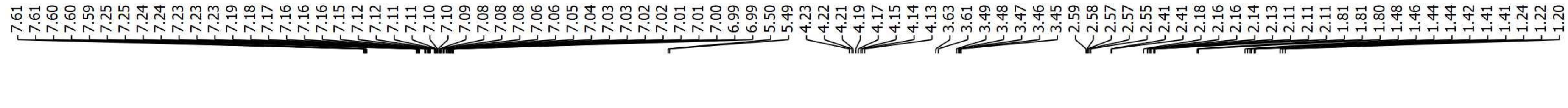

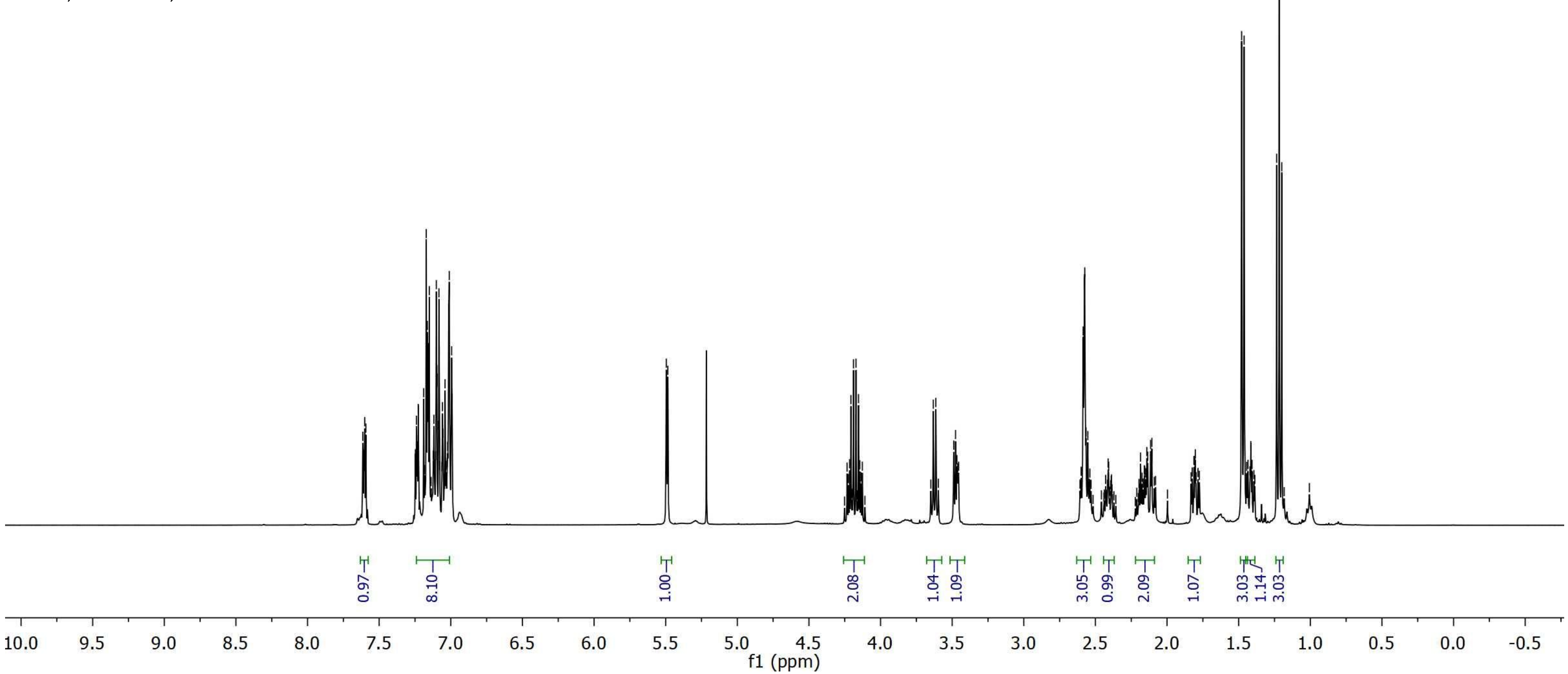

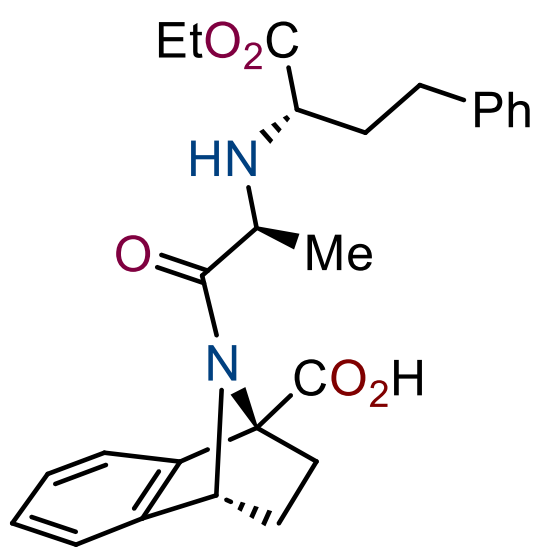

5B

^{13}C NMR, 101 MHz, $CDCl_3$

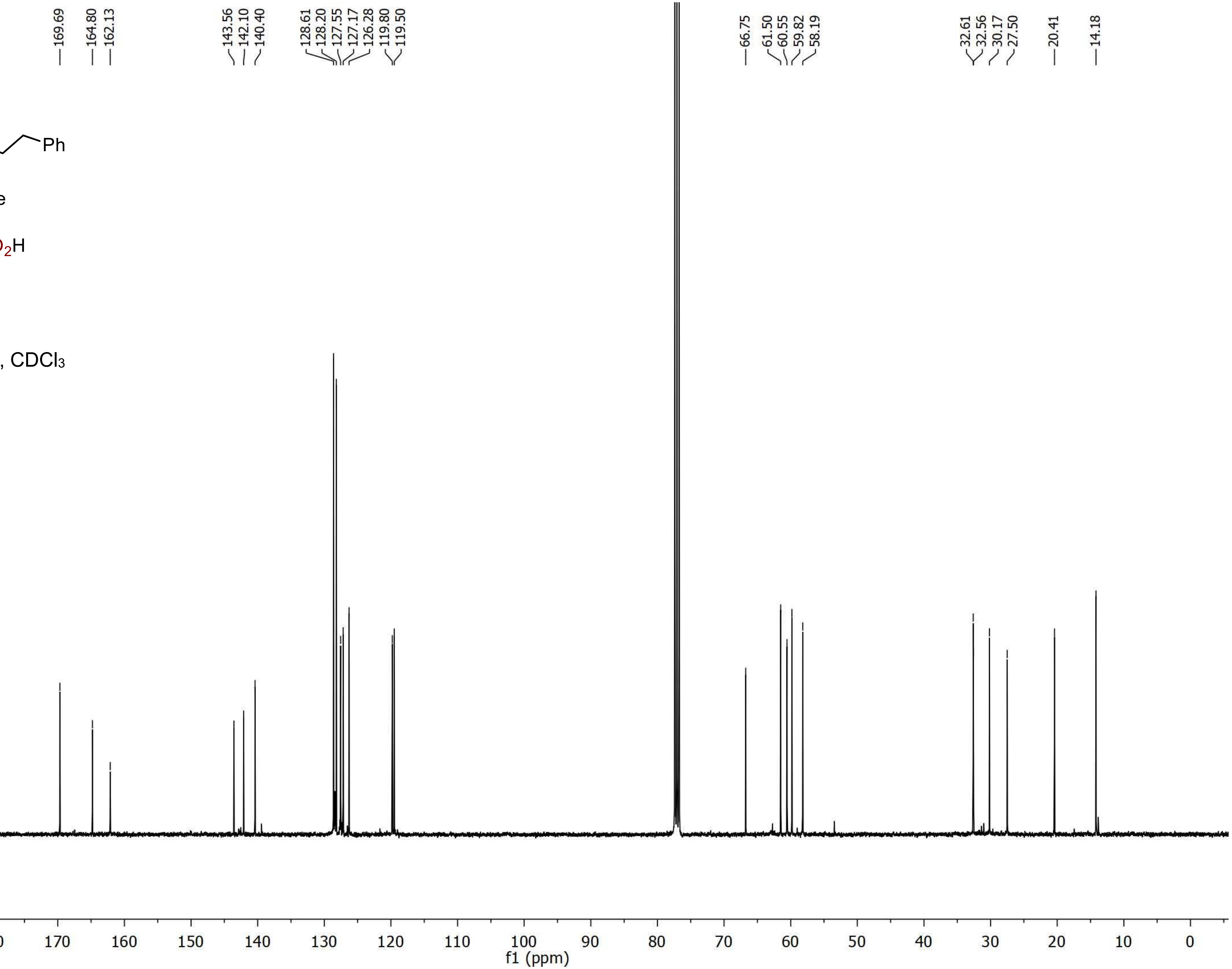

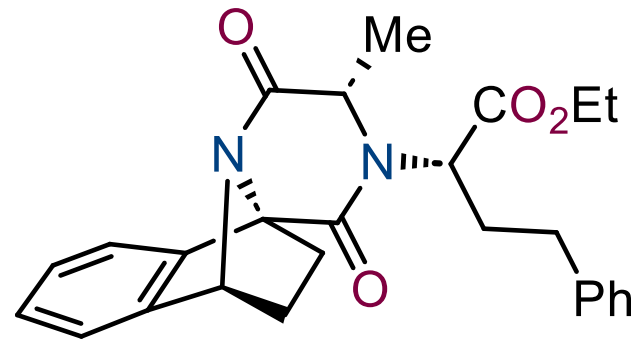

S22

^{1}H NMR, 400 MHz, $CDCl_3$

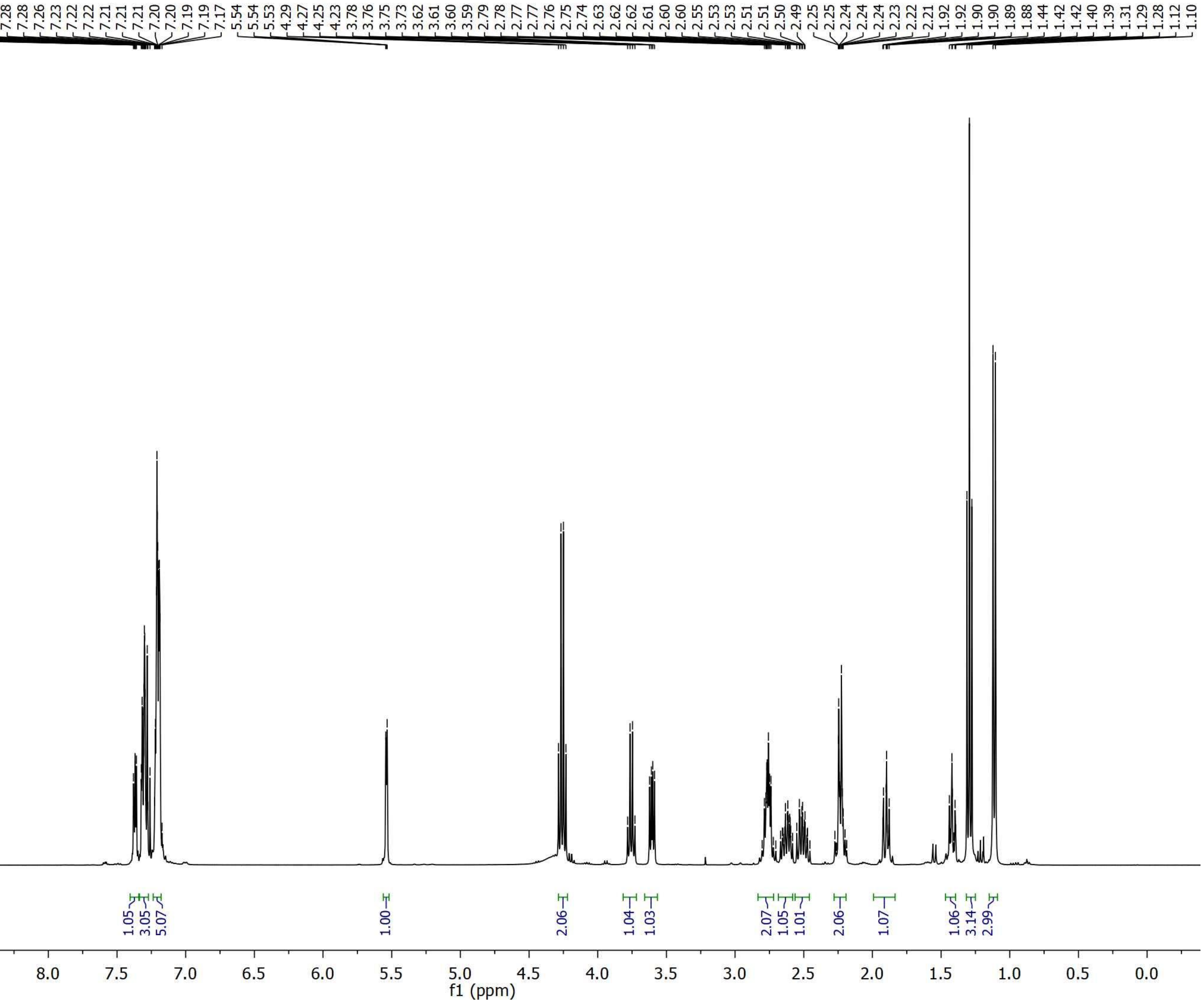

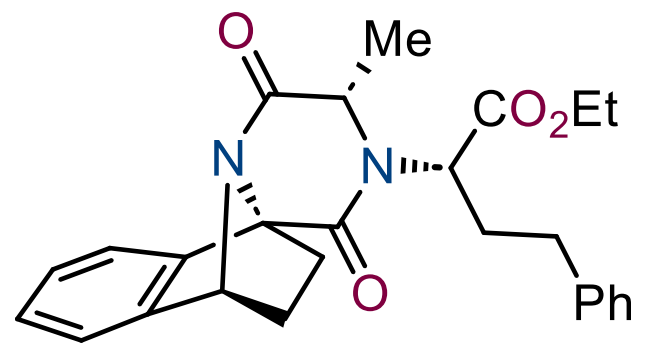

S22

^{13}C NMR, 101 MHz, $CDCl_3$

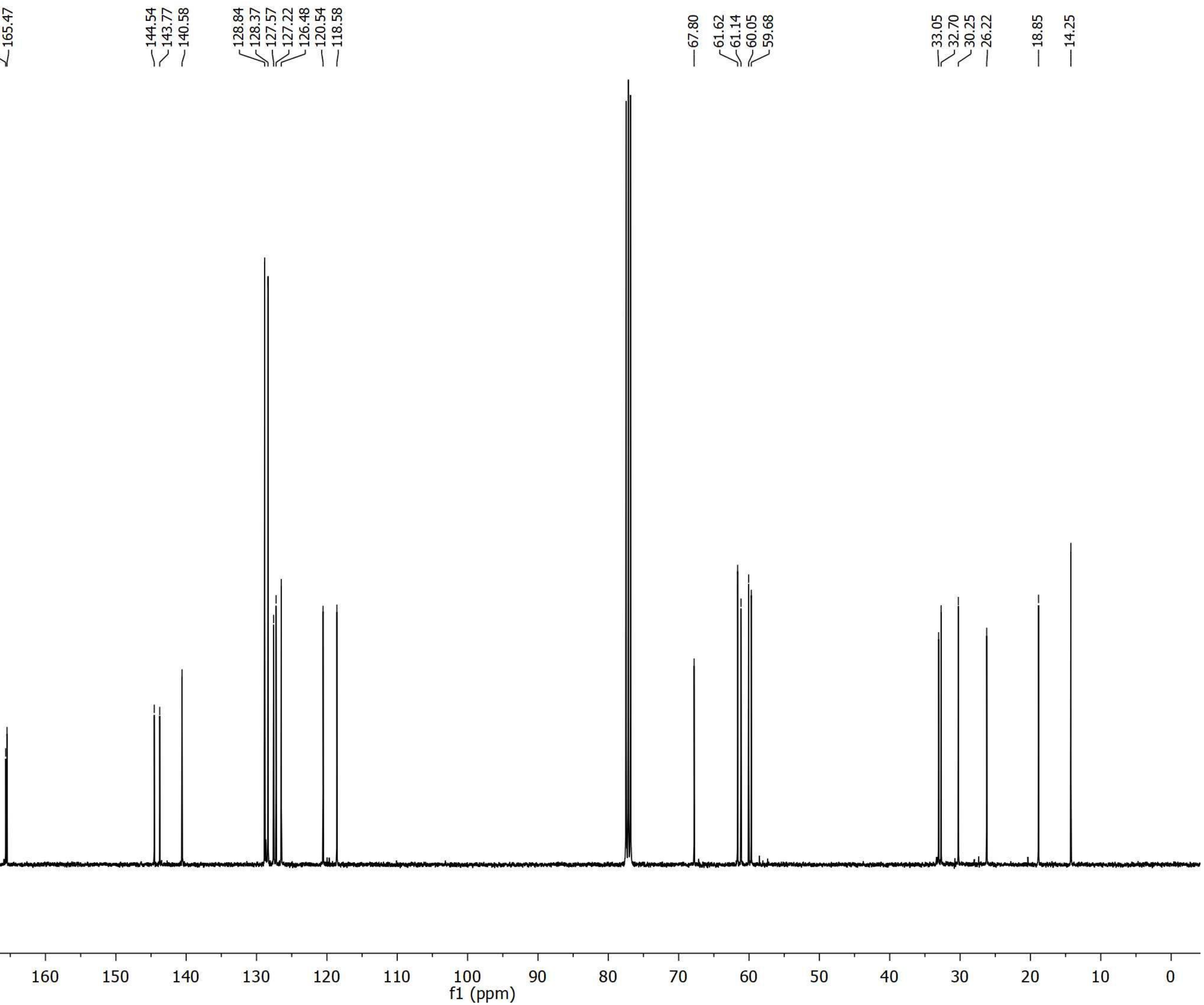

References

[1] D. Lücke, A. S. Campbell, M. Petzold, R. Sarpong, "Access to Naphthoic Acid Derivatives through a Oxabenzonorbornadiene Rearrangement" *Org. Lett*. **2023**, *25*, 7349.

[2] S. M. Bronner, N. K. Garg, "Efficient Synthesis of 2-(Trimethylsilyl)phenyl Trifluoromethanesulfonate: A Versatile Precursor to o-Benzyne" *J. Org. Chem*. **2009**, *74*, 8842.

[3] D. G. Batt, D. G. Jones, S. La Greca, "Regioselectivity in the acid-catalyzed isomerization of 2-substituted 1, 4-dihydro-1, 4-epoxynaphthalenes" *J. Org. Chem*. **1991**, *56*, 6704.

[4] M. Xu, J. Tu, R.M. Franzini, "Rapid and efficient tetrazine-induced drug release from highly stable benzonorbornadiene derivatives" *Chem. Commun*., **2017**, *53*, 6271.

[5] H. M. L. Davies, J. J. Matasi, G. Ahmed, "Divergent Pathways in the Intramolecular Reactions between Rhodium-Stabilized Vinylcarbenoids and Pyrroles: Construction of Fused Tropanes and 7-Azabicyclo[4.2.0]octadienes" *J. Org. Chem*. **1996**, *61*, 2305.

[6] R.N. Warrener, G.J. Collin, P.J. Foley, "7-(1-Acetoxymethylidene) Benzonorbornadiene: A Versatile Reagent for the Synthesis of 7-Formyl and 7-Hydroxymethyl Benzonorbornadienes" *Molecules* **2001**, *6*, 194.

[7] C. P. Breen, T. F. Jamison, "Continuous Flow Synthesis of ACE Inhibitors From N-Substituted L-Alanine Derivatives" *Chem. Eur. J*. **2019**, *25*, 14527.

[8] Rigaku Oxford Diffraction, 2021. CrysAlisPro Software system, version 1.171.41.104a, Rigaku Corporation, Wroclaw, Poland.

[9] A. Altomare, M.C. Burla, M. Camalli, G.L. Cascarano, C. Giacovazzo, A. Guagliardi, A.G.G. Moliterni, G. Polidori, R. Spagna, *J. Appl. Crystallogr*. **1999**, *32*, 115.

[10] G. M. Sheldrick, *Acta Crystallogr*. **2015**, *C71*, 3.
